# Maxent-directed field surveys identify new populations of narrowly endemic habitat specialists

**DOI:** 10.7717/peerj.3632

**Published:** 2017-07-31

**Authors:** Cody M. Rhoden, William E. Peterman, Christopher A. Taylor

**Affiliations:** 1 Illinois Natural History Survey, University of Illinois Urbana—Champaign, Champaign, IL, United States of America; 2 School of Environment and Natural Resources, Ohio State University, Columbus, OH, United States of America

**Keywords:** Arkansas, *Fallicambarus harpi*, Fine scale, Species distribution model, *Procambarus reimeri*, Ouachita mountains, Generalized linear model, Zero inflated, Crayfish

## Abstract

**Background:**

Rare or narrowly endemic organisms are difficult to monitor and conserve when their total distribution and habitat preferences are incompletely known. One method employed in determining distributions of these organisms is species distribution modeling (SDM).

**Methods:**

Using two species of narrowly endemic burrowing crayfish species as our study organisms, we sought to ground validate Maxent, a commonly used program to conduct SDMs. We used fine scale (30 m) resolution rasters of pertinent habitat variables collected from historical museum records in 2014. We then ground validated the Maxent model in 2015 by randomly and equally sampling the output from the model.

**Results:**

The Maxent models for both species of crayfish showed positive relationships between predicted relative occurrence rate and crayfish burrow abundance in both a Receiver Operating Characteristic and generalized linear model approach. The ground validation of Maxent led us to new populations and range extensions of both species of crayfish.

**Discussion:**

We conclude that Maxent is a suitable tool for the discovery of new populations of narrowly endemic, rare habitat specialists and our technique may be used for other rare, endemic organisms.

## Introduction

Understanding the factors influencing species distributions and habitat selection are critical to researchers (Baldwin, 2009) because rare species or those with small native ranges (defined herein as those occurring in a single river drainage or a 1,000 sq. km area), are difficult to monitor and conserve when their total distribution and habitat preferences are not completely known. These problems can be addressed using species distribution models (SDMs), which are correlative models using environmental and/or geographic information to explain observed patterns of species occurrences (Elith & Graham, 2009). SDMs can provide useful information for exploring and predicting species distributions across the landscape (Elith et al., 2011). Models estimated from species observations can also be applied to produce measures of habitat suitability (Franklin, 2013). This information can be useful for detecting unknown populations of rare, endemic, or threatened species (e.g., Williams et al., 2009; Rebelo & Jones, 2010; Peterman, Crawford & Kuhns, 2013; Searcy & Shaffer, 2014; Fois et al., 2015). SDMs can also limit search efforts by selecting suitable sampling areas *a priori*, leading to a cost-effective and efficient use of sampling effort (Fois et al., 2015).

One of the most widely used SDMs in recent years is the program Maxent (Kramer-Schadt et al., 2013). Maxent is a presence-only modeling algorithm using predictor variables such as climatic and remotely sensed variables (Phillips, Anderson & Schapire, 2006; Phillips & Dudík, 2008). These data are used to predict the relative occurrence rate (ROR) of a focal species across a predefined landscape (Fithian & Hastie, 2013). Recent studies focusing on the performance of Maxent have revealed it to perform well in comparison to other SDMs (Elith et al., 2006). Maxent also performs well with small sample sizes (Pearson et al., 2007; Wisz et al., 2008), rare species (Williams et al., 2009; Rebelo & Jones, 2010), narrowly endemic species (Rinnhofer et al., 2012), and when used as a habitat suitability index (Latif et al., 2015).

However, the potential for the inaccurate execution and interpretation of an SDM is well documented (Baldwin, 2009; Syfert, Smith & Coomes, 2013; Fourcade et al., 2014; Guillera-Arroita, Lahoz-Monfort & Elith, 2014). Specific issues surrounding the interpretation of Maxent analyses include sampling bias (Phillips & Dudík, 2008; Syfert, Smith & Coomes, 2013; Fourcade et al., 2014), the lack of techniques to assess model quality (Hijmans, 2012), overfitting of model predictions (Elith, Kearney & Phillips, 2010; Warren & Seifert, 2011), or assessment of detection probabilities (Lahoz-Monfort, Guillera-Arroita & Wintle, 2014). Researchers have sought to solve the aforementioned issues by reducing sampling bias through spatial filtering (Boria et al., 2014), assessing model quality with a null model approach (Raes & Ter Steege, 2007), utilizing the R package ENMeval (Muscarella et al., 2014) to balance goodness-of-fit and model complexity, and collecting data informative about imperfect detectability (Lahoz-Monfort, Guillera-Arroita & Wintle, 2014). The utility of Maxent has also been burdened with issues of model validation (Hijmans, 2012). Most model validation methods involve subsets of the input data with the predictions generated by the models (Rebelo & Jones, 2010). Historically, validation of Maxent predictions has lacked an independent assessment of model performance (Greaves, Mathieu & Seddon, 2006), such as a novel set of presence locations. Recent studies have found ground validation of Maxent has been a suitable method to determine the accuracy of predictions (Stirling et al., 2016). The need for independent validation is especially important for rare species exhibiting a wider knowledge gap in distribution than more common species (Rebelo & Jones, 2010). For example, North American primary burrowing crayfishes are a poorly understood and understudied taxon for which SDMs could provide novel insight into distributions and habitat relationships and thus provide an excellent case study for validation of SDMs.

North America has the highest diversity of crayfishes worldwide (Taylor et al., 2007). Within North America, 22% of the species listed as endangered or threatened in a conservation review of crayfishes were primary burrowing crayfishes (Taylor et al., 2007). It is hypothesized all crayfishes have the ability to construct refugia by way of burrowing down into the soil or substrate (Hobbs Jr, 1981; Berrill & Chenoworth, 1982). Primary burrowing crayfishes differ from stream dwelling crayfishes in their life history traits, they spend most of their life cycle underground, leaving their burrows only to forage and find a mate (Hobbs Jr, 1981). This difference in life history traits allow primary burrowing crayfish to persist in areas that are not connected to above-ground sources of water. This persistence allows primary burrowing crayfish to use habitats such as seeps, perched wetlands, and even roadside ditches.

Amongst the three types of burrowers, the least is known regarding the natural history of primary burrowing crayfishes (Taylor et al., 2007; Moore, DiStefano & Larson, 2013) due to the challenges in sampling these largely fossorial animals (Larson & Olden, 2010). However, the narrowly endemic nature of North American crayfishes is well documented (Page, 1985; Taylor et al., 2007; Simmons & Fraley, 2010; Morehouse & Tobler, 2013). Primary burrowing crayfishes in Arkansas are no exception (Robison et al., 2008). Of the 12 species of primary burrowers in Arkansas (*Fallicambarus dissitus, F. fodiens, F. gilpini, F. harpi*, *F. jeanae, F. petilicarpus, F. strawni*, *Procambarus curdi, P. liberorum*, *P. parasimulans*, *P. regalis,* and *P. reimeri*) six (50%) are known from only one ecoregion. The limited geographic distribution of any taxa makes them more vulnerable to localized extirpation. Because these animals occur at such a constrained geographic scale, it is important to understand and document their existing distribution to manage and preserve current populations.

The rarity of and difficulties surrounding the collection of natural history information, specifically habitat suitability, make primary burrowing crayfishes ideal candidates for SDMs. To test the ability of Maxent to predict the distribution of suitable habitat for two narrowly endemic habitat specialists, we constructed SDMs for *Fallicambarus harpi* and *Procambarus reimeri* and validated the models using sampling data collected after completion of each SDM. These species are vulnerable to population declines and are currently recorded under the Endangered (*P. reimeri*) and Vulnerable (*F. harpi*) conservation status categories (Taylor et al., 2007) based on modifications to or reductions of habitat in their already restricted ranges. Both crayfishes are endemic to the Ouachita Mountains Ecoregion (OME; Woods et al., 2004), which is characterized by remnant pine-bluestem (*Pinus-Schizachyrium*) communities and silty loam soil (Hlass, Fisher & Turton, 1998). We used these two narrowly endemic species to reinforce the performance of Maxent with small sample sizes and rare species along with addressing problems associated with Maxent to maximize the accuracy of our predictions. We also sought to investigate the use of Maxent to identify suitable habitat and locate new occurrences of both species of crayfish.

## Materials and Methods

### Presence data and environmental variables

To determine habitat requirements of *F. harpi* and *P. reimeri*, we queried natural history museums or databases (Illinois Natural History Survey Crustacean Collection, the National Museum of Natural History Smithsonian Institution, and the Arkansas Department of Natural Heritage) for historic locations of both species, and a subset of those locations were visited in 2014 (Rhoden, Taylor & Peterman, 2016). We sampled only those sites with confirmed presences in the past 20 years that were not based on obvious misidentifications occurring well outside of the known range of each species known in 2014 and were able to be located given historical information associated with respective collection events. At each location, we measured habitat variables hypothesized to determine burrow placement: percent tree canopy cover, percent herbaceous ground cover, stem density, the number of burrows, presence of standing water at the site, remotely sensed variables and the presence or absence of hydrophilic sedges. We found canopy cover and the presence of hydrophilic sedges were the most important factors in predicting crayfish abundance (Rhoden, Taylor & Peterman, 2016).

**Table 1:** Environmental variables. Description, origin, resolution, general statistics, and units of environmental variables used in the Maxent analysis of two primary burrowing crayfish species (*Fallicambarus harpi* and *Procambarus reimeri*) in western Arkansas.

Variable	Description	Source	Resolution	Min/max(unit)	µ(sd)
Canopy cover	Percent tree canopy cover	National Land Cover Database 2011 USFS	30 m	0/100(% cover)	52.23(43.48)
Elevation	Digital elevation model of the study site	USGS National Elevation Dataset	10 m	50.50/818.96 (m)	229(100.85)
Distance to nearest waterbody	Euclidean distance to nearest permanent waterbody across the study site	ESRI Spatial Analyst Tools; National Hydrology Dataset composed of stream segments of study site	10 m	0/1740.26(m)	187.48(161.04)
Compound topographic index	A function of slope and the upstream contributing area per unit width orthogonal to the flow direction (Evans et al., 2010)	ArcGIS Geomorphometry and Gradient Metrics Toolbox 2.0 (Evans et al., 2010); National Elevation Dataset	10 m	2.67/27.58(index value)	7.58(1.94)
Solar radiation	Incoming solar radiation value (watt hours per m^2^) based on direct and diffuse insolation from the unobstructed sky directions	ESRI Spatial Analyst Tools; National Elevation Dataset	10 m	3542.41/64134 (watt hours/m^2^)	5955.14(116.01)

The presence locations used for the Maxent analysis, based on the field surveys of 2014, consisted of 58 locations for *F. harpi* (of which 56 were used for the SDM analysis) and 53 locations for *P. reimeri* (of which 50 were used for the SDM analysis). To minimize spatial autocorrelation, a subset of the original presence data was used. All duplicate presence locations falling within the same cell of a 30 m resolution raster were removed before the SDM analysis. The selected presence locations used for the SDM analysis were near (<90 m) primary, secondary, and tertiary roadways. The environmental variables used for the SDM analysis consisted of canopy cover, elevation, distance to nearest waterbody, compound topographic index value (CTI), and solar radiation value (Table 1). These habitat variables reflect habitat characteristics associated with *F. harpi* (Robison & Crump, 2004) and *P.* *reimeri* (Robison, 2008), and other primary burrowing crayfish species (Hobbs Jr, 1981; Welch & Eversole, 2006; Loughman, Simon & Welsh, 2012). Canopy cover was estimated using a United States Forest Service percent canopy raster (National Land Cover Database 2011; 30 m). Elevation was estimated using a United States Geological Survey digital elevation map (DEM; 10 m). Distance to nearest water body was estimated by constructing a raster of the Euclidean distance from all permanent waterbodies. Compound topographic index values were determined using the Geomorphometry and Gradient Metrics (version a1.0; Evans et al., 2010) toolbox; this metric is a representation of surface wetness across the landscape (Evans et al., 2010). CTI is a steady state wetness index, where a larger CTI value represents areas topographically suitable for water accumulation. We measured solar radiation by calculating the watt-hour/m^2^ of the delineated sampling area using the Area Solar Radiation tool in ArcMap (Table 1). These values were calculated using digital elevation maps (National Elevation Dataset: http://ned.usgs.gov/, accessed 07/21/2014) and surface water maps (National Hydrography Dataset: http://nhd.usgs.gov/index.html, accessed 07/21/2014). The entire OME was used as a delineation for both species of crayfish in the SDM analysis. Each surface was resampled to a common resolution of 30 m to match the resolution of the canopy surface.

### Maxent analysis

We created species-specific distribution models using Maxent (version 3.3k; Phillips, Anderson & Schapire, 2006). For each species, we generated 2,500 random background points within 10 km^2^ polygons that were situated around the historic museum localities for which we confirmed species presence in the field in 2014. This approach follows Peterman, Crawford & Kuhns (2013) and was implemented to reduce model bias described by Phillips (2008). We fit a full model for each species, and used the ENMeval package (Muscarella et al., 2014) in program R (R 3.1.1; R Development Core Team, 2014) to tune the Maxent model parameter settings minimizing the SDM model AICc. ENMeval automatically executes Maxent across a range of settings and outputs evaluation metrics to aid in identifying settings balancing model fit and predictive ability (Muscarella et al., 2014). Using jackknife and the standard settings, this analysis suggested the *F. harpi* model should be fit with a betamultiplier of 2.5 and linear, quadratic, and hinge features, and the *P. reimeri* model should be fit with a betamultiplier of 1.5 and linear, quadratic, and hinge features to provide the most parsimonious fit to our data. We then re-ran each species’ model using the refined regularization multiplier and feature classes to increase the rigor in building and evaluating the distribution model for each species based on presence only data.

We assessed the performance of the tuned models using the null model approach of Raes & Ter Steege (2007) with package ENMtools (Warren, Glor & Turelli, 2010). We generated two groups of 999 random data sets containing 56 and 50 samples, which correspond to the number of presence locations used for *F. harpi* and *P. reimeri* (respectively) in the initial model. These points were drawn without replacement from the OME delineation used in the initial model. Both model Area Under the Curve (AUC) values were compared to the 95th percentile of the null AUC frequency distribution.

The final Maxent models were calculated with the maximum number of iterations set to 5,000 and the analysis of variable importance was measured by jackknife and response curves. The form of replication used was bootstrap. These settings, the refined regularization multiplier and feature classes, and the recommended default values were used for our final Maxent model runs. Due to the endemic nature of both species and the small amount of presence locations in the initial model, we did not include a bias file or spatial filtering.

### Field sampling and validation

**Figure 1:**
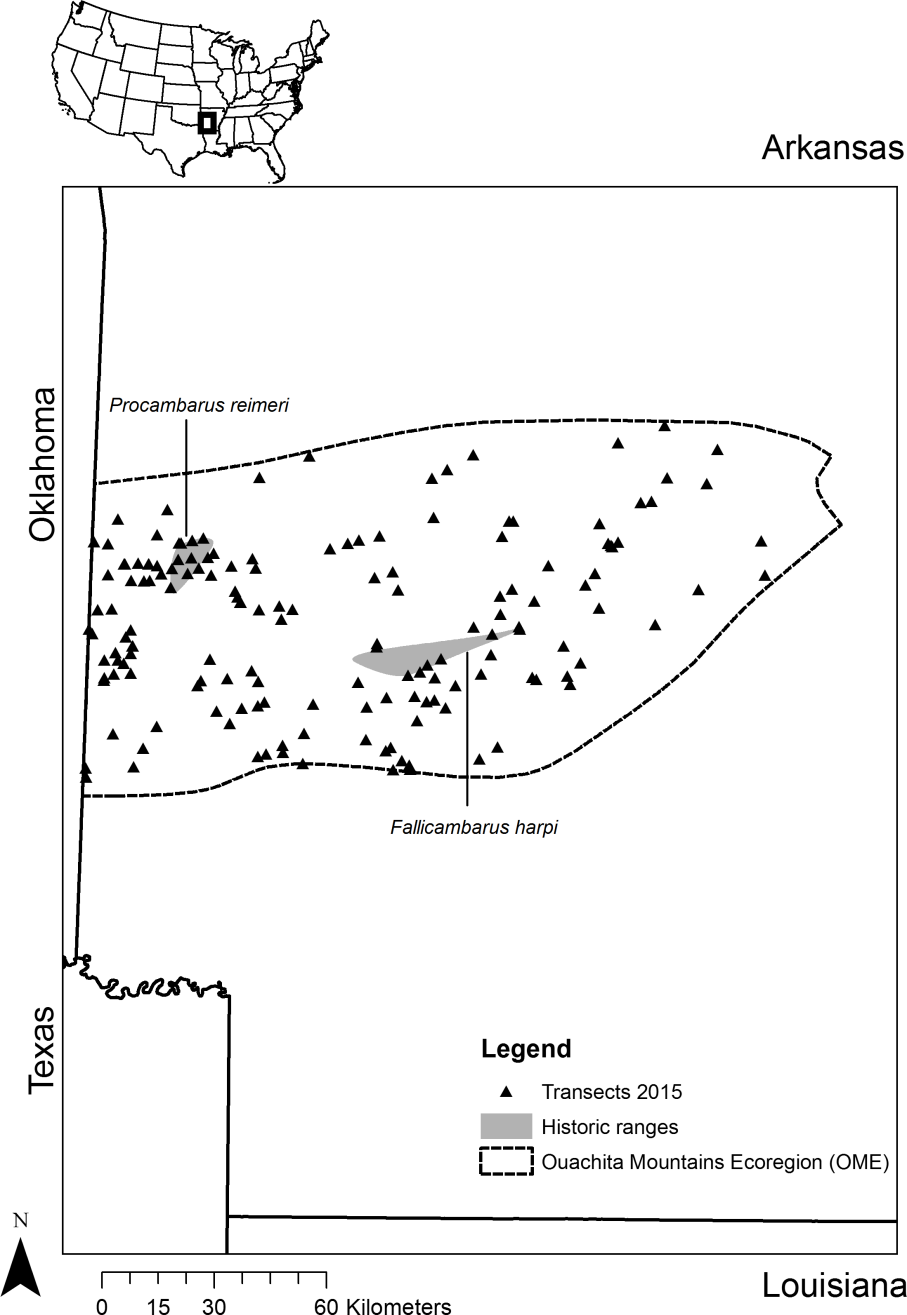
Sampling sites. Map depicting the location of sites sampled in western Arkansas in the spring of 2015 based on the predictions from a Maxent analysis of two primary burrowing crayfish species (*Fallicambarus harpi* and *Procambarus reimeri*).

The refined Maxent models (one for each species) were used to select 80 semi-random sampling sites for each species within the OME. These sites were semi-random because we restricted our sampling to areas of public access (roadside ditches). We sampled thirteen counties encompassing the known range of both species of primary burrowing crayfish: from east to west, those counties were Pulaski, Saline, Perry, Garland, Hot Spring, Clark, Yell, Montgomery, Pike, Scott, Howard, Polk, and Sevier (Fig. 1). The Maxent output for both species was discretized into four categories based on the relative occurrence rate (ROR; Fithian & Hastie, 2013). The Maxent output is considered a relative occurrence rate because the presence data are proportional but not equal to occurrence. The first category ranged from an ROR of 0 to the lowest presence threshold (LPT = minimum training presence threshold of Maxent software; Wisz et al., 2008) of each species. The LPT is the smallest logistic value associated with one of the observed species localities. The second class ranged from the LPT to 50% of the maximum ROR of each species. The third category ranged from 50% of the maximum ROR to 75% of the maximum ROR of each species. The fourth category ranged from 75% of the maximum ROR to the maximum ROR of each species.

The final Maxent model outputs for both species were placed into the described categories in ArcMap. The projection of the Maxent model onto the environmental variables was converted into polygons in ArcMap, which represented each category. Any polygon representing a single pixel or island (one 30 m × 30 m area in original output raster) was removed. All category polygons were then overlaid with a layer representing public right of ways and other public areas (state parks, natural areas, etc.).

We generated 40 random points in each category polygon using the final polygon layer. All points within each category polygon had a spatial buffer of 2 km and were checked before sampling to ensure accessibility. If a point was inaccessible in the field, the next closest accessible point within the respected category was chosen and sampled. To assess the accuracy of the Maxent predictions, we calculated the receiver operating characteristic (ROC) and the AUC for the average ROR of occupied transects vs. the average ROR of unoccupied transects (Fawcett, 2006) with the pROC package in program R (Robin et al., 2011). A ROC graph is a technique for visualizing, organizing and selecting classifiers based on their performance (Fawcett, 2006), and displays the performance of a binary classification method (presence/absence) with a continuous (Maxent prediction) ordinal output (Robin et al., 2011). Furthermore, the ROC plot shows the sensitivity (proportion of correctly classified positive observations) and specificity (the proportion of correctly classified negative observations) as the output threshold is moved over the range of all possible values (Robin et al., 2011).

To assess how the number of burrows encountered on a transect related to habitat variables and ROR, we fit zero-inflated (package:pscl; Zeileis, Kleiber & Jackman, 2008) and negative binomial generalized linear models (package:MASS; Venables & Ripley, 2002) for *F. harpi* and *P. reimeri*, respectively. Zero-inflated models were fit for both species, however model selection suggested that the negative binomial was a better fit for the *P. reimeri* data. The response variable was the number of burrows in each transect for the *F. harpi* and *P. reimeri* models. We modeled excess zeros in *F. harpi* data by including “sedge” as a predictor in the zero-inflation logit model (Table 2). The sedge variable indicated the number of quadrats in a transect that contained sedges. Sedge was modeled in this manner due to its significant relationship with the presence of both crayfish species across the landscape (see Rhoden, Taylor & Peterman, 2016), as well as our inability to accurately identify sites with sedges from spatial GIS data. The predictor variables for both analyses were based on averages of habitat data collected at each transect during the search of burrows in each quadrat (Table 2).

**Table 2:** Model variables. Variables and their descriptions for generalized linear model analysis of two primary burrowing crayfishes in Arkansas (*Fallicambarus harpi* and *Procambarus reimeri*). Quadrats were 1 m^2^ and transects were 50 m in length.

Variable	Description
Sedge	Presence of hydrophilic sedge in transect (binary: yes/no)
Herb	% herbaceous ground cover measured in each quadrat, averaged across each transect
Solar	Incoming solar radiation value (watt-hour/m^2^) averaged across each transect location based on direct and diffuse insolation from the unobstructed sky directions (ArcGIS, Environmental System Research Institute, Redlands, California)
Water_dist	Euclidean distance to nearest waterbody calculated at central point (25 m) of each transect location (National Hydrography Dataset; http://nhd.usgs.gov/index.html)
CTI	Average compound topographic index value calculated for each transect location (Evans et al., 2010)
Soil1, Soil2	Transformed soil composition (% sand, silt, clay) value calculated for each soil sample averaged across each transect (Van den Boogaart, Tolosana & Bren, 2014)
Mxnt	Average relative occurrence rate (ROR) calculated for each transect

We assessed model convergence and fit and then adjusted the optimization algorithm as needed. The full candidate model set is shown in Table 3. We compared candidate models with Akaike Information Criterion corrected for small sample sizes (AICc; Akaike, 1974) with the package MuMIn (Barton, 2014) by means of model selection and averaging described by Burnham & Anderson (2002) and Luckacs, Burnham & Anderson (2009).

**Table 3:** Candidate models. Candidate models in the generalized linear model analysis for *Fallicambarus harpi* and *Procambarus reimeri* in Arkansas. The response variable used in each model was burrow abundance/presence in each 50 m transect. See Table 2 for variable names.

Model name	Variables
Mod 1(global)	herb + solar + water_dist + cti + soil1 + soil2 + mxnt
Mod 2	mxnt + soil2
Mod 3	soil2 + soil1
Mod 4	solar + water_dist
Mod 5	cti + mxnt
Mod 6	herb + water_dist + cti
Mod 7	mxnt
Mod 8	mxnt + soil1

Field sampling occurred in March and April of 2015, the period of peak activity for both *F. harpi* and *P. reimeri* (Robison & Crump, 2004; Robison, 2008). Field sampling was conducted under funding agency Scientific Collection Permit number 030620151. At each sampling point, one 50-m linear transect was searched for the presence of burrows in six 1-m^2^ quadrats placed at 10 m intervals along each transect. Within a sampling polygon, the area surrounding the transect was also thoroughly searched for burrows. If burrows were present along the transect, quadrat, or within the vicinity of the transect, animals were captured with hand excavation by using a hand shovel to slowly dig around the burrow entrance and inserting one’s arm into the burrow feeling for the crayfish. This method was chosen over other methods due to the success rate and limited amount of time spent at each burrow location (Ridge et al., 2008).

## Results

### Maxent analysis

**Figure 2:**
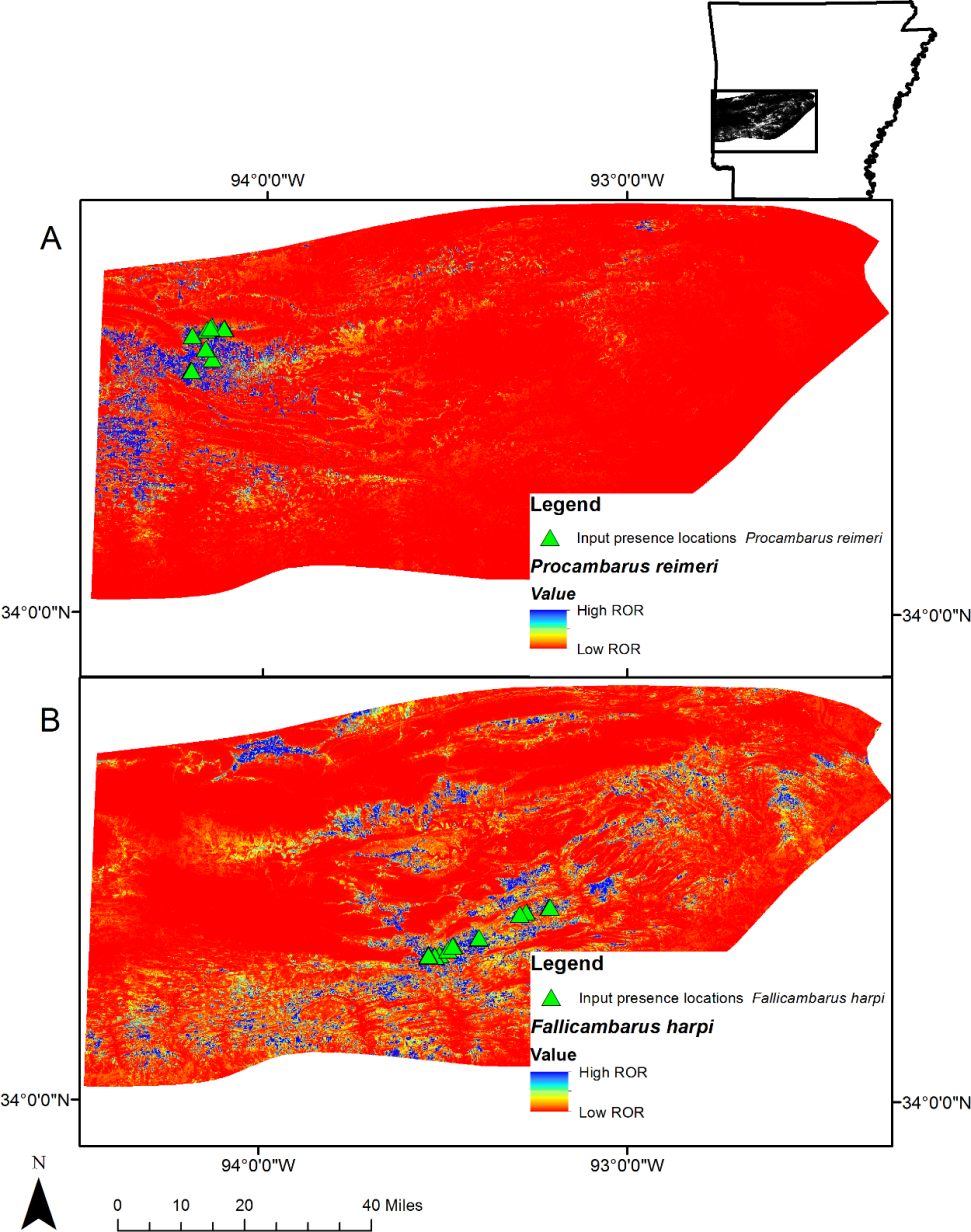
Projection of Maxent results. Projection of the Maxent models for (A) *Procambarus reimeri* and (B) *Fallicambarus harpi* onto the environmental variables (Table 1) used for analysis in western Arkansas. The total shaded area represents the Ouachita Mountains Ecoregion (OME). Cooler colors show areas with better predicted conditions (relative occurrence rates [ROR]).

The AUC converged to 0.959 and 0.976 for the final *F. harpi* and *P. reimeri* models, respectively. The model for *F. harpi* converged after 520 iterations and the model for *P. reimeri* converged after 420 iterations. Both models were significantly better than the random AUC estimations from the null models (*p* < 0.01). Of the parameters included in the model, canopy cover was the variable with the highest percent contribution for both species (48.8% and 47.2% *F. harpi* and *P. reimeri*, respectively; Table 4). Both species showed a steady decline in the probability of presence as canopy cover increased. The variable with the highest gain when used in isolation was elevation for both species (Table 4). An elevation between 150 m and 200 m was most suitable for *F. harpi* and between 300 m and 350 m was most suitable for *P. reimeri*. The concentration of the highest ROR was centered around the presence locations for both species (Fig. 2). The LPT was 0.07 for *F. harpi* and 0.26 for *P. reimeri*. In the *F. harpi* model, 10% of the area in the OME was predicted to be above the LPT. In the *P. reimeri* model, 2% of the OME was predicted to be above the LPT (Table 5).

**Table 4:** Maxent results. Percent contribution and permutation importance of each environmental variable analyzed in the final Maxent models for two primary burrowing crayfish species (*Fallicambarus harpi* and *Procambarus reimeri*) in western Arkansas.

Variable	Percent contribution	Permutation importance
*Fallicambarus harpi*		
Canopy	48.8	15.7
Elevation	37.9	56.3
CTI	7.6	2
Solar	4	25.4
Distance to nearest waterbody	1.8	0.6
*Procambarus reimeri*		
Canopy	47.2	27.5
Elevation	39.8	41.9
Distance to nearest waterbody	7.1	16.8
CTI	5.5	9
Solar	0.4	4.9

**Table 5:** Ground validation statistics. (A) Threshold values (relative occurrence rate; ROR), land area (ha), and percentage of Ouachita Mountains Ecoregion (OME) of each relative occurrence category; and (B) number of presence and absence quadrats, average canopy cover (%) of quadrats sampled in each relative occurrence category, and percentage of quadrats in each relative occurrence category with sedges present from the field sampling based on Maxent models for two primary burrowing crayfish species (*Fallicambarus harpi* and *Procambarus reimeri*) in western Arkansas.

**A**				
Species	Category 1	Category 2	Category 3	Category 4
	Thresholds (ROR)			
*Fallicambarus harpi*	0.00–0.07	0.07–0.44	0.44–0.66	0.66–0.88
*Procambarus reimeri*	0.00–0.26	0.26–0.42	0.42–0.64	0.64–0.85
	Land area (ha)			
*Fallicambarus harpi*	1374105	143139	21894	4996
*Procambarus reimeri*	1515441	15209	9756	3728
	Percentage of OME			
*Fallicambarus harpi*	89	9	1	<1
*Procambarus reimeri*	98	1	1	<1

**B**				
Variable	Category 1	Category 2	Category 3	Category 4
*Fallicambarus harpi*				
Present	0	0	5	14
Absent	121	120	115	105
Average Canopy Cover	34	26	16	7
Percent Quad w/ Sedge	27	73	44	46
*Procambarus reimeri*				
Present	0	12	14	15
Absent	122	106	106	105
Average Canopy Cover	38	18	19	15
Percent Quad w/ Sedge	51	55	63	45

### Field sampling and validation

**Figure 3:**
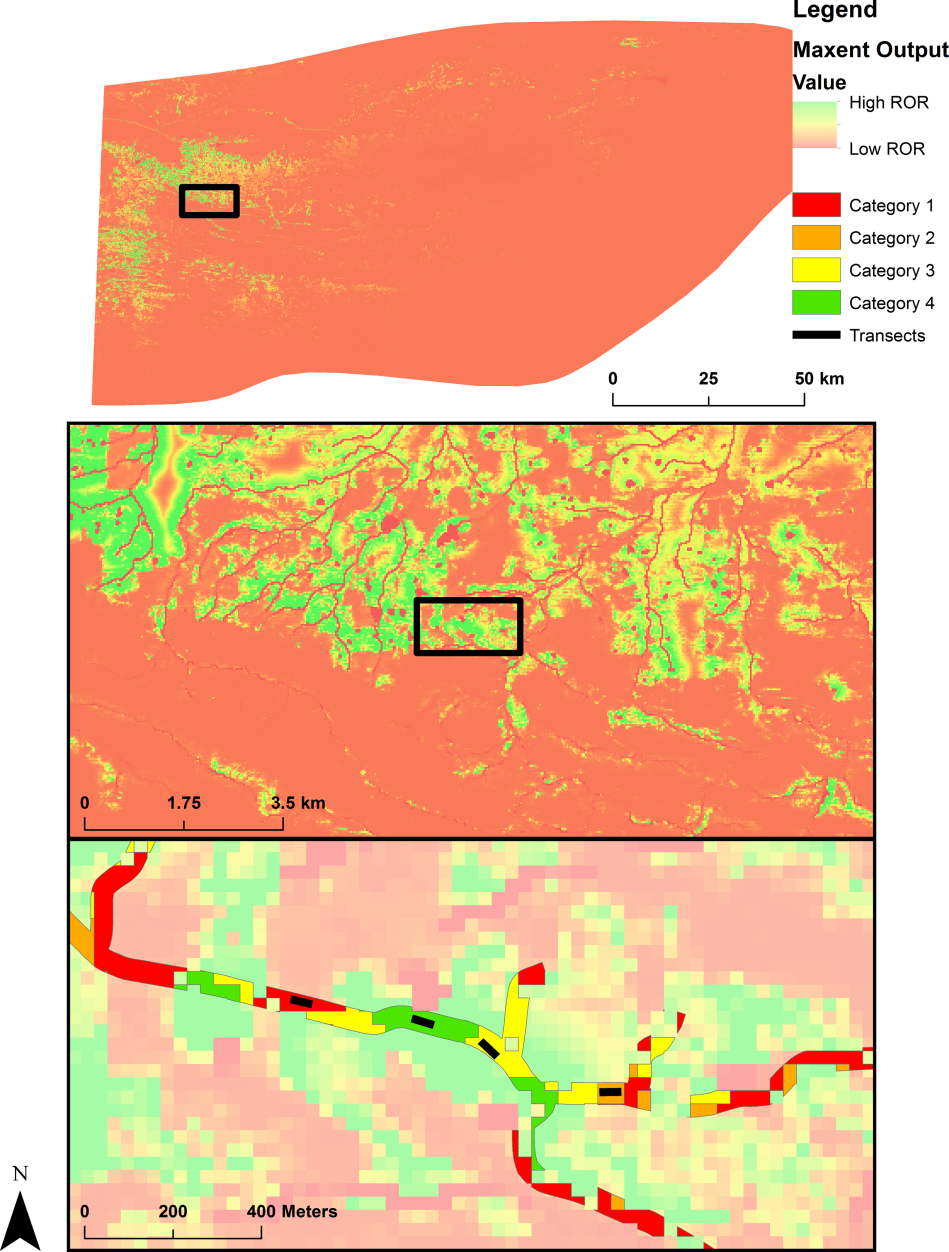
Map of ground validation. Map representing the sampling scheme based on the predictions from a Maxent analysis of two primary burrowing crayfish species (*Fallicambarus harpi* and *Procambarus reimeri*) in western Arkansas in the spring of 2015. Each color represents a relative occurrence category upon which the field validation sampling procedure was based. The black lines in the lower graphic depict 50-m transects used to assess presence or absence of the target species at each site. The linear, focused colors in the bottom graphic represent the accessible polygons in which the transect sampling was carried out.

All sites were sampled in the right of way of primary, secondary, and tertiary roadways (Fig. 3). Most (89% for *F. harpi* and 98% for *P. reimeri*) of the land area in the OME was in the first (lowest ROR) category (Table 5). No individuals of either species were caught in areas predicted below the LPT (category 1). Most (74%) of the presence locations for *F.* *harpi* were in category 4 (Table 5). The presence locations for *P. reimeri* were more evenly distributed between categories 2, 3, and 4 (Table 5). *Fallicambarus harpi* was captured in 19 of 480 quadrats within 5 of the 80 transects surveyed for the species. *Procambarus reimeri* was captured in 41 of 480 quadrats within 15 of the 80 transects surveyed for the species. We counted 70 burrows each for *F. harpi* and *P. reimeri*. The updated range of *F. harpi* extends 2.8 km to the north and 2 km to the south of its historical range while the updated range of *P. reimeri* extends 51.6 km to the east, 12.1 km to the south, and 19.2 km to the west of its historical range. Thus, the total range for both species was approximately 265 km^2^ for *F. harpi* and 1467 km^2^ for *P. reimeri* using a minimum convex polygon approach in ArcGIS encompassing all known capture localities from both years and historic museum data.

The AUC for the *F. harpi* field validation was 78.67 (63.29–94.04). The AUC for the *P.* *reimeri* field validation was 69.54 (56.16–82.92). The threshold values (prediction with the highest specificity and sensitivity) were 0.48 and 0.29 for *F. harpi* and *P. reimeri*, respectively. In both the *F. harpi* and *P. reimeri* models, the variable of ROR (Mxnt; Table 3) was in the top (AICc ≤4) models and was shown to have a positive relationship with the abundance of crayfish burrows in each transect (Table 6). Sedge was an important predictor of excess zeros in our *F. harpi* data (Table 7). As the number quadrats in a transect containing sedges increased, the likelihood of an excess zero decreased.

**Table 6:** Candidate generalized linear model results. Model name, number of model parameters (K), Akaike’s Information Criterion adjusted for small sample size (AICc), difference in AICc (ΔAICc), Akaike cumulative weights (*w*_*c*_), and log liklihood (LL) for models from a suite of variables modeled with a generalized linear model analysis for 2 primary burrowing crayfish species, *Fallicambarus harpi* (*n* = 80 transects) and *Procambarus reimeri* (*n* = 80 transects) in Arkansas. See Tables 2 and 3 for a description of each model and the variables included.

Model	K	AICc	ΔAICc	*w*_*c*_	LL
*Fallicambarus harpi*					
Mod 5^a^	6	65.5	0	0.69	−26.19
Mod 1^a^	11	68.9	3.35	0.82	−21.50
Mod 6	7	69.3	3.78	0.92	−26.88
Mod 8^a^	6	71.1	5.6	0.96	−29
Mod 7^a^	5	71.9	6.4	0.99	−30.6
Mod 2^a^	6	74.3	8.8	1	−30.6
Mod 3	6	77.1	11.5	1	−32.0
Mod 4	6	80.8	15.3	1	−33.8
*Procambarus reimeri*					
Mod 7^a^	3	137.7	0	0.40	−65.68
Mod 4	4	139.3	1.67	0.57	−65.41
Mod 2^a^	4	139.8	2.12	0.71	−65.63
Mod 5^a^	4	139.8	2.17	0.85	−65.66
Mod 8^a^	4	139.9	2.22	0.98	−65.68
Mod 3	4	144.2	6.56	1	−67.85
Mod 6	5	145.5	7.84	1	−67.35
Mod 1^a^	9	150.5	12.86	1	−64.99

**Notes.**

a Represent inclusion of ROR parameter (mxnt).

**Table 7:** Parameter estimates of generalized linear model analysis. Conditional model-averaged parameter estimates of the full candidate models (Table 6) for two primary burrowing crayfishes species (*Fallicambarus harpi* and *Procambarus reimeri*) in Arkansas. See Table 2 for a description of the variables included.

Species and variable	Model-averaged estimate (SE)	*p* > |*z*|
*Fallicambarus harpi*		
Herb	−0.75 (1.55)	0.63
Solar	1.45 (5.14)	0.78
Water_dist	−1.93 (1.75)	0.23
CTI	−0.98 (0.38)	0.01
Soil1	−5.77 (5.43)	0.29
Soil2	2.08 (1.68)	0.21
mxnt	3.96 (1.70)	0.02
Sedge (zero-infl)	−0.50 (0.30)	0.10
Count Intercept	−1.16 (2.38)	0.63
Zero-infl Intercept	3.51 (1.28)	0.01
*Procambarus reimeri*		
Solar	1.70 (0.69)	0.02
Water_dist	0.11 (0.47)	0.80
Cti	0.11 (0.50)	0.82
Soil1	−0.07 (0.55)	0.90
Soil2	−0.16 (0.56)	0.78
mxnt	1.25 (0.49)	0.01
Intercept	−0.61 (0.47)	0.20

## Discussion

We demonstrate that Maxent is a useful tool to predict new occurrences and the distribution of suitable habitats for two narrowly endemic, rare species with unique natural histories that span both terrestrial and aquatic life styles. Our models were successful in directing us to new populations of both species. We used a suite of functions to assess model fit and safeguard against potential pitfalls associated with the Maxent program (Phillips & Dudík, 2008; Warren & Seifert, 2011; Elith et al., 2011; Hijmans, 2012; Lahoz-Monfort, Guillera-Arroita & Wintle, 2014). We also used biologically relevant habitat information at a constrained geographic scale to increase the accuracy of our predictions (Guisan & Thuiller, 2005). These habitat variables and the scale at which we delineated them were a result of previous field sampling and analysis of habitat preference of both species (Rhoden, Taylor & Peterman, 2016), which revealed both crayfish to be microhabitat specialists; using open, low-herbaceous microhabitats. We validated the models through a stratified sampling of our Maxent model predictions based on the LPT and the maximum ROR. We then equally sampled each category across the entire OME. Both models performed well in the ROC analysis and subsequent generalized linear models. The ROR was shown to be positively associated with the number of burrows in a transect (represented as “mxnt” in Table 7) during the generalized linear models for each species. This analysis revealed that regions with higher estimated ROR not only are more likely to be occupied, but will harbor more individuals. This validation resulted in an expansion of both species’ known ranges and the discovery of new populations. The models performed well by directing sampling efforts to treeless areas on the landscape that tended to have greater predicted probabilities of occurrence. However, the models did a poor job of identifying the wet, low-herbaceous microhabitats most frequently associated with occurrence in the field and previous studies (Robison & Crump, 2004; Robison, 2008; Rhoden, Taylor & Peterman, 2016).

The habitat attributes of sites in which animals were present consisted of treeless, wet, low-herbaceous microhabitats. The average canopy cover for the categories above the LPT (category 2, 3, and 4) was 17% for both species. Quadrats where we detected our focal crayfish species had an average canopy cover of 5%. Hydrophilic sedges were present in over 90% of the quadrats having *F. harpi* and *P. reimeri* but were present in less than half of the quadrats predicted above the LPT (categories 2, 3, and 4). The sites recorded as being above the LPT (categories 2, 3, and 4) not having the target species were treeless for the most part, but those sites did not exhibit a moist microhabitat. The Maxent models thus did not capture the perched water table observed across the landscape associated with other primary burrowing crayfishes (Welch, Eversole & Riley, 2007). It is likely the model did not capture these moist, low herbaceous habitats due to the spatial resolution and variables chosen for the Maxent analysis (canopy cover, CTI, elevation, solar radiation, and distance to nearest waterbody). Future studies could incorporate remotely sensed data to better identify these unique habitats.

The use of the LPT to determine the threshold between the probability of presence or absence at any given predicted output location (Pearson et al., 2007) is well documented (Rinnhofer et al., 2012; Boria et al., 2014; Fois et al., 2015). We successfully used this value in our field validation techniques: no animal was captured in an area predicted below the LPT (Table 3). The land area above the LPT for the *F. harpi* model comprised 10% of the OME and 2% for the *P. reimeri* model in Arkansas. The ROC analysis identified threshold values of 0.48 and 0.29 for the *F. harpi* and *P. reimeri* models, respectively, which optimized the sensitivity (100 for both *F. harpi* and *P. reimeri*) and specificity (58.7 and 38.5 for *F. harpi* and *P. reimeri*, respectively) of our model (Robin et al., 2011). These values are far more conservative than the LPT and are based on the field validation results from both species. Using these threshold metrics, the area predicted as suitable habitat for *F. harpi* and *P. reimeri* is less than 1% of the OME. We recommend the use of this threshold based on the ROC analysis for a more fine-tuned sampling effort for high-quality habitat for both species in the future.

Our SDMs used fine-scale (30 m) rasters of biological variables relevant to our two study species (canopy cover, CTI, solar radiation, elevation, and distance to waterbody). In the past, it has been common to use coarse (≥1 km) climatic data to construct models (e.g., Peterson, 2001; Welch, Eversole & Riley, 2007; Chunco et al., 2013). The use of coarse-scale habitat variables in Maxent has been addressed in previous studies (Araújo & Guisan, 2006; Jiménez-Alfaro, Draper & Nogués-Bravo, 2012). Others using fine-scale inputs have found new populations of other rare species such as the discovery of new breeding ponds for a salamander species in east central Illinois (*Ambystoma jeffersonianum;* Peterman, Crawford & Kuhns, 2013). Using fine-scale spatial surfaces of specific habitat variables for narrowly endemic habitat specialists was more appropriate than the more general approach of coarse-scale climatic data due to the resolution one gains with specific habitat information and fine-scale inputs. This fine-scale resolution was necessary to capture elements of the microhabitat the crayfishes prefer by differentiating between suitable and unsuitable habitat within anthropogenically altered habitat situated in natural landscapes (e.g., roadside ditches). However, we note that the specific surfaces or resolution in our study still failed to completely capture essential habitat features or indicators of preferred habitat, such as sedges.

Conservation efforts for rare species benefit by narrowing the knowledge gap in distribution information, adding localities for monitoring persistence in roadside ditches, and providing habitat preference information. Our study shows Maxent was an appropriate tool to analyze habitat suitability and discover populations of narrowly endemic, rare species. Our method of reinvestigating museum localities, verifying species persistence, and collecting habitat data from verified locations added precision to the presence locations we used for analysis. Our initial surveys also added valuable information regarding the habitat preferences of both *F. harpi* and *P. reimeri*, which in turn guided the selection of our habitat variables for both models. Our concentrated search efforts resulted in the discovery of five new populations of *F. harpi* and 16 new populations of *P. reimeri* and known range expansions of approximately 91 km^2^ and 1,404 km^2^, respectively.

## Conclusion

Recent studies have found ground validation of Maxent has been a suitable method to determine the accuracy of predictions (Stirling et al., 2016). Our study supports this conclusion and offers a unique method, incorporating historic museum localities to inform an SDM of pertinent habitat variables and validating the localities before conducting the SDM. We have also shown Maxent works well with narrowly endemic, rare habitat specialists and fine scale (30 m) raster inputs. Constructing models followed by ground validation has added valuable habitat information to two spatially restricted, understudied species and illustrates the potential effectiveness of such a strategy for other rare habitat specialists.

##  Supplemental Information

10.7717/peerj.3632/supp-1 Figure S1 AUC graph of the Maxent output for the crayfish species *Fallicambarus harpi*

10.7717/peerj.3632/supp-2 Figure S2 AUC graph of the Maxent output for the crayfish species *Procambarus reimeri*

10.7717/peerj.3632/supp-3 Data S1 Supplemental raw data for *Fallicambarus harpi*

10.7717/peerj.3632/supp-4 Data S2 Supplemental raw data for *Procambarus reimeri*

10.7717/peerj.3632/supp-5 Supplemental Information 1 Thesis document Thesis document in which raw data and protocols for data collection are stored.

## Additional Information and Declarations

### Field Study Permissions

The following information was supplied relating to field study approvals (i.e., approving body and any reference numbers): Field experiments were approved by the Arkansas Game and Fish Commission.

### Data Availability

The following information was supplied regarding data availability: The thesis document with associated raw data can be found at http://hdl.handle.net/2142/90597 and has also been supplied as a Supplemental File.
