# Supplementary figures and images for: Maxent-directed field surveys identify new populations of narrowly endemic habitat specialists
Source: PeerJ. 2017 Jul 31;5:e3632. doi: 10.7717/peerj.3632 (PMC5541929; doi:10.7717/peerj.3632)

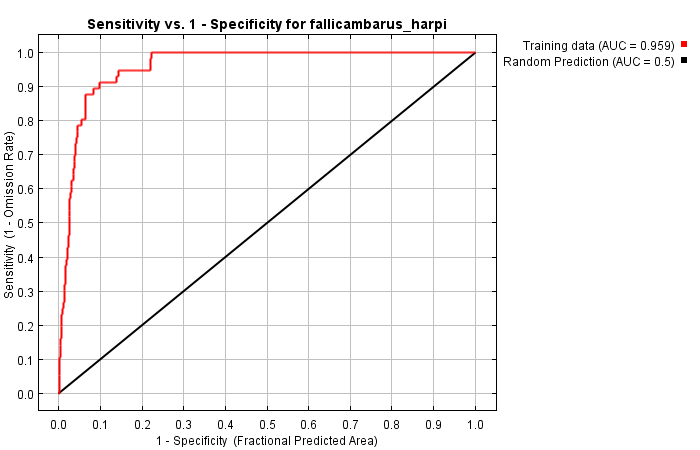

Supplement: Figure S1 [file peerj-05-3632-s001.png]

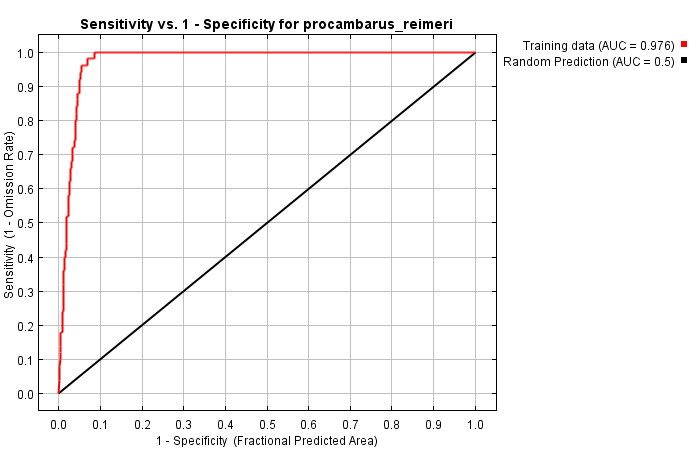

Supplement: Figure S2 [file peerj-05-3632-s002.png]
