# Supplementary material for: Maxent-directed field surveys identify new populations of narrowly endemic habitat specialists
Source: PeerJ. 2017 Jul 31;5:e3632. doi: 10.7717/peerj.3632 (PMC5541929; doi:10.7717/peerj.3632)
Supplement: Supplemental Information 1 — Thesis document in which raw data and protocols for data collection are stored. [file peerj-05-3632-s005.pdf]

THE USE OF RIGHT OF WAYS BY PRIMARY BURROWING CRAYFISHES IN THE OUACHITA MOUNTAINS ECOREGION OF ARKANSAS

BY

CODY MARTIN RHODEN

THESIS

Submitted in partial fulfillment of the requirements
for the degree of Master of Science in Natural Resources and Environmental Sciences
in the Graduate College of the
University of Illinois at Urbana-Champaign, 2016

Urbana, Illinois

Master's Committee:

Adjunct Assistant Professor Christopher A. Taylor, Adviser
Associate Professor Robert L. Schooley
Adjunct Assistant Professor Michael Dreslik
Assistant Professor William E. Peterman, The Ohio State University

ABSTRACT

Roadside ditches can harbor beneficial and detrimental attributes for animal persistence. I sought to determine if roadside ditches could be preferred by two narrowly endemic primary burrowing crayfish species, *Fallicambarus harpi* and *Procambarus reimeri*. To investigate this interaction, I collected habitat data, locality information, and tested computer generated habitat models for these two species in the Ouachita Mountains Ecoregion (OME) of western Arkansas in the spring of 2014 and 2015. My first objective was to determine the fine-scale habitat preferences of *F. harpi* and *P. reimeri* in relation to their occurrence in roadside ditches. My analysis revealed these species to be habitat specialists, preferring open habitat with a low-herbaceous, wet microhabitat; similar to habitat found in roadside ditches. My second objective was to determine the ability of habitat models to accurately predict the occurrence of these two crayfishes across the OME. To investigate this objective, I used the locality data gained in the first field season to construct species distribution models using the program Maxent. I then used the species distribution model as a guide to sample for both crayfish species across the OME. My analysis revealed that species distribution models, specifically Maxent, are a suitable tool for analyzing and discovering new populations of both *F. harpi* and *P. reimeri*. My concentrated search efforts resulted in a documented range expansion of both species in the OME. My third objective was to assess the conservation status of both *F. harpi* and *P. reimeri*. Using the locality data that I collected over the two years of study (2014 and 2015), I was able to determine *F. harpi* and *P. reimeri* are constrained geographically but relatively stable throughout their range. I discovered new populations of both species, moderately expanding the range of *F. harpi* (<100 km^2) and *P. reimeri* by a larger distance (>1000 km^2). I conclude that the microhabitat of roadside ditches can be beneficial to the persistence of these two narrowly endemic habitat specialists in the Ouachita Mountains Ecoregion in Arkansas.

To my grandparents, Bill and Gloria Emrich, and my parents, Steve and Kara Rhoden, who gave me the gift of higher education

ACKNOWLEDGEMENTS

I would like to thank my advisor, Dr. Christopher Taylor for all of his guidance and support throughout my graduate studies. The knowledge and patience that he has shown me have been invaluable to my education and development as a biologist. I would also like to thank Dr. Bill Peterman for sharing his extensive knowledge in modeling and statistical analysis, without which my results would be much less informative. Also thanks to Drs. Mike Dreslik and Bob Schooley for assistance with the initial study design and statistical analysis. I am grateful for those who helped me in the field and with permitting, namely Brian Wagner, Josh Seagraves, Allison Fowler, Andrea Daniel, Benjamin Thesing, and Justin Stroman. Special thanks to Sarah Tomke and Dan Wylie for their relenting assistance in digging mudbugs in the ditches of Arkansas with me. Finally, I would like to thank my friends and family for their unwavering support and encouragement during my graduate research.

TABLE OF CONTENTS

CHAPTER 1: GENERAL INTRODUCTION.. 1

CHAPTER 2: ROADSIDES AS PREFERRED HABITAT FOR TWO NARROWLY ENDEMIC CRAYFISHES 12

CHAPTER 3: FIELD VALIDATION OF MAXENT MODELING OF TWO NARROWLY ENDEMIC CRAYFISHES 40

CHAPTER 4: HABITAT ASSESSMENT AND RANGE UPDATES FOR TWO RARE ARKANSAS BURROWING CRAYFISHES: *FALLICAMBARUS HARPI* AND *PROCAMBARUS REIMERI* 69

SUMMARY 85

APPENDIX A: CRAYFISH SAMPLING SUPPLEMENTARY MATERIAL 87

APPENDIX B: CRAYFISH CAPTURE SUPPLEMENTARY MATERIAL....................... 89

APPENDIX C: *FALLICAMBARUS HARPI* SAMPLING SUPPLEMENTARY MATERIAL.. 94

APPENDIX D: *PROCAMBARUS REIMERI* SAMPLING SUPPLEMENTARY MATERIAL.. 120

CHAPTER 1: GENERAL INTRODUCTION

The conservation of rare species is critical for the continuation of ecosystem function. Species considered rare can have a larger role in ecosystems than other more common species (e.g. keystone species; Power et al. 1996). Rare species contribute to ecosystem function by influencing pathways of energy and material flows (Hooper et al. 2005), resisting invasion (Lyons and Schwartz 2001), and delivering more unusual and important functions than their abundance would suggest (Mouillot et al. 2013). Conservation of rare species also adds to the biodiversity of an ecosystem, which in turn maintains the health of the ecosystem (Lyons et al. 2005). Unfortunately, the very nature of rare species (i.e., low abundance, narrow endemism) makes them difficult to study comprehensively. One such taxon in which rare species remain understudied are North American crayfishes (Taylor et al. 2007).

Approximately two-thirds of the 500+ species of crayfish in the world occur in North America (Taylor et al. 2007). Within North America, the majority (99%) of species are assigned to the family Cambaridae (Taylor et al. 2007), which reaches its highest diversity in the southeastern United States. Increases in species diversity lead to an increase in ecosystem function across many taxa (see review by Hooper et al. 2005), and in particular, the diversity of crayfishes observed in North America results in species' playing multiple roles in ecosystems.

Throughout their range, crayfishes are vital to ecosystem function (Momot 1995). Crayfishes act as ecosystem engineers by consuming detritus (Usio and Townsend, 2002), creating terrestrial habitat (Carpenter, 1953; Horwitz and Knott, 1991; Kingsbury and Coppola, 2000; Seigel, 1986; Welch et al., 2008), modifying sediment layers (Harvey et al., 2011; Statzner et al., 2000), and breaking down leaf litter in streams (Creed and Reed, 2004; Schofield et al., 2001). Crayfishes serve as an important prey item for sport fish (Clady, 1974; Lewis et al.,

1961), rare herpetofauna (Penn, 1950; Peterson at al., 1989; Smith et al., 1948), and many other aquatic and terrestrial animal species. Crayfishes can also act as predators (Parkyn et al., 2001; Taylor and Soucek, 2010) by preying upon aquatic and terrestrial invertebrates, fish, and other crayfishes.

However, as the importance and number of roles crayfishes inhabit become clearer, so do the nature and breadth of the threats they face. Approximately one-third of the world's crayfish species are threatened with extinction (Richman et al. 2015). In the United States and Canada, 48% of all crayfishes are considered imperiled (Taylor et al. 2007). In fact, crayfishes in the United States trail only freshwater mussels and snails in their level of imperilment (Wilcove and Master 2005). The specific threats facing native species of crayfishes include modification of species' habitats or ranges, over-utilization, disease, and limited distributions (Taylor et al. 2007). Animals that are rare or possess a restricted range are more sensitive to these cumulative stresses than more common, widespread fauna. Intensifying these threats is the lack of natural history data for many crayfish species, particularly North American primary burrowing crayfishes (Moore et al. 2013).

It is hypothesized all crayfishes have the ability to construct refugia and access ground or atmospheric water for oxygen extraction by burrowing into the soil or substrate (Berrill and Chenoworth 1982; Hobbs 1981). Based on differences in natural history, Hobbs (1981) described three classes of burrowing crayfishes: tertiary, secondary, and primary burrowers. Tertiary burrowers dig shallow burrows only to escape frost, lay and brood eggs, or avoid desiccation. Secondary burrowers spend much of their lives in their burrows, which normally have a connection to an open, permanent water body; however they do move out into open water occasionally. In contrast, primary burrowing crayfishes spend their complete life cycle

underground. As primary burrowers only leave their burrows to forage and locate a burrow of the opposite sex for mating (Hobbs 1981), their burrows are rarely tied to permanent open water. Rather, primary burrowers use subsurface groundwater for moisture and oxygenation. Primary burrowing crayfishes reach a high level of diversity in the eastern United States.

The Ouachita Highlands Freshwater Ecoregion, which covers southeastern Oklahoma, northeastern Texas, southern Arkansas, and northwestern Louisiana, has the sixth highest diversity of native crayfishes in the United States and Canada (Moore et al. 2013). Arkansas has 13 endemic crayfish species (Robison et al. 2008), of which eight are primary burrowing crayfishes. Within the Ouachita Highlands Freshwater Ecoregion, the Ouachita Mountains Ecoregion (OME) of southwestern Arkansas (Woods et al. 2004) has six species (*Fallicambarus harpi, F. jeanae, F. strawni, Procambarus liberorum, P. parasimulans*, and *P. reimeri*), which represents the highest diversity of primary burrowing crayfishes in the state. Because these crayfish occur at such a constrained geographic scale, it is important to accurately describe their habitat preferences to ensure management of habitats appropriate for those species.

Two narrowly endemic, primary burrowing crayfishes found in the OME are *Fallicambarus harpi* and *Procambarus reimeri*. *Fallicambarus harpi* was discovered in 1985 by H.H. Hobbs, Jr. and H.W. Robison and was known from Montgomery, Hot Spring, Garland, and Pike counties in Arkansas before 2015. *Fallicambarus harpi* is categorized as a primary burrower and is known from wet seepage areas with abundant sedges such as roadside ditches and other right of ways (Robison and Crump 2004). However, since 2004, no new information has been published relating to the range or habitat requirements of *F. harpi*. *Procambarus reimeri* was first discovered in 1979 by H.H. Hobbs, Jr. and was known from only one county (Polk) in western Arkansas before 2015. This primary burrowing crayfish reportedly constructs

simple burrows in sandy clay soil in wet seepage areas as well as roadside ditches (Hobbs 1979, Robison 2008), though no information has been published relating to the range or habitat requirements of *P. reimeri* since 2008. Much of the information that does exist fails to address the relationships between the crayfishes and their habitat. The relevant studies lean heavily on descriptions of the habitat during sampling periods and lack empirical evidence of habitat suitability. However, the current habitat preferences of these animals can be used to make inferences about their historic suitable habitat, undisturbed by anthropogenic change.

Human-made, linear right of ways (ROWs) are known to have burrowing crayfish burrows. The habitat in which these animals occurred naturally, before human development, could have been functionally similar to a human-made linear ROW. There are multiple records of burrowing crayfishes occurring in ROWs. These records are from species descriptions and natural history research (Doran and Richards, 1996; Hobbs and Whiteman, 1991; Norrocky, 1991; Page, 1985; Robison and Crump, 2004), and several museum databases (Illinois Natural History Survey Crustacean Database, Arkansas Department of Natural Heritage Database, and National Museum of Natural History Invertebrate Zoology Collection). This association is potentially due to the ease of sampling, as the ROW is highly accessible and provides a sampling area that results in high detectability from roadway maintenance, such as mowing and spraying woody vegetation. This roadway maintenance also contributes to a wet, low-herbaceous microhabitat. There currently is a lack of understanding as to whether the observed occurrence of these species in ROWs is an artifact of the accessibility of these habitats or whether the actual habitat created by ROW activities is preferred.

To assess the possibility *F. harpi* and *P. reimeri* prefer the microhabitat of the ROW and to fill in the knowledge gaps associated with their habitat preferences and population statuses, I

developed a study with the following objectives: 1) to test *a priori* hypotheses about the habitat use of two primary burrowing crayfish species (*F. harpi* and *P. reimeri*) in relation to their occurrence in roadways, 2) to investigate the use of statistical modeling to predict the occurrence of individuals across the landscape and use these predictions to refine future sampling, and 3) to delineate the geographic distribution of both species using field-validated distribution models.

The body of this thesis is divided into three separate chapters. Chapter 2 describes the habitat preferences of *F. harpi* and *P. reimeri* in relation to their occurrence in ROWs. The use of computer modeling to predict the occurrence of both crayfishes is described in Chapter 3. The final chapter consists of range delineations for both species and a conservation assessment.

LITERATURE CITED

Berrill, M., and B. Chenoworth. 1982. The Burrowing Ability of Nonburrowing Crayfish. American Midland Naturalist 108:199–201.

Carpenter, C. C. 1953. A Study of Hibernacula and Hibernating Associations of Snakes and Amphibians in Michigan. Ecology 34:74–80.

Clady, M. D. 1974. Food Habits of Yellow Perch, Smallmouth Bass and Largemouth Bass in Two Unproductive Lakes in Northern Michigan. American Midland Naturalist 91(2):453–459.

Creed Jr., R. P., and J. M. Reed. 2004. Ecosystem engineering by crayfish in a headwater stream community. *Journal of the North American Benthological Society* 23(2):224–236.

Doran, N., and K. Richards. 1996. Management requirements for rare and threatened burrowing crayfish in Tasmania. Tasmanian Public Land Use Commission. Tasmania RFA Environment and Heritage Technical Committee. Hobart, Tasmania.

Harvey, G. L., T. P. Moorhouse, N. J. Clifford, A. J. Henshaw, M. F. Johnson, D. W. Macdonald, I. Reid, and S. P. Rice. 2011. Evaluating the role of invasive aquatic species as drivers of fine sediment-related river management problems: The case of the signal crayfish (*Pacifastacus leniusculus*). Progress in Physical Geography 35(4):517–533.

Hobbs, H. H., Jr. and M. Whiteman. 1991. Notes on the Burrows, Behavior, and Color of the Crayfish *Fallicambarus* (*F.*) *devastator* (Decapoda: Cambaridae). The Southwestern Naturalist 36:127–135.

Hobbs, H. H., Jr. 1979. A new crayfish from the Ouachita River basin in Arkansas (Decapoda: Cambaridae). Proceedings of the Biological Society of Washington 92(4):804–811.

Hobbs, H.H., Jr. and H.W. Robison. 1985. A new burrowing crayfish (Decapoda: Cambaridae) from southwestern Arkansas. Proceedings of the Biological Society of Washington 98(4):1035-1041.

Hobbs, H. H., Jr. 1981. The Crayfishes of Georgia. Smithsonian Contributions to Zoology. Smithsonian Contributions to Zoology, Washington D.C.

Hooper, D.U., F.S. Chapin III, J.J. Ewel, A. Hector, P. Inchausti, S. Lavorel, J.H. Lawton, D.M. Lodge, M. Loreau, S. Naeem, B. Schmid, H. Setālä, A.J. Symstad, J. Vandermeer, and D.A. Wardle. 2005. Effects of biodiversity on ecosystem functioning: a consensus of current knowledge. Ecological Monographs 75(1):3-35.

Horwitz, P., and B. Knott. 1991. The faunal assemblage in freshwater crayfish burrows in sedgeland and forest at Lightning Plains, Western Tasmania. Papers and Proceedings of the Royal Society of Tasmania 125:29–32.

Kingsbury, B. A., and C. J. Coppola. 2000. Hibernacula of the Copperbelly Water Snake (*Nerodia erythrogaster neglecta*) in Southern Indiana and Kentucky. *Journal of Herpetology* 34(2):294–298.

Lewis, W. M., G. E. Gunning, E. Lyles, and W. L. Bridges. 1961. Food Choice of Largemouth Bass as a Function of Availability and Vulnerability of Food Items. Transactions of the American Fisheries Society 90(3):277–280.

Lyons, K.G., M.W. Schwartz. 2001. Rare species loss alters ecosystem function – invasion resistance. Ecology Letters 4:358-365.

Lyons, K. G., C. A. Brigham, B. H. Traut, and M. W. Schwartz. 2005. Rare Species and Ecosystem Functioning. Conservation Biology 19(4):1019–1024.

Momot, W. T. 1995. Redefining the role of crayfish in aquatic ecosystems. Reviews in Fisheries Science 3:33-63.

Moore, M. J., R. J. DiStefano, and E. R. Larson. 2013. An assessment of life-history studies for USA and Canadian crayfishes: identifying biases and knowledge gaps to improve conservation and management. Freshwater Science 32(4):1276–1287.

Mouillot, D., D. R. Bellwood, C. Baraloto, J. Chave, R. Galzin, M. Harmelin-Vivien, M. Kulbicki, S. Lavergne, S. Lavorel, N. Mouquet, C. E. T. Paine, J. Renaud, and W. Thuiller. 2013. Rare Species Support Vulnerable Functions in High-Diversity Ecosystems. PLoS Biology 11(5):e1001569.

Norrocky, M. J. 1991. Observations on the Ecology, Reproduction, and Growth of the Burrowing Crayfish *Fallicambarus* (*Creaserinus*) *fodiens* (Decapoda: Cambaridae) in North-central Ohio. American Midland Naturalist 125:75–86.

Page, L. 1985. The crayfishes and shrimps (Decapoda) of Illinois. Illinois Natural History Survey Bulletin. 33(4):i-339.

Parkyn, S., K. Collier, and B. Hicks. 2001. New Zealand stream crayfish: functional omnivores but trophic predators? Freshwater Biology 46:641–652.

Penn, G. H. 1950. Utilization of crawfishes by cold-blooded vertebrates in the Eastern United States. American Midland Naturalist 44(3):643–658.

Peterson, C. L., J. W. Reed, and R. F. Wilkinson. 1989. Seasonal Food Habits of *Cryptobranchus alleganiensis* (Caudata: Cryptobranchidae). The Southwestern Naturalist 34:438–441.

Power, M. E., D. Tilman, J. A. Estes, B. A. Menge, W. J. Bond, L. S. Mills, G. Daily, J. C. Castilla, J. Lubchenco, and R. T. Paine. 1996. Challenges in the Quest for Keystones. BioScience 46(8):609–620.

Richman, N. I., M. Böhm, S. B. Adams, F. Alvarez, E. A. Bergey, J. J. S. Bunn, Q. Burnham, J. Cordeiro, J. Coughran, K. A. Crandall, K. L. Dawkins, R. J. DiStefano, N. E. Doran, L. Edsman, A. G. Eversole, L. Füreder, J. M. Furse, F. Gherardi, P. Hamr, D. M. Holdich, P. Horwitz, K. Johnston, C. M. Jones, J. P. G. Jones, R. L. Jones, T. G. Jones, T. Kawai, S. Lawler, M. López-Mejía, R. M. Miller, C. Pedraza-Lara, J. D. Reynolds, A. M. M. Richardson, M. B. Schultz, G. A. Schuster, P. J. Sibley, C. Souty-Grosset, C. A. Taylor, R. F. Thoma, J. Walls, T. S. Walsh, and B. Collen. 2015. Multiple drivers of decline in the global status of freshwater crayfish (Decapoda: Astacidea). Philosophical transactions of the Royal Society of London. Series B, Biological sciences 370:20140060.

Robison, H. W., and B. Crump. 2004. Distribution, natural history aspects, and status of the Arkansas endemic crayfish, *Fallicambarus harpi* Hobbs and Robison, 1985. *Journal of the Arkansas Academy of Science* 58:91–94.

Robison, H. W., C. McAllister, C. Carlton, and G. Tucker. 2008. The Arkansas endemic biota: An update with additions and deletions. *Journal of the Arkansas Academy of Science* 62:84-96.

Robison, H. W. 2008. Distribution, life history aspects, and conservation status of three Ouachita Mountain crayfishes: *Procambarus tenuis, P. reimeri,* and *Orconectes menae*. U.S.D.A. Forest Service, Ouachita National Forest. Hot Springs, Arkansas.

Schofield, K. A., C. M. Pringle, J. L. Meyer, and A. B. Sutherland. 2001. The importance of crayfish in the breakdown of rhododendron leaf litter. Freshwater Biology 46:1191–1204.

Seigel, R. A. 1986. Ecology and conservation of an endangered rattlesnake, *Sistrurus catenatus*, in Missouri, USA. Biological Conservation 35:333–346.

Smith, H. M., C. W. Nixon, and P. E. Smith. 1948. A Partial Description of the Tadpole of *Rana areolata circulosa* and Notes on the Natural History of the Race. American Midland Naturalist 39(3):608–614.

Statzner, B., E. Fievet, J. Champagne, R. Morel, and E. Herouin. 2000. Crayfish as geomorphic agents and ecosystem engineers: biological behavior affects sand and gravel erosion in experimental streams. Limnology and Oceanography 45(5):1030–1040.

Taylor, C. A., G. A. Schuster, J. E. Cooper, R. J. DiStefano, A. G. Eversole, P. Hamr, H. H. Hobbs III, H. W. Robison, C. E. Skelton, and R. F. Thoma. 2007. A reassessment of the conservation status of crayfishes of the United States and Canada after 10+ years of increased awareness. Fisheries 32(8):372–389.

Taylor, C., and D. Soucek. 2010. Re-examining the importance of fish in the diets of stream-dwelling crayfishes: implications for food web analyses and conservation. The American Midland Naturalist 163:280–293.

Usio, N., and C. R. Townsend. 2002. Functional significance of crayfish in stream food webs: roles of omnivory, substrate heterogeneity, and sex. Oikos 98:512–522.

Welch, S. M., J. L. Waldron, A. G. Eversole, and J. C. Simoes. 2008. Seasonal Variation and Ecological Effects of Camp Shelby Burrowing Crayfish (*Fallicambarus gordoni*) Burrows. The American Midland Naturalist 159(2):378–384.

Wilcove, D. S., and L. L. Master. 2005. How Many Endangered Species are there in the United States? Frontiers in Ecology and the Environment 3:414–420.

Woods, A. J., T. L. Foti, S. S. Chapman, J. M. Omernik, J. A. Wise, E. O. Murray, W. L. Prior, J. B. J. Pagan, J. A. Comstock, and M. Radford. 2004. Ecoregions of Arkansas (color poster

with map, descriptive text, summary tables, and photographs). US Geological Survey map scale 1:1,000,000. U.S. Geological Survey, Reston, Virginia.

CHAPTER 2: ROADSIDES AS PREFERRED HABITAT FOR TWO NARROWLY ENDEMIC CRAYFISHES[1]

INTRODUCTION

Human-made linear right-of-ways (ROWs) such as roads, roadside ditches, public utility easements, and railroad lines, and their maintenance are dramatically altered landscape features that traditionally have been observed to have a negative effect on habitat and life-history attributes of animal populations (Rytwinski and Fahrig 2013). For example, ROWs can disrupt wildlife movements (Richardson et al. 1997), fragment habitat (Andrews 1990), and directly cause mortality (Ashley and Robinson 1996, Lode 2000). Roads can act as a physical barrier to movement as well as a behavioral barrier (Oxley et al. 1974, Riley et al. 2006). In addition, the particulate matter emissions from vehicles can be a negative attribute of roadside microhabitat environments (Thorpe and Harrison 2008). Conversely, roadsides can positively contribute to plant and animal persistence by acting as vectors for native and nonnative species dispersal (Gelbard and Belnap 2003) and have led to higher animal densities and diversity than in surrounding habitat (Adams and Geis 1983). Such interactions observed in these areas are directly contributable to the construction and maintenance of the linear ROW.

The objectives of roadside maintenance activities have changed very little since their conversion from dirt trails to paved roads and include maintaining hydraulic capacity of ditches, eliminating vegetative obstructions, and providing wildlife habitat where compatible with roadway traffic (Berger 2005). The ROW environment is disturbed constantly by roadside maintenance (e.g. mowing, spraying herbicide, tree cutting) and remains open, resulting in habitats that resemble early successional stages in natural landscapes. Thus, roadside

[1] This chapter includes material in press at the time of writing this thesis. Citation: Rhoden, C.M., C.A. Taylor, W.E. Peterman. 2016. Highway to Heaven? Roadsides as preferred habitat for two narrowly endemic crayfish. Freshwater Science. 35(3):XXXX

maintenance can lead to open habitat within a matrix of forested habitat (Watkins et al. 2003), an alteration that can be both beneficial and detrimental to the persistence of wildlife.

The characteristics of animal populations that are vulnerable to negative road effects have been documented as those having high intrinsic mobility, high migration potential, multiple resource needs, low density/large area requirements, and a low reproductive rate; being a forest interior species; and displaying a behavioral avoidance of roads (Forman et al. 2003). Animals that display these traits are inhibited by the physical presence of the road and influences associated with the ROW, such as edge effects. Investigators have studied the responses of various biotic communities to ROWs (see Spellerberg 1998), but the interaction of biotic communities and ROWs is still not fully understood. Fahrig and Rytwinski (2009) reviewed biotic communities and roadsides and found that 59% of interactions resulted in a negative effect on animal abundance. The minority of animal populations that experience some positive effects from roadsides have a small territory range, have a high reproductive rate, and are small bodied. Investigators have shown a positive response from fauna that exhibit these life-history characteristics (Peris and Pescador 2004, Rosa and Bissonette 2007, Ward et al. 2008). Small populations of endemic habitat specialists often experience negative effects from ROW construction and maintenance (e.g. Altrichter and Boaglio 2004, Pocock and Lawrence 2005, Semlitsch et al. 2007). However, construction and maintenance of ROWs could benefit directly some narrowly endemic habitat specialist taxa by creating suitable habitat (Forman et al. 2003). One such taxon experiencing these benefits could be North American primary burrowing crayfishes.

All crayfishes are hypothesized to have the ability to construct refugia by way of burrowing into the soil or substrate (Hobbs 1981, Berrill and Chenoworth 1982). Construction of

burrows and the open space within them allow access to ground or atmospheric water for O_2 extraction. Hobbs (1981) described three classes of burrowing crayfishes based on differences in natural history: tertiary, secondary, and primary burrowers. Tertiary burrowers dig shallow burrows only to escape frost or seek shelter when the body of water they are inhabiting dries up. Secondary burrowers spend much of their lives in their burrows; however, they do move out into open water occasionally, and their burrows normally have a connection to an open, permanent water body. Primary burrowing crayfishes spend their complete life cycle underground. As primary burrowers leave their burrows only to forage and locate a burrow of the opposite sex for mating (Hobbs 1981), their burrows are rarely tied to permanent open water. Rather, these species use subsurface groundwater for moisture and oxygenation.

Prior to the 20th century, the habitat in which some primary burrowing crayfishes occurred naturally could have been functionally similar to some human-made ROWs. Specifically, human-made ROWs, such as roadsides, could imitate the hypothesized natural habitat of these animals by creating a landscape that is void of trees, supports a perched water table, and maintains an open, low-herbaceous microhabitat. To study the relationship between crayfishes and ROWs, we examined two narrowly endemic habitat specialists, *Fallicambarus harpi* and *Procambarus reimeri*, known from the Ouachita Mountains Ecoregion (OME) of western Arkansas (Woods et al. 2004). These species are vulnerable to population declines and are currently listed as endangered (*P. reimeri*) and vulnerable (*F. harpi*) according to Taylor et al. 2007. The conservation categories of endangered and vulnerable are based upon the American Fisheries Society Endangered Species Committee, which follows Williams et al. 1993. In addition, these species were included in a recent petition filed by the privately funded Center for Biological Diversity for protection under the US Endangered Species Act.

Both species are known historically from <40 individual sampling sites in restricted areas of the OME. Robison and Crump (2004) reported *F. harpi* as occurring in wet grassy areas that often had abundant sedges and grasslands such as ditches and pastures. Robison (2008) reported the habitat in which *P. reimeri* was observed as wet seepage areas and roadside ditches. Based on historic accounts of both species, we predicted some habitat attributes would be more important than others, particularly the presence of sedges and open canopy. We also expected soil composition would be a strong driver of burrow placement. Soil cues are important for other burrowing crayfishes (e.g. Grow and Merchant 1980, Barbaresi et al. 2004, Helms et al. 2013). To evaluate whether *F. harpi* and *P. reimeri* could be experiencing a positive effect from the microhabitat in ROWs, we developed a study based on extensive field sampling and habitat modeling of multiple variables to determine the fine-scale habitat preferences of both *F. harpi* and *P. reimeri* in relation to ROWs.

METHODS

Study site

Our study sites were situated in the Ouachita and Caddo River drainages of southwestern Arkansas. We focused on five counties in the OME that encompassed the entire known range of *F. harpi* and *P. reimeri* (**Fig. 2.1**). The OME has the highest diversity of primary burrowing crayfishes in the state. Six species occur there: *Fallicambarus harpi, F. jeanae, F. strawni, Procambarus liberorum, P. parasimulans,* and *P. reimeri*.

The Ouachita Mountains are composed of parallel, folded, east–west ridges underlain by shale and sandstone (Miser 1929). The soils of this region are generally categorized as silty clay and silty loam (Hlass et al. 1998). The most common forest community is mixed pine–hardwood;

however, remnant pine-bluestem (*Pinus-Schizachyrium*) communities do exist (Phillips and Marion 2005). Logging and recreation make up the major land uses of this area, and pastureland and hay fields are found in the broader valleys (Woods et al. 2004). We focused our sampling effort in these broader valleys.

Field collections

All sampling took place in April 2014 during the peak activity period for both species (Robison and Crump 2004, Robison 2008) and thus would result in highest species detection. The databases of the Illinois Natural History Survey Crustacean Collection, National Museum of Natural History Invertebrate Zoology Collection, and Arkansas Department of Natural Heritage were used to identify known historical locations for both *F. harpi* and *P. reimeri*. For each species, we selected historic localities that were accessible and could be validated with geographic positioning information. At each sampling site we positioned three to six 50 m transects ≤100 m from the initial transect. The initial transects were parallel to the road and within the ROW. We placed the initial transect at each sampling site where burrows were present, ensuring the initial transect was situated at the historical museum location. All transects were delineated with a fiberglass measuring tape. We laid out each transect and then checked for the presence or absence of burrows and standing water. After we obtained a global positioning system location and azimuth at the 0-m mark, we placed a 1-m^2 polyvinyl chloride quadrat over the tape every 10 m, which resulted in six 1-m^2 quadrats/50-m transect. After we completed the initial transect, we completed the remaining two to five transects in adjacent habitat in the same manner. We decided the number of transects to be sampled at each site based on habitat heterogeneity. If a site was homogenous, we sampled fewer transects to increase the number of sampling sites that could be visited during our sampling window. We defined adjacent habitat as

having significantly more or less canopy cover, seemingly different soil moisture content, higher or lower elevation, or a different dominant vegetation type compared with the initial transect. We excavated burrows at each sampling site and along each transect to ensure any burrows counted at a sampling site harbored the target species. We collected voucher specimens of each target species from all sites with burrows present and deposited them in the Illinois Natural History Survey Crustacean Collection.

Habitat variables

We collected the following habitat variables within each 1-m^2 quadrat: percent tree canopy cover, percent herbaceous ground cover, stem density, number of burrows, and the presence or absence of hydrophilic sedges (**Appendix A**; **Table A.1**). We estimated percent tree canopy cover with a concave spherical densiometer (Spherical Densiometer, Model-C; Robert E. Lemmon, Forest Densiometers, Bartlesville, Oklahoma). We calculated percent herbaceous ground cover by inverting the concave spherical densiometer over the 1-m^2 quadrat. We calculated stem density by counting the stems within a smaller (100-cm^2) quadrat placed within the upper right-hand corner of each 1-m^2 quadrat. We scored the presence vs absence of hydrophilic sedges by recording the presence or absence of herbaceous plants that had three-ranked leaves, an angular stem, and a spiked fruiting body. At each transect, we collected three evenly spaced soil samples with a soil probe (AMS 7/8 in. [2.2 cm] diameter open-end probe) at a minimum depth of 43 cm and a maximum depth of 66 cm. These depths reflect the column of soil the crayfishes are using for burrow construction (Robison and Crump 2004 and validated in the field). We analyzed the soil samples with laser diffraction on a Malvern Mastersizer 3000 (Malvern Instruments, Malvern, UK) to obtain a percent composition (sand, silt, and clay) for each sample. We computed the isometric log-ratio transformation for these soil data (Egozcue et

al. 2003). We constructed a soil texture plot with the package soiltexture in R (Fig. 4; Moeys 2015). The selected habitat variables reflect habitat characteristics associated with *F. harpi* (Robison and Crump 2004) and *P. reimeri* (Robison 2008), other primary burrowing crayfish species (Hobbs 1981, Welch and Eversole 2006, Loughman et al. 2012), and biological intuition. The habitat variables and a description of each model term used in the statistical analysis are shown in **Table 2.1**.

We mapped the quadrat locations in ArcGIS (version 10.2; Environmental System Research Institute, Redlands, California). We then calculated the following habitat variables for each quadrat with ArcGIS: elevation, distance to nearest waterbody, compound topographic index value (CTI), and solar radiation value. We measured elevation as the height in meters above sea level and distance to nearest waterbody as the Euclidean distance from all permanent waterbodies. We assessed CTI with the Geomorphometry and Gradient Metrics toolbox for ArcGIS (version a1.0; Evans et al. 2010). This metric is a function of both the slope and the upstream contributing area per unit width orthogonal to the flow direction. CTI is a steady state wetness index, where a larger CTI value represents areas that are topographically suitable for water accumulation. We measured solar radiation by calculating the watt-hour/m^2 of the delineated sampling area using the Area Solar Radiation tool in ArcMap. We calculated all ArcGIS values with digital elevation maps (National Elevation Dataset; http://ned.usgs.gov/) and surface water maps (National Hydrography Dataset; http://nhd.usgs.gov/index.html) at a resolution of 10 m in an attempt to minimize autocorrelation. We combined the habitat variables collected in the field and those calculated with ArcGIS into one data set for a fine-scale analysis of habitat features affecting crayfish burrow placement on the landscape.

Modeling analysis

We conducted all fine-scale statistical analyses in R (version 3.1.1; R Project for Statistical Computing, Vienna, Austria). We made the isometric log-ratio transformations with the package compositions (van den Boogaart et al. 2014) and used generalized linear mixed models to analyze the data (package lme4; Bates et al. 2014). The response variable in each model was the number of burrows within each 1-m^2 quadrat and was modeled with a Poisson error distribution and log link. We modeled burrow counts separately for each species. To account for potential site effects, we modeled transects nested within sites as a random effect in each model**.** We scaled and centered all habitat variables by subtracting the variable mean from each respective value and dividing by the standard deviation of that variable. We tested for overdispersion for each model before comparison of all models. We assessed model convergence and fit and then adjusted the optimization algorithm as needed. We did not include covariates having a Spearman correlation coefficient of >0.60 in the confined candidate model set in an attempt to avoid multicollinearity of our variables in the modeling suite. The full candidate model set and each hypothesis tested is shown in **Table 2.2**. We compared candidate models with Akaike's Information Criterion corrected for small sample sizes (AICc; Akaike 1974). We examined the relative support for each model and calculated unbiased model-averaged parameter estimates from the top models (ΔAICc < 4) with the package MuMIn (Barton 2014) by means of model selection and averaging methods described by Burnham and Anderson (2002) and Luckacs et al. (2009).

RESULTS

Field collections

Our search of museum databases resulted in 57 unique historic capture records (24 for *F. harpi* and 33 for *P. reimeri*). The records ranged from 1967 to 2008, and the oldest record we visited was from 1973. We sampled 11 of these localities (35 transects, 210 quadrats) for *F. harpi* and 9 of these localities (37 transects, 222 quadrats) for *P. reimeri*. Most (75%) of these localities were in the ROW of secondary, local, and private roads. Other sampling sites (25%) were situated in yards, pastures, and adjacent habitat farther from the ROW (up to 90 m). *Fallicambarus harpi* was present at all 11 sites (20 transects, 58 quadrats). *Procambarus reimeri* was present at 8 of the 9 sites sampled (23 transects, 52 quadrats). In total, we counted 143 burrows for *F. harpi* and 71 burrows for *P. reimeri*.

Modeling analysis

Fallicambarus harpi and *P. reimeri* had similar patterns of habitat selection. For both species, canopy cover was the most important habitat variable, and it was present in all top models (ΔAICc < 4, **Table 2.3**). Model-averaged parameter estimates for both species are shown in **Table 2.4**. The number of burrows in a quadrat was negatively associated with canopy cover (**Fig. 2.2A, 2.3A**) while the presence of hydrophilic sedges was positively associated with the number of burrows in a quadrat (**Fig 2.2B, 2.3B**). The transformed soil variables and stem density variable were also present in the top models. Burrows were generally present in quadrats with little to no canopy cover (mean ± SD, 4.4% ± 17.7, *n* = 110). No burrow was observed in a quadrat with complete canopy cover (100%). Sedges were present in 83% of the quadrats that harbored burrows of either species (*n* = 110).

DISCUSSION

We developed a suite of models to assess our predictions regarding the habitat preferences of *F. harpi* and *P. reimeri*. We found support for some of our predictions, whereas some results were counterintuitive. Open-canopy habitat and the presence of sedges were important for burrow placement across the sampled landscape. Our predictions that these variables would be preferred by both species of crayfish were supported by the models fitted with generalized linear mixed-model analysis. The presence of hydrophilic sedges is an indication of a seepage area or that the water table is relatively close to the ground surface (Schütz 2000). The absence of tree canopy cover also contributes to these wet seepage areas (Eastham et al. 1994). Our prediction that soil would be a strong predictor was not supported. This outcome was potentially a result of the way we spatially segregated our soil samples. We collected soil samples that fell into only three distinct soil textural classes (silt loam, loam, and sandy loam; **Fig. 2.4**), which did not capture the variation seen across the entire OME. Overall, however, our findings point to the preference of ROW-like habitat for *F. harpi* and *P. reimeri*. The habitat in which animals were most abundant was treeless, wet seepage areas with abundant low grasses and sedges. The soil composition at these occupied sites was primarily loam and silt loam (Soil Survey Division Staff 1993; 90% of *F. harpi* quadrats, 92% of *P. reimeri* quadrats). The burrows of *F. harpi* and *P. reimeri* were complex, 0.5–1 m in depth, and connected to groundwater. Our results highlight the specific importance of these wet, open-canopy habitats as a preferred environment for both species.

Previous studies of other primary burrowing crayfish species have revealed the existence of habitat specialists and habitat generalists. Specialist species occur in habitats ranging from pitcher plant bogs (*Fallicambarus gordoni* [Johnston and Figiel 1997]) to sand ridges

(*Distocambarus crokeri* [Welch and Eversole 2006]), whereas generalist species such as *Procambarus gracilis* (Hobbs and Rewolinski 1985), *Fallicambarus devastator* (Hobbs and Whiteman 1991), *Fallicambarus fodiens* (Norrocky 1991), *Cambarus catagius* (Mcgrath 1994), *Cambarus dubius* (Loughman 2010), and *Cambarus thomai* (Loughman et al. 2012) can be found in both forested floodplains and open habitat throughout their respective ranges. Based on the modeling and field observations, *F. harpi* and *P. reimeri* can be considered habitat specialists. They occur in wet, open herbaceous areas and not in the adjacent forested habitat. We believe the microhabitat of the roadside ditch is acting as suitable habitat for these specialists within a matrix of unsuitable habitat.

We sampled transects adjacent to known localities to better model habitat preference for each species. The habitats sampled by these transects generally differed in composition from the ROW (**Appendix A**; **Table A.1**) but were spatially proximate so as to be accessible to crayfishes. These sites composed 25% of the sampling locations and were not in the ROW. We designed this sampling scheme with the knowledge that it would be unnecessary and cost ineffective to sample the entirety of the OME randomly. Primary burrowing crayfishes rarely, if ever, inhabit permanent open water (Hobbs 1981) such as streams, lakes, and swamps, or high-gradient slopes found in the larger OME. A review of over 2000 freshwater crayfish collections made in the state of Arkansas (Illinois Natural History Survey Crustacean Collection and National Museum of Natural History Invertebrate Zoology Collection) revealed no observations of *F. harpi* or *P. reimeri* in either of these habitat types. Thus, we think the microhabitat available to primary burrowing crayfishes is spatially restricted because of their life-history characteristics (Hobbs 1981), and those available habitats were represented in our sampling design.

The microhabitat found in roadside ditches where these animals occur is a result of the physical presence of the road and roadside maintenance. The surface of the road is less permeable than the surrounding habitat, which diverts precipitation into the surrounding terrain (MacDonald et al. 2001). The roadside ditch also intercepts groundwater flow, adding more water to the roadside microhabitat (Forman et al. 2003). Roadside maintenance halts succession by removing woody stems and constantly disturbs the herbaceous community with mowing and herbicide application. The removal of woody stems also increases the soil moisture in the roadside habitat (Eastham et al. 1994) because woody stems have deeper root systems and are capable of transpiring more water from the soil than are herbaceous plants. Through these road-maintenance activities, the ROW habitat is uniformly distributed where the road occurs. These attributes of the roadside appear to have created suitable habitat for these two species of primary burrowing crayfishes.

The characteristics of animal populations vulnerable to negative road effects have been documented as having a high level of mobility, behaviorally avoiding roads, and being habitat generalist or forest interior species. The negative responses observed in the biota that have these characteristics are not seen in *F. harpi* or *P. reimeri*. These two species do not show a high level of mobility or a behavioral avoidance of roads: some of the ROW sites revisited in our study were first discovered >40 years ago. These animals are also not perceived as being habitat generalists or forest interior species. They were observed in open, wet, herbaceous areas, which suggests these species are habitat specialists within the broader matrix of forested habitat that dominates the OME. They may also be avoiding other direct negative effects of inhabiting ROWs by spending most of their life underground. Other species of burrowing crayfishes have

been observed evading the effects of pesticide use by occupying a buffer zone within their burrows during pesticide application (Sommer 1983).

Our findings add to the understanding of the interactions between ROWs and the biota that live within them. Previous research has shown positive and negative responses of biota to ROWs (e.g. Adams and Geis 1983, Forman et al. 2003, Fahrig and Rytwinski 2009). Our study is the first to show a positive interaction between a narrowly endemic habitat specialist and a ROW habitat that is commonly seen as highly altered and detrimental to endemic wildlife populations. We are confident that we captured different potential habitat types available to *F. harpi* and *P. reimeri* by sampling the adjacent habitat. The adjacent habitats were out of the ROW and generally did not have the habitat characteristics of the roadside ditch. We think these crayfishes prefer the ROW microhabitat because of the lack of canopy and presence of sedges, which presents a moist, low-herbaceous environment. These data support the benefit of ROWs to the persistence of these narrowly endemic habitat specialists. The use of this habitat by these species could also encourage dispersal along these linear corridors. Future work is needed to assess this possibility and to investigate locations within the OME within and well beyond the roadside ditch, where these animals are not known to occur.

TABLES AND FIGURES

Figure 2.1. Sites sampled for *Fallicambarus harpi* and *Procambarus reimeri* in Arkansas

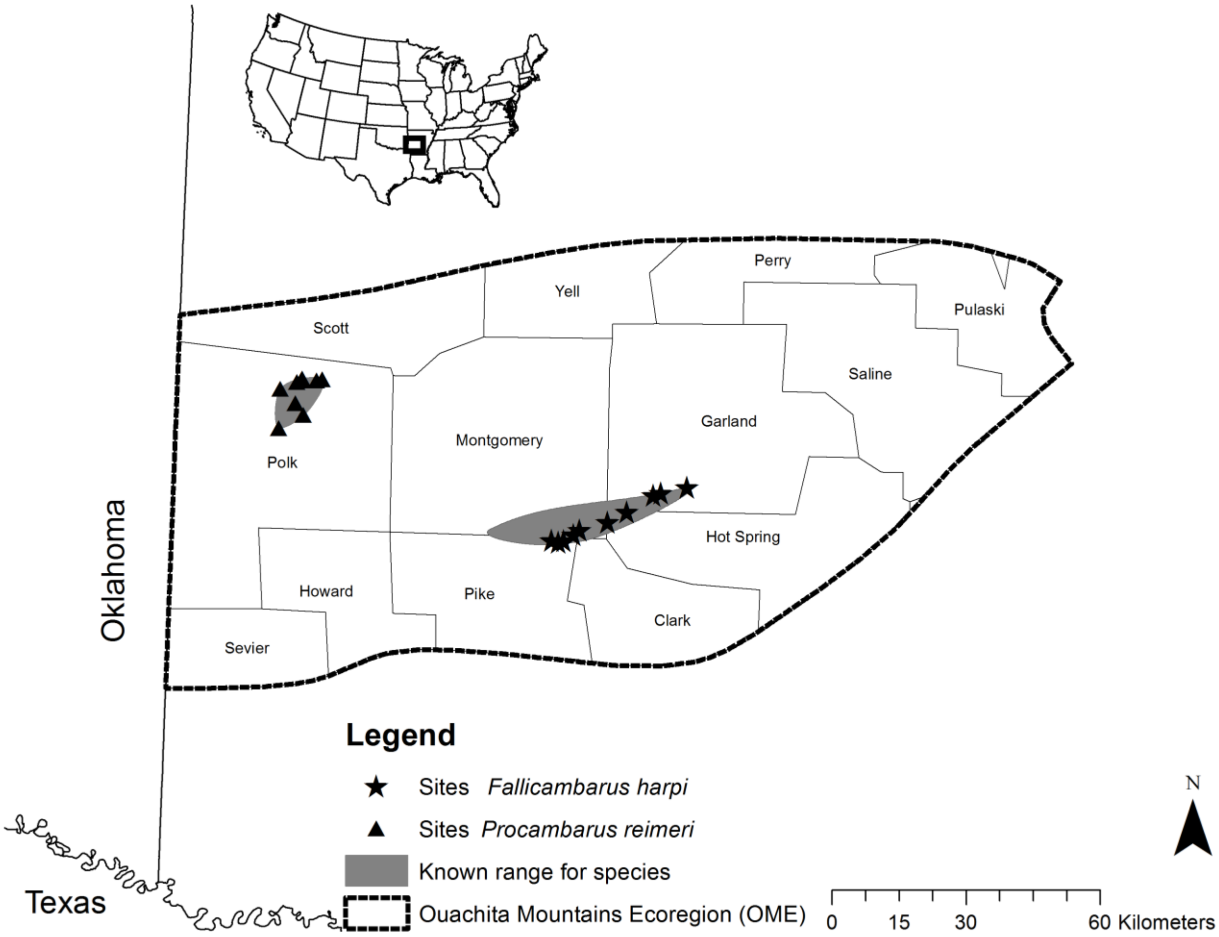

Figure 2.2: Estimated number of *Fallicambarus harpi* burrows/m^2 in relation to the percent tree canopy cover over a quadrat (A). The shaded area indicates the 95% confidence interval (CI) of burrow counts in relation to the variable canopy. Mean (95% CI) number of burrows in quadrats with and without sedges (B).

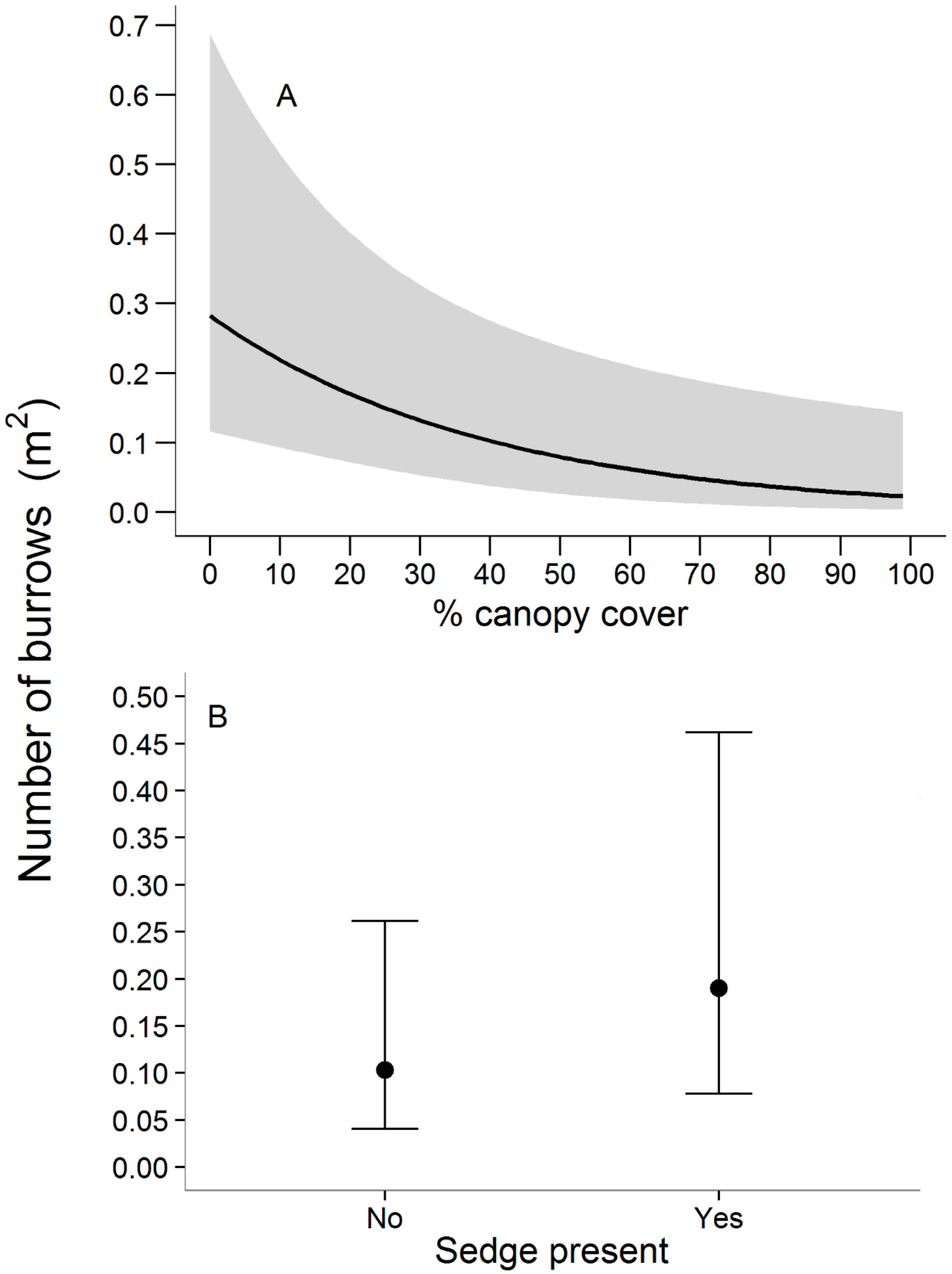

Figure 2.3: Estimated number of *Procambarus reimeri* burrows/m^2 in relation to the percent tree canopy cover over a quadrat (A). The shaded area indicates the 95% confidence interval (CI) of burrow counts in relation to the variable canopy. Mean (95% CI) number of burrows in quadrats with and without sedges (B).

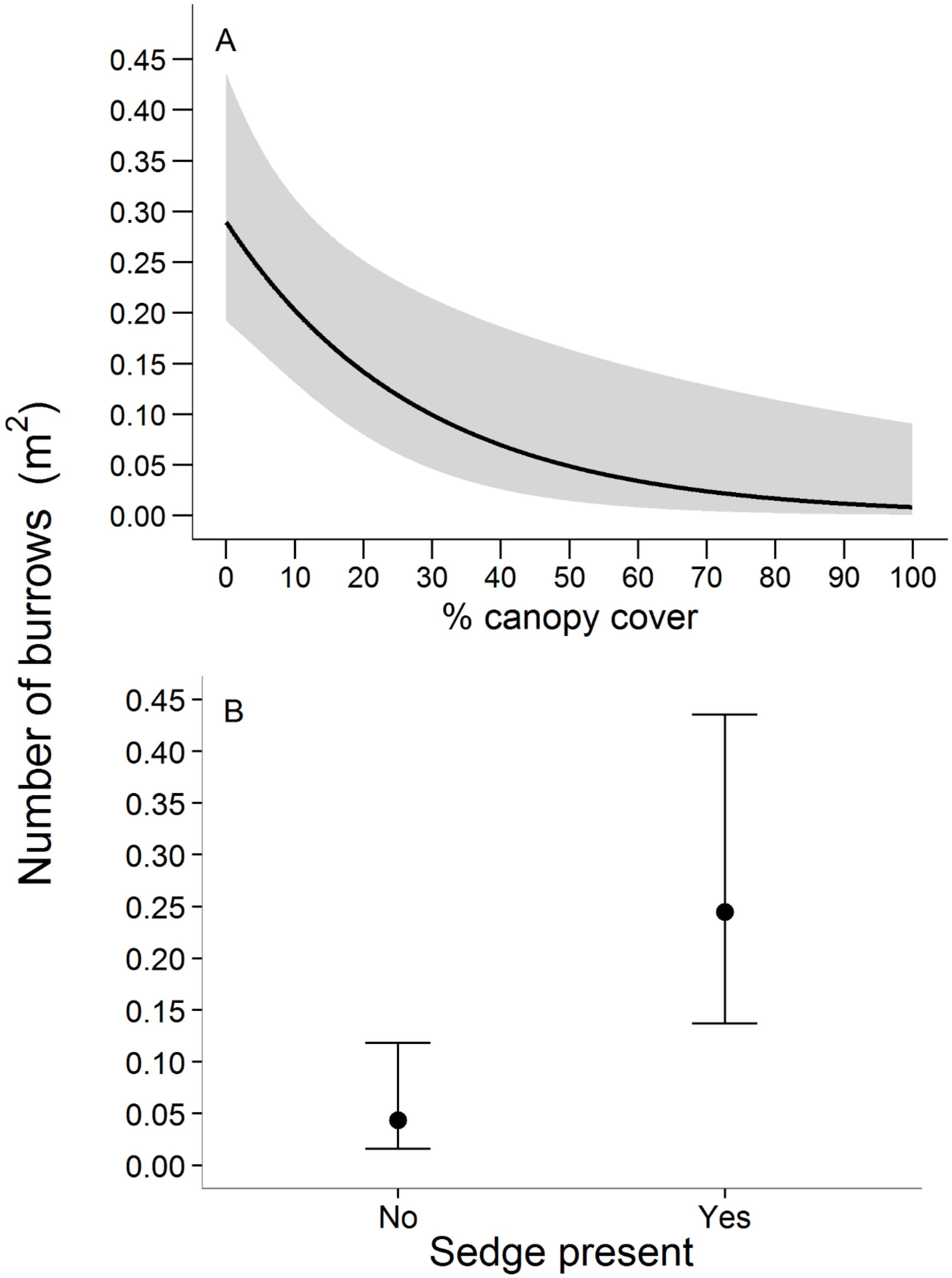

Figure 2.4: Soil texture plot for soil samples collected in the spring 2014. Cl = clay, SiCl = silty clay, SaCl = sandy clay, ClLo = clay loam, SiClLo = silty clay loam, SaClLo = sandy clay loam, Lo = loam, SiLo = silty loam, SaLo = sandy loam, Si = silt, Sa = sand, and LoSa = loamy sand. Texture classes follow those of US Department of Agriculture (Soil Survey Staff 1993). Classes obscured by data points are loam (lower center of figure) and silty loam (lower right of figure).

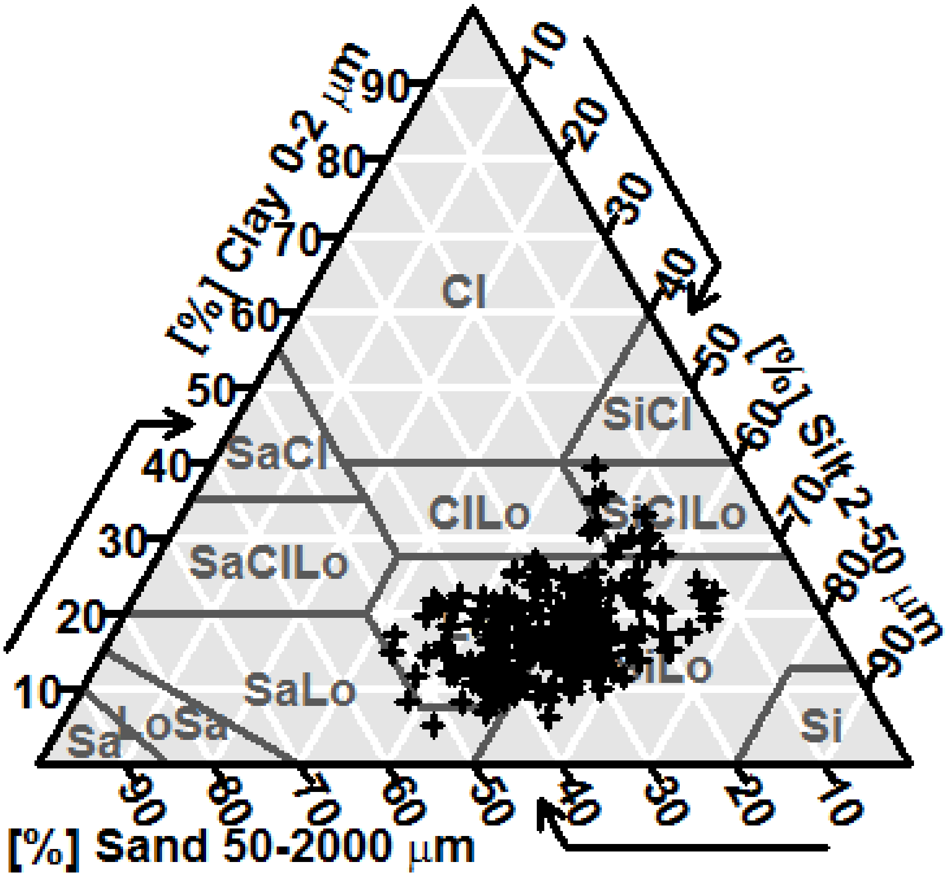

Table 2.1: Variables and their descriptions for a generalized linear mixed-model analysis of the habitat of two primary burrowing crayfishes in Arkansas (*Fallicambarus harpi* and *Procambarus reimeri*). Quadrats were 1 m^2.

Variable	Description
**Water_trans**	Presence of standing water in the length of the 50-m transect (binary: yes/no)
**Sedge**	Presence of hydrophilic sedge in quadrat (binary: yes/no)
**Canopy**	% tree canopy cover taken at each quadrat
**Herb**	% herbaceous ground cover taken at each quadrat
**Stem**	Stem-density count taken at each quadrat
**Elevation**	Elevation calculated at each quadrat location (National Elevation Dataset, resolution 10 m; http://ned.usgs.gov/)
**Solar**	Incoming solar radiation value (watt-hour/m^2) calculated at each quadrat location based on direct and diffuse insolation from the unobstructed sky directions (ArcGIS, Environmental System Research Institute, Redlands, California)
**Water_dist**	Euclidean distance to nearest waterbody calculated for each quadrat location (National Hydrography Dataset; http://nhd.usgs.gov/index.html)
**CTI**	Compound topographic index value calculated for each quadrat location (Evans et al. 2010)
**Soil1, Soil2**	Transformed soil composition (% sand, silt, clay) value calculated for each soil sample (van den Boogaart et al. 2014)

Table 2.2: Candidate models and hypotheses tested in the generalized linear mixed-model analysis for *Fallicambarus harpi* and *Procambarus reimeri* in Arkansas. The response variable used in each model was burrow abundance in each 1-m^2 quadrat. See Table 2.1 for variable names.

Model name:	Variables	Hypothesis
**Mod 1(global)**	Water_trans + sedge + canopy + herb + stem + elevation + solar + water_dist + CTI + soil1 + soil2	Crayfish selection based on transect- and quadrat-level wetness characteristics, canopy cover, herbaceous community, erosion potential, topographic position, and soil cues
**Mod 2**	Canopy + sedge	Crayfish selection based on canopy cover and quadrat-level wetness
**Mod 3**	Water_trans + sedge + solar + water_dist	Crayfish selection based on transect- and quadrat-level wetness characteristics and topographic position
**Mod 4**	Water_trans + sedge + CTI	Crayfish selection based on transect-and quadrat-level wetness characteristics and topographic position
**Mod 5**	Water_dist + CTI	Crayfish selection based on topographic position
**Mod 6**	Solar + CTI	Crayfish selection based on topographic position
**Mod 7**	Elevation + water_dist	Crayfish selection based on topographic position
**Mod 8**	Sedge + stem	Crayfish selection based on herbaceous community
**Mod 9**	Canopy + herb	Crayfish selection based on canopy and herbaceous community
**Mod 10**	Herb + soil1 + soil2	Crayfish selection based on herbaceous community and soil cues
**Mod 11**	Canopy + soil1 + soil2 + sedge	Crayfish selection based on canopy cover, soil cues, and quadrat-level wetness
**Mod 12**	Canopy + solar + soil1 + soil2	Crayfish selection based on canopy cover, solar radiation potential, and soil cues
**Mod 13**	Canopy + sedge + stem	Crayfish selection based on canopy cover, quadrat-level wetness, and erosion potential
**Mod 14**	Soil1 + soil2 + water_trans	Crayfish selection based on soil cues and transect-level wetness
**Mod 15**	Canopy	Crayfish selection based on canopy cover
**Mod 16**	Soil1 + soil2	Crayfish selection based on soil cues
**Mod 17**	Solar	Crayfish selection based on solar radiation potential

Table 2.3: Model name, number of model parameters (K), Akaike's Information Criterion adjusted for small sample size (AICc), difference in AICc (ΔAICc), Akaike weights (w_i), and log likelihood (LL) for the top habitat models (ΔAICc < 4) from a suite of variables modeled with a generalized linear mixed-model analysis for two primary burrowing crayfish species, *Fallicambarus harpi* (*n* = 210 quadrats) and *Procambarus reimeri* (*n* = 222 quadrats) in Arkansas. See Tables 2.1 and 2.2 for a description of each model and the variables included.

Model	K	AICc	ΔAICc	w_i	LL
			Fallicambarus harpi		
**Mod 11**	7	374.63	0	0.50	−180.04
**Mod 2**	5	375.99	1.36	0.26	−182.85
**Mod 13**	6	377.92	3.29	0.10	−182.76
			Procambarus reimeri		
**Mod 2**	5	285.6	0	0.67	−137.66
**Mod 13**	6	287.7	2.11	0.23	−137.66
**Mod 11**	7	289.42	3.82	0.1	−137.45

Table 2.4: Unbiased model-averaged parameter estimates of the top models (Table 2.3) for two primary burrowing crayfishes species (*Fallicambarus harpi* and *Procambarus reimeri*) in Arkansas. See Table 2.1 for a description of the variables included. Sedge1 = presence of sedge in quadrat, CL = confidence limits.

Variable	Model-averaged estimate (SE)	95% CL	p > │z│
	Fallicambarus harpi		
**Canopy**	−1.003 (0.3863)	−1.761, −0.246	0.009
**Sedge1**	0.546 (0.239)	0.077, 1.015	0.071
**Stem**	0.045 (0.105)	−0.161, 0.250	0.899
**Soil1**	0.1436 (0.125)	−0.102, 0.388	0.509
**Soil2**	−0.293 (0.127)	−0.542, −0.044	0.364
**Intercept**	−2.183 (0.485)	−3.135, −1.232	–
	Procambarus reimeri		
**Canopy**	−1.317 (0.468)	−2.234, −0.401	0.005
**Sedge1**	1.727 (0.474)	0.797, 2.656	0.0002
**Stem**	0.003 (0.073)	−0.281, 0.306	0.968
**Soil1**	0.003 (0.047)	−0.258, 0.320	0.948
**Soil2**	0.007 (0.059)	−0.272, 0.414	0.905
**Intercept**	−3.135 (0.513)	−4.140, −2.130	–

LITERATURE CITED

Adams, L. W., and A. D. Geis. 1983. Effects of roads on small mammals. *Journal of Applied Ecology* 20:403–415.

Akaike, H. 1974. A new look at the statistical model identification. IEEE Transactions on Automatic Control 19:716–723.

Altrichter, M., and G. I. Boaglio. 2004. Distribution and relative abundance of peccaries in the Argentine Chaco: associations with human factors. Biological Conservation 116:217–225.

Andrews, A. 1990. Fragmentation of habitat by roads and utility corridors: a review. Australian Zoologist 26:130–141.

Ashley, E. P., and J. T. Robinson. 1996. Road mortality of amphibians, reptiles and other wildlife on the Long Point Causeway, Lake Erie, Ontario. Canadian Field Naturalist 110:403–412.

Barbaresi, S., E. Tricarico, and F. Gherardi. 2004. Factors inducing the intense burrowing activity of the red-swamp crayfish, *Procambarus clarkii*, an invasive species. Die Naturwissenschaften 91:342–345.

Barton, K. 2014. MuMIn: multi-model inference. R Project for Statistical Computing, Vienna, Austria. (Available from: http://cran.r-project.org/package=MuMIn)

Bates, D., M. Maechler, B. Bolker, and S. Walker. 2014. lme4: linear mixed-effects models using 'Eigen' and S4. R Project for Statistical Computing, Vienna, Austria. (Available from: http://cran.r-project.org/package=lme4)

Berger, R. L. 2005. Integrated roadside vegetation management: a synthesis of highway practice. NCHRP Synthesis 341. Transportation Research Board, Washington, DC. (Available from: http://www.trb.org/Publications/Blurbs/155791.aspx)

Berrill, M., and B. Chenoworth. 1982. The burrowing ability of nonburrowing crayfish. American Midland Naturalist 108:199–201.

Burnham, K. P., and D. R. Anderson. 2002. Model selection and multimodel inference: a practical information-theoretic approach. 2nd edition. Springer, New York.

Eastham, J., P. R. Scott, and R. Steckis. 1994. Components of the water balance for tree species under evaluation for agroforestry to control salinity in the wheatbelt of Western Australia. Agroforestry Systems 26:157–169.

Egozcue, J. J., V. Pawlowsky-Glahn, G. Mateu-Figueras, and C. Barceló-Vidal. 2003. Isometric logratio transformations for compositional data analysis. Mathematical Geology 35:279–300.

Evans, J., J. Oakleaf, S. Cushman, and D. Theobald. 2010. An ArcGIS toolbox for surface gradient and geomorphic modeling, version a1.0. (Available from: http://evansmurphy.wix.com/evansspatial#!arcgis-gradient-metrics-toolbox/crro)

Fahrig, L., and T. Rytwinski. 2009. Effects of roads on animal abundance: an empirical review and synthesis. Ecology and Society 14:397–405.

Forman, R. T. T., D. Sperling, J. A. Bissonette, A. P. Clevenger, C. D. Cutshall, V. H. Dale, L. Fahrig, R. France, C. R. Goldlman, K. Heanue, J. A. Jones, F. J. Swanson, T. Turrentine, and T. C. Winter. 2003. Road ecology: science and solutions. Island Press, Washington, DC.

Gelbard, J. L., and J. Belnap. 2003. Roads as conduits for exotic plant invasions in a semiarid landscape. Conservation Biology 17:420–432.

Grow, L., and H. Merchant. 1980. The burrow habitat of the crayfish, *Cambarus diogenes diogenes* (Girard). American Midland Naturalist 103:231–237.

Helms, B., W. Budnick, P. Pecora, J. Skipper, E. Kosnicki, J. Feminella, and J. Stoeckel. 2013. The influence of soil type, congeneric cues, and floodplain connectivity on the local distribution of the devil crayfish (*Cambarus diogenes* Girard). Freshwater Science 32:1333–1344.

Hlass, L. J., W. L. Fisher, and D. J. Turton. 1998. Use of the index of biotic integrity to assess water quality in forested streams of the Ouachita Mountains Ecoregion, Arkansas. *Journal of Freshwater Ecology* 13:181–192.

Hobbs, H. H. 1981. The crayfishes of Georgia. Smithsonian Contributions to Zoology 318. Smithsonian Institution Press, Washington, DC.

Hobbs, H. H., and S. A. Rewolinski. 1985. Notes on the burrowing crayfish *Procambarus* (*Girardiella*) *gracilis* (Bundy) (Decapoda, Cambaridae) from Southeastern Wisconsin, U.S.A. Crustaceana 48:26–33.

Hobbs, H. H., and M. Whiteman. 1991. Notes on the burrows, behavior, and color of the crayfish *Fallicambarus* (*F.*) *devastator* (Decapoda: Cambaridae). Southwestern Naturalist 36:127–135.

Johnston, C. E., and C. Figiel. 1997. Microhabitat parameters and life-history characteristics of *Fallicambarus gordoni* Fitzpatrick, a crayfish associated with pitcher-plant bogs in Southern Mississippi. *Journal of Crustacean Biology* 17:687–691.

Lode, T. 2000. Effect of a motorway on mortality and isolation of wildlife populations. *AMBIO: A Journal of the Human Environment* 29:163–166.

Loughman, Z. J. 2010. Ecology of *Cambarus dubius* (upland burrowing crayfish) in north-central West Virginia. Southeastern Naturalist 9:217–230.

Loughman, Z. J., T. P. Simon, and S. A. Welsh. 2012. Occupancy rates of primary burrowing crayfish in natural and disturbed large river bottomlands. *Journal of Crustacean Biology* 32:557–564.

Luckacs, P. M., K. P. Burnham, and D. R. Anderson. 2009. Model selection bias and Freedman's paradox. Annals of the Institute of Statistical Mathematics 62:117–125.

MacDonald, L. H., R. W. Sampson, and D. M. Anderson. 2001. Runoff and road erosion at the plot and road segment scales, St John, US Virgin Islands. Earth Surface Processes and Landforms 26:251–272.

Mcgrath, C. 1994. Status survey for the Greensboro burrowing crayfish. Proceedings of the Annual Conference of the Southeastern Association of Fish and Wildlife Agencies 48:343–349.

Miser, H. D. 1929. Structure of the Ouachita Mountains of Oklahoma and Arkansas. Oklahoma Geological Survey Bulletin 50:1–30.

Moeys, J. 2015. soiltexture: Functions for Soil Texture Plot, Classification and Transformation. R package version 1.3.3. http://CRAN.R-project.org/package=soiltexture

Norrocky, M. J. 1991. Observations on the ecology, reproduction and growth of the burrowing crayfish *Fallicambarus* (*Creaserinus*) *fodiens* (Decapoda: Cambaridae) in north-central Ohio. American Midland Naturalist 125:75–86.

Oxley, D. J., M. B. Fenton, and G. R. Carmody. 1974. The effects of roads on populations of small mammals. *Journal of Applied Ecology* 11:51–59.

Peris, S. J., and M. Pescador. 2004. Effects of traffic noise on passerine populations in Mediterranean wooded pastures. Applied Acoustics 65:357–366.

Phillips, J. D., and D. A. Marion. 2005. Biomechanical effects, lithological variations, and local pedodiversity in some forest soils of Arkansas. Geoderma 124:73–89.

Pocock, Z., and R. E. Lawrence. 2005. How far into a forest does the effect of a road extend? Defining road edge effect in eucalypt forests of south-eastern Australia. Pages 397–405 *in* C. L. Irwin, P. Garrett, and K.P. McDermott (editors). Proceedings of the 2005 International Conference on Ecology and Transportation. Center for Transportation and the Environment, North Carolina State University, Raleigh, North Carolina.

Richardson, J. H., R. F. Shore, and J. R. Treweek. 1997. Are major roads a barrier to small mammals? *Journal of Zoology* 243:840–846.

Riley, S. P. D., J. P. Pollinger, R. M. Sauvajot, E. C. York, C. Bromley, T. K. Fuller, and R. K. Wayne. 2006. A southern California freeway is a physical and social barrier to gene flow in carnivores. Molecular Ecology 15:1733–1741.

Robison, H. W. 2008. Distribution, life history aspects, and conservation status of three Ouachita Mountain crayfishes: *Procambarus tenuis*, *P. reimeri*, and *Orconectes menae*. U.S.D.A. Forest Service, Ouachita National Forest. Hot Springs, Arkansas.

Robison, H. W., and B. Crump. 2004. Distribution, natural history aspects, and status of the Arkansas endemic crayfish, *Fallicambarus harpi* Hobbs and Robison, 1985. *Journal of the Arkansas Academy of Science* 58:91–94.

Rosa, S., and J. Bissonette. 2007. Roads and desert small mammal communities: positive interaction? Pages 351–365 *in* C. L. Irwin, D. Nelson, and K.P. McDermott (editors). Proceedings of the 2007 International Conference on Ecology and Transportation. Center for Transportation and the Environment, North Carolina State University, Raleigh, North Carolina.

Rytwinski, T., and L. Fahrig. 2013. Why are some animal populations unaffected or positively affected by roads? Oecologia (Berlin) 173:1143–1156.

Schütz, W. 2000. Ecology of seed dormancy and germination in sedges (*Carex*). Perspectives in Plant Ecology, Evolution, and Systematics 3:67–89.

Semlitsch, R. D., T. J. Ryan, K. Hamed, M. Chatfield, B. Drehman, N. Pekarek, M. Spath, and A. Watland. 2007. Salamander abundance along road edges and within abandoned logging roads in Appalachian forests. Conservation Biology 21:159–167.

Soil Survey Division Staff. 1993. Soil survey manual. Handbook 1. Soil Conservation Service, US Department of Agriculture, Washington, DC. (Available from: http://www.nrcs.usda.gov/Internet/FSE_DOCUMENTS/nrcs142p2_050993.pdf)

Sommer, T. R. 1983. Laboratory and field studies on the toxic effects of thiobencarb (Bolero) to the crawfish (*Procambarus clarkii*). *Journal of the World Mariculture Society* 14:434–440.

Spellerberg, I. F. 1998. Ecological effects of roads and traffic: a literature review. Global Ecology and Biogeography 7:317–333.

Taylor, C. A. , G. A. Schuster, J. E. Cooper , R. J. DiStefano, A. G. Eversole, P. Hamr, H. H. Hobbs III, H. W. Robison, C. E. Skelton, and R. F. Thoma. 2007. A Reassessment of the

Conservation Status of Crayfishes of the United States and Canada after 10+ Years of Increased Awareness. Fisheries 32:8 372-389.

Thorpe, A., and R. M. Harrison. 2008. Sources and properties of non-exhaust particulate matter from road traffic: a review. Science of the Total Environment 400:270–282.

van den Boogaart, K. G., R. Tolosana, and M. Bren. 2014. compositions: compositional data analysis. R Project for Statistical Computing, Vienna, Austria. (Available from: http://cran.r-project.org/package=compositions)

Ward, R. L., J. T. Anderson, and J. T. Petty. 2008. Effects of road crossings on stream and streamside salamanders. *Journal of Wildlife Management* 72:760–771.

Watkins, R. Z., J. Chen, J. Pickens, and K. D. Brosofske. 2003. Effects of forest roads on understory plants in a managed hardwood landscape. Conservation Biology 17:411–419.

Welch, S. M., and A. G. Eversole. 2006. The occurrence of primary burrowing crayfish in terrestrial habitat. Biological Conservation 130:458–464.

Williams, J. D., M. L. Warren Jr., K. S. Cummings, J. L. Harris, and R. J. Neves. 1993. Conservation status of freshwater mussels of the United States and Canada. Fisheries 18(9):6-22.

Woods, A. J., T. L. Foti, S. S. Chapman, J. M. Omernik, J. A. Wise, E. O. Murray, W. L. Prior, J. B. J. Pagan, J. A. Comstock, and M. Radford. 2004. Ecoregions of Arkansas (color poster with map, descriptive text, summary tables, and photographs). US Geological Survey map scale 1:1,000,000. US Geological Survey, Reston, Virginia. (Available from: http://archive.epa.gov/wed/ecoregions/web/html/ar_eco.html)

CHAPTER 3: FIELD VALIDATION OF MAXENT MODELING OF TWO NARROWLY ENDEMIC CRAYFISHES

INTRODUCTION

Understanding the factors influencing species distributions and habitat selection are critical to researchers (Baldwin 2009). For example, rare or narrowly endemic plants and animals are difficult to monitor and conserve when their total distribution and habitat preferences are not completely known. One method used in this research is species distribution models (SDMs). SDMs are correlative models using environmental and/or geographic information to explain observed patterns of species occurrences (Elith and Graham 2009). SDMs can provide useful information for exploring and predicting species distributions across the landscape (Elith et al. 2011). Models estimated from species observations can also be applied to produce measures of habitat suitability (Franklin 2013). This information can be useful for detecting unknown populations of rare, endemic, or threatened species (e.g. Williams et al. 2009, Rebelo and Jones 2010, Peterman et al. 2013, Searcy and Shaffer 2014, Fois et al. 2015). Due to the wide range of applications and open-access spatial databases (e.g. National Elevation Dataset http://ned.usgs.gov/ and National Hydrography Dataset http://nhd.usgs.gov/index.html), SDMs rank among the most widely reviewed topics in the ecological literature (Araújo and Peterson 2012). SDMs can also limit search efforts by selecting suitable sampling areas *a priori*, leading to a cost-effective and efficient use of sampling effort (Fois et al. 2015). One of the most widely used SDMs in recent years is the program Maxent (Kramer-Schadt et al. 2013).

Maxent is a presence-only modeling algorithm using a set of known occurrences together with predictor variables such as topographic, climatic, edaphic, biogeographic, and remotely sensed variables (Phillips et al. 2006, Phillips and Dudík 2008). These data are used to predict the relative occurrence rate of a focal species across a predefined landscape (Fithian and Hastie

2013). Recent studies focusing on the performance of Maxent have revealed it to perform well in comparison to other SDMs (Elith et al. 2006). Maxent also performs well with small sample sizes (e.g. Hernandez et al. 2006, Pearson et al. 2007, Wisz et al. 2008), rare species (e.g. Williams et al. 2009, Rebelo and Jones 2010), narrowly endemic species (e.g. Rinnhofer et al. 2012), and when used as a habitat suitability index (Latif et al. 2015).

In all of the above applications, the potential for the inaccurate execution and interpretation of an SDM is well documented (e.g. Araújo and Guisan 2006, Baldwin 2009, Yackulic et al. 2013, Guillera-Arroita et al. 2014). Specific issues surrounding the interpretation of Maxent analyses include sampling bias (Phillips and Dudík 2008, Boria et al. 2014) and the lack of techniques to assess model quality (Hijmans 2012), overfitting of model predictions (Elith et al. 2010, Warren and Seifert 2011), or assessment of detection probabilities (Lahoz-Monfort et al. 2014). With these issues in mind we created our models carefully: we used a null model approach to assess model quality (Raes and ter Steege 2007), we used the R package ENMeval (Muscarella et al. 2014) to balance model fit, and we sampled at the time of highest burrow detectability (spring 2015; Rhoden et al. *in press*). The utility of Maxent has also been burdened with issues of model validation (Hijmans 2012). Most model validation methods involve subsets of the input data with the predictions generated by the models (Rebelo and Jones 2010). Historically, validation of Maxent predictions has lacked an independent assessment of model performance (Greaves et al. 2006) such as a novel set of presence locations. The need for independent validation is especially important for rare species exhibiting a wider knowledge gap in distribution than more common species (Rebelo and Jones 2010). North American primary burrowing crayfishes are a group of poorly understood and understudied taxon for which SDMs

could provide novel insight into distributions and habitat relationships, and thus provide an excellent case study for validation.

North America has the highest diversity of crayfishes worldwide (Taylor et al. 2007). Within North America, 22% of the species listed as endangered or threatened in a recent conservation review of crayfishes were primary burrowing crayfishes (Taylor et al. 2007). These listings were based upon the American Fisheries Society Endangered Species Committee, which follows the criteria of Williams et al. (1993). It is hypothesized all crayfishes have the ability to construct refugia by way of burrowing down into the soil or substrate (Berrill and Chenoworth, 1982; Hobbs, 1981). Hobbs (1981) described three classes of burrowing crayfishes based on differences in natural history: tertiary, secondary, and primary burrowers. Tertiary burrowers dig shallow burrows only to escape frost or seek shelter and when the body of water they are inhabiting dries up. Secondary burrowers spend much of their lives in their burrows; however, they do move out into open water occasionally, and their burrows normally have a connection to an open, permanent water body. Primary burrowing crayfishes spend their complete life cycle underground. As primary burrowers leave their burrows only to forage and find a burrow of the opposite sex for mating (Hobbs 1981), their burrows are rarely tied to permanent open water.

Amongst the three types of burrowers, the least is known regarding the natural history of primary burrowing crayfishes (Moore et al. 2013; Taylor et al. 2007) due to the challenges in sampling these largely fossorial animals (Larson and Olden 2010). However, the narrowly endemic nature of North American crayfishes is well documented (Morehouse and Tobler, 2013; Page, 1985; Simmons and Fraley, 2010; Taylor et al. 2007). Primary burrowing crayfishes in Arkansas are no exception (Robison et al. 2008). Of the 12 species of primary burrowers in Arkansas (*Fallicambarus dissitus, F. fodiens, F. gilpini, F. harpi, F. jeanae, F. petilicarpus, F.*

strawni, *Procambarus curdi*, *P. liberorum*, *P. parasimulans*, *P. regalis,* and *P. reimeri*), ten (83%) are known from three or fewer ecoregions, with six (50%) known from only one ecoregion. The limited geographic distribution of any taxa makes them more vulnerable to localized extirpation. Because these animals occur at such a constrained geographic scale, it is important to understand and document the existing distribution to manage and preserve current populations.

The rarity of and difficulties surrounding the collection of natural history information, specifically habitat suitability, make primary burrowing crayfishes ideal candidates for SDM. To test the ability of SDMs to predict the distribution of suitable habitat for two narrowly endemic habitat specialists, we constructed SDMs for *F. harpi* and *P. reimeri* and validated the models using independent sampling data. These species are vulnerable to population declines and are currently recorded under the Endangered (*P. reimeri*) and Vulnerable (*F. harpi*) conservation status categories (Taylor et al. 2007) based on modifications to or reductions of habitat in their already restricted ranges. Additionally, these species were included in a recent petition filed by the privately funded Center for Biological Diversity for protection under the Federal Endangered Species Act.

METHODS

Study Area

Our study sites were located in the Ouachita and Caddo River drainages of southwestern Arkansas in the Ouachita Mountains Ecoregion (OME; Woods et al. 2004). The remnant pine-bluestem (*Pinus-Schizachyrium*) communities (Phillips and Marion 2005) and silty loam soil (Hlass et al. 1998) make this region of Arkansas ideal habitat for primary burrowing crayfishes

and in fact has the highest diversity of primary burrowing crayfishes in the entire state with six species (*Fallicambarus harpi*, *F. jeanae*, *F. strawni*, *Procambarus liberorum*, *P. parasimulans*, and *P. reimeri*). We sampled thirteen counties encompassing the known range of both species of primary burrowing crayfish: from east to west, those counties were Pulaski, Saline, Perry, Garland, Hot Spring, Clark, Yell, Montgomery, Pike, Scott, Howard, Polk, and Sevier (**Fig. 3.1**).

Presence data and environmental variables

To determine habitat requirements of *F. harpi* and *P. reimeri*, we queried natural history museums or databases (Illinois Natural History Survey Crustacean Collection, the National Museum of Natural History Smithsonian Institution, and the Arkansas Department of Natural Heritage) for historic locations of both species, and a subset of those locations were visited (Rhoden et al. *in press*). At each location, we measured the following suite of habitat variables: percent tree canopy cover, percent herbaceous ground cover, stem density, the number of burrows, presence of standing water at the site, remotely sensed variables and the presence or absence of hydrophilic sedges. We found canopy cover and the presence of hydrophilic sedges were the most important factors in predicting crayfish abundance (Rhoden et al. *in press*).

The environmental variables used for the SDM analysis consisted of canopy cover, elevation, distance to nearest waterbody, compound topographic index value (CTI), and solar radiation value (**Table 3.1**). Canopy cover was estimated using a United States Forest Service percent canopy raster (National Land Cover Database 2011). Elevation was estimated using a United States Geological Survey digital elevation map (DEM; 10 m). Distance to nearest water body was estimated by constructing a raster of the Euclidean distance from all permanent waterbodies. Compound topographic index values were determined using the Geomorphometry and Gradient Metrics (version a1.0) toolbox; this metric is a function of both the slope and the

upstream contributing area per unit width orthogonal to the flow direction (Evans et al. 2010). CTI is a steady state wetness index, where a larger CTI value represents areas that are topographically suitable for water accumulation. We measured solar radiation by calculating the watt-hour/m^2 of the delineated sampling area using the Area Solar Radiation tool in ArcMap (**Table 3.1**). These habitat variables reflect habitat characteristics associated with *F. harpi* (Robison and Crump 2004) and *P. reimeri* (Robison 2008), and other primary burrowing crayfish species (Hobbs 1981, Welch and Eversole 2006, Loughman et al. 2012). These values were calculated using digital elevation maps (National Elevation Dataset http://ned.usgs.gov/ accessed 07/21/2014) and surface water maps (National Hydrography Dataset http://nhd.usgs.gov/index.html accessed 07/21/2014). The entire OME was used as a delineation for both species of crayfish in the SDM analysis. Each surface was resampled to a common resolution of 30 m to match the resolution of the canopy surface.

Maxent analysis

Upon gathering the presence localities and calculating rasters of pertinent habitat variables (canopy cover, CTI, elevation, solar radiation, and distance to nearest waterbody), we created our distribution models, refined our models, tested the models against a null model and generated two final SDMs. The SDM algorithm used was Maxent (version 3.3.3k; Phillips et al. 2006). Along with the presence localities of each species, we incorporated 2500 background points to create our models. These points were randomly generated within a 10 km^2 polygon situated around each historic museum locality sampled in the field. A 10 km^2 buffer was used due to the distance between sites and the relative size of the area in which we projected our Maxent predictions (the OME). This approach followed Peterman et al. (2013) and was

implemented to reduce model bias as described by Phillips (2008). The initial model fit was assessed using the area under the receiver operator curve (AUC).

Upon calculation of the initial Maxent models, a comparison of spatial predictions was conducted with the ENMeval package (Muscarella et al. 2014) in program R (R Development Core Team 2014). ENMeval analysis of *F. harpi* identified a betamultiplier of 2.5 and a linear quadratic hinge feature to provide the most parsimonious fit to our data. ENMeval analysis of *P. reimeri* identified a betamultiplier of 1.5 and a linear quadratic hinge feature to provide the most parsimonious fit to our data. Spatial predictions were then re-run using the refined regularization multiplier and feature classes to increase the rigor in building and evaluating our SDMs based on presence-only data.

The refined models' performance was determined using the null model approach of Raes and ter Steege (2007) using ENMtools (Warren et al. 2010). We generated two groups of 999 random data sets containing 56 and 50 samples, which corresponded to the number of presence locations used for *F. harpi* and *P. reimeri* (respectively) in the initial model. These points were drawn without replacement from the OME delineation used in the initial model. Both model AUC values were compared to the 95 percentile of the null AUC frequency distribution.

The final Maxent models were calculated with the maximum number of iterations set to 5000 and the analysis of variable importance was measured by jackknife and response curves. The bootstrap form of replication was used. These settings, the refined regularization multiplier and feature classes, and the recommended default values were used for our final Maxent model runs. Due to the narrowly endemic nature of both species and the small amount of presence locations in the initial model, we did not include a bias file or spatial filtering.

Field sampling and validation

The refined Maxent models (one for *F. harpi* and one for *P. reimeri*) were used to select 80 semi-random sampling sites for each species within the OME. These sites were semi-random because we restricted our sampling to areas of public access (roadside ditches). The Maxent output for both species was placed into four categories based on the relative occurrence rate (ROR; Fithian and Hastie 2013). The first category ranged from 0 to the lowest presence threshold (LPT) of each species (Pearson et al. 2007). The LPT is the smallest logistic value associated with one of the observed species localities. The second class ranged from the LPT to 50% of the maximum ROR of each species. The third category ranged from 50% of the maximum ROR to 75% of the maximum ROR of each species. The fourth category ranged from 75% of the maximum ROR to the maximum ROR of each species.

The final Maxent model outputs for both species were placed into the described categories in ArcMap. A polygon in ArcMap represented each category. Any polygon representing a single pixel or island (one 30 m x 30 m area) was removed. All category polygons were then overlaid with a layer representing the public right of ways and other public areas (state parks, natural areas, etc.). We generated 40 random points in each category polygon using the final polygon layer. All points within each category polygon had a spatial buffer of 2 km and were checked before sampling to ensure accessibility. If a point was inaccessible in the field, the next closest accessible point within the respected category was chosen and sampled. To assess the accuracy of the Maxent predictions, we calculated the receiver operating characteristic (ROC) and the AUC for the ROR of occupied quadrats vs. the ROR of unoccupied quadrats (Fawcett 2006) with the pROC package in program R (Robin et al. 2011). A ROC graph is a technique for visualizing, organizing and selecting classifiers based on their performance

(Fawcett 2006). A ROC plot displays the performance of a binary classification method (presence/absence) with a continuous (Maxent prediction) ordinal output (Robin et al. 2011). Furthermore, the ROC plot shows the sensitivity (proportion of correctly classified positive observations) and specificity (the proportion of correctly classified negative observations) as the output threshold is moved over the range of all possible values (Robin et al. 2011).

Field sampling occurred in March and April of 2015, the period of peak activity for both *F. harpi* and *P. reimeri* (Robison and Crump 2004). At each sampling point, one 50-m linear transect was searched for the presence of burrows in six 1-m^2 quadrats placed at 10 m intervals along each transect. Within a sampling polygon, the area surrounding the transect was also thoroughly searched for burrows (**Fig. 3.2**). If burrows were present along the transect, quadrat, or within the vicinity of the transect, animals were captured by hand excavation using a hand shovel to slowly dig around the burrow entrance and inserting one's arm into the burrow feeling for the crayfish. This method was chosen over other methods due to the success rate and limited amount of time spent at each burrow location (Ridge et al. 2008). Voucher specimens of crayfishes collected were deposited into the Illinois Natural History Survey Crustacean Collection.

RESULTS

Presence data

The presence locations used for the Maxent analysis, based on the field surveys of 2014, consisted of 58 locations for *F. harpi* (of which 56 were used for the SDM analysis) and 53 locations for *P. reimeri* (of which 50 were used for the SDM analysis). To minimize spatial autocorrelation, a subset of the original presence data was used. All duplicate presence locations

falling within the same cell of a 30 m resolution raster were removed before the SDM analysis. The selected presence locations used for the SDM analysis were near (<90 m) primary and secondary roadways.

Maxent analysis

The AUC converged to 0.959 and 0.976 for the final *F. harpi* and *P. reimeri* models, respectively. Both models were significantly better than the random AUC estimations from the null models ($p<0.001$). Of the parameters included in the model, canopy cover was the variable with the highest percent contribution for both species (48.8% and 47.2% *F. harpi* and *P. reimeri*, respectively; **Table 3.2**). Both species showed a steady decline in the probability of presence as canopy cover increased. The variable with the highest gain when used in isolation was elevation for both species (**Table 3.2)**. An elevation between 150 m and 200 m was most suitable for *F. harpi* and between 300 m and 350 m was most suitable for *P. reimeri*. The concentration of the highest ROR was centered around the presence locations for both species (**Fig. 3.3)**. The LPT was 0.07 for *F. harpi* and 0.26 for *P. reimeri*. In the *F. harpi* model, 10% of the area in the OME was predicted to be above the LPT. In the *P. reimeri* model, 2% of the OME was predicted to be above the LPT (**Table 3.3)**.

Field sampling and validation

Most (89% for *F. harpi* and 98% for *P. reimeri*) of the land area in the OME was in the first (lowest ROR) category (**Table 3.3)**. No individuals of either species were caught in areas predicted below the LPT (category 1). Most (74%) of the presence locations for *F. harpi* were in category 4 (**Table 3.3**). The presence locations for *P. reimeri* were more evenly distributed between categories 2, 3, and 4 (**Table 3.3**). *Fallicambarus harpi* was captured in 19 of the 480

quadrats surveyed for the species (**Fig. 3.4**). *Procambarus reimeri* was captured in 41 of the 480 quadrats surveyed for the species (**Fig. 3.4**). We counted 70 burrows for each *F. harpi* and *P. reimeri*. The historic range of *F. harpi* was extended by 2.8 km to the north and 3.2 km to the south of its historic range while the historic range of *P. reimeri* was extended by 51.6 km to the east, 12.1 km to the south, and 19.2 km to the west of its historic range. Thus, the total range for both species was approximately 265 km^2 for *F. harpi* and 1467 km^2 for *P. reimeri* using a minimum convex polygon approach in ArcGIS encompassing all known capture localities from both years and historic museum data.

The AUC for the *F. harpi* field validation was 80.96 (73.94-87.98). The AUC for the *P. reimeri* field validation was 70.5 (63.33 – 77.67). The ROC plot, AUC, and 95% confidence intervals for each model are shown in **Figure 3.4**. The threshold values (prediction with the highest specificity and sensitivity) were 0.68 and 0.57 for *F. harpi* and *P. reimeri*, respectively.

DISCUSSION

We found SDMs to be a useful tool to predict the occurrence and distribution of suitable habitat of two narrowly endemic, burrowing crayfish species in Arkansas. We used Maxent along with a suite of functions to assess model fit and safeguard against potential pitfalls associated with the Maxent program (Phillips and Dudík 2008, Elith et al. 2010, Warren and Seifert 2011, Hijmans 2012, Lahoz-Monfort et al. 2014). We also used biologically relative habitat information at a constrained geographic scale to increase the accuracy of our predictions (Guisan and Thuiller 2005). These habitat variables and the scale at which we delineated them were a result of previous field sampling and analysis of habitat preference of both species (Rhoden et al. *in press*), which revealed both crayfish to be microhabitat specialists; using open, low-herbaceous microhabitats. We validated the model through a stratified sampling of our

Maxent model predictions based on the LPT and the maximum ROR. We then equally sampled each category across the entire OME. This validation resulted in the range expansion of both species and the discovery of new populations. The models performed well by directing sampling efforts to treeless areas on the landscape that tended to have greater predicted probabilities of occurrence. However, the models did a poor job of identifying the wet, low-herbaceous microhabitats most frequently associated with occurrence in the field and previous studies (Robison and Crump 2004, Robison 2008, Rhoden et al. *in press*).

The use of the LPT to determine the threshold between the probability of presence or absence at any given predicted output location (Pearson et al. 2007) is well documented (Rinnhofer et al. 2012, Boria et al. 2014, Fois et al. 2015). We successfully used this value in our field validation techniques: no animal was captured in an area predicted below the LPT (**Table 3.3**). The land area above the LPT for the *F. harpi* model comprised 10% of the Ouachita Mountain Ecoregion (OME) and 2% for the *P. reimeri* model in Arkansas. The ROC analysis identified threshold values of 0.68 and 0.57 for the *F. harpi* and *P. reimeri* models, respectively, which optimized the sensitivity and specificity of our model (Robin et al. 2011). These values are far more conservative than the LPT and are based on the field validation results from both species. Using the threshold metrics, the area predicted as suitable habitat for *F. harpi* and *P. reimeri* is less than 1% of the OME. We recommend the use of this threshold based on the ROC analysis for a more fine-tuned sampling effort for high-quality habitat for both species in the future.

Our SDMs used fine-scale (30 m) rasters of relative biological variables (canopy cover, CTI, solar radiation, elevation, and distance to waterbody). In the past, it has been common to use coarse (≥1 km) climatic data to construct models (e.g. Peterson 2001, Chunco et al. 2013,

Barnhart and Gillam 2014). The use of coarse-scale habitat variables in Maxent has been addressed in previous studies (Araújo and Guisan 2006, Jiménez-Alfaro et al. 2012). Others using fine-scale inputs have found new populations of other rare species such as the discovery of new breeding ponds for a salamander species in east central Illinois (*Ambystoma jeffersonianum*; Peterman et al. 2013). Using fine-scale rasters of specific habitat variables for narrowly endemic habitat specialists was more appropriate than the more general approach of coarse-scale climatic data due to the resolution one gains with specific habitat information and fine-scale inputs. We believe this resolution was necessary to capture elements of the microhabitat the crayfishes prefer, differentiating between suitable and unsuitable habitat at a scale to assess animals occurring within anthropogenically altered habitat situated in natural landscapes (roadside ditches).

The habitat attributes of sites in which animals were present consisted of treeless, wet, low-herbaceous microhabitats. The average canopy cover for the categories above the LPT (category 2, 3, and 4) was 17% for both species. The presence quadrats for both species had an average canopy cover of 5%. Hydrophilic sedges were present in over 90% of the quadrats having *F. harpi* and *P. reimeri*, however sedges were present in less than half of the quadrats predicted above the LPT (category 2, 3, and 4). The sites recorded as being above the LPT (categories 2, 3, and 4) not having the target species were treeless for the most part, however those sites did not exhibit a moist microhabitat. The Maxent models did not capture the perched water table observed across the landscape that has been associated with other primary burrowing crayfishes (Welch et al. 2007). It is likely the model did not capture these moist, low herbaceous habitats due to the variables chosen for the Maxent analysis (canopy cover, CTI, elevation, solar

radiation, and distance to nearest waterbody). Future studies could incorporate remotely sensed data to better identify these unique habitats.

Our study shows Maxent is an appropriate tool to analyze and discover populations of narrowly endemic species in the Ouachita Mountains of Arkansas. Our method of initially collecting habitat data using museum records in the spring of 2014 added precision to our presence locations used for analysis. Our initial surveys also added valuable information regarding the habitat preferences of both *F. harpi* and *P. reimeri*, which in turn guided the selection of our habitat variables for both models. Our concentrated search efforts resulted in the discovery of five new populations of *F. harpi* and 16 new populations of *P. reimeri* and range expansions of approximately 91 km^2 and 1404 km^2, respectively. The discovery of new populations support the contention both species appear to be locally abundant where habitat is suitable for persistence will aid in the conservation of these rare species. This is accomplished by narrowing the knowledge gap in distribution information, adding localities for monitoring persistence in roadside ditches, and providing habitat preference information. All of these attributes are required for the refinement of conservation and management decisions. Constructing models followed by ground validation has added valuable habitat information to two spatially restricted, understudied species of primary burrowing crayfish in southwestern Arkansas and illustrates the effectiveness of such a strategy for other rare habitat specialists.

TABLES AND FIGURES

Table 3.1: Description, origin, resolution, general statistics, and units of environmental variables used in the Maxent analysis of two primary burrowing crayfish species (*Fallicambarus harpi* and *Procambarus reimeri*) in southwestern Arkansas.

Variable	Description	Source	Resolution	Min/max(unit)	μ (sd)
**Canopy cover**	Percent tree canopy cover	National Land Cover Database 2011 USFS	30 m	0/100 (% cover)	52.23(43.48)
**Elevation**	Digital elevation model of the study site	USGS National Elevation Dataset	10 m	50.50/818.96 (m)	229(100.85)
**Distance to nearest waterbody**	Euclidean distance to nearest permanent waterbody across the study site	ESRI Spatial Analyst Tools; National Hydrology Dataset composed of stream segments of study site	10 m	0/1740.26 (m)	187.48(161.04)
**Compound topographic index**	A function of slope and the upstream contributing area per unit width orthogonal to the flow direction (Evans et al., 2010)	ArcGIS Geomorphometry and Gradient Metrics Toolbox 2.0 (Evans et. al., 2010); National Elevation Dataset	10 m	2.67/27.58 (index value)	7.58(1.94)
**Solar radiation**	Incoming solar radiation value (watt hours per m^2) based on direct and diffuse insolation from the unobstructed sky directions	ESRI Spatial Analyst Tools; National Elevation Dataset	10 m	3542.41/6413.34 (watthours/m^2)	5955.14(116.01)

Table 3.2: Percent contribution and permutation importance of each environmental variable analyzed in the final Maxent models for two primary burrowing crayfish species (*Fallicambarus harpi* and *Procambarus reimeri*) in southwestern Arkansas.

Fallicambarus harpi		
**Variable**	**Percent contribution**	**Permutation importance**
**Canopy**	48.8	15.7
**Elevation**	37.9	56.3
**CTI**	7.6	2
**Solar**	4	25.4
**Distance to nearest waterbody**	1.8	0.6
Procambarus reimeri		
**Variable**	**Percent contribution**	**Permutation importance**
**Canopy**	47.2	27.5
**Elevation**	39.8	41.9
**Distance to nearest waterbody**	7.1	16.8
**CTI**	5.5	9
**Solar**	0.4	4.9

Table 3.3: (A) Threshold values (relative occurrence rate; ROR), land area (ha), and percentage of Ouachita Mountains Ecoregion (OME) of each relative occurrence category; and (B) number of presence and absence quadrats, average canopy cover (%) of quadrats sampled in each relative occurrence category, and percentage of quadrats in each relative occurrence category with sedges present from the field sampling based on Maxent models for two primary burrowing crayfish species (*Fallicambarus harpi* and *Procambarus reimeri*) in southwestern Arkansas.

A.

**Species**	**Category 1**	**Category 2**	**Category 3**	**Category 4**
		**Thresholds (ROR)**		
Fallicambarus harpi	0.00 – 0.07	0.07 – 0.44	0.44 – 0.66	0.66 – 0.88
Procambarus reimeri	0.00 – 0.26	0.26 – 0.42	0.42 – 0.64	0.64 – 0.85
		**Land area (ha)**		
Fallicambarus harpi	1374105	143139	21894	4996
Procambarus reimeri	1515441	15209	9756	3728
		**Percentage of OME**		
Fallicambarus harpi	89	9	1	<1
Procambarus reimeri	98	1	1	<1

B.

**Variable**	**Category 1**	**Category 2**	**Category 3**	**Category 4**
		Fallicambarus harpi		
**Present**	0	0	5	14
**Absent**	121	120	115	105
**Average Canopy Cover**	34	26	16	7
**Percent Quad w/sedge**	27	73	44	46
		Procambarus reimeri		
**Present**	0	12	14	15
**Absent**	122	106	106	105
**Average Canopy Cover**	38	18	19	15
**Percent Quad w/sedge**	51	55	63	45

Figure 3.1: Map depicting the location of sites sampled in southwestern Arkansas in the spring of 2015 based on the predictions from a Maxent analysis of two primary burrowing crayfish species (*Fallicambarus harpi* and *Procambarus reimeri*).

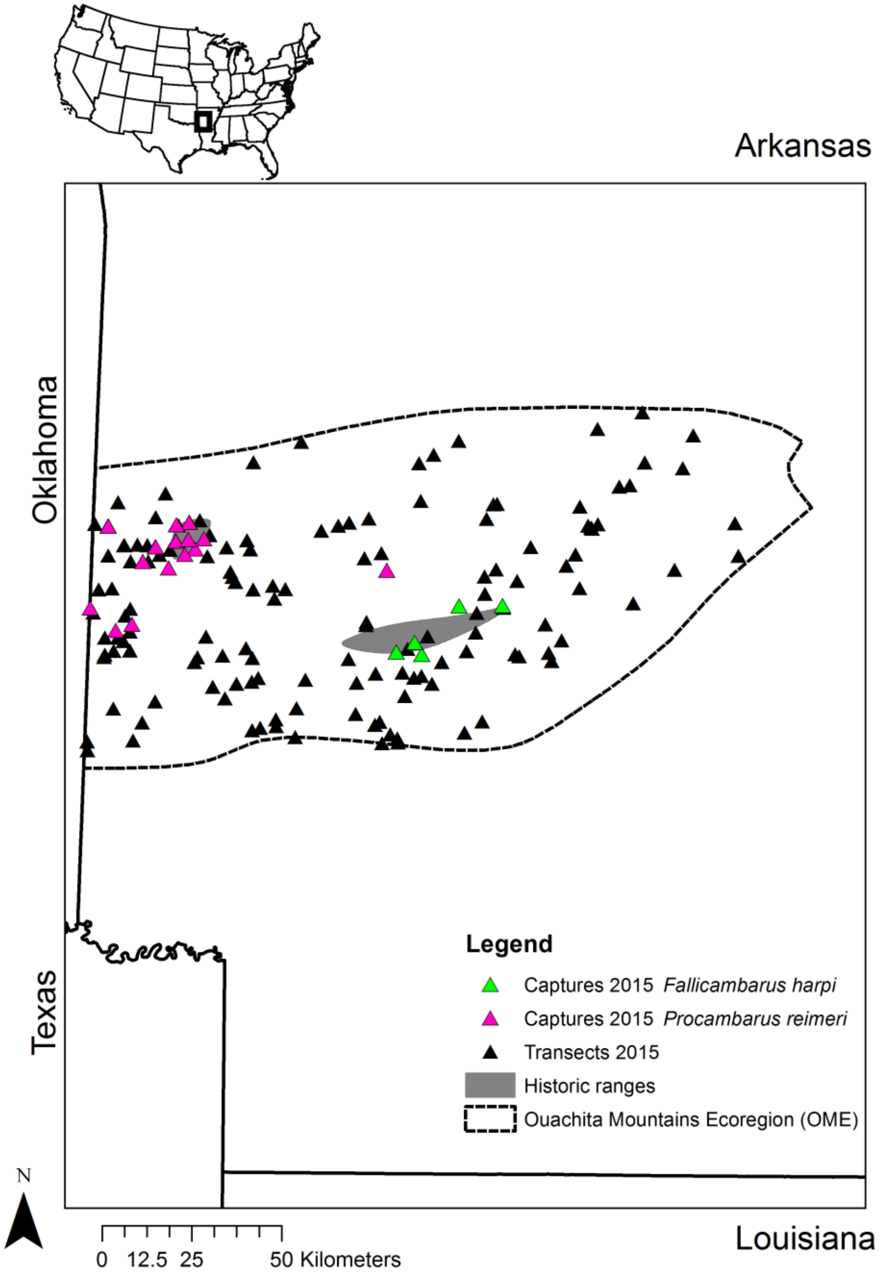

Figure 3.2: Map representing the sampling scheme based on the predictions from a Maxent analysis of two primary burrowing crayfish species (*Fallicambarus harpi* and *Procambarus reimeri*) in southwestern Arkansas in the spring of 2015. Each color (green, yellow, orange, and red) represents a relative occurrence category (1, 2, 3, 4) upon which the field validation sampling procedure was based. The black lines in the lower graphic depict 50-m transects used to assess presence or absence of the target species at each site. The linear, focused colors in the bottom graphic represent the accessible polygons in which the transect sampling was carried out.

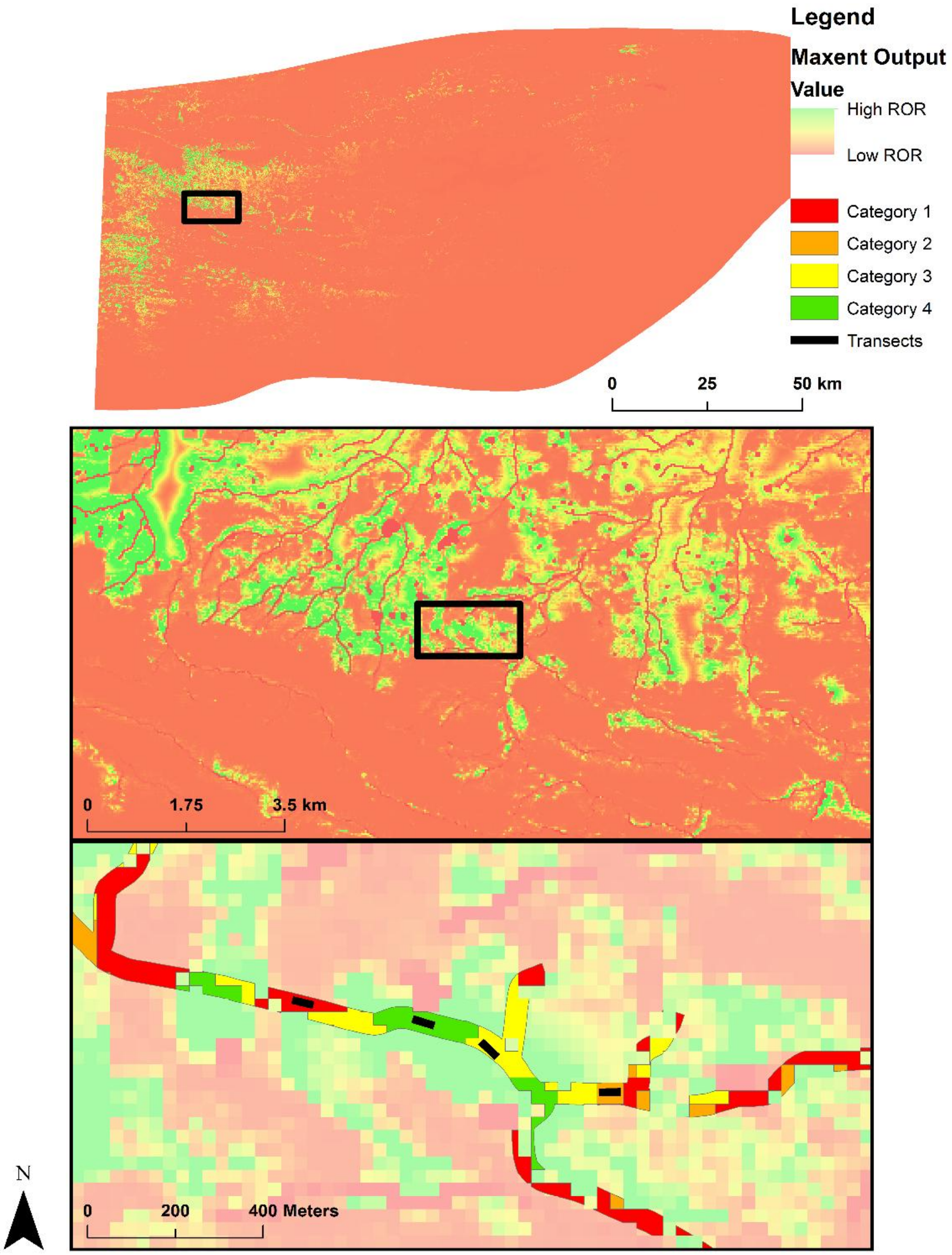

Figure 3.3: Projection of the Maxent models for (A) *Procambarus reimeri* and (B) *Fallicambarus harpi* onto the environmental variables (Table 3.1) used for analysis in southwestern Arkansas. The total shaded area represents the Ouachita Mountains Ecoregion (OME). Cooler colors show areas with better predicted conditions (relative occurrence rates [ROR]).

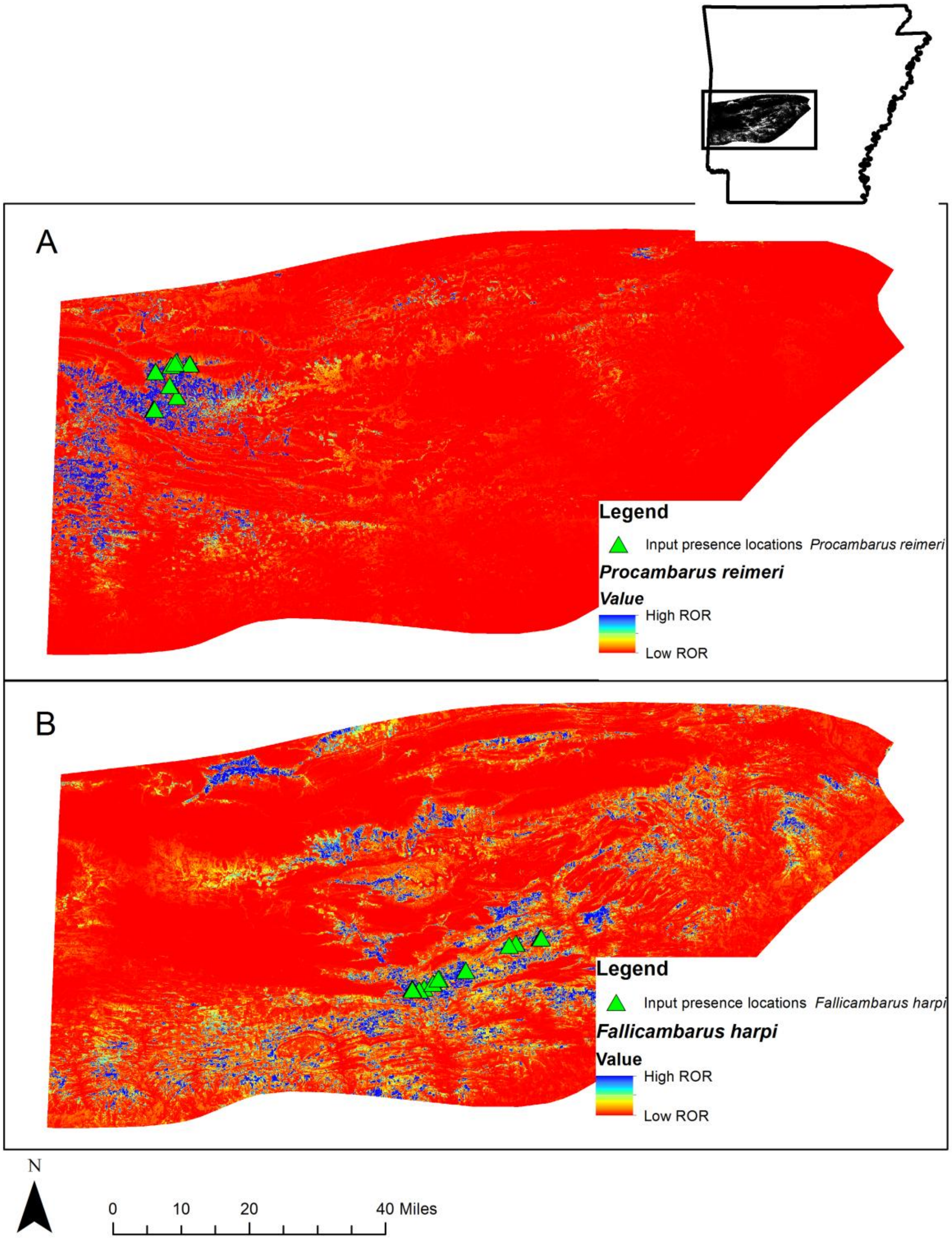

Figure 3.4: ROC analysis for two primary burrowing crayfish species, *Fallicambarus harpi* (A) and *Procambarus reimeri* (B), in southwestern Arkansas. Input data derived from the predictions of two Maxent models and presence/absence data from field surveys conducted in the spring of 2015. The cross represents the 95% confidence interval and shaded bars represent the associated error for each ROC curve. AUC values are given with 95% confidence intervals in parenthesis. A higher AUC value depicts a better classification. Inset bar chart represents the percentage of quadrats (y-axis) in each category 1, 2, 3, and 4 (x-axis) that harbored each species of burrowing crayfish.

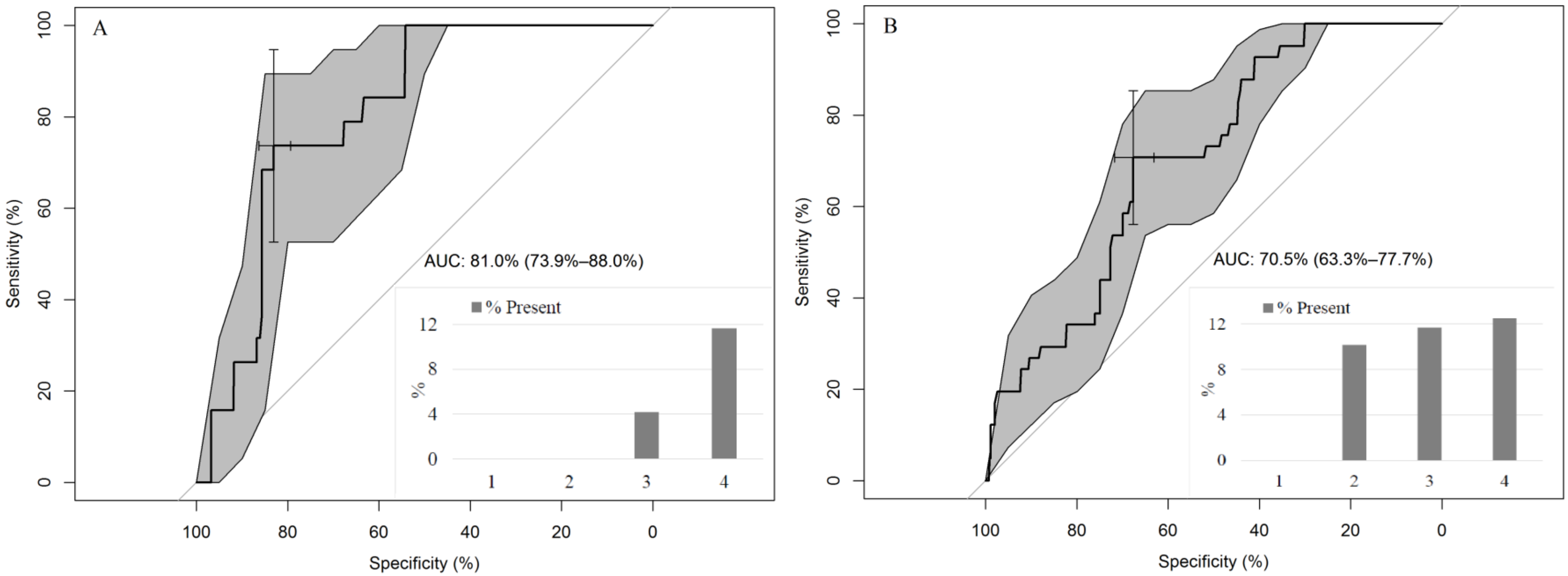

LITERATURE CITED

Araújo, M. B., and A. Guisan. 2006. Five (or so) challenges for species distribution modelling. *Journal of Biogeography* 33:1677–1688.

Araújo, M. B., and A. T. Peterson. 2012. Uses and misuses of bioclimatic envelope modeling. Ecology 93(7):1527–1539.

Baldwin, R. A. 2009. Use of maximum entropy modeling in wildlife research. Entropy 11:854–866.

Barnhart, P. R., and E. H. Gillam. 2014. The Impact of Sampling Method on Maximum Entropy Species Distribution Modeling for Bats. Acta Chiropterologica 16:241–248.

Berrill M. and B. Chenoweth. 1982. The burrowing ability of nonburrowing crayfish. American Midland Naturalist 108(1):199-201.

Boria, R. A., L. E. Olson, S. M. Goodman, and R. P. Anderson. 2014. Spatial filtering to reduce sampling bias can improve the performance of ecological niche models. Ecological Modelling 275:73–77.

Chunco, A. J., S. Phimmachak, N. Sivongxay, and B. L. Stuart. 2013. Predicting Environmental Suitability for a Rare and Threatened Species (Lao Newt, *Laotriton laoensis*) using Validated Species Distribution Models. PLoS ONE 8.

Elith, J., C. H. Graham, R. P. Anderson, M. Dudık, S. Ferrier, A. Guisan, R. J. Hijmans, F. Huettmann, J. R. Leathwick, A. Lehmann, J. Li, L. G. Lohmann, B. A. Loiselle, G. Manion, C. Moritz, M. Nakamura, Y. Nakazawa, J. M. Overton, A. T. Peterson, S. J. Phillips, K. Richardson, R. E. Scachetti-Pereira, Ricardo S, Schapire, J. Soberon, S. Williams, M. S. Wisz, and N. E. Zimmermann. 2006. Novel methods improve prediction of species' distributions from occurrence data. Ecography 29:129–151.

Elith, J., and C. H. Graham. 2009. Do they? How do they? Why do they differ? On finding reasons for differing performances of species distribution models. Ecography 32:66–77.

Elith, J., M. Kearney, and S. Phillips. 2010. The art of modelling range-shifting species. Methods in Ecology and Evolution 1:330–342.

Elith, J., S. J. Phillips, T. Hastie, M. Dudík, Y. E. Chee, and C. J. Yates. 2011. A statistical explanation of MaxEnt for ecologists. Diversity and Distributions 17:43–57.

Evans, J., J. Oakleaf, S. Cushman, and D. Theobald. 2010. An ArcGIS toolbox for surface gradient and geomorphic modeling, version a1.0. (Available from: http://evansmurphy.wix.com/evansspatial#!arcgis-gradient-metrics-toolbox/crro)

Fawcett, T. 2006. An introduction to ROC analysis. Pattern Recognition Letters 27:861–874.

Fithian, W., and T. Hastie. 2013. Finite-sample equivalence in statistical models for presence-absence only data. Annals of Applied Statistics 7:1917–1939.

Fois, M., G. Fenu, A. Cuena Lombraña, D. Cogoni, and G. Bacchetta. 2015. A practical method to speed up the discovery of unknown populations using Species Distribution Models. *Journal for Nature Conservation* 24:42–48.

Franklin, J. 2013. Species distribution models in conservation biogeography: developments and challenges. Diversity and Distributions 19:1217–1223.

Greaves, G. J., R. Mathieu, and P. J. Seddon. 2006. Predictive modelling and ground validation of the spatial distribution of the New Zealand long-tailed bat (*Chalinolobus tuberculatus*). Biological Conservation 132:211–221.

Guillera-Arroita, G., J. J. Lahoz-Monfort, and J. Elith. 2014. Maxent is not a presence-absence method: a comment on Thibaud et al. R. B. O’Hara, editor. Methods in Ecology and Evolution 5:1192–1197.

Guisan, A., and W. Thuiller. 2005. Predicting species distribution: Offering more than simple habitat models. Ecology Letters 8:993–1009.

Hernandez, P. A., C. H. Graham, L. L. Master, and D. L. Albert. 2006. The effect of sample size and species characteristics on performance of different species distribution modeling methods. Ecography 29:773–785.

Hijmans, R. J. 2012. Cross-validation of species distribution models: Removing spatial sorting bias and calibration with a null model. Ecology 93:679–688.

Hlass, L. J., W. L. Fisher, and D. J. Turton. 1998. Use of the Index of Biotic Integrity to Assess Water Quality in Forested Streams of the Ouachita Mountains Ecoregion, Arkansas. *Journal of Freshwater Ecology* 13:181–192.

Hobbs, H. H., Jr. 1979. A new crayfish from the Ouachita River basin in Arkansas (Decapoda: Cambaridae). Proceedings of the Biological Society of Washington 92:804–811.

Hobbs, H. H., Jr. 1981. The Crayfishes of Georgia. Smithsonian Contributions to Zoology 318. Smithsonian Institution Press, Washington, D. C.

Hobbs, H. H., Jr. and H. W. Robison. 1985. A new burrowing crayfish (Decapoda: Cambaridae) from southwestern Arkansas. Proceedings of the Biological Society of Washington 98(4):1035-1041.

Jiménez-Alfaro, B., D. Draper, and D. Nogués-Bravo. 2012. Modeling the potential area of occupancy at fine resolution may reduce uncertainty in species range estimates. Biological Conservation 147:190–196.

Kramer-Schadt, S., J. Niedballa, J. D. Pilgrim, B. Schröder, J. Lindenborn, V. Reinfelder, M. Stillfried, I. Heckmann, A. K. Scharf, D. M. Augeri, S. M. Cheyne, A. J. Hearn, J. Ross, D. W. Macdonald, J. Mathai, J. Eaton, A. J. Marshall, G. Semiadi, R. Rustam, H.

Bernard, R. Alfred, H. Samejima, J. W. Duckworth, C. Breitenmoser-Wuersten, J. L. Belant, H. Hofer, and A. Wilting. 2013. The importance of correcting for sampling bias in MaxEnt species distribution models. Diversity and Distributions 19:1366–1379.

Lahoz-Monfort, J. J., G. Guillera-Arroita, and B. A. Wintle. 2014. Imperfect detection impacts the performance of species distribution models. Global Ecology and Biogeography 23:504–515.

Larson, E. R., and J. D. Olden. 2010. Latent extinction and invasion risk of crayfishes in the southeastern United States. Conservation Biology 24:1099–1110.

Latif, Q. S., V. A. Saab, K. Mellen-Mclean, and J. G. Dudley. 2015. Evaluating habitat suitability models for nesting white-headed woodpeckers in unburned forest. *The Journal of Wildlife Management* 79:263–273.

Loughman, Z. J., T. P. Simon, and S. A. Welsh. 2012. Occupancy rates of primary burrowing crayfish in natural and disturbed large river bottomlands. *Journal of Crustacean Biology* 32:557–564.

Moore, M. J., R. J. DiStefano, and E. R. Larson. 2013. An assessment of life-history studies for USA and Canadian crayfishes: knowledge gaps to improve conservation and management. Freshwater Science 32(3): 1276-1287.

Morehouse, R. L., and M. Tobler. 2013. Crayfishes (Decapoda: Cambaridae) of Oklahoma: identification, distributions, and natural history. Zootaxa 3717:101–157.

Muscarella, R., P. J. Galante, M. Soley-Guardia, R. A. Boria, J. M. Kass, M. Uriarte, and R. P. Anderson. 2014. ENMeval: An R package for conducting spatially independent evaluations and estimating optimal model complexity for Maxent ecological niche models. Methods in Ecology and Evolution 5:1198–1205.

Page, L. 1985. The crayfishes and shrimps (Decapoda) of Illinois. Illinois Natural History Survey Bulletin 33(4):i-339.

Pearson, R. G., C. J. Raxworthy, M. Nakamura, and A. Townsend Peterson. 2007. Predicting species distributions from small numbers of occurrence records: a test case using cryptic geckos in Madagascar. *Journal of Biogeography* 34:102–117.

Peterman, W. E., J. A. Crawford, and A. R. Kuhns. 2013. Using species distribution and occupancy modeling to guide survey efforts and assess species status. *Journal for Nature Conservation* 21:114–121.

Peterson, A. T. 2001. Predicting Species' Geographic Distributions Based on Ecological Niche Modeling. The Condor 103(3):599–605.

Phillips, J. D., and D. A. Marion. 2005. Biomechanical effects, lithological variations, and local pedodiversity in some forest soils of Arkansas. Geoderma 124:73–89.

Phillips, S. J., R. P. Anderson, and R. E. Schapire. 2006. Maximum entropy modeling of species geographic distributions. Ecological Modelling 190:231–259.

Phillips, S. J., and M. Dudík. 2008. Modeling of species distributions with Maxent: new extensions and a comprehensive evaluation. Ecography 31:161–175.

Phillips, S. J. 2008. Transferability, sample selection bias and background data in presence-only modelling: A response to Peterson et al. (2007). Ecography 31:272–278.

R Development Core Team. 2014. R: A Language and Environment for Statistical Computing. R Foundation for Statistical Computing, Vienna, Austria. (http://www.r-project.org).

Raes, N. and H. ter Steege. 2007. A null-model for significance testing of presence-only species distribution models. Ecography 30: 727-736.

Rebelo, H., and G. Jones. 2010. Ground validation of presence-only modelling with rare species: A case study on barbastelles *Barbastella barbastellus* (Chiroptera: Vespertilionidae). *Journal of Applied Ecology* 47:410–420.

Rhoden, C. M., C. A. Taylor, and W. E. Peterman. 2016. Highway to heaven? Roadsides as

preferred habitat for two narrowly endemic crayfish. Freshwater Science 35:(in press).

Ridge, J., T. P. Simon, D. Karns, and J. Robb. 2008. Comparison of Three Burrowing Crayfish Capture Methods Based on Relationships with Species Morphology, Seasonality, and Habitat Quality. *Journal of Crustacean Biology* 28:466–472.

Rinnhofer, L. J., N. Roura-Pascual, W. Arthofer, T. Dejaco, B. Thaler-Knoflach, G. A. Wachter, E. Christian, F. M. Steiner, and B. C. Schlick-Steiner. 2012. Iterative species distribution modelling and ground validation in endemism research: an Alpine jumping bristletail example. Biodiversity and Conservation 21:2845–2863.

Robin, X., N. Turck, A. Hainard, N. Tiberti, F. Lisacek, J.-C. Sanchez, and M. Müller. 2011. pROC: an open-source package for R and S+ to analyze and compare ROC curves. BMC bioinformatics 12:77.

Robison, H. W., and B. Crump. 2004. Distribution, natural history aspects, and status of the Arkansas endemic crayfish, *Fallicambarus harpi* Hobbs and Robison, 1985. *Journal of the Arkansas Academy of Science* 58:91–94.

Robison, H. W. 2008. Distribution, life history aspects, and conservation status of three Ouachita Mountain crayfishes: *Procambarus tenuis, P. reimeri,* and *Orconectes menae*. U.S.D.A. Forest Service, Ouachita National Forest. Hot Springs, Arkansas.

Robison, H. W., C. McAllister, C. Carlton, and G. Tucker. 2008. The Arkansas endemic biota: An update with additions and deletions. *Journal of the Arkansas Academy of Science* 62: 84-96.

Searcy, C. A., and H. B. Shaffer. 2014. Field validation supports novel niche modeling strategies in a cryptic endangered amphibian. Ecography 37:983-992.

Simmons, J. W., and S. J. Fraley. 2010. Distribution, Status, and Life-History Observations of Crayfishes in Western North Carolina. Southeastern Naturalist 9:79–126.

Taylor, C. A., G. A. Schuster, J. E. Cooper, R. J. DiStefano, A. G. Eversole, P. Hamr, H. H. Hobbs III, H. W. Robison, C. E. Skelton, and R. F. Thoma. 2007. A reassessment of the conservation status of crayfishes of the United States and Canada after 10+ years of increased awareness. Fisheries 32:372–389.

Warren, D. L., R. E. Glor, and M. Turelli. 2010. ENMTools: A toolbox for comparative studies of environmental niche models. Ecography 33:607–611.

Warren, D. L., and S. N. Seifert. 2011. Ecological niche modeling in Maxent: the importance of model complexity and the performance of model selection criteria. Ecological Applications. 21(2):335-342.

Welch, S. M., A. G. Eversole, and J. Riley. 2007. Using the spatial information implicit in the habitat specificity of the burrowing crayfish *Distocambarus crockeri* to identify a lost landscape component. Ecography 30:349–358.

Welch, S. M., and A. G. Eversole. 2006. The occurrence of primary burrowing crayfish in terrestrial habitat. Biological Conservation 130:458–464.

Williams, J. N., C. Seo, J. Thorne, J. K. Nelson, S. Erwin, J. M. O'Brien, and M. W. Schwartz. 2009. Using species distribution models to predict new occurrences for rare plants. Diversity and Distributions 15:565–576.

Williams, J. D., M. L. Warren Jr., K. S. Cummings, J. L. Harris, and R. J. Neves. 1993. Conservation status of freshwater mussels of the United States and Canada. Fisheries 18(9):6-22.

Wisz, M. S., R. J. Hijmans, J. Li, A. T. Peterson, C. H. Graham, and A. Guisan. 2008. Effects of sample size on the performance of species distribution models. Diversity and Distributions 14:763–773.

Woods, A. J., T. L. Foti, S. S. Chapman, J. M. Omernik, J. A. Wise, E. O. Murray, W. L. Prior, J. B. J. Pagan, J. A. Comstock, and M. Radford. 2004. Ecoregions of Arkansas (color poster with map, descriptive text, summary tables, and photographs). US Geological Survey map scale 1:1,000,000. US Geological Survey, Reston, Virginia. (Available from: http://archive.epa.gov/wed/ecoregions/web/html/ar_eco.html)

Yackulic, C. B., R. Chandler, E. F. Zipkin, J. A. Royle, J. D. Nichols, E. H. Campbell Grant, and S. Veran. 2013. Presence-only modelling using MAXENT: when can we trust the inferences? Methods in Ecology and Evolution 4:236–243.

CHAPTER 4: HABITAT ASSESSMENT AND RANGE UPDATES FOR TWO RARE ARKANSAS BURROWING CRAYFISHES: *FALLICAMBARUS HARPI* AND *PROCAMBARUS REIMERI*

INTRODUCTION

North America has the highest diversity of crayfishes worldwide (Taylor et al., 2007). Within North America, 22% of the species listed as endangered or threatened in a recent conservation review (Taylor et al., 2007) were primary burrowing crayfishes. Primary burrowing crayfishes differ from stream-dwelling crayfishes in their life history traits. They spend their complete life cycle underground, leaving their burrows only to forage and search for a burrow of the opposite sex for mating (Hobbs 1981). The Ouachita Highlands Freshwater Ecoregion is the 6th most diverse freshwater ecoregion for native crayfishes in the United States and Canada (Moore et al. 2013). Within the Ouachita Highlands Freshwater Ecoregion, the Ouachita Mountains Ecoregion (Woods et al. 2004; OME) of southwestern Arkansas harbors the highest diversity of primary burrowing crayfishes in the state with six species (*Fallicambarus harpi, F. jeanae, F. strawni, Procambarus liberorum, P. parasimulans,* and *P. reimeri*).

The narrowly endemic nature of North American crayfishes is well documented (Morehouse and Tobler, 2013; Page, 1985; Simmons and Fraley, 2010; Taylor et al., 2007), and primary burrowing crayfishes in Arkansas are no exception (Robison et al., 2008). The endemic nature of any taxa makes them more vulnerable to localized extirpation. Because these animals occur at such a constrained geographic scale, it is important to accurately describe their habitat preferences and range to ensure the persistence of local populations through the management of suitable habitat and monitoring of existing populations. Two such primary burrowing crayfishes in Arkansas in need of these conservation assessments are *Fallicambarus harpi* (Ouachita Burrowing Crayfish) and *Procambarus reimeri* (Irons Fork Burrowing Crayfish).

Since their formal descriptions, little information has been published regarding the habitat preferences and range assessments of *F. harpi* and *P. reimeri*. Both crayfishes are endemic to the OME in southwestern Arkansas. These species are vulnerable to population declines and are currently recorded under the Endangered (*P. reimeri*) and Vulnerable (*F. harpi*) conservation status categories based on modifications to or reductions in habitat in their already restricted ranges (Taylor et al. 2007). Also, these species were included in a recent petition filed by the privately funded Center for Biological Diversity for protection under the Federal Endangered Species Act. To assess these conservation concerns, we developed a study with the following objectives: 1) to determine the holistic range of both species (*F. harpi* and *P. reimeri*) within the OME of Arkansas and 2) to refine the description of suitable habitat for both *F. harpi* and *P. reimeri*.

TARGET SPECIES ACCOUNTS

Fallicambarus (*Fallicambarus*) *harpi*- The Ouachita Burrowing Crayfish was described from two locations in Pike County, Arkansas, in 1985 by H.H. Hobbs, Jr. and H.W. Robison (Hobbs and Robison 1985). Robison and Crump (2004) reviewed and updated the status of this endemic crayfish, revealing 12 new populations in Montgomery, Hot Spring, Garland, and Pike counties in Arkansas. Since 2004, no other information has been published relating to the range or habitat requirements of this species. This primary burrowing crayfish is known from wet seepage areas with abundant sedges such as roadside ditches and other right of ways (Robison and Crump 2004). *Fallicambarus harpi* most closely resembles both *F. strawni* and *F. jeanae* but differs by possessing a free, never adnate, cephalic process on the first pleopod of the first form male (Hobbs and Robison 1985).

Procambarus (*Girardiella*) *reimeri*- The Irons Fork Burrowing Crayfish was described from six locations in Polk County, Arkansas, by H.H. Hobbs, Jr. in 1979 (Hobbs 1979). Robison (2008) most recently reviewed and updated the status of this Arkansas endemic, revealing the species only to occur in Polk County, in the vicinity of Mena, Arkansas. Since 2008, no other information has been published relating to the range or habitat requirements of this species. This primary burrowing crayfish reportedly constructs relatively simple burrows in sandy clay soil in wet seepage areas and roadside ditches (Hobbs 1979, Robison 2008). This species most closely resembles *P. gracilis* and *P. liberorum* but differs from both by possessing a broader areola and lacking tubercles on the annulus ventralis (Hobbs 1979).

METHODS

Field surveys were conducted in the spring of 2014 and 2015 for these two primary burrowing crayfishes in southwestern Arkansas. We sampled in the spring of both years as this is the time of peak activity for both species, which correlates with the time of highest species detection. In 2014, we sampled historic localities from museum databases. Historical records for both species were queried from the Illinois Natural History Survey Crustacean Collection, National Museum of Natural History Invertebrate Zoology Collection, and Arkansas Department of Natural Heritage. In 2015, we based our sampling sites on species distribution models created from the previous year's capture records. We sampled 13 counties in western Arkansas encompassing the known range of both species of primary burrowing crayfish: Pulaski, Saline, Perry, Garland, Hot Spring, Clark, Yell, Montgomery, Pike, Scott, Howard, Polk, and Sevier (listed from east to west).

In 2014, three to six 50-m transects were positioned at and near the historical museum locality. The first transect at each sampling site was placed where burrows were present,

ensuring the first transect was indeed situated over the historical museum location. After a GPS location and azimuth were taken at the 0-m mark, a 1-m^2 PVC quadrat was placed over the linear transect every 10 m resulting in six 1-m^2 quadrats per 50-m transect. After the initial transect was completed over the historical location, two to five transects were completed in the same way in adjacent habitat at the sampling site. Habitat heterogeneity determined the number of transects sampled at each site. If a site was homogenous, fewer transects were sampled to increase the number of sampling sites to be visited during our sampling window.

In 2015, we sampled a semi-random group of sites based on two species distribution models, one for *F. harpi* and one for *P. reimeri*. These species distribution models were constructed with Maxent (Phillips et al. 2006). Maxent is a presence-only modeling algorithm using a set of known occurrences together with predictor variables such as topographic, climatic, edaphic, biogeographic, and remotely sensed variables (Phillips et al. 2006, Phillips and Dudík 2008). These data are used to predict the relative occurrence rate of a focal species across a predefined landscape (Fithian and Hastie 2013). At each sampling site, one 50-m transect was positioned at a GPS location selected *a priori* from the output of the Maxent model. After a GPS location and azimuth were taken at the 0-m mark, a 1-m^2 PVC quadrat was placed over the linear transect every 10 m resulting in six 1-m^2 quadrats per 50-m transect. In both years, supplemental sampling was conducted in the vicinity (<100 m) of each sampling site to confirm or deny the presence of each species.

The density of individuals per 1-m^2 quadrat was estimated with the following equation:

$$\left[\frac{\left(\frac{\sum x}{z}\right)}{q} \pm \frac{\left(\frac{\sigma[x]}{\sigma[z]}\right)}{q}\right] \times n = individuals\ per\ m^2$$

$$Where:$$

$$x = burrows\ per\ occupied\ quadrat\ of\ target\ species\ \ within\ transects\ occupied\ by\ only\ target\ species$$

$$z = average\ number\ of\ burrow\ openings\ per\ excavated\ burrow\ of\ target\ species$$

$$q = number\ of\ occupied\ quadrats\ of\ only\ target\ species$$

$$n = assumed\ population\ of\ single\ burrow$$

In the case that two individuals were found in several burrows (n=3), average quadrat density was calculated for burrow populations of n=1 and n=2.

In both years (2014 and 2015) all quadrats along each transect were searched thoroughly for the presence of active burrows. A burrow was considered active if it had freshly deposited mud at the entrance or the presence of a chimney and lack of debris or spider webs at entrance (Simon 2004). We collected a suite of habitat variables at each quadrat, these variables were later analyzed using generalized linear mixed-models (version 3.1.1; R Project for Statistical Computing, Vienna, Austria; Bates et al. 2014; package lme4) to determine the fine-scale habitat preferences of both species of crayfish. The response variable in each model was the number of burrows within each 1-m^2 quadrat and was modeled with a Poisson error distribution and log link. We modeled burrow counts separately for each species. To account for potential site effects, we modeled transects nested within sites as a random effect in each model**.** We compared candidate models with Akaike's Information Criterion corrected for small sample sizes (AICc; Akaike 1974). A subset of active burrows were hand excavated at each sampling site and along each transect to ensure any burrow counted at a sampling site harbored the target species. Hand excavation consists of using a hand shovel to dig slowly around the burrow entrance and following the main burrow tunnel, feeling for the crayfish as one works their way through the burrow complex. This method was chosen over other methods due to the success rate and limited amount of time spent at each burrow location (Ridge et al. 2008). Voucher specimens of each

target species were collected and preserved in 70% ethanol from all sites with burrows present and deposited in the Illinois Natural History Survey Crustacean Collection.

RESULTS

The field surveys from 2014 and 2015 resulted in the sampling of 690 quadrats across 91 sites for *F. harpi* and 702 quadrats across 89 sites for *P. reimeri* (**Fig. 4.1; Appendix C, D; Table C.1, D.1**) in western Arkansas. All of the historic localities sampled were near (<50 m) primary, secondary, and tertiary roadways. In 2014, we sampled adjacent habitat near (<100 m) the historic sites, out of the right of way in an attempt to survey different habitat types spatially available to individuals at each site. In 2015, all of the sites sampled were in the right of way of primary, secondary, and tertiary roadways. Based on these field surveys, *F. harpi* was found to be present in 79 quadrats across 15 sites, and *P. reimeri* was found to be present in 90 quadrats across 20 sites. During the field sampling we encountered 14 other species of crayfish (*Cambarus ludovicianus*, *F. fodiens*, *F. jeanae*, *F. jeanae x F. strawni*, *F. strawni*, *P. (Girardiella) sp.*, *P. acutus*, *P. curdi*, *P. liberorum*, *P. parasimulans*, *P. regalis*, *P. simulans*, *P. tenuis*, and *P. tulanei*; **Appendix B; Table B.1**). Voucher specimens of these species were deposited in the Illinois Natural History Survey Crustacean Collection.

Fallicambarus harpi

Of the 91 sites sampled for *F. harpi*, the species was found at all historical sites (n = 11) and 5% of sites sampled in 2015 (n = 5) (**Fig. 4.2**). We counted 214 active burrows in both years. The modeling analysis revealed the number of burrows in a quadrat was negatively associated with canopy cover, herbaceous ground cover, and percent clay composition of the soil. The presence of hydrophilic sedges and amount of solar radiation was positively associated with the

number of burrows in a quadrat. Burrows were generally present in quadrats with little to no canopy cover (n = 79, μ = 4.8%, σ = 18.7). Sedges were present in 76% of the quadrats with burrows present (n = 60) and present in 40% of quadrats where burrows were absent (n = 247). Assuming a burrow population of one individual, the density of individuals per 1-m^2 quadrat harboring burrows of only *F. harpi* was 1.82 (±0.06) individuals per m^2 of occupied quadrat. Assuming a burrow population of two individuals the density of individuals per 1-m^2 quadrat harboring burrows of only *F. harpi* was 3.64 (±0.12) individuals per m^2 of occupied quadrat. *Fallicambarus harpi* was captured in one transect with *P. parasimulans* present and one transect with *P. simulans*.

Procambarus reimeri

Of the 89 sites sampled for *P. reimeri*, the species was found at all but one historical site sampled (n = 8) and 14% of the sites sampled in 2015 (n = 16) (**Fig. 4.2**). We counted 140 active burrows in both years. The modeling analysis revealed the number of burrows in a quadrat was negatively associated with canopy cover. The presence of hydrophilic sedges and amount of solar radiation was positively associated with the number of burrows in a quadrat. Burrows were generally present in quadrats with little to no canopy cover (n = 90, μ = 4.1%, σ = 12.8). Sedges were present in 94% of the quadrats with burrows present (n = 85) and present in 44% of quadrats where burrows were absent (n = 269). Assuming a burrow population of one individual the density of individuals per 1-m^2 quadrat harboring burrows of only *P. reimeri* was 0.93 (±0.01) individuals per m^2 of occupied quadrat. Assuming a burrow population of two individuals the density of individuals per 1-m^2 quadrat harboring burrows of only *P. reimeri* was 1.86 (±0.02) individuals per m^2 of occupied quadrat. *Procambarus reimeri* was captured in one transect where *P. tenuis* was present.

DISCUSSION

Our study assessed the range and habitat of two primary burrowing crayfish species in southwestern Arkansas. We recorded *F. harpi* at all of the historic sites sampled and *P. reimeri* at all but one historic site. Although we did not visit all historical locations, historic sites sampled encompassed the (then) known range of both species. Our sampling scheme resulted in most (91% and 93% for *F. harpi* and *P. reimeri*) of the sampling sites to be near or in the right of way of primary, secondary, and tertiary roadways. Although most of the sampling was conducted in these highly altered habitats, our field surveys revealed new populations of both species. Both species of primary burrowing crayfish showed common patterns of habitat preference, which correspond with early accounts of both species preferred habitat (Hobbs 1979, Hobbs and Robison 1985). Based on this study, we recommend *F. harpi* retain its conservation status category of Vulnerable and *P. reimeri* be downgraded to Vulnerable based on Taylor et al. 2007.

The range of *F. harpi* was expanded marginally by approximately 91 km^2 with five new populations, including one found in a county (Clark) not previously documented in its range (**Fig. 4.2**). The range of *P. reimeri* was expanded by a larger margin, approximately 1404 km^2, including 16 new populations. Once again, one of these populations was discovered in a county (Montgomery) previously not known in its range (**Fig. 4.2**). The range of both species continues to be relatively small compared to most of the other primary burrowing crayfishes in the OME (e.g. *F. jeanae*, *F. strawni*, *P. liberorum,* and *P. parasimulans*). We are confident the ranges of *F. harpi* and *P. reimeri* are now accurately delineated in Arkansas. *P. reimeri* was captured near (<1 km) the state line of Oklahoma in Arkansas (**Fig. 4.2**), leading to the conclusion *P. reimeri* may occur in Oklahoma. We recommend more field surveys to assess this possibility.

Both *F. harpi* and *P. reimeri* showed common patterns of habitat use. The habitat we found animals to be most abundant in was composed of treeless, wet seepage areas with an abundance of low grasses and sedges. The soil composition at these occupied sites was primarily loam and silt loam (Soil Survey Division Staff 1993), present in 92% of *F. harpi* quadrats and 94% of *P. reimeri* quadrats. The burrows of *F. harpi* and *P. reimeri* excavated in the field were complex, 0.5 to 1 meter in depth, and connected to groundwater. Our results confirm the specific importance of these wet, open canopy habitats as a preferred environment for both species.

The success in finding *F. harpi* at all of the historical sites sampled and five new populations indicates this crayfish is geographically constrained but relatively stable. Its total range is estimated to be 265 km^2 using a minimum convex polygon encompassing all known populations. The area within this range deemed as suitable habitat is estimated as 109 km^2. This area was calculated by summing the area predicted above the lowest relative occurrence rate associated with any one of the presence localities from 2014 and 2015 from the Maxent analysis. These results suggest this crayfish continue to be categorized as Vulnerable due to its restricted range in accordance with Taylor et al. 2007. The success of finding *P. reimeri* at all but one historical site and 16 new populations, one of which was greater than 50 km away from the previously known range, indicates this crayfish is somewhat geographically constrained but is more widespread than originally thought. Its total range is estimated to be 1467 km^2 using a minimum convex polygon encompassing all known populations. The area within this range deemed as suitable habitat is estimated as 178 km^2. This area was calculated by summing the area predicted above the lowest relative occurrence rate associated with any one of the presence localities from 2014 and 2015 from the Maxent analysis. These results suggest *P. reimeri* should be categorized as Vulnerable as opposed to Endangered as dictated by Taylor et al. 2007.

TABLES AND FIGURES

Figure 4.1: Sampling locations for two species of primary burrowing crayfish, *Fallicambarus harpi* (top) and *Procambarus reimeri* (bottom), in western Arkansas in the spring of 2014 and 2015. Some triangles and squares represent more than one quadrat.

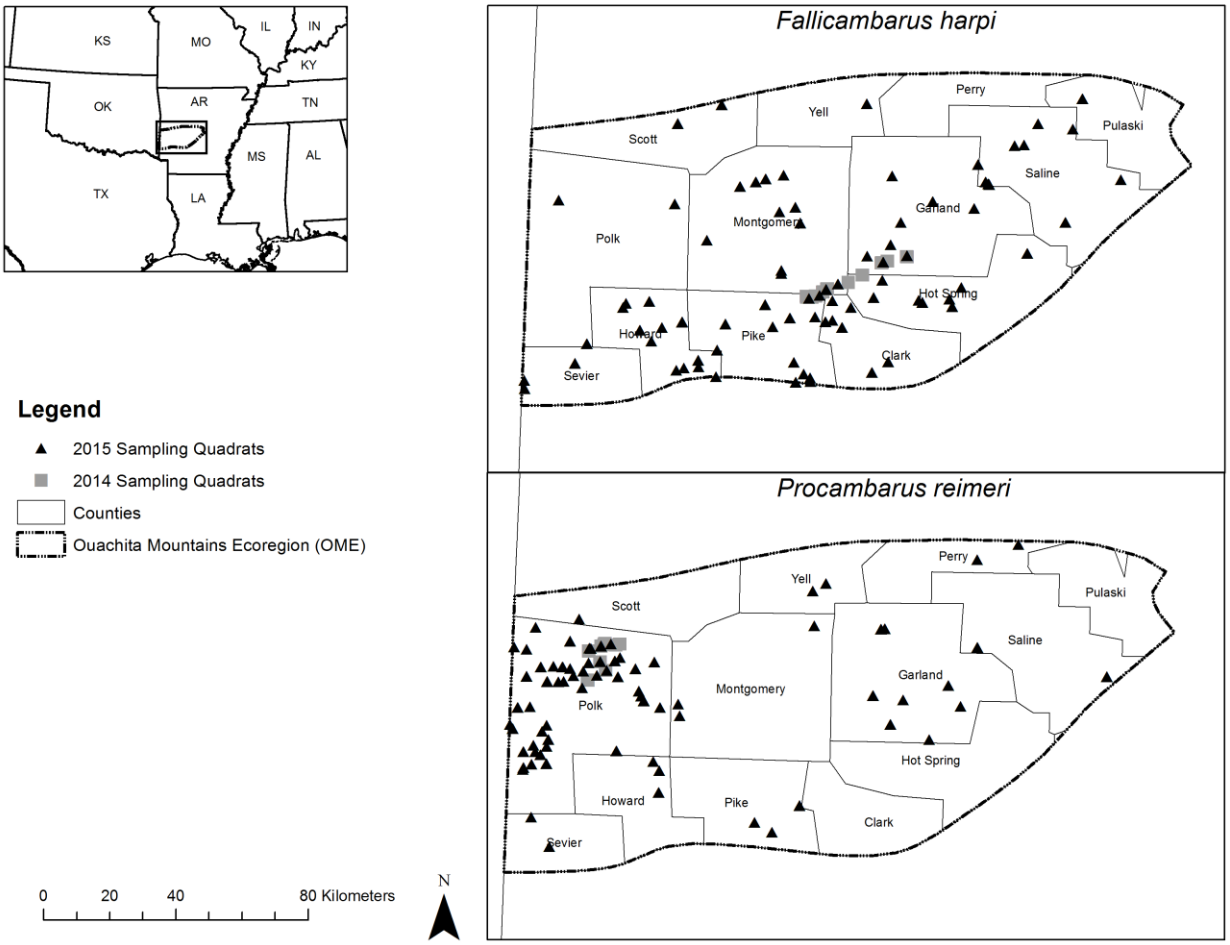

Figure 4.2: Historical and current capture locations of *Fallicambarus harpi* (right) and *Procambarus reimeri* (left) in southwestern Arkansas. Some triangles and squares represent more than one presence location.

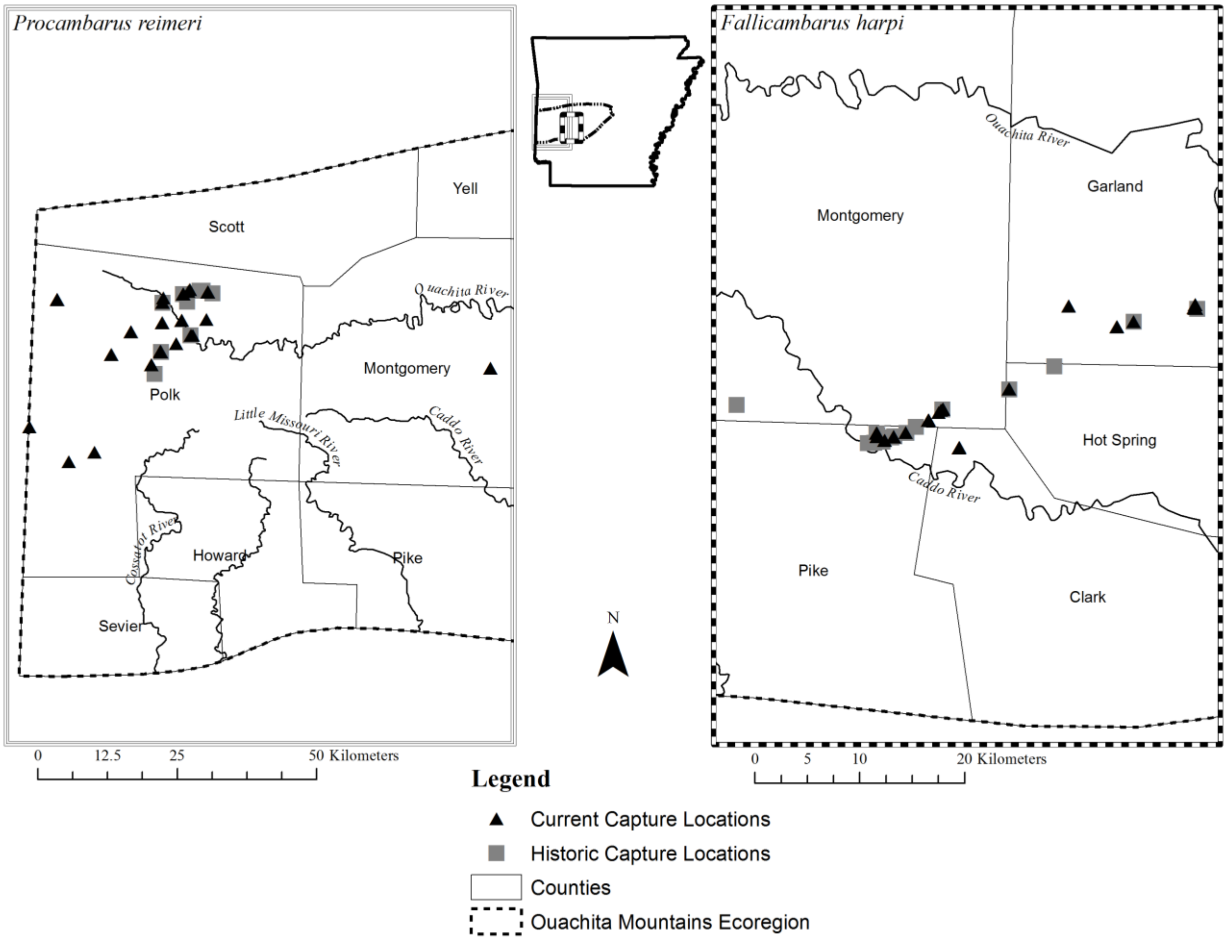

Table 4.1: Capture information for *Fallicambarus harpi* in the spring of 2014 and 2015 in south-western Arkansas.

Date	Drainage	County	Latitude	Longitude	Form I	Form II	Female	Total
4/8/2014	Ouachita River	Garland	34.44381	-93.21650	3	0	3	6
4/8/2014	Ouachita River	Garland	34.44020	-93.21207	0	0	4	4
4/8/2014	Ouachita River	Garland	34.44383	-93.21063		1		1
4/8/2014	Ouachita River	Garland	34.43239	-93.27458	1	0	1	2
4/8/2014	Ouachita River	Garland	34.43238	-93.27550	1	1	1	3
4/8/2014	Ouachita River	Garland	34.42776	-93.29308		1	1	2
4/9/2014	Ouachita River	Garland	34.42746	-93.29530	1	2		3
4/9/2014	Ouachita River	Hot Spring	34.39401	-93.35677		1		1
4/9/2014	Ouachita River	Hot Spring	34.39405	-93.35772			1	1
4/9/2014	Caddo River	Pike	34.33596	-93.51162		1		1
4/9/2014	Caddo River	Pike	34.33606	-93.51176			1	1
4/9/2014	Caddo River	Pike	34.33268	-93.52367	1	1	2	4
4/9/2014	Caddo River	Pike	34.33539	-93.54180	1		2	3
4/10/2014	Caddo River	Montgomery	34.34728	-93.48791	2	1	0	3
4/10/2014	Caddo River	Montgomery	34.35593	-93.47363	1	1		2
4/10/2014	Caddo River	Montgomery	34.35617	-93.47311			1	1
4/10/2014	Caddo River	Montgomery	34.35578	-93.47449	1		1	2
4/10/2014	Ouachita River	Hot Spring	34.37383	-93.40457	1	1		2
3/28/2015	Ouachita River	Pike	34.32927	-93.53305			4	4
3/28/2015	Ouachita River	Garland	34.44567	-93.34285		1		1
3/29/2015	Ouachita River	Clark	34.32352	-93.45580	2		2	4
3/28/2015	Ouachita River	Montgomery	34.35361	-93.47739	1			1
3/26/2015	Ouachita River	Garland	34.44689	-93.21102	1		2	3

Table 4.2: Capture information for *Procambarus reimeri* in the spring of 2014 and 2015 in south-western Arkansas.

Date	Drainage	County	Latitude	Longitude	Form I	Form II	Female	Total
4/21/2012	Ouachita River	Polk	34.58384	-94.14814			2	2
4/21/2012	Ouachita River	Polk	34.58403	-94.14822	1		1	2
4/21/2012	Ouachita River	Polk	34.60709	-94.16787			1	1
4/22/2014	Ouachita River	Polk	34.65405	-94.11627	1			1
4/22/2014	Ouachita River	Polk	34.65777	-94.15186		1		1
4/22/2014	Ouachita River	Polk	34.65734	-94.15216			1	1
4/22/2014	Ouachita River	Polk	34.65041	-94.16422			1	1
4/23/2014	Ouachita River	Polk	34.63678	-94.20505	1		3	4
4/23/2014	Ouachita River	Polk	34.55721	-94.20782	1			1
4/23/2014	Ouachita River	Polk	34.55682	-94.20712			4	4
4/23/2014	Ouachita River	Polk	34.65411	-94.11646	2			2
4/23/2014	Ouachita River	Polk	34.65422	-94.15506			2	2
3/31/2015	Ouachita River	Montgomery	34.53489	-93.56304	1		1	2
4/14/2015	Ouachita River	Polk	34.65025	-94.16466	1			1
4/11/2015	Ouachita River	Polk	34.53557	-94.22560		1		1
4/15/2015	Ouachita River	Polk	34.60364	-94.20482		1	1	2
4/18/2015	Red River	Polk	34.43276	-94.46193			3	3
4/18/2015	Red River	Polk	34.37649	-94.38387	1	1	1	3
4/11/2015	Red River	Polk	34.58804	-94.26631		1	6	7
4/10/2015	Red River	Polk	34.63903	-94.41092	1		3	4
4/18/2015	Red River	Polk	34.39311	-94.33360	2			2
4/15/2015	Ouachita River	Polk	34.60993	-94.11894	1			1
4/9/2015	Red River	Polk	34.55400	-94.30660	1		1	2
4/15/2015	Ouachita River	Polk	34.58384	-94.14469			1	1
4/15/2015	Ouachita River	Polk	34.57040	-94.17729	2			2
4/14/2015	Ouachita River	Polk	34.64380	-94.20291	1		1	2
4/14/2015	Ouachita River	Polk	34.65010	-94.16549	1			1

LITERATURE CITED

Bates, D., M. Maechler, B. Bolker, and S. Walker. 2014. lme4: linear mixed-effects models using 'Eigen' and S4. R Project for Statistical Computing, Vienna, Austria. (Available from: http://cran.r-project.org/package=lme4)

Fithian, W., and T. Hastie. 2013. Finite-sample equivalence in statistical models for presence-absence only data. Annals of Applied Statistics 7:1917–1939.

Hobbs, H.H., Jr. and H.W. Robison. 1985. A new burrowing crayfish (Decapoda: Cambaridae) from southwestern Arkansas. Proceedings of the Biological Society of Washington 98(4):1035-1041.

Hobbs, H. H., Jr. 1979. A new crayfish from the Ouachita River basin in Arkansas (Decapoda: Cambaridae). Proceedings of the Biological Society of Washington 92(4):804–811.

Hobbs, H. H., Jr. 1981. The Crayfishes of Georgia. Smithsonian Contributions to Zoology. Smithsonian Contributions to Zoology, City of Washington.

Moore, M. J., R. J. DiStefano, and E. R. Larson. 2013. An assessment of life-history studies for USA and Canadian crayfishes: identifying biases and knowledge gaps to improve conservation and management. Freshwater Science 32(4):1276–1287.

Morehouse, R. L., and M. Tobler. 2013. Crayfishes (Decapoda: Cambaridae) of Oklahoma: identification, distributions, and natural history. Zootaxa 3717(2):101–157.

Page, L. 1985. The crayfishes and shrimps (Decapoda) of Illinois. Illinois Natural History Survey Bulletin. 33(4):i-339.

Phillips, S. J., R. P. Anderson, and R. E. Schapire. 2006. Maximum entropy modeling of species geographic distributions. Ecological Modelling 190:231–259.

Phillips, S. J., and M. Dudík. 2008. Modeling of species distributions with Maxent: new extensions and a comprehensive evaluation. Ecography 31:161–175.

Ridge, J., T. P. Simon, D. Karns, and J. Robb. 2008. Comparison of Three Burrowing Crayfish Capture Methods Based on Relationships with Species Morphology, Seasonality, and Habitat Quality. *Journal of Crustacean Biology* 28(3):466–472.

Robison, H. W., and B. Crump. 2004. Distribution, natural history aspects, and status of the Arkansas endemic crayfish, *Fallicambarus harpi* Hobbs and Robison, 1985. *Journal of the Arkansas Academy of Science* 58:91–94.

Robison, H. W., C. McAllister, C. Carlton, and G. Tucker. 2008. The Arkansas endemic biota: An update with additions and deletions. *Journal of the Arkansas Academy of Science* 62:84-96.

Robison, H. W. 2008. Distribution, life history aspects, and conservation status of three Ouachita Mountain crayfishes: *Procambarus tenuis*, *P. reimeri*, and *Orconectes menae*. U.S.D.A. Forest Service, Ouachita National Forest. Hot Springs, Arkansas.

Simmons, J. W., and S. J. Fraley. 2010. Distribution, Status, and Life-History Observations of Crayfishes in Western North Carolina. Southeastern Naturalist 9(3):79–126.

Simon, T. P. 2004. Standard operating procedures for the collection and study of burrowing crayfish in Indiana. I. Methods for the collection of burrowing crayfish in streams and terrestrial habitats. Miscellaneous Papers of the Indiana Biological Survey Aquatic Research Center Number 2.

Soil Survey Division Staff. 1993. Soil survey manual. Handbook 1. Soil Conservation Service, US Department of Agriculture, Washington, DC. (Available from: http://www.nrcs.usda.gov/Internet/FSE_DOCUMENTS/nrcs142p2_050993.pdf)

Taylor, C. A., G. A. Schuster, J. E. Cooper, R. J. DiStefano, A. G. Eversole, P. Hamr, H. H. Hobbs III, H. W. Robison, C. E. Skelton, and R. F. Thoma. 2007. A reassessment of the

conservation status of crayfishes of the United States and Canada after 10+ years of increased awareness. Fisheries 32:372–389.

Woods, A. J., T. L. Foti, S. S. Chapman, J. M. Omernik, J. A. Wise, E. O. Murray, W. L. Prior, J. B. J. Pagan, J. A. Comstock, and M. Radford. 2004. Ecoregions of Arkansas (color poster with map, descriptive text, summary tables, and photographs). US Geological Survey map scale 1:1,000,000. U.S. Geological Survey, Reston, Virginia.

SUMMARY

Rare species are important to ecosystem function and are frequently the target of conservation priorities. The very nature of rare species makes them difficult to study comprehensively. A group of rare understudied species are North American primary burrowing crayfishes, which reach their highest diversity in the southeastern United States. Within the southeast, primary burrowing crayfishes reach high diversity in the Ouachita Mountains Ecoregion of Arkansas. Two species of primary burrowing crayfish, *Fallicambarus harpi,* and *Procambarus reimeri,* endemic to this ecoregion had a large knowledge gap in basic natural history. To address this knowledge gap, my research assessed the habitat preferences, distribution, and conservation statuses of both crayfishes.

My study of the habitat preferences *F. harpi* and *P. reimeri* revealed both to be habitat specialists, keying in on the open canopy, wet microhabitats. These habitats were similar to or within roadside ditches, and field observations showed the animals largely did not occur in the adjacent habitat. I conclude that these results support the benefit of the right of ways (ROWs) to the persistence of these narrowly endemic habitat specialists. My study on the ability of species distribution models to predict suitable habitat outside of the historically known range of both species revealed Maxent to be a suitable tool for this endeavor. The results of my study revealed new populations of both species, expanding the range of *F. harpi* marginally (<100 km^2) and the range of *P. reimeri* by a larger margin (>1000 km^2), both into counties from which they were not previously known. The conservation status of both crayfishes can be assessed with my data, and it revealed that while both *F. harpi* and *P. reimeri* are geographically constrained, they are locally stable in many roadside ditches. I conclude that the roadside ditch is functioning as

suitable and preferred habitat within a matrix of unsuitable habitat for these two species within the Ouachita Mountains Ecoregion.

APPENDIX A: CRAYFISH SAMPLING SUPPLEMENTARY MATERIAL

Table A.1: General statistics of habitat covariates and counts measured at each quadrat sampled for two crayfish species (*Fallicambarus harpi* and *Procambarus reimeri*) in western Arkansas in the spring of 2014 and 2015. See Table 2.1 in main body of thesis for a description of each variable.

2014 General Statistics

	n	Sedge	Canopy	Herb	Stem	Burrow	Elevation	Solar	Water_dist	CTI	Sand	Silt	Clay
Fallicambarus harpi			average(std)										
All quadrats	210	126	25.8(39.9)	67.2(35.2)	17.9(15.7)	0.7(1.4)	186.1(12.7)	6213(15.9)	142.1(100.4)	8.4(1.6)	36(8.2)	49.2(6.3)	14.8(5.3)
Quadrats with burrows present	58	43	6.6(21.8)	82(22.9)	23.5(16.81)	2.5(1.7)	183.4(10.4)	6215.9(8.3)	159.2(81.6)	8.5(1.6)	33.3(8.6)	40.6(6.5)	16.1(6.7)
Quadrats with burrows absent	152	83	33.2(42.9)	61.6(37.5)	15.7(14.9)	0(0)	187.1(13.4)	6211.3(17.8)	135.6(106.5)	8.3(1.7)	37.0(7.9)	48.6(6.2)	16.1(6.7)
Quadrats in ROW	150	98	5.6(16.4)	80.9(24.4)	22.7(15.4)	0.9(1.6)	184.9(12.3)	6211.6(17.5)	162.8(109.0)	8.4(1.5)	35.6(8.0)	49.1(5.8)	15.3(5.7)
Quadrats scored as adjacent	60	28	76.5(36.3)	33.1(34.8)	5.7(8.4)	0.1(0.3)	189.0(13.3)	6214.9(10.5)	90.3(42.7)	8.2(1.8)	36.8(8.7)	49.5(7.5)	13.6(3.9)
Procambarus reimeri			average(std)										
All quadrats	222	142	22.5(37.2)	71.4(36.1)	31(21.4)	0.3(0.7)	325.6(17.0)	6089(10.7)	93.9(48.6)	8.9(1.6)	29.6(8.6)	50.6(7.4)	19.8(5.5)
Quadrats with burrows present	52	48	2.3(12.0)	83.9(22.3)	37.0(18.2)	1.4(0.7)	326.6(15.8)	6087.5(9.7)	105.4(54.9)	8.7(1.6)	28.2(9.4)	50.8(7.9)	20.9(4.6)
Quadrats with burrows absent	170	94	28.7(40.1)	67.5(38.7)	29.2(22.0)	0(0)	325.3(14.4)	6089.1(11.0)	90.3(46.3)	9.0(1.6)	30.0(8.4)	50.5(7.24)	19.5(5.8)
Quadrats in ROW	174	121	6.6(20.4)	84.6(23.1)	38.2(18.1)	0.4(0.7)	323.9(16.8)	6087.0(10.2)	95.9(53.8)	9.0(1.6)	28.8(8.9)	50.7(7.6)	20.5(5.5)
Quadrats scored as adjacent	48	21	80.4(24.7)	22.4(31.9)	4.9(7.6)	0(0)	331.8(16.3)	6095.2(9.9)	86.5(19.4)	8.8(1.5)	32.3(7.2)	50.3(6.7)	17.4(4.9)

2015 General Statistics

	n	Sedge	Canopy	Herb	Stem	Burrow	Elevation	Solar	Water_dist	CTI	Sand	Silt	Clay
Fallicambarus harpi			average(std)										
All quadrats	480	220	21(28.5)	66.5(31.6)	24.7(30.9)	0.1(1)	190.7(38.3)	5997.4(41.7)	191.3(122.7)	8.4(1.8)	32(12.3)	56.3(10)	11.7(4.3)
Quadrats with burrows present	21	17	0(0)	63(24.5)	23.7(11.6)	3.3(3.5)	182.8(11.8)	6000.7(6)	219.6(82.9)	8.8(1.9)	36.4(6.3)	51.9(2.5)	11.6(4.8)
Quadrats with burrows absent	459	203	22(28.8)	66.6(31.9)	24.7(21.2)	0(0)	191.1(39.0)	5997.3(42.6)	190(124)	8.4(1.8)	31.8(12.5)	56.5(10.1)	11.7(4.3)
Procambarus reimeri			average(std)										
All quadrats	480	214	22.4(30.6)	71.8(28.4)	22.8(19.9)	0.1(0.6)	294.6(69.1)	6063.3(48.2)	186.6(155.1)	8.1(1.4)	30.2(10.2)	57.7(8.2)	12.1(3.1)
Quadrats with burrows present	38	37	6.6(13.5)	71.1(15.2)	29.3(16.9)	1.8(1)	325.2(18.5)	6089(15.6)	187.1(102.5)	8.7(1.2)	30.2(8.3)	57.6(6.7)	12.2(2.1)
Quadrats with burrows absent	442	177	23.8(31.3)	71.9(29.2)	22.2(20)	0(0)	292(71.2)	6061.1(49.4)	186.5(158.8)	8.1(1.4)	30.2(10.3)	57.7(8.3)	12.1(3.2)

Table A.1 cont'd

2014 and 2015 General Statistics													
	**n**	**Sedge**	**Canopy**	**Herb**	**Stem**	**Burrow**	**Elevation**	**Solar**	**Water_dist**	**CTI**	**Sand**	**Silt**	**Clay**
Fallicambarus harpi			average(std)										
All quadrats	690	346	22.5(32.5)	66.6(32.4)	22.7(19.7)	0.5(1.4)	189.3(32.8)	6062.9(105.3)	176.3(118.5)	8.4(1.7)	33.2(11.4)	54.1(9.6)	12.6(4.8)
Quadrats with burrows present	79	60	4.8(18.7)	77.0(24.7)	23.5(15.5)	2.6(2.1)	183.2(10.7)	6158.7(95.3)	175.3(85.7)	8.6(1.6)	34.1(8.1)	50.9(5.7)	14.9(6.5)
Quadrats with burrows absent	611	286	24.7(33.2)	65.2(33.0)	22.6(20.2)	0	190.1(34.5)	6050.5(100.0)	176.4(122.1)	4(1.7)	33.1(11.7)	54.5(9.9)	12.3(4.5)
Procambarus reimeri			average(std)										
All quadrats	702	356	22.5(32.8)	71.7(31.0)	25.4(20.7)	0.2(0.6)	304.4(59.7)	6071.3(42.0)	157.2(138.0)	8.4(1.5)	30.0(9.7)	55.5(8.6)	14.6(5.4)
Quadrats with burrows present	90	85	4.1(12.8)	78.5(20.4)	33.7(18.0)	1.6(0.9)	326.0(16.9)	6088.1(12.5)	139.9(88.2)	8.7(1.4)	29.1(9.0)	53.7(8.1)	17.3(5.7)
Quadrats with burrows absent	612	271	25.2(34.0)	70.7(32.2)	24.2(20.8)	0	301.3(63.0)	6068.9(44.2)	159.8(143.8)	8.3(1.5)	30.1(9.8)	55.7(8.7)	14.2(5.2)

APPENDIX B: CRAYFISH CAPTURE SUPPLEMENTARY MATERIAL

Table B.1: Capture information for all crayfishes captured during the spring of 2014 and 2015 in western Arkansas during a study to assess the habitat preferences of two primary burrowing crayfish species (*Fallicambarus harpi* and *Procambarus reimeri*). "# of openings" refers to the number of burrow openings counted during the excavation of each captured individual.

Transect #	Date	Latitude	Longitude	Species	Form I	Form II	Female	County	# of openings
1	8-Apr-14	34.44381	-93.2165	*Fallicambarus harpi*	1		1	Garland	1
1	8-Apr-14	34.44381	-93.2165	*Fallicambarus harpi*	2		2	Garland	2
2	8-Apr-14	34.4402	-93.21207	*Fallicambarus harpi*			2	Garland	1
2	8-Apr-14	34.4402	-93.21207	*Fallicambarus harpi*			2	Garland	2
3	8-Apr-14	34.44383	-93.21063	*Fallicambarus harpi*		1		Garland	1
4	8-Apr-14	34.43239	-93.27458	*Fallicambarus harpi*			1	Garland	2
4	8-Apr-14	34.43239	-93.27458	*Fallicambarus harpi*	1			Garland	1
6	8-Apr-14	34.43238	-93.2755	*Fallicambarus harpi*	1		1	Garland	1
6	8-Apr-14	34.43238	-93.2755	*Fallicambarus harpi*		1		Garland	2
8	8-Apr-14	34.42776	-93.29308	*Fallicambarus harpi*		1	1	Garland	2
10	8-Apr-14	34.42746	-93.2953	*Fallicambarus harpi*	1	2		Garland	Unknown
11	9-Apr-14	34.39401	-93.35677	*Fallicambarus harpi*		1		Hot Spring	2
14	9-Apr-14	34.39405	-93.35772	*Fallicambarus harpi*			1	Hot Spring	1
15	9-Apr-14	34.33596	-93.51162	*Fallicambarus harpi*		1		Pike	1
16	9-Apr-14	34.33606	-93.51176	*Fallicambarus harpi*			1	Pike	1
18	9-Apr-14	34.33268	-93.52367	*Fallicambarus harpi*	1	1	1	Pike	1
18	9-Apr-14	34.33268	-93.52367	*Fallicambarus harpi*			1	Pike	2
21	9-Apr-14	34.33539	-93.5418	*Fallicambarus harpi*	1		2	Pike	1
26	10-Apr-14	34.34728	-93.48791	*Fallicambarus harpi*	1			Montgomery	2
26	10-Apr-14	34.34728	-93.48791	*Fallicambarus harpi*	1	1		Montgomery	1
29	10-Apr-14	34.35593	-93.47363	*Fallicambarus harpi*	1	1		Montgomery	1
30	10-Apr-14	34.35617	-93.47311	*Fallicambarus harpi*			1	Montgomery	1
31	10-Apr-14	34.35578	-93.47449	*Fallicambarus harpi*	1		1	Montgomery	Unknown
35	10-Apr-14	34.37383	-93.40457	*Fallicambarus harpi*	1			Hot Spring	1
35	10-Apr-14	34.37383	-93.40457	*Fallicambarus harpi*		1		Hot Spring	2

Table B.1 cont'd

Transect #	Date	Latitude	Longitude	Species	Form I	Form II	Female	County	# of openings
36	21-Apr-12	34.58384	-94.14814	*Procambarus reimeri*			2	Polk	1
38	21-Apr-12	34.58403	-94.14822	*Procambarus reimeri*	1		1	Polk	3
39	21-Apr-12	34.60709	-94.16787	*Procambarus reimeri*			1	Polk	1
45	22-Apr-14	34.65405	-94.11627	*Procambarus reimeri*	1			Polk	1
50	22-Apr-14	34.65777	-94.15186	*Procambarus reimeri*		1		Polk	2
51	22-Apr-14	34.65734	-94.15216	*Procambarus reimeri*			1	Polk	3
54	22-Apr-14	34.65041	-94.16422	*Procambarus reimeri*			1	Polk	1
56	23-Apr-14	34.63678	-94.20505	*Procambarus reimeri*			1	Polk	1
56	23-Apr-14	34.63678	-94.20505	*Procambarus reimeri*			2	Polk	2
56	23-Apr-14	34.63678	-94.20505	*Procambarus reimeri*	1			Polk	3
63	23-Apr-14	34.55721	-94.20782	*Procambarus reimeri*	1			Polk	2
65	23-Apr-14	34.55682	-94.20712	*Procambarus reimeri*			2	Polk	1
65	23-Apr-14	34.55682	-94.20712	*Procambarus reimeri*			1	Polk	2
65	23-Apr-14	34.55682	-94.20712	*Procambarus reimeri*			1	Polk	3
68	23-Apr-14	34.65411	-94.11646	*Procambarus reimeri*	2			Polk	1
70	23-Apr-14	34.65422	-94.15506	*Procambarus reimeri*			2	Polk	1
9	23-Mar-15	34.74965	-92.82456	*Fallicambarus fodiens*	1			Saline	Unknown
12	23-Apr-15	34.6647	-93.26058	*Procambarus liberorum*	1	1	2	Garland	2
14	28-Mar-15	34.32955	-93.53259	*Fallicambarus harpi*			4	Pike	3
14	28-Mar-15	34.32955	-93.53259	*Procambarus parasimulans*	1	1		Pike	2
16	28-Mar-15	34.4465	-93.34284	*Fallicambarus harpi*		1		Garland	2
18	29-Mar-15	34.32352	-93.4558	*Fallicambarus harpi*	2		2	Clark	2
25	1-Apr-15	34.13671	-93.94329	*Procambarus parasimulans*	1	1		Howard	Unknown
32	26-Mar-15	34.32851	-93.07111	*Fallicambarus jeanae*	1	1	3	Hot Spring	3
45	28-Mar-15	34.47732	-93.26605	*Fallicambarus sp.*		1	1	Garland	2

Table B.1 cont'd

Transect #	Date	Latitude	Longitude	Species	Form I	Form II	Female	County	# of openings
48	23-Mar-15	34.80642	-92.77906344	*Fallicambarus fodiens*		2	1	Saline	1
49	1-Apr-15	34.18689	-93.83407	*Fallicambarus strawni*	2		3	Pike	2
51	31-Mar-15	34.53489	-93.56304	*Procambarus reimeri*	1		1	Montgomery	3
52	29-Mar-15	34.40977	-93.624246	*Procambarus parasimulans*		1	1	Montgomery	Unknown
53	28-Mar-15	34.35356	-93.47742	*Fallicambarus harpi*	1			Montgomery	1
58	1-Apr-15	34.130704	-93.967227	*Fallicambarus strawni*	2	2	2	Howard	2
68	26-Mar-15	34.44689	-93.211023	*Fallicambarus harpi*	1		2	Garland	1
70	31-Mar-15	34.65402	-93.6782	*Procambarus liberorum*	1	3	1	Montgomery	Unknown
77	2-Apr-15	34.24662	-94.01553	*Fallicambarus strawni*		3	2	Howard	2
87	27-Mar-15	34.1227	-93.55003	*Fallicambarus strawni*		2		Pike	3
90	31-Mar-15	34.25821	-93.80802	*Fallicambarus jeanae*	1	2	2	Pike	3
92	26-Mar-15	34.30846	-93.06246	*Fallicambarus jeanae*			2	Hot Spring	Unknown
93	16-Apr-15	34.58444	-93.97327	*Procambarus liberorum*	1	2		Polk	2
94	26-Mar-15	34.31986	-93.16096	*Fallicambarus jeanae*	1		1	Hot Spring	3
97	27-Mar-15	34.15429	-93.58212	*Cambarus ludovicianus*			1	Pike	1
97	27-Mar-15	34.15429	-93.58212	*Fallicambarus jeanae*		1	1	Pike	2
97	27-Mar-15	34.15429	-93.58212	*Procambarus tulanei*		1		Pike	2
104	25-Mar-15	34.64087	-92.93977	*Fallicambarus fodiens*			1	Saline	1
109	1-Apr-15	34.15819	-93.8943	*Procambarus tenuis*		9		Howard	Unknown
117	2-Apr-15	34.09761	-94.46558	*Procambarus regalis*		1	1	Sevier	Unknown
120	23-Mar-15	34.74667	-92.85654	*Fallicambarus fodiens*		5		Saline	Unknown
132	29-Mar-15	34.26529	-93.47874	*Fallicambarus jeanae*	1			Clark	2
140	1-Apr-15	34.1141	-93.83714	*Fallicambarus strawni*		1		Howard	Unknown
166	14-Apr-15	34.650252	-94.164662	*Procambarus reimeri*	1			Polk	Unknown
167	11-Apr-15	34.53557	-94.2256	*Procambarus reimeri*		1		Polk	1
170	15-Apr-15	34.60364	-94.20482	*Procambarus reimeri*		1	1	Polk	1

Table B.1 cont'd

Transect #	Date	Latitude	Longitude	Species	Form I	Form II	Female	County	# of openings
175	18-Apr-15	34.43276	-94.46193	*Procambarus reimeri*			3	Polk	1
197	18-Apr-15	34.37649	-94.38387	*Procambarus reimeri*	1	1	1	Polk	2
205	11-Apr-15	34.58804	-94.26631	*Procambarus reimeri*		1	6	Polk	1
216	10-Apr-15	34.63903	-94.41092	*Procambarus reimeri*	1		3	Polk	3
221	19-Apr-15	34.3512	-94.35957	*Procambarus liberorum*	1		1	Polk	1
236	18-Apr-15	34.39311	-94.3336	*Procambarus reimeri*	2			Polk	2
239	2-Apr-15	34.36409	-94.10996	*Fallicambarus strawni*		1	1	Polk	6
241	15-Apr-15	34.609934	-94.118939	*Procambarus reimeri*	1			Polk	1
247	9-Apr-15	34.554	-94.3066	*Procambarus reimeri*	1		1	Polk	1
259	15-Apr-15	34.58384	-94.14469	*Procambarus reimeri*			1	Polk	2
269	15-Apr-15	34.570403	-94.17729	*Procambarus reimeri*	2			Polk	1
279	14-Apr-15	34.643799	-94.202914	*Procambarus reimeri*	1		1	Polk	2
279	14-Apr-15	34.643799	-94.202914	*Procambarus sp.*		1	10	Polk	Unknown
296	28-Mar-15	34.17333	-93.65472	*Fallicambarus strawni*		1		Pike	Unknown
300	30-Mar-15	34.70973	-93.46104	*Procambarus sp.*		11		Montgomery	Unknown
307	27-Mar-15	34.14621	-93.59632	*Fallicambarus strawni*		1	3	Pike	3
308	29-Mar-15	34.21942	-93.50683	*Fallicambarus jeanae x F. strawni*	1		2	Pike	2
318	30-Mar-15	34.8255	-93.42265	*Procambarus liberorum*			1	Yell	Unknown
182/234	10-Apr-15	34.59201	-94.28992	*Procambarus (Girardiella) sp.*		1	6	Polk	1
207/166	14-Apr-15	34.6501	-94.16549	*Procambarus reimeri*	1			Polk	1
	7-Apr-15	34.25258	-93.809908	*Fallicambarus jeanae*	1		1	Pike	2
	29-Mar-15	34.26569	-93.45975	*Fallicambarus jeanae*	1		1	Clark	Unknown
	7-Apr-15	34.05919	-93.69859	*Fallicambarus fodiens*			2	Pike	1
	7-Apr-15	34.05919	-93.69859	*Procambarus acutus*		3	1	Pike	0
	8-Apr-15	34.248893	-93.628901	*Fallicambarus jeanae*	1		3	Pike	1
	7-Apr-15	34.310806	-93.746535	*Fallicambarus sp.*			1	Pike	2
	7-Apr-15	34.224156	-93.896305	*Cambarus ludovicianus*			2	Pike	2

Table B.1 cont'd

Transect #	Date	Latitude	Longitude	Species	Form I	Form II	Female	County	# of openings
	7-Apr-15	34.224156	-93.896305	*Fallicambarus strawni*			2	Pike	2
	8-Apr-15	34.106697	-93.879246	*Fallicambarus fodiens*		1	7	Howard	2
	8-Apr-15	34.106697	-93.879246	*Fallicambarus jeanae*		1	6	Howard	2
	8-Apr-15	34.042506	-93.737006	*Fallicambarus jeanae*	1	1	1	Pike	2
	7-Apr-15	34.05585	-93.7023	*Fallicambarus sp.*			14	Pike	1
	7-Apr-15	34.05585	-93.7023	*Procambarus sp.*		2	16	Pike	0
	7-Apr-15	34.073844	-93.676618	*Fallicambarus jeanae*	1		3	Pike	
	8-Apr-15	34.23484	-93.51176	*Fallicambarus jeanae*			1	Pike	1
	8-Apr-15	34.08532	-93.70537	*Fallicambarus jeanae*	1	1	9	Pike	2
	7-Apr-15	34.08194	-93.66749	*Fallicambarus sp.*			2	Pike	

APPENDIX C: *FALLICAMBARUS HARPI* SAMPLING SUPPLEMENTARY MATERIAL

Table C.1: Habitat covariates and counts measured at each quadrat for *Fallicambarus harpi* in western Arkansas in the spring of 2014 and 2015. See Table 2.1 in main body of thesis for a description of each variable. The water_trns corresponds to the water_trans covariate in the main body, this variable was not measured in 2015. The burrow ownership column depicts the assumed ownership of each burrow counted in the corresponding quadrat.

Site	Transect	Quadrat	Date	Latitude	Longitude	Canopy	Herb	Sedge	Stem	Burrow	Elevation	Solar	Water_dist	CTI	Sand	Silt	Clay	Burrow ownership	Water_trns
1	1	1	4/8/2014	34.444382	-93.212699	0	96	1	14	1	172.07	6215.49	327.01	6.55	29.55	51.43	19.02	*F. harpi*	N
1	1	2	4/8/2014	34.44436	-93.212593	0	90	0	20	0	172.03	6214.54	317.12	6.53	29.55	51.43	19.02		N
1	1	3	4/8/2014	34.444338	-93.212486	0	70	0	33	4	171.40	6212.66	307.15	7.63	36.13	49.16	14.71	*F. harpi*	N
1	1	4	4/8/2014	34.444315	-93.212379	0	95	1	8	4	171.08	6211.40	297.18	7.65	36.13	49.16	14.71	*F. harpi*	N
1	1	5	4/8/2014	34.444293	-93.212272	0	91	0	26	3	170.85	6211.70	287.22	8.34	35.01	48.22	16.63	*F. harpi*	N
1	1	6	4/8/2014	34.444271	-93.212165	1	87	0	9	0	170.76	6211.81	277.29	9.40	35.01	48.22	16.63		N
1	2	7	4/8/2014	34.444024	-93.212069	0	92	0	7	0	170.12	6210.04	268.56	8.20	27.85	59.98	12.17		N
1	2	8	4/8/2014	34.444002	-93.211962	0	97	0	15	1	170.17	6210.01	258.90	8.03	27.85	59.98	12.17	*F. harpi*	N
1	2	9	4/8/2014	34.44398	-93.211855	0	96	1	35	5	170.12	6209.98	249.27	8.03	28.36	57.53	14.11	*F. harpi*	N
1	2	10	4/8/2014	34.443958	-93.211748	0	99	0	11	2	170.23	6209.73	239.68	8.04	28.36	57.53	14.11	*F. harpi*	N
1	2	11	4/8/2014	34.443936	-93.211641	0	98	0	22	1	170.36	6210.38	230.14	7.27	33.03	53.27	13.37	*F. harpi*	N
1	2	12	4/8/2014	34.443914	-93.211534	0	97	1	21	4	170.42	6210.70	220.65	8.06	33.03	53.27	13.37	*F. harpi*	N
1	3	13	4/8/2014	34.443901	-93.210743	71	100	0	26	0	170.66	6209.84	148.87	8.30	34.17	52.68	13		Y
1	3	14	4/8/2014	34.443879	-93.210636	5	97	0	34	0	170.66	6210.26	139.60	7.56	34.17	52.68	13		Y
1	3	15	4/8/2014	34.443857	-93.210529	1	100	0	36	0	170.65	6209.95	130.45	8.79	26.02	52.88	21.1		Y
1	3	16	4/8/2014	34.443835	-93.210422	0	97	1	0	1	170.65	6209.91	121.47	8.81	26.02	52.88	21.1	*F. harpi*	Y
1	3	17	4/8/2014	34.443813	-93.210315	0	96	1	10	0	170.66	6210.07	112.68	9.60	31.4	53.16	15.44		Y
1	3	18	4/8/2014	34.443791	-93.210209	30	97	1	9	0	170.50	6209.33	104.21	9.47	31.4	53.16	15.44		Y
2	4	19	4/8/2014	34.43239	-93.27458	0	25	1	9	0	178.64	6213.54	131.09	8.12	26.32	50.83	22.85		Y
2	4	20	4/8/2014	34.432372	-93.274688	0	22	1	9	0	178.42	6211.96	135.81	8.14	26.32	50.83	22.85		Y
2	4	21	4/8/2014	34.432355	-93.274796	0	96	0	23	0	178.08	6210.38	141.18	9.40	34.92	51	14.08		Y
2	4	22	4/8/2014	34.432337	-93.274904	0	82	1	32	0	177.73	6210.74	146.97	9.67	34.92	51	14.08		Y
2	4	23	4/8/2014	34.43232	-93.275012	0	95	1	28	1	177.04	6214.75	153.28	10.48	47.4	44.48	8.12	*F. harpi*	Y

Table C.1 cont'd

Site	Transect	Quadrat	Date	Latitude	Longitude	Canopy	Herb	Sedge	Stem	Burrow	Elevation	Solar	Water_dist	CTI	Sand	Silt	Clay	Burrow ownership	Water_trns
2	4	24	4/8/2014	34.432302	-93.275119	1	32	1	17	0	176.75	6213.06	159.82	12.71	47.4	44.48	8.12		Y
2	5	25	4/8/2014	34.43214	-93.27487	84	2	0	0	0	177.53	6209.28	130.73	7.84	26.46	57.32	16.22		N
2	5	26	4/8/2014	34.432123	-93.274978	3	1	0	0	0	177.12	6210.34	137.67	7.64	26.46	57.32	16.22		N
2	5	27	4/8/2014	34.432105	-93.275086	92	21	0	0	0	176.83	6209.64	144.93	7.34	53.44	38.24	8.32		N
2	5	28	4/8/2014	34.432087	-93.275194	97	3	0	0	0	176.56	6213.28	152.51	9.05	53.44	38.24	8.32		N
2	5	29	4/8/2014	34.43207	-93.275302	98	23	0	1	0	176.52	6214.63	160.42	12.38	43.09	45.6	10.92		N
2	5	30	4/8/2014	34.432052	-93.275409	100	7	1	0	0	176.63	6215.16	168.44	7.90	43.09	45.6	10.92		N
2	6	31	4/8/2014	34.43238	-93.2755	29	85	0	8	1	177.42	6217.43	169.37	8.01	44	43.68	12.32	*F. harpi*	Y
2	6	32	4/8/2014	34.432363	-93.275608	6	52	1	50	0	177.48	6216.95	162.50	8.92	44	43.68	12.32		Y
2	6	33	4/8/2014	34.432345	-93.275716	1	84	1	3	0	177.57	6215.81	155.84	8.40	43.67	46.69	9.64		Y
2	6	34	4/8/2014	34.432327	-93.275824	28	56	1	32	0	177.64	6214.15	149.18	8.72	43.67	46.69	9.64		Y
2	6	35	4/8/2014	34.43231	-93.275932	74	94	1	12	0	177.67	6212.30	142.71	8.02	40.8	46.37	12.48		Y
2	6	36	4/8/2014	34.432292	-93.276039	32	84	1	5	0	177.63	6211.04	136.82	9.41	40.8	46.37	12.48		Y
3	7	37	4/8/2014	34.427891	-93.293133	0	100	0	33	0	181.99	6217.31	181.44	7.60	46.85	41.14	12.01		N
3	7	38	4/8/2014	34.427913	-93.293024	0	100	0	30	0	182.08	6216.44	191.17	7.83	46.85	41.14	12.01		N
3	7	39	4/8/2014	34.427934	-93.292915	0	100	0	25	0	182.19	6215.59	184.09	7.94	41.78	45.85	12.37		N
3	7	40	4/8/2014	34.427956	-93.292806	0	93	0	22	0	182.26	6215.39	177.23	7.94	41.78	45.85	12.37		N
3	7	41	4/8/2014	34.427977	-93.292697	0	100	0	21	0	182.45	6216.07	170.82	7.85	41.5	47.53	10.97		N
3	7	42	4/8/2014	34.427999	-93.292588	0	100	0	26	0	182.54	6216.19	164.70	8.82	41.5	47.53	10.97		N
3	8	43	4/8/2014	34.42776	-93.29308	26	100	0	12	0	181.63	6211.70	179.84	8.78	40.81	50	9.19		Y
3	8	44	4/8/2014	34.427781	-93.292971	36	99	0	12	0	181.85	6214.96	190.01	8.01	40.81	50	9.19		Y
3	8	45	4/8/2014	34.427803	-93.292862	0	94	1	22	2	182.09	6215.04	194.72	8.59	29	58.82	12.18	*F. Harpi*	Y
3	8	46	4/8/2014	34.427824	-93.292753	0	86	0	5	0	182.18	6214.39	188.42	8.21	29	58.82	12.18		Y
3	8	47	4/8/2014	34.427846	-93.292644	0	88	1	20	3	182.27	6213.52	182.38	8.32	35.65	54.13	10.22	*F. Harpi*	Y
3	8	48	4/8/2014	34.427867	-93.292535	0	94	1	6	1	182.32	6212.88	176.84	11.49	35.65	54.13	10.22	*F. Harpi*	Y
3	9	49	4/8/2014	34.428028	-93.293175	72	69	1	14	0	182.14	6215.92	185.50	11.15	43.73	45.48	10.79		N

Table C.1 cont'd

Site	Transect	Quadrat	Date	Latitude	Longitude	Canopy	Herb	Sedge	Stem	Burrow	Elevation	Solar	Water_dist	CTI	Sand	Silt	Clay	Burrow ownership	Water_trns
3	9	50	4/8/2014	34.428	-93.293282	85	52	0	15	0	182.01	6216.40	175.52	11.86	43.73	45.48	10.79		N
3	9	51	4/8/2014	34.427972	-93.293389	98	66	1	3	0	181.85	6217.58	165.58	7.33	45.61	38.49	15.9		N
3	9	52	4/8/2014	34.427944	-93.293497	0	80	1	3	0	181.47	6216.37	155.61	7.34	45.61	38.49	15.9		N
3	9	53	4/8/2014	34.427916	-93.293604	91	90	0	10	0	181.43	6216.85	145.78	11.99	45.84	29.4	19.63		N
3	9	54	4/8/2014	34.427888	-93.293711	95	3	0	4	0	181.30	6218.55	136.02	7.19	45.84	29.4	19.63		N
3	10	55	4/8/2014	34.42746	-93.2953	0	77	0	19	0	176.32	6214.39	32.16	7.83	33.19	46.42	20.4		Y
3	10	56	4/8/2014	34.427429	-93.295405	0	96	1	34	0	176.30	6214.07	30.80	7.76	33.19	46.42	20.4		Y
3	10	57	4/8/2014	34.427399	-93.295511	0	93	1	36	0	175.72	6213.40	30.18	7.25	37.78	47.93	14.29		Y
3	10	58	4/8/2014	34.427368	-93.295616	0	95	1	17	0	175.64	6213.92	29.70	11.55	37.78	47.93	14.29		Y
3	10	59	4/8/2014	34.427338	-93.295722	0	97	1	19	0	175.58	6214.33	29.36	6.87	40.25	45.18	14.42		Y
3	10	60	4/8/2014	34.427307	-93.295827	0	89	0	22	0	175.07	6213.32	29.73	6.84	40.25	45.18	14.42		Y
4	11	61	4/9/2014	34.393746	-93.357599	8	90	1	18	0	214.37	6227.02	49.42	6.71	43.58	44.6	11.81		N
4	11	62	4/9/2014	34.393709	-93.357699	0	88	1	21	0	214.29	6228.65	52.85	6.73	43.58	44.6	11.81		N
4	11	63	4/9/2014	34.393673	-93.3578	1	88	1	11	0	213.92	6237.21	56.13	6.79	46.35	41.83	11.82		N
4	11	64	4/9/2014	34.393637	-93.3579	4	100	1	22	0	213.77	6236.50	57.93	9.01	46.35	41.83	11.82		N
4	11	65	4/9/2014	34.393601	-93.358001	0	100	1	33	0	213.52	6233.49	57.99	7.77	45.54	41.34	13.12		N
4	11	66	4/9/2014	34.393565	-93.358101	6	100	1	16	0	213.52	6232.51	58.09	9.98	45.54	41.34	13.12		N
4	12	67	4/9/2014	34.393711	-93.357577	1	84	0	4	0	214.48	6223.65	53.34	6.91	35.69	47.53	16.78		Y
4	12	68	4/9/2014	34.393675	-93.357678	5	96	1	7	0	214.37	6230.82	56.75	6.73	35.69	47.53	16.78		Y
4	12	69	4/9/2014	34.39364	-93.357778	2	96	1	3	0	213.92	6237.21	59.96	8.08	27.7	55.81	16.49		Y
4	12	70	4/9/2014	34.393604	-93.357879	1	10	0	0	0	213.51	6234.70	62.05	9.01	27.7	55.81	16.49		Y
4	12	71	4/9/2014	34.393568	-93.357979	3	80	1	4	0	213.52	6233.49	62.15	12.17	45.45	40.59	13.96		Y
4	12	72	4/9/2014	34.393532	-93.35808	24	68	1	6	0	213.52	6232.51	62.22	9.98	45.45	40.59	13.96		Y
4	13	73	4/9/2014	34.39364	-93.357531	98	3	0	0	0	214.67	6224.49	60.83	7.81	39.41	48.79	11.8		N
4	13	74	4/9/2014	34.393606	-93.357632	96	15	0	0	0	214.32	6226.25	64.69	6.54	39.41	48.79	11.8		N
4	13	75	4/9/2014	34.393571	-93.357734	97	3	0	1	0	213.83	6231.00	67.88	7.78	38.3	55.6	6.1		N
4	13	76	4/9/2014	34.393537	-93.357835	99	10	0	5	0	213.54	6234.55	70.39	12.18	38.3	55.6	6.1		N

Table C.1 cont'd

Site	Transect	Quadrat	Date	Latitude	Longitude	Canopy	Herb	Sedge	Stem	Burrow	Elevation	Solar	Water_dist	CTI	Sand	Silt	Clay	Burrow ownership	Water_trns
4	13	77	4/9/2014	34.393503	-93.357936	100	15	0	5	0	213.52	6230.65	70.33	12.17	41.3	46.31	12.39		N
4	13	78	4/9/2014	34.393469	-93.358038	96	35	0	2	0	213.52	6230.88	70.15	11.15	41.3	46.31	12.39		N
4	14	79	4/9/2014	34.39405	-93.35772	100	8	1	9	0	213.08	6201.24	15.00	6.75	31.6	55.12	13.27		Y
4	14	80	4/9/2014	34.394016	-93.357821	98	3	1	0	0	212.76	6202.52	18.09	6.83	31.6	55.12	13.27		Y
4	14	81	4/9/2014	34.393982	-93.357923	96	3	0	0	0	212.50	6204.66	20.56	6.90	25.76	55.96	18.28		Y
4	14	82	4/9/2014	34.393947	-93.358024	98	8	1	0	0	212.75	6201.97	21.83	6.54	25.76	55.96	18.28		Y
4	14	83	4/9/2014	34.393913	-93.358126	100	1	0	0	0	212.41	6199.01	21.65	6.53	40.07	49.53	9.83		Y
4	14	84	4/9/2014	34.393879	-93.358227	100	1	1	0	0	212.82	6193.76	21.51	9.65	40.07	49.53	9.83		Y
5	15	85	4/9/2014	34.33596	-93.51162	0	100	1	43	0	183.21	6167.98	432.27	7.20	36.81	44.37	18.82		N
5	15	86	4/9/2014	34.335998	-93.511521	0	100	0	31	0	183.77	6158.19	423.47	6.73	36.81	44.37	18.82		N
5	15	87	4/9/2014	34.336036	-93.511422	0	100	0	27	0	183.75	6170.86	414.72	7.40	32.81	41.86	25.33		N
5	15	88	4/9/2014	34.336074	-93.511323	0	100	1	34	0	184.33	6172.15	406.04	7.20	32.81	41.86	25.33		N
5	15	89	4/9/2014	34.336112	-93.511224	0	100	1	33	0	184.25	6179.89	397.42	12.18	39.27	45.86	14.87		N
5	15	90	4/9/2014	34.33615	-93.511124	0	100	0	20	0	184.75	6178.99	388.81	12.05	39.27	45.86	14.87		N
5	16	91	4/9/2014	34.33606	-93.51176	0	28	1	3	0	182.18	6198.48	431.30	8.17	16.6	56.1	27.3		Y
5	16	92	4/9/2014	34.336098	-93.511661	0	76	1	16	0	182.67	6184.76	422.39	7.56	16.6	56.1	27.3		Y
5	16	93	4/9/2014	34.336136	-93.511562	0	80	1	37	0	182.77	6189.36	413.51	12.35	20.49	57.79	21.72		Y
5	16	94	4/9/2014	34.336174	-93.511463	0	88	1	32	0	183.25	6180.86	404.62	12.15	20.49	57.79	21.72		Y
5	16	95	4/9/2014	34.336212	-93.511364	0	88	1	39	2	183.33	6184.74	395.79	6.18	14.12	53.03	32.85	*F. harpi*	Y
5	16	96	4/9/2014	34.33625	-93.511264	0	97	1	44	2	183.85	6186.10	386.97	6.24	14.12	53.03	32.85	*F. harpi*	Y
5	17	97	4/9/2014	34.33521	-93.51141	0	100	0	44	0	188.69	6193.52	395.74	5.79	38.87	43.1	17.95		N
5	17	98	4/9/2014	34.335248	-93.511311	0	98	0	26	0	189.39	6169.24	402.76	7.24	38.87	43.1	17.95		N
5	17	99	4/9/2014	34.335286	-93.511212	0	96	0	30	0	189.51	6181.50	399.62	6.29	30.32	58.05	11.63		N
5	17	100	4/9/2014	34.335324	-93.511113	0	100	0	27	0	190.80	6146.43	394.09	7.12	30.32	58.05	11.63		N
5	17	101	4/9/2014	34.335362	-93.511014	0	98	0	36	0	193.05	6130.11	388.74	6.35	37.47	43.46	18.78		N

Table C.1 cont'd

Site	Transect	Quadrat	Date	Latitude	Longitude	Canopy	Herb	Sedge	Stem	Burrow	Elevation	Solar	Water_dist	CTI	Sand	Silt	Clay	Burrow ownership	Water_trns
5	17	102	4/9/2014	34.3354	-93.510914	0	76	0	29	0	192.70	6140.15	383.50	6.50	37.47	43.46	18.78		N
6	18	103	4/9/2014	34.33268	-93.52367	0	92	1	0	0	170.71	6202.88	75.38	8.13	42.28	49.33	8.39		Y
6	18	104	4/9/2014	34.332713	-93.523568	0	20	1	8	0	170.69	6197.48	72.25	7.33	42.28	49.33	8.39		Y
6	18	105	4/9/2014	34.332745	-93.523466	0	28	1	7	0	170.68	6197.36	69.61	8.31	31.8	56.21	11.99		Y
6	18	106	4/9/2014	34.332778	-93.523364	0	16	1	0	0	170.43	6193.91	67.62	7.67	31.8	56.21	11.99		Y
6	18	107	4/9/2014	34.332811	-93.523262	0	100	1	19	0	170.51	6195.86	67.10	8.25	33.27	56.02	10.7		Y
6	18	108	4/9/2014	34.332844	-93.52316	0	92	1	12	0	170.59	6197.12	68.08	10.78	33.27	56.02	10.7		Y
6	19	109	4/9/2014	34.33297	-93.52381	0	2	1	3	1	169.97	6198.11	44.48	11.08	25.02	57.76	17.22	*F. harpi*	Y
6	19	110	4/9/2014	34.333003	-93.523708	0	2	1	0	0	170.02	6199.57	39.76	9.01	25.02	57.76	17.22		Y
6	19	111	4/9/2014	34.333035	-93.523606	0	60	1	4	0	170.09	6200.88	36.37	11.74	20.34	64.64	15.02		Y
6	19	112	4/9/2014	34.333068	-93.523504	0	16	1	4	0	170.05	6200.84	33.62	8.07	20.34	64.64	15.02		Y
6	19	113	4/9/2014	34.333101	-93.523402	0	40	1	4	0	170.06	6201.19	32.46	9.82	16.64	65.95	17.41		Y
6	19	114	4/9/2014	34.333134	-93.5233	0	100	1	22	0	170.15	6201.42	34.35	9.15	16.64	65.95	17.41		Y
6	20	115	4/9/2014	34.33226	-93.52361	0	100	0	28	1	170.15	6211.00	122.06	8.01	33.93	56.6	9.47	*F. harpi*	N
6	20	116	4/9/2014	34.332237	-93.523715	0	32	1	19	1	170.32	6211.58	118.35	7.89	33.93	56.6	9.47	*F. harpi*	N
6	20	117	4/9/2014	34.332214	-93.523821	0	96	0	9	1	170.14	6210.91	110.23	8.01	45.32	48.02	6.66	*F. harpi*	N
6	20	118	4/9/2014	34.332191	-93.523926	0	84	0	22	0	170.20	6211.34	102.23	8.82	45.32	48.02	6.66		N
6	20	119	4/9/2014	34.332168	-93.524032	0	38	1	4	0	170.28	6211.69	94.53	7.52	44.78	44.54	10.68		N
6	20	120	4/9/2014	34.332145	-93.524137	0	38	0	14	0	170.29	6212.02	87.36	7.89	44.78	44.54	10.68		N
7	21	121	4/9/2014	34.33539	-93.5418	92	40	1	3	1	186.43	6222.50	110.75	7.32	32.47	50.97	16.56	*F. harpi*	N
7	21	122	4/9/2014	34.335298	-93.541801	100	3	0	0	0	186.04	6222.62	101.65	7.64	32.47	50.97	16.56		N
7	21	123	4/9/2014	34.335206	-93.541803	100	1	0	0	0	185.70	6221.54	92.82	8.68	33.76	52.09	14.14		N
7	21	124	4/9/2014	34.335114	-93.541804	99	1	1	3	0	185.47	6221.48	84.24	7.46	33.76	52.09	14.14		N
7	21	125	4/9/2014	34.335022	-93.541805	100	1	0	1	0	185.18	6221.79	76.07	8.33	32.77	51.86	15.37		N
7	21	126	4/9/2014	34.33493	-93.541806	100	1	0	0	0	184.89	6222.16	68.45	7.35	32.77	51.86	15.37		N
7	22	127	4/9/2014	34.33554	-93.54141	98	3	0	0	1	187.39	6219.12	115.74	9.19	38.83	49.76	11.41	*F. harpi*	N
7	22	128	4/9/2014	34.335448	-93.541411	96	84	1	8	0	187.15	6221.01	105.75	6.81	38.83	49.76	11.41		N

Table C.1 cont'd

Site	Transect	Quadrat	Date	Latitude	Longitude	Canopy	Herb	Sedge	Stem	Burrow	Elevation	Solar	Water_dist	CTI	Sand	Silt	Clay	Burrow ownership	Water_trns
7	22	129	4/9/2014	34.335356	-93.541413	95	94	0	11	0	186.75	6222.62	95.82	7.15	36.19	54.03	9.78		N
7	22	130	4/9/2014	34.335264	-93.541414	99	28	0	1	0	186.31	6223.44	85.94	7.69	36.19	54.03	9.78		N
7	22	131	4/9/2014	34.335172	-93.541415	100	28	0	3	0	185.88	6224.29	76.14	7.94	39.14	50.59	10.27		N
7	22	132	4/9/2014	34.33508	-93.541417	96	12	0	0	0	185.37	6224.57	66.45	10.53	39.14	50.59	10.27		N
7	23	133	4/9/2014	34.33562	-93.54108	7	100	0	24	0	188.81	6217.87	108.60	6.36	34.23	50.43	15.34		N
7	23	134	4/9/2014	34.335528	-93.541081	30	90	1	26	1	188.67	6219.66	112.51	7.73	34.23	50.43	15.34	*F. harpi*	N
7	23	135	4/9/2014	34.335436	-93.541083	67	95	1	13	0	188.43	6222.29	102.31	6.31	32.64	47.67	19.69		N
7	23	136	4/9/2014	34.335344	-93.541084	97	85	1	2	1	188.01	6223.87	92.11	6.83	32.64	47.67	19.69	*F. harpi*	N
7	23	137	4/9/2014	34.335252	-93.541085	96	1	0	0	0	187.42	6223.32	81.90	7.08	27.98	51.44	20.59		N
7	23	138	4/9/2014	34.33516	-93.541086	90	1	0	0	0	186.56	6222.22	71.70	5.75	27.98	51.44	20.59		N
8	24	139	4/9/2014	34.33228	-93.54092	0	90	1	9	0	178.19	6215.64	220.47	7.68	36.03	48.51	15.46		N
8	24	140	4/9/2014	34.332371	-93.540924	0	90	1	22	4	178.39	6216.04	210.39	12.53	36.03	48.51	15.46	*F. harpi*	N
8	24	141	4/9/2014	34.332461	-93.540928	0	85	1	3	4	178.61	6216.34	200.41	8.69	29.65	57.6	12.75	*F. harpi*	N
8	24	142	4/9/2014	34.332552	-93.540932	0	80	1	4	2	178.86	6216.77	190.32	8.49	29.65	57.6	12.75	*F. harpi*	N
8	24	143	4/9/2014	34.332642	-93.540935	0	90	1	6	1	179.06	6216.22	180.36	8.30	29	56.65	14.35	*F. harpi*	N
8	24	144	4/9/2014	34.332733	-93.540939	0	99	1	32	3	179.23	6216.57	170.27	7.95	29	56.65	14.35	*F. harpi*	N
8	25	145	4/9/2014	34.33235	-93.54105	0	99	1	11	2	178.49	6215.51	212.10	12.59	27.14	47.94	24.85	*F. harpi*	N
8	25	146	4/9/2014	34.332441	-93.541054	0	80	1	28	4	178.66	6216.22	202.01	9.63	27.14	47.94	24.85	*F. harpi*	N
8	25	147	4/9/2014	34.332531	-93.541058	0	100	1	25	4	178.90	6216.49	192.02	8.53	37.1	54.46	8.44	*F. harpi*	N
8	25	148	4/9/2014	34.332622	-93.541062	0	90	0	33	8	179.07	6215.93	181.93	8.40	37.1	54.46	8.44	*F. harpi*	N
8	25	149	4/9/2014	34.332712	-93.541065	0	95	1	24	2	179.25	6216.77	171.94	7.84	30.28	58.29	11.43	*F. harpi*	N
8	25	150	4/9/2014	34.332803	-93.541069	0	95	0	11	4	179.50	6216.47	161.85	7.64	30.28	58.29	11.43	*F. harpi*	N
9	26	151	4/10/2014	34.34728	-93.48791	0	76	1	11	0	185.40	6220.87	143.32	6.62	27.34	54.77	17.89		N
9	26	152	4/10/2014	34.347333	-93.48782	1	34	1	27	0	185.65	6221.29	143.99	6.76	27.34	54.77	17.89		N
9	26	153	4/10/2014	34.347386	-93.487729	8	64	1	17	0	185.54	6221.09	138.38	6.54	39.22	46.4	14.38		N
9	26	154	4/10/2014	34.347438	-93.487639	1	55	1	6	0	185.87	6220.05	133.20	9.77	39.22	46.4	14.38		N
9	26	155	4/10/2014	34.347491	-93.487549	0	55	1	22	0	185.68	6221.11	128.58	10.37	43.38	44.77	11.85		N

Table C.1 cont'd

Site	Transect	Quadrat	Date	Latitude	Longitude	Canopy	Herb	Sedge	Stem	Burrow	Elevation	Solar	Water_dist	CTI	Sand	Silt	Clay	Burrow ownership	Water_trns
9	26	156	4/10/2014	34.347544	-93.487459	1	3	0	24	0	186.14	6219.99	124.02	7.38	43.38	44.77	11.85		N
9	27	157	4/10/2014	34.346806	-93.487963	8	77	1	27	2	183.47	6218.94	99.37	7.45	30.29	45.02	24.69	*F. harpi*	N
9	27	158	4/10/2014	34.346859	-93.487872	3	97	1	31	0	183.79	6218.92	99.73	7.45	30.29	45.02	24.69		N
9	27	159	4/10/2014	34.346912	-93.487782	1	98	1	27	1	183.78	6218.57	101.16	10.54	36.84	46.9	16.26	*F. harpi*	N
9	27	160	4/10/2014	34.346965	-93.487692	6	81	1	19	7	184.09	6218.65	103.58	8.92	36.84	46.9	16.26	*F. harpi*	N
9	27	161	4/10/2014	34.347017	-93.487601	12	76	0	6	4	184.01	6217.83	106.39	8.62	43.79	46.01	10.2	*F. harpi*	N
9	27	162	4/10/2014	34.34707	-93.487511	13	90	0	21	0	184.23	6218.33	106.95	8.35	43.79	46.01	10.2		N
9	28	163	4/10/2014	34.346718	-93.488097	1	95	1	35	0	183.11	6218.19	99.21	7.23	41.21	44.65	14.14		N
9	28	164	4/10/2014	34.346665	-93.488186	0	90	1	17	0	182.97	6216.82	100.17	7.40	41.21	44.65	14.14		N
9	28	165	4/10/2014	34.346612	-93.488275	0	95	0	20	0	183.22	6216.90	97.96	9.20	40.76	47.5	11.73		N
9	28	166	4/10/2014	34.346559	-93.488363	0	95	1	29	0	183.09	6216.91	96.71	9.20	40.76	47.5	11.73		N
9	28	167	4/10/2014	34.346506	-93.488452	8	90	1	19	1	183.35	6216.91	96.23	8.16	29.19	45.57	25.24	*F. harpi*	N
9	28	168	4/10/2014	34.346453	-93.488541	32	74	0	25	0	183.20	6217.05	96.74	7.94	29.19	45.57	25.24		N
10	29	169	4/10/2014	34.35593	-93.47363	0	79	1	40	0	200.31	6227.26	119.84	8.12	18.86	46.51	34.57		Y
10	29	170	4/10/2014	34.355975	-93.473534	0	76	1	45	2	200.56	6223.26	117.98	8.22	18.86	46.51	34.57	*F. harpi*	Y
10	29	171	4/10/2014	34.35602	-93.473437	0	72	1	51	1	200.64	6221.10	116.93	7.81	40.82	44.82	14.36	*F. harpi*	Y
10	29	172	4/10/2014	34.356065	-93.473341	26	75	1	38	0	200.70	6217.43	116.80	8.42	40.82	44.82	14.36		Y
10	29	173	4/10/2014	34.35611	-93.473245	0	15	1	3	1	200.79	6214.29	117.56	8.58	39.37	40.85	19.78	*F. harpi*	Y
10	29	174	4/10/2014	34.356155	-93.473149	0	83	1	15	1	200.76	6216.73	119.01	7.22	39.37	40.85	19.78	*F. harpi*	Y
10	30	175	4/10/2014	34.35617	-93.47311	0	79	1	47	0	200.84	6213.47	119.44	8.73	50.82	37.56	11.56		Y
10	30	176	4/10/2014	34.356215	-93.473014	0	53	1	27	4	200.76	6218.14	121.91	10.68	50.82	37.56	11.56	*F. harpi*	Y
10	30	177	4/10/2014	34.35626	-93.472917	0	65	1	20	6	200.90	6217.26	125.14	10.30	44.15	47.95	7.9	*F. harpi*	Y
10	30	178	4/10/2014	34.356305	-93.472821	0	53	0	55	1	201.17	6222.77	129.09	7.07	44.15	47.95	7.9	*F. harpi*	Y
10	30	179	4/10/2014	34.35635	-93.472725	0	80	1	45	1	201.43	6219.79	129.38	6.88	36.31	43.63	20.05	*F. harpi*	Y
10	30	180	4/10/2014	34.356395	-93.472629	0	74	1	46	4	201.43	6207.14	122.90	11.91	36.31	43.63	20.05	*F. harpi*	Y
10	31	181	4/10/2014	34.35578	-93.47449	0	20	1	38	0	199.55	6231.24	146.61	7.34	36.57	46.45	16.98		Y
10	31	182	4/10/2014	34.355728	-93.474581	0	80	1	19	0	199.40	6230.16	140.66	7.29	36.57	46.45	16.98		Y

Table C.1 cont'd

Site	Transect	Quadrat	Date	Latitude	Longitude	Canopy	Herb	Sedge	Stem	Burrow	Elevation	Solar	Water_dist	CTI	Sand	Silt	Clay	Burrow ownership	Water_trns
10	31	183	4/10/2014	34.355675	-93.474671	0	69	1	13	0	199.64	6230.65	135.33	7.69	41.1	47.45	11.45		Y
10	31	184	4/10/2014	34.355623	-93.474762	0	95	1	66	0	199.53	6230.52	130.45	8.01	41.1	47.45	11.45		Y
10	31	185	4/10/2014	34.355571	-93.474852	0	90	0	30	1	199.77	6229.81	126.28	7.68	27.28	61.34	11.38	*F. harpi*	Y
10	31	186	4/10/2014	34.355519	-93.474943	0	85	1	12	1	199.78	6232.06	122.72	6.54	27.28	61.34	11.38	*F. harpi*	Y
11	32	187	4/10/2014	34.373446	-93.404412	97	90	0	31	0	186.94	6220.37	83.71	10.22	41.93	47.71	10.37		N
11	32	188	4/10/2014	34.373394	-93.404502	26	89	1	20	0	186.85	6219.65	85.36	6.47	41.93	47.71	10.37		N
11	32	189	4/10/2014	34.373341	-93.404592	97	40	1	3	0	187.42	6206.59	88.26	5.57	48.16	37.4	14.44		N
11	32	190	4/10/2014	34.373289	-93.404683	24	20	1	7	0	188.16	6201.65	92.06	5.89	48.16	37.4	14.44		N
11	32	191	4/10/2014	34.373236	-93.404773	97	55	1	15	0	187.95	6208.07	96.90	6.28	41.54	48.38	10.08		N
11	32	192	4/10/2014	34.373183	-93.404863	32	3	0	0	0	188.66	6220.47	102.52	6.72	41.54	48.38	10.08		N
11	33	193	4/10/2014	34.373349	-93.404349	95	73	1	12	0	186.62	6222.02	95.92	7.44	52.2	42.68	5.1		N
11	33	194	4/10/2014	34.373296	-93.404439	92	100	1	38	0	186.31	6222.05	97.62	9.97	52.2	42.68	5.1		N
11	33	195	4/10/2014	34.373244	-93.404529	84	45	1	12	0	186.34	6220.83	100.21	8.15	42.73	44.41	12.86		N
11	33	196	4/10/2014	34.373191	-93.404619	97	3	1	2	0	186.10	6212.33	103.83	6.77	42.73	44.41	12.86		N
11	33	197	4/10/2014	34.373139	-93.404709	99	20	0	0	0	186.60	6188.38	108.18	8.15	31.94	50.28	17.77		N
11	33	198	4/10/2014	34.373086	-93.404799	95	35	0	0	0	187.60	6195.96	113.37	8.26	31.94	50.28	17.77		N
11	34	199	4/10/2014	34.37373	-93.40442	13	75	1	3	0	187.70	6223.65	57.11	11.41	32.63	55.63	11.74		N
11	34	200	4/10/2014	34.373683	-93.404513	58	100	1	68	0	187.48	6221.14	56.39	11.31	32.63	55.63	11.74		N
11	34	201	4/10/2014	34.373635	-93.404607	100	15	1	0	0	187.64	6219.12	56.98	9.42	39.32	46.19	14.49		N
11	34	202	4/10/2014	34.373588	-93.4047	100	0	0	0	0	187.90	6208.41	59.25	9.88	39.32	46.19	14.49		N
11	34	203	4/10/2014	34.37354	-93.404793	51	40	0	12	0	188.15	6204.61	63.15	6.82	37.41	52.27	10.32		N
11	34	204	4/10/2014	34.373493	-93.404886	0	95	0	68	0	188.76	6198.55	68.21	8.72	37.41	52.27	10.32		N
11	35	205	4/10/2014	34.374093	-93.404119	0	99	1	53	5	188.30	6223.63	40.05	11.24	31.75	50.31	17.94	*F. harpi*	N
11	35	206	4/10/2014	34.374041	-93.404209	0	90	1	22	6	188.16	6224.43	40.24	8.58	31.75	50.31	17.94	*F. harpi*	N
11	35	207	4/10/2014	34.373988	-93.4043	0	81	1	61	4	188.60	6225.94	42.37	9.32	51.91	33.3	14.79	*F. harpi*	N
11	35	208	4/10/2014	34.373935	-93.40439	0	90	1	80	3	189.14	6226.69	46.59	9.16	51.91	33.3	14.79	*F. harpi*	N
11	35	209	4/10/2014	34.373883	-93.40448	0	100	1	26	2	189.11	6226.79	52.29	7.19	14.74	55.11	30.15	*F. harpi*	N

Table C.1 cont'd

Site	Transect	Quadrat	Date	Latitude	Longitude	Canopy	Herb	Sedge	Stem	Burrow	Elevation	Solar	Water_dist	CTI	Sand	Silt	Clay	Burrow ownership	Water_trns
11	35	210	4/10/2014	34.37383	-93.40457	0	100	0	33	1	189.70	6227.28	58.91	6.77	14.74	55.11	30.15	*F. harpi*	N
3	3	432	3/29/2015	34.25165	-93.6528	28	55	0	22	0	187.99	6003.80	179.58	6.60	23.25	58.88	17.87		
3	3	433	3/29/2015	34.25165	-93.6528	76	80	0	12	0	187.50	6005.23	170.75	8.66	23.25	58.88	17.87		
3	3	434	3/29/2015	34.25165	-93.6528	11	90	0	9	0	187.27	6008.10	161.98	9.07	23.25	58.88	17.87		
3	3	435	3/29/2015	34.25165	-93.6528	11	90	0	21	0	187.05	6008.75	153.36	13.52	23.25	58.88	17.87		
3	3	436	3/29/2015	34.25165	-93.6528	31	55	0	37	0	186.94	6009.33	145.00	6.89	23.25	58.88	17.87		
3	3	437	3/29/2015	34.25165	-93.6528	40	85	0	42	0	186.78	6009.73	136.83	8.99	23.25	58.88	17.87		
6	6	438	3/26/2015	34.37954	-93.29277	0	80	0	17	0	183.82	6002.75	131.36	10.56	17.95	62.35	19.70		
6	6	439	3/26/2015	34.37954	-93.29277	1	200	1	20	0	183.90	6002.51	136.38	8.33	17.95	62.35	19.70		
6	6	440	3/26/2015	34.37954	-93.29277	5	98	0	27	0	183.88	6001.79	141.90	8.49	17.95	62.35	19.70		
6	6	441	3/26/2015	34.37954	-93.29277	2	95	0	12	0	183.83	6000.97	147.80	8.42	17.95	62.35	19.70		
6	6	442	3/26/2015	34.37954	-93.29277	0	100	0	58	0	183.82	6001.96	141.19	8.82	17.95	62.35	19.70		
6	6	443	3/26/2015	34.37954	-93.29277	0	100	0	62	0	183.71	6000.30	133.67	7.14	17.95	62.35	19.70		
8	8	444	3/26/2015	34.32465	-93.173	0	95	0	39	0	185.93	6015.41	200.60	8.03	23.63	62.74	13.63		
8	8	445	3/26/2015	34.32465	-93.173	0	90	0	58	0	186.63	6016.66	195.39	8.01	23.63	62.74	13.63		
8	8	446	3/26/2015	34.32465	-93.173	0	70	0	46	0	187.33	6017.00	190.63	7.87	23.63	62.74	13.63		
8	8	447	3/26/2015	34.32465	-93.173	0	40	0	7	0	188.00	6017.15	186.21	7.72	23.63	62.74	13.63		
8	8	448	3/26/2015	34.32465	-93.173	0	55	0	46	0	188.64	6015.19	182.31	7.21	23.63	62.74	13.63		
8	8	449	3/26/2015	34.32465	-93.173	0	28	0	39	0	189.77	6018.45	178.75	6.99	23.63	62.74	13.63		
9	9	450	3/23/2015	34.7494	-92.82497	0	35	0	12	0	193.82	6012.11	154.52	9.11	18.28	61.07	20.65		
9	9	451	3/23/2015	34.7494	-92.82497	0	15	1	5	0	194.01	6012.26	164.36	8.93	18.28	61.07	20.65		
9	9	452	3/23/2015	34.7494	-92.82497	0	85	1	18	0	194.03	6011.79	174.29	11.22	18.28	61.07	20.65		
9	9	453	3/23/2015	34.7494	-92.82497	0	85	1	13	0	193.99	6011.46	184.24	12.06	18.28	61.07	20.65		
9	9	454	3/23/2015	34.7494	-92.82497	0	90	1	20	0	194.06	6010.29	194.11	9.92	18.28	61.07	20.65		
9	9	455	3/23/2015	34.7494	-92.82497	0	2	0	0	0	194.16	6009.09	204.02	9.59	18.28	61.07	20.65		
10	10	456	3/31/2015	34.56465	-93.63181	0	65	0	13	0	194.15	6009.33	129.30	10.94	11.81	76.57	11.62		
10	10	457	3/31/2015	34.56465	-93.63181	0	80	1	22	0	194.15	6008.07	137.54	10.71	11.81	76.57	11.62		

Table C.1 cont'd

Site	Transect	Quadrat	Date	Latitude	Longitude	Canopy	Herb	Sedge	Stem	Burrow	Elevation	Solar	Water_dist	CTI	Sand	Silt	Clay	Burrow ownership	Water_trns
10	10	458	3/31/2015	34.56465	-93.63181	0	35	1	36	0	194.31	6007.26	145.87	9.96	11.81	76.57	11.62		
10	10	459	3/31/2015	34.56465	-93.63181	0	65	1	37	0	194.33	6008.90	154.11	7.66	11.81	76.57	11.62		
10	10	460	3/31/2015	34.56465	-93.63181	0	70	1	24	0	194.33	6008.38	162.44	10.81	11.81	76.57	11.62		
10	10	461	3/31/2015	34.56465	-93.63181	0	75	1	27	0	194.37	6007.62	170.41	7.75	11.81	76.57	11.62		
12	12	462	3/25/2015	34.6647	-93.26058	0	100	0	49	1	192.06	6009.98	162.73	13.40	21.59	60.33	18.08		
12	12	463	3/25/2015	34.6647	-93.26058	0	95	0	36	0	192.03	6011.62	172.32	13.60	21.59	60.33	18.08		
12	12	464	3/25/2015	34.6647	-93.26058	12	96	0	48	0	192.04	6011.15	182.03	7.08	21.59	60.33	18.08		
12	12	465	3/25/2015	34.6647	-93.26058	20	90	0	12	0	192.04	6010.90	191.76	7.08	21.59	60.33	18.08		
12	12	466	3/25/2015	34.6647	-93.26058	0	60	1	0	0	191.52	6013.75	201.45	7.12	21.59	60.33	18.08		
12	12	467	3/25/2015	34.6647	-93.26058	8	55	1	22	0	191.60	6013.10	211.25	7.55	21.59	60.33	18.08		
13	13	468	3/29/2015	34.2505	-93.42452	0	80	0	68	0	174.75	6004.43	189.01	6.15	38.93	50.66	10.41		
13	13	469	3/29/2015	34.2505	-93.42452	0	100	0	94	0	174.43	6001.88	198.76	6.69	38.93	50.66	10.41		
13	13	470	3/29/2015	34.2505	-93.42452	0	95	0	78	0	174.08	6000.30	208.51	7.13	38.93	50.66	10.41		
13	13	471	3/29/2015	34.2505	-93.42452	0	90	0	22	0	173.67	5996.38	218.27	7.61	38.93	50.66	10.41		
13	13	472	3/29/2015	34.2505	-93.42452	0	80	0	27	0	173.18	6001.12	228.02	11.69	38.93	50.66	10.41		
13	13	473	3/29/2015	34.2505	-93.42452	27	85	0	32	0	173.18	6002.90	237.77	8.09	38.93	50.66	10.41		
14	14	474	3/28/2015	34.32955	-93.53259	0	20	1	6	16	177.71	5995.32	245.37	8.95	31.68	53.11	15.21	*F. harpi*	
14	14	475	3/28/2015	34.32955	-93.53259	0	65	1	26	7	177.81	5995.34	237.11	7.77	31.68	53.11	15.21	*F. harpi*	
14	14	476	3/28/2015	34.32955	-93.53259	0	30	1	21	5	177.79	5995.49	228.99	7.55	31.68	53.11	15.21	*F. harpi*	
14	14	477	3/28/2015	34.32955	-93.53259	0	65	1	12	4	177.79	5995.49	220.93	7.55	31.68	53.11	15.21	*F. harpi*	
14	14	478	3/28/2015	34.32955	-93.53259	0	60	1	26	4	177.63	5997.67	212.93	7.53	31.68	53.11	15.21	*F. harpi*	
14	14	479	3/28/2015	34.32955	-93.53259	0	80	0	38	0	177.49	5998.54	205.01	9.73	31.68	53.11	15.21		
16	16	480	3/28/2015	34.44565	-93.34284	0	90	1	32	2	170.72	5994.74	131.09	10.31	24.82	56.49	18.69	*F. harpi*	
16	16	481	3/28/2015	34.44565	-93.34284	0	55	1	25	1	170.72	5994.71	136.74	15.02	24.82	56.49	18.69	*F. harpi*	
16	16	482	3/28/2015	34.44565	-93.34284	0	85	1	17	1	170.72	5996.95	140.30	8.37	24.82	56.49	18.69	*F. harpi*	
16	16	483	3/28/2015	34.44565	-93.34284	0	60	1	37	0	170.56	6000.24	144.46	9.51	24.82	56.49	18.69		
16	16	484	3/28/2015	34.44565	-93.34284	0	85	1	42	0	170.32	6001.65	142.66	9.25	24.82	56.49	18.69		

Table C.1 cont'd

Site	Transect	Quadrat	Date	Latitude	Longitude	Canopy	Herb	Sedge	Stem	Burrow	Elevation	Solar	Water_dist	CTI	Sand	Silt	Clay	Burrow ownership	Water_trns
16	16	485	3/28/2015	34.44565	-93.34284	0	55	0	42	0	170.01	6000.81	132.58	9.71	24.82	56.49	18.69		
18	18	486	3/29/2015	34.32352	-93.4558	0	60	0	21	7	188.13	6011.20	231.83	8.65	41.95	50.37	7.68	*F. harpi*	
18	18	487	3/29/2015	34.32352	-93.4558	0	55	1	16	5	187.92	6010.71	237.99	9.00	41.95	50.37	7.68	*F. harpi*	
18	18	488	3/29/2015	34.32352	-93.4558	0	35	1	27	4	187.85	6009.94	244.46	9.20	41.95	50.37	7.68	*F. harpi*	
18	18	489	3/29/2015	34.32352	-93.4558	0	55	1	23	4	187.65	6009.79	250.89	9.98	41.95	50.37	7.68	*F. harpi*	
18	18	490	3/29/2015	34.32352	-93.4558	0	35	1	17	1	187.55	6007.77	257.45	9.30	41.95	50.37	7.68	*F. harpi*	
18	18	491	3/29/2015	34.32352	-93.4558	0	45	1	12	1	187.49	6005.81	264.22	12.31	41.95	50.37	7.68	*F. harpi*	
24	24	492	3/28/2015	34.27573	-93.59502	8	100	0	64	0	190.22	6009.58	105.59	9.46	38.60	48.15	13.25		
24	24	493	3/28/2015	34.27573	-93.59502	80	25	0	7	0	190.23	6009.88	109.56	7.97	38.60	48.15	13.25		
24	24	494	3/28/2015	34.27573	-93.59502	50	15	0	0	0	190.03	6010.27	113.91	7.91	38.60	48.15	13.25		
24	24	495	3/28/2015	34.27573	-93.59502	84	65	0	3	0	189.95	6010.44	118.97	9.23	38.60	48.15	13.25		
24	24	496	3/28/2015	34.27573	-93.59502	90	65	0	22	0	189.91	6011.05	124.60	7.78	38.60	48.15	13.25		
24	24	497	3/28/2015	34.27573	-93.59502	8	70	0	19	0	189.29	6010.37	130.77	9.79	38.60	48.15	13.25		
25	25	498	4/1/2015	34.13671	-93.94329	2	78	1	37	5	180.93	6003.87	408.06	10.68	39.95	54.66	5.38		
25	25	499	4/1/2015	34.13671	-93.94329	3	85	1	22	2	181.13	6005.99	410.18	6.96	39.95	54.66	5.38		
25	25	500	4/1/2015	34.13671	-93.94329	2	65	1	27	1	181.53	6005.03	412.53	7.13	39.95	54.66	5.38		
25	25	501	4/1/2015	34.13671	-93.94329	4	85	1	41	1	181.56	6005.15	415.10	11.66	39.95	54.66	5.38		
25	25	502	4/1/2015	34.13671	-93.94329	1	77	1	32	0	181.62	6005.86	417.81	9.78	39.95	54.66	5.38		
25	25	503	4/1/2015	34.13671	-93.94329	0	45	1	13	0	181.70	6006.36	420.84	9.17	39.95	54.66	5.38		
27	27	504	3/29/2015	34.39679	-93.6251	0	90	0	27	0	185.00	5992.95	261.89	8.83	20.61	55.36	24.03		
27	27	505	3/29/2015	34.39679	-93.6251	0	70	1	12	0	184.90	5992.64	251.89	9.18	20.61	55.36	24.03		
27	27	506	3/29/2015	34.39679	-93.6251	0	80	1	32	0	184.75	5992.00	241.96	9.20	20.61	55.36	24.03		
27	27	507	3/29/2015	34.39679	-93.6251	0	90	1	47	0	184.59	5990.83	231.96	8.90	20.61	55.36	24.03		
27	27	508	3/29/2015	34.39679	-93.6251	0	80	1	33	0	184.26	5989.71	222.04	8.82	20.61	55.36	24.03		
27	27	509	3/29/2015	34.39679	-93.6251	0	80	1	52	0	183.97	5991.00	212.05	9.40	20.61	55.36	24.03		
29	29	510	3/28/2015	34.3375	-93.49864	0	100	0	84	0	180.04	5999.59	48.91	7.94	41.95	50.37	7.68		
29	29	511	3/28/2015	34.3375	-93.49864	0	100	0	114	0	179.79	5998.09	47.16	8.17	41.95	50.37	7.68		

Table C.1 cont'd

Site	Transect	Quadrat	Date	Latitude	Longitude	Canopy	Herb	Sedge	Stem	Burrow	Elevation	Solar	Water_dist	CTI	Sand	Silt	Clay	Burrow ownership	Water_trns
29	29	512	3/28/2015	34.3375	-93.49864	0	99	0	100	0	179.55	5997.61	45.79	8.00	41.95	50.37	7.68		
29	29	513	3/28/2015	34.3375	-93.49864	0	100	0	106	0	179.21	5998.48	44.55	8.12	41.95	50.37	7.68		
29	29	514	3/28/2015	34.3375	-93.49864	0	100	0	120	0	178.79	6002.26	44.70	11.84	41.95	50.37	7.68		
29	29	515	3/28/2015	34.3375	-93.49864	0	100	0	99	0	178.72	6006.06	45.48	6.97	41.95	50.37	7.68		
30	30	516	3/30/2015	34.80357	-93.9707	0	90	1	38	0	189.86	6005.88	132.03	8.28	31.03	58.33	10.64		
30	30	517	3/30/2015	34.80357	-93.9707	0	85	0	32	0	189.92	6005.81	140.26	8.68	31.03	58.33	10.64		
30	30	518	3/30/2015	34.80357	-93.9707	0	85	0	25	0	189.99	6006.18	147.66	9.13	31.03	58.33	10.64		
30	30	519	3/30/2015	34.80357	-93.9707	0	70	0	35	0	189.99	6006.92	154.95	8.93	31.03	58.33	10.64		
30	30	520	3/30/2015	34.80357	-93.9707	0	80	0	7	0	189.99	6007.23	160.65	8.72	31.03	58.33	10.64		
30	30	521	3/30/2015	34.80357	-93.9707	0	45	0	0	0	189.99	6007.22	166.64	8.58	31.03	58.33	10.64		
32	32	522	3/26/2015	34.32851	-93.07111	0	90	1	47	7	178.30	6004.16	36.17	8.36	37.96	54.17	7.87		
32	32	523	3/26/2015	34.32851	-93.07111	0	95	1	37	7	177.91	6004.64	45.71	8.39	37.96	54.17	7.87		
32	32	524	3/26/2015	34.32851	-93.07111	0	88	1	39	3	177.80	6008.25	55.30	11.43	37.96	54.17	7.87		
32	32	525	3/26/2015	34.32851	-93.07111	0	75	1	12	3	177.77	6009.15	65.11	8.75	37.96	54.17	7.87		
32	32	526	3/26/2015	34.32851	-93.07111	16	65	1	27	3	177.87	6009.57	74.96	9.31	37.96	54.17	7.87		
32	32	527	3/26/2015	34.32851	-93.07111	50	65	1	7	3	178.04	6009.18	84.86	7.73	37.96	54.17	7.87		
34	34	528	3/25/2015	34.53791	-93.23159	0	100	0	48	0	189.71	6007.03	146.87	8.46	29.70	56.02	14.28		
34	34	529	3/25/2015	34.53791	-93.23159	0	100	0	57	0	189.64	6005.51	141.29	8.30	29.70	56.02	14.28		
34	34	530	3/25/2015	34.53791	-93.23159	0	100	0	82	0	189.64	6004.94	136.31	9.01	29.70	56.02	14.28		
34	34	531	3/25/2015	34.53791	-93.23159	0	100	0	46	0	189.61	6007.42	131.89	8.16	29.70	56.02	14.28		
34	34	532	3/25/2015	34.53791	-93.23159	0	100	0	42	0	189.47	6009.58	127.65	8.63	29.70	56.02	14.28		
34	34	533	3/25/2015	34.53791	-93.23159	0	90	0	36	0	189.28	6010.34	123.86	8.94	29.70	56.02	14.28		
36	36	534	4/1/2015	34.1408	-93.89461	0	70	0	35	0	179.92	6005.58	241.73	8.34	69.01	27.73	3.26		
36	36	535	4/1/2015	34.1408	-93.89461	0	65	0	52	0	179.83	6004.61	249.10	10.18	69.01	27.73	3.26		
36	36	536	4/1/2015	34.1408	-93.89461	0	70	0	56	0	179.77	6004.87	256.78	7.38	69.01	27.73	3.26		
36	36	537	4/1/2015	34.1408	-93.89461	0	80	0	79	0	179.68	6005.49	264.48	9.59	69.01	27.73	3.26		
36	36	538	4/1/2015	34.1408	-93.89461	0	85	0	42	0	179.72	6005.76	272.47	9.71	69.01	27.73	3.26		

Table C.1 cont'd

Site	Transect	Quadrat	Date	Latitude	Longitude	Canopy	Herb	Sedge	Stem	Burrow	Elevation	Solar	Water_dist	CTI	Sand	Silt	Clay	Burrow ownership	Water_trns
36	36	539	4/1/2015	34.1408	-93.89461	0	30	0	0	0	179.61	6005.54	279.28	9.67	69.01	27.73	3.26		
37	37	540	3/27/2015	34.10069	-93.57496	0	99	0	27	0	177.12	6004.02	116.42	8.62	31.02	57.97	11.01		
37	37	541	3/27/2015	34.10069	-93.57496	0	100	0	29	0	177.08	6003.55	111.25	9.05	31.02	57.97	11.01		
37	37	542	3/27/2015	34.10069	-93.57496	0	95	1	17	0	177.07	6003.41	106.75	9.49	31.02	57.97	11.01		
37	37	543	3/27/2015	34.10069	-93.57496	0	90	1	22	0	177.05	6003.45	102.25	9.41	31.02	57.97	11.01		
37	37	544	3/27/2015	34.10069	-93.57496	0	99	0	32	0	177.03	6002.99	97.79	9.92	31.02	57.97	11.01		
37	37	545	3/27/2015	34.10069	-93.57496	0	90	1	25	0	177.03	6002.37	94.02	10.67	31.02	57.97	11.01		
38	38	546	4/3/2015	34.14672	-94.30036	100	46	0	36	0	190.52	6011.67	329.66	8.54	35.53	54.25	10.22		
38	38	547	4/3/2015	34.14672	-94.30036	5	80	0	17	0	190.39	6012.11	332.47	7.34	35.53	54.25	10.22		
38	38	548	4/3/2015	34.14672	-94.30036	8	85	0	17	0	190.24	6012.19	335.31	8.75	35.53	54.25	10.22		
38	38	549	4/3/2015	34.14672	-94.30036	2	80	0	42	0	189.99	6013.28	338.51	8.70	35.53	54.25	10.22		
38	38	550	4/3/2015	34.14672	-94.30036	92	20	0	7	0	189.73	6013.80	341.88	8.70	35.53	54.25	10.22		
38	38	551	4/3/2015	34.14672	-94.30036	0	45	0	14	0	189.39	6013.65	345.60	9.10	35.53	54.25	10.22		
43	43	552	3/26/2015	34.36023	-93.03284	28	80	0	21	0	192.98	6005.74	371.58	7.79	17.62	65.56	16.82		
43	43	553	3/26/2015	34.36023	-93.03284	12	70	0	39	0	193.08	6004.94	361.99	7.15	17.62	65.56	16.82		
43	43	554	3/26/2015	34.36023	-93.03284	0	55	0	25	0	192.91	6004.12	352.37	8.97	17.62	65.56	16.82		
43	43	555	3/26/2015	34.36023	-93.03284	0	95	1	23	0	192.96	6003.47	342.82	9.06	17.62	65.56	16.82		
43	43	556	3/26/2015	34.36023	-93.03284	0	85	0	7	0	193.00	6002.45	333.25	8.45	17.62	65.56	16.82		
43	43	557	3/26/2015	34.36023	-93.03284	0	85	0	14	0	192.83	6002.67	323.75	8.97	17.62	65.56	16.82		
45	45	558	3/28/2015	34.47732	-93.26605	48	90	1	27	0	163.00	5982.89	69.77	11.91	30.42	57.12	12.46		
45	45	559	3/28/2015	34.47732	-93.26605	5	95	0	32	0	163.02	5981.12	60.67	14.78	30.42	57.12	12.46		
45	45	560	3/28/2015	34.47732	-93.26605	36	100	1	54	0	163.02	5981.40	51.91	12.82	30.42	57.12	12.46		
45	45	561	3/28/2015	34.47732	-93.26605	3	100	0	60	0	163.02	5982.40	43.68	16.82	30.42	57.12	12.46		
45	45	562	3/28/2015	34.47732	-93.26605	5	90	0	74	0	163.02	5982.40	36.34	16.82	30.42	57.12	12.46		
45	45	563	3/28/2015	34.47732	-93.26605	1	95	0	38	0	163.02	5982.63	30.60	10.31	30.42	57.12	12.46		
48	48	564	3/23/2015	34.80642	-92.77906	47	100	1	30	3	177.59	5995.43	185.99	12.53	23.60	66.03	10.37		
48	48	565	3/23/2015	34.80642	-92.77906	78	70	1	0	1	177.91	5999.93	193.19	6.68	23.60	66.03	10.37		

Table C.1 cont'd

Site	Transect	Quadrat	Date	Latitude	Longitude	Canopy	Herb	Sedge	Stem	Burrow	Elevation	Solar	Water dist	CTI	Sand	Silt	Clay	Burrow ownership	Water trns
48	48	566	3/23/2015	34.80642	-92.77906	62	78	1	18	0	178.04	6001.68	200.63	9.82	23.60	66.03	10.37		
48	48	567	3/23/2015	34.80642	-92.77906	60	90	1	45	0	178.49	6001.84	208.36	6.67	23.60	66.03	10.37		
48	48	568	3/23/2015	34.80642	-92.77906	48	95	1	55	0	178.79	6000.62	216.21	7.49	23.60	66.03	10.37		
48	48	569	3/23/2015	34.80642	-92.77906	0	90	1	38	0	179.06	5999.52	224.23	13.12	23.60	66.03	10.37		
49	49	570	4/1/2015	34.18689	-93.83407	4	70	1	12	6	196.56	6014.11	121.30	8.63	40.87	53.48	5.65		
49	49	571	4/1/2015	34.18689	-93.83407	22	45	1	11	6	197.31	6014.56	91.65	7.41	40.87	53.48	5.65		
49	49	572	4/1/2015	34.18689	-93.83407	6	20	1	3	4	197.55	6008.66	83.82	6.52	40.87	53.48	5.65		
49	49	573	4/1/2015	34.18689	-93.83407	5	40	1	13	4	196.78	6014.40	99.66	7.72	40.87	53.48	5.65		
49	49	574	4/1/2015	34.18689	-93.83407	43	15	1	13	4	196.56	6014.11	115.54	8.63	40.87	53.48	5.65		
49	49	575	4/1/2015	34.18689	-93.83407	22	30	0	13	2	196.54	6011.93	107.44	8.24	40.87	53.48	5.65		
51	51	576	3/31/2015	34.53489	-93.56304	0	95	0	24	1	207.45	5993.98	59.72	7.10	42.72	52.94	4.34		
51	51	577	3/31/2015	34.53489	-93.56304	0	99	0	22	1	207.39	5996.18	68.05	8.68	42.72	52.94	4.34		
51	51	578	3/31/2015	34.53489	-93.56304	0	95	1	44	1	207.34	5998.68	76.66	7.28	42.72	52.94	4.34		
51	51	579	3/31/2015	34.53489	-93.56304	0	99	1	12	0	207.35	6001.15	81.34	7.39	42.72	52.94	4.34		
51	51	580	3/31/2015	34.53489	-93.56304	0	65	1	22	0	207.35	6003.62	73.53	7.53	42.72	52.94	4.34		
51	51	581	3/31/2015	34.53489	-93.56304	0	45	1	13	0	207.35	6005.20	66.30	7.63	42.72	52.94	4.34		
52	52	582	3/29/2015	34.40523	-93.620426	0	99	0	42	0	191.41	5998.33	208.11	9.20	20.61	55.36	24.03		
52	52	583	3/29/2015	34.40523	-93.620426	0	95	0	11	0	191.46	5999.88	201.13	8.40	20.61	55.36	24.03		
52	52	584	3/29/2015	34.40523	-93.620426	0	90	0	24	0	191.45	6000.16	194.14	11.04	20.61	55.36	24.03		
52	52	585	3/29/2015	34.40523	-93.620426	0	85	1	35	0	191.62	6001.40	187.48	10.26	20.61	55.36	24.03		
52	52	586	3/29/2015	34.40523	-93.620426	0	85	1	35	0	191.70	6001.69	181.05	8.05	20.61	55.36	24.03		
52	52	587	3/29/2015	34.40523	-93.620426	0	80	1	42	0	191.80	6002.70	172.30	10.07	20.61	55.36	24.03		
53	53	588	3/28/2015	34.35356	-93.47742	0	65	1	39	0	198.08	6020.91	99.32	7.65	41.95	50.37	7.68		
53	53	589	3/28/2015	34.35356	-93.47742	0	85	0	37	0	198.49	6018.53	98.68	7.11	41.95	50.37	7.68		
53	53	590	3/28/2015	34.35356	-93.47742	0	90	0	48	0	198.42	6019.68	99.14	11.64	41.95	50.37	7.68		
53	53	591	3/28/2015	34.35356	-93.47742	0	80	0	52	0	198.46	6019.49	99.88	9.84	41.95	50.37	7.68		
53	53	592	3/28/2015	34.35356	-93.47742	0	85	0	64	0	198.98	6023.57	101.56	7.56	41.95	50.37	7.68		

Table C.1 cont'd

Site	Transect	Quadrat	Date	Latitude	Longitude	Canopy	Herb	Sedge	Stem	Burrow	Elevation	Solar	Water dist	CTI	Sand	Silt	Clay	Burrow ownership	Water trns
53	53	593	3/28/2015	34.35356	-93.47742	0	95	0	68	0	198.99	6022.94	100.72	8.27	41.95	50.37	7.68		
55	55	594	3/23/2015	34.79232	-92.66393	11	100	0	18	0	186.10	6005.96	123.60	6.78	20.64	64.61	14.76		
55	55	595	3/23/2015	34.79232	-92.66393	0	45	0	6	0	186.25	6004.61	123.78	7.20	20.64	64.61	14.76		
55	55	596	3/23/2015	34.79232	-92.66393	13	20	0	4	0	186.35	6004.68	124.07	7.28	20.64	64.61	14.76		
55	55	597	3/23/2015	34.79232	-92.66393	0	35	0	23	0	186.44	6005.14	124.25	7.33	20.64	64.61	14.76		
55	55	598	3/23/2015	34.79232	-92.66393	14	90	0	10	0	186.48	6005.82	124.42	7.25	20.64	64.61	14.76		
55	55	599	3/23/2015	34.79232	-92.66393	10	90	0	8	0	186.04	6010.43	124.71	7.10	20.64	64.61	14.76		
56	56	600	3/26/2015	34.33267	-93.32069	78	3	0	0	0	173.89	6001.21	289.69	8.86	19.59	63.74	16.67		
56	56	601	3/26/2015	34.33267	-93.32069	48	100	1	37	0	173.72	5996.12	295.72	9.86	19.59	63.74	16.67		
56	56	602	3/26/2015	34.33267	-93.32069	100	100	1	18	0	173.74	5993.30	301.88	9.39	19.59	63.74	16.67		
56	56	603	3/26/2015	34.33267	-93.32069	48	85	0	12	0	173.52	5994.06	308.37	8.73	19.59	63.74	16.67		
56	56	604	3/26/2015	34.33267	-93.32069	84	70	0	7	0	173.54	5992.43	314.91	8.55	19.59	63.74	16.67		
56	56	605	3/26/2015	34.33267	-93.32069	90	25	1	2	0	173.60	5991.73	321.71	8.92	19.59	63.74	16.67		
58	58	606	4/1/2015	34.13091	-93.96602	0	10	0	0	7	167.90	5993.80	238.24	7.48	39.95	54.66	5.38		
58	58	607	4/1/2015	34.13091	-93.96602	0	8	1	2	5	167.40	5996.86	231.06	8.04	39.95	54.66	5.38		
58	58	608	4/1/2015	34.13091	-93.96602	0	5	1	0	2	166.95	5994.51	223.85	8.34	39.95	54.66	5.38		
58	58	609	4/1/2015	34.13091	-93.96602	10	3	1	0	1	166.73	5995.94	216.66	9.58	39.95	54.66	5.38		
58	58	610	4/1/2015	34.13091	-93.96602	52	15	0	0	0	166.39	5997.26	208.86	9.88	39.95	54.66	5.38		
58	58	611	4/1/2015	34.13091	-93.96602	28	3	0	0	0	166.22	5996.57	200.28	10.31	39.95	54.66	5.38		
62	62	612	3/27/2015	34.11237	-93.52864	0	90	0	12	0	208.99	6027.30	155.19	8.17	56.15	34.93	7.67		
62	62	613	3/27/2015	34.11237	-93.52864	0	80	1	27	0	208.84	6027.82	147.87	8.49	56.15	34.93	7.67		
62	62	614	3/27/2015	34.11237	-93.52864	0	85	0	19	0	208.63	6025.55	140.84	9.35	56.15	34.93	7.67		
62	62	615	3/27/2015	34.11237	-93.52864	0	95	0	36	0	208.63	6026.49	134.23	7.70	56.15	34.93	7.67		
62	62	616	3/27/2015	34.11237	-93.52864	0	90	1	21	0	208.43	6028.25	128.04	7.94	56.15	34.93	7.67		
62	62	617	3/27/2015	34.11237	-93.52864	0	35	1	33	0	208.21	6029.04	122.37	8.13	56.15	34.93	7.67		
65	65	618	3/29/2015	34.26972	-93.45657	0	80	0	34	3	168.79	5999.32	186.65	5.82	38.93	50.66	10.41		
65	65	619	3/29/2015	34.26972	-93.45657	0	85	0	32	2	168.79	5999.32	194.88	5.82	38.93	50.66	10.41		

Table C.1 cont'd

Site	Transect	Quadrat	Date	Latitude	Longitude	Canopy	Herb	Sedge	Stem	Burrow	Elevation	Solar	Water dist	CTI	Sand	Silt	Clay	Burrow ownership	Water trns
65	65	620	3/29/2015	34.26972	-93.45657	0	90	0	42	2	169.76	6005.20	203.25	6.18	38.93	50.66	10.41		
65	65	621	3/29/2015	34.26972	-93.45657	4	80	1	68	1	169.78	5995.13	211.67	6.20	38.93	50.66	10.41		
65	65	622	3/29/2015	34.26972	-93.45657	0	45	1	13	1	170.03	5991.24	220.31	7.14	38.93	50.66	10.41		
65	65	623	3/29/2015	34.26972	-93.45657	16	65	1	56	0	169.63	5995.67	229.08	7.70	38.93	50.66	10.41		
67	67	624	3/27/2015	34.10209	-93.52663	0	15	0	0	0	201.20	6006.28	377.51	8.81	56.15	34.93	7.67		
67	67	625	3/27/2015	34.10209	-93.52663	32	35	1	7	0	201.48	6008.48	373.55	8.21	56.15	34.93	7.67		
67	67	626	3/27/2015	34.10209	-93.52663	0	90	0	19	0	201.53	6013.17	369.82	8.34	56.15	34.93	7.67		
67	67	627	3/27/2015	34.10209	-93.52663	0	70	1	39	0	201.52	6015.67	366.32	7.76	56.15	34.93	7.67		
67	67	628	3/27/2015	34.10209	-93.52663	1	80	1	19	0	201.52	6015.67	363.07	7.76	56.15	34.93	7.67		
67	67	629	3/27/2015	34.10209	-93.52663	0	20	0	3	0	201.41	6018.39	359.92	7.73	56.15	34.93	7.67		
68	68	630	3/26/2015	34.44685	-93.21099	0	100	0	42	2	173.27	5998.05	345.14	6.67	37.98	48.68	13.35	*F. harpi*	
68	68	631	3/26/2015	34.44685	-93.21099	0	90	0	27	1	172.98	5995.88	352.71	8.30	37.98	48.68	13.35	*F. harpi*	
68	68	632	3/26/2015	34.44685	-93.21099	0	55	1	7	1	172.61	5996.35	345.62	9.23	37.98	48.68	13.35	*F. harpi*	
68	68	633	3/26/2015	34.44685	-93.21099	0	100	0	47	1	172.22	5997.77	335.75	7.30	37.98	48.68	13.35	*F. harpi*	
68	68	634	3/26/2015	34.44685	-93.21099	0	100	0	20	0	172.22	5997.77	325.98	7.30	37.98	48.68	13.35		
68	68	635	3/26/2015	34.44685	-93.21099	0	100	0	31	0	172.08	5994.31	316.22	6.54	37.98	48.68	13.35		
70	70	636	3/31/2015	34.65402	-93.6782	0	90	0	64	2	213.88	6019.61	111.10	10.50	21.64	63.39	14.97		
70	70	637	3/31/2015	34.65402	-93.6782	0	95	1	33	1	213.86	6019.90	117.33	12.78	21.64	63.39	14.97		
70	70	638	3/31/2015	34.65402	-93.6782	0	88	1	39	0	213.87	6019.26	123.30	11.71	21.64	63.39	14.97		
70	70	639	3/31/2015	34.65402	-93.6782	0	75	1	35	0	213.82	6018.70	129.40	11.15	21.64	63.39	14.97		
70	70	640	3/31/2015	34.65402	-93.6782	0	90	1	32	0	213.77	6018.53	135.09	10.64	21.64	63.39	14.97		
70	70	641	3/31/2015	34.65402	-93.6782	0	80	1	54	0	213.58	6018.51	140.06	7.69	21.64	63.39	14.97		
75	75	642	3/28/2015	34.27823	-93.51379	0	85	0	35	0	175.55	6001.46	332.63	6.92	30.56	58.63	10.81		
75	75	643	3/28/2015	34.27823	-93.51379	38	70	0	17	0	175.55	5999.06	327.18	8.70	30.56	58.63	10.81		
75	75	644	3/28/2015	34.27823	-93.51379	36	65	0	37	0	175.84	6002.06	321.98	8.66	30.56	58.63	10.81		
75	75	645	3/28/2015	34.27823	-93.51379	29	30	0	25	0	176.02	6003.59	316.98	9.89	30.56	58.63	10.81		
75	75	646	3/28/2015	34.27823	-93.51379	76	55	0	39	0	176.65	6002.79	312.26	8.02	30.56	58.63	10.81		

Table C.1 cont'd

Site	Transect	Quadrat	Date	Latitude	Longitude	Canopy	Herb	Sedge	Stem	Burrow	Elevation	Solar	Water dist	CTI	Sand	Silt	Clay	Burrow ownership	Water trns
75	75	647	3/28/2015	34.27823	-93.51379	49	25	0	0	0	176.93	6004.80	307.49	9.39	30.56	58.63	10.81		
76	76	648	3/25/2015	34.64294	-92.94296	18	99	1	34	0	179.15	6004.16	130.71	8.98	29.68	59.40	10.92		
76	76	649	3/25/2015	34.64294	-92.94296	52	99	1	14	0	179.48	6005.30	129.79	8.70	29.68	59.40	10.92		
76	76	650	3/25/2015	34.64294	-92.94296	60	35	0	0	0	179.81	6006.07	128.88	8.41	29.68	59.40	10.92		
76	76	651	3/25/2015	34.64294	-92.94296	33	80	1	19	0	180.14	6006.38	127.89	8.16	29.68	59.40	10.92		
76	76	652	3/25/2015	34.64294	-92.94296	100	25	0	2	0	180.15	6006.48	126.98	8.13	29.68	59.40	10.92		
76	76	653	3/25/2015	34.64294	-92.94296	60	3	0	0	0	180.47	6006.13	126.06	12.32	29.68	59.40	10.92		
77	77	654	4/2/2015	34.24662	-94.01553	29	95	0	24	3	211.22	6018.27	52.01	10.43	51.36	42.15	6.49		
77	77	655	4/2/2015	34.24662	-94.01553	24	98	0	21	3	211.31	6017.80	54.71	10.07	51.36	42.15	6.49		
77	77	656	4/2/2015	34.24662	-94.01553	25	80	0	38	3	211.43	6016.99	57.29	12.70	51.36	42.15	6.49		
77	77	657	4/2/2015	34.24662	-94.01553	10	85	0	12	2	211.58	6016.27	60.00	12.51	51.36	42.15	6.49		
77	77	658	4/2/2015	34.24662	-94.01553	15	99	0	22	2	211.75	6015.74	62.59	12.34	51.36	42.15	6.49		
77	77	659	4/2/2015	34.24662	-94.01553	10	95	0	19	0	212.06	6008.76	65.28	8.32	51.36	42.15	6.49		
78	78	660	3/30/2015	34.57817	-93.57967	4	95	1	22	0	176.82	5991.79	116.08	9.29	15.63	74.09	10.28		
78	78	661	3/30/2015	34.57817	-93.57967	0	88	0	42	0	176.82	5991.15	107.76	9.69	15.63	74.09	10.28		
78	78	662	3/30/2015	34.57817	-93.57967	2	65	1	9	0	176.83	5990.62	99.38	9.27	15.63	74.09	10.28		
78	78	663	3/30/2015	34.57817	-93.57967	24	45	1	12	0	176.86	5989.20	90.65	8.33	15.63	74.09	10.28		
78	78	664	3/30/2015	34.57817	-93.57967	56	70	1	54	0	177.02	5983.28	81.60	7.24	15.63	74.09	10.28		
78	78	665	3/30/2015	34.57817	-93.57967	52	60	0	32	0	177.32	5979.85	72.23	6.91	15.63	74.09	10.28		
80	80	666	4/2/2015	34.20946	-94.05049	98	75	1	32	0	156.84	5980.20	55.85	8.17	39.90	48.71	11.39		
80	80	667	4/2/2015	34.20946	-94.05049	56	80	0	29	0	156.92	5980.98	61.12	7.47	39.90	48.71	11.39		
80	80	668	4/2/2015	34.20946	-94.05049	4	65	0	47	0	156.94	5981.31	64.74	8.54	39.90	48.71	11.39		
80	80	669	4/2/2015	34.20946	-94.05049	18	85	0	46	0	156.97	5981.38	68.23	8.18	39.90	48.71	11.39		
80	80	670	4/2/2015	34.20946	-94.05049	63	85	1	28	0	157.02	5981.46	72.05	7.51	39.90	48.71	11.39		
80	80	671	4/2/2015	34.20946	-94.05049	92	78	1	32	0	156.76	5982.48	76.85	7.17	39.90	48.71	11.39		
83	83	672	3/24/2015	34.59437	-93.1265	0	100	0	32	0	231.91	6035.28	24.44	15.56	20.44	65.93	13.63		
83	83	673	3/24/2015	34.59437	-93.1265	0	100	0	33	0	231.96	6033.42	28.75	8.95	20.44	65.93	13.63		

Table C.1 cont'd

Site	Transect	Quadrat	Date	Latitude	Longitude	Canopy	Herb	Sedge	Stem	Burrow	Elevation	Solar	Water dist	CTI	Sand	Silt	Clay	Burrow ownership	Water trns
83	83	674	3/24/2015	34.59437	-93.1265	0	100	0	56	0	232.02	6035.56	34.33	8.14	20.44	65.93	13.63		
83	83	675	3/24/2015	34.59437	-93.1265	0	100	0	28	0	232.03	6035.43	40.39	9.53	20.44	65.93	13.63		
83	83	676	3/24/2015	34.59437	-93.1265	0	100	39	0	0	232.06	6035.20	46.37	9.12	20.44	65.93	13.63		
83	83	677	3/24/2015	34.59437	-93.1265	68	100	0	23	0	232.11	6035.26	51.93	8.52	20.44	65.93	13.63		
85	85	678	3/31/2015	34.63339	-93.76313	0	45	1	3	0	197.95	6016.06	162.79	7.95	39.43	49.92	10.65		
85	85	679	3/31/2015	34.63339	-93.76313	0	75	0	9	0	197.90	6015.49	158.45	7.53	39.43	49.92	10.65		
85	85	680	3/31/2015	34.63339	-93.76313	0	85	1	14	0	197.77	6017.73	154.37	7.27	39.43	49.92	10.65		
85	85	681	3/31/2015	34.63339	-93.76313	0	85	0	10	0	197.50	6017.66	150.83	8.01	39.43	49.92	10.65		
85	85	682	3/31/2015	34.63339	-93.76313	0	80	0	6	0	197.37	6014.64	147.84	8.15	39.43	49.92	10.65		
85	85	683	3/31/2015	34.63339	-93.76313	0	78	0	9	0	197.35	6014.82	145.52	8.17	39.43	49.92	10.65		
87	87	684	3/27/2015	34.1227	-93.55003	0	90	0	7	3	174.79	5976.73	124.61	10.31	41.95	50.37	7.68		
87	87	685	3/27/2015	34.1227	-93.55003	0	65	0	9	2	174.60	5978.47	120.92	11.15	41.95	50.37	7.68		
87	87	686	3/27/2015	34.1227	-93.55003	0	15	0	8	2	174.55	5980.19	116.56	13.03	41.95	50.37	7.68		
87	87	687	3/27/2015	34.1227	-93.55003	5	35	1	3	0	174.57	5980.46	111.19	12.29	41.95	50.37	7.68		
87	87	688	3/27/2015	34.1227	-93.55003	0	45	1	9	0	174.65	5978.87	106.38	10.11	41.95	50.37	7.68		
87	87	689	3/27/2015	34.1227	-93.55003	0	45	1	4	0	174.79	5969.52	102.07	6.39	41.95	50.37	7.68		
87	87	690	3/28/2015	34.36918	-93.43809	45	80	0	26	0	191.20	6010.66	117.98	6.68	56.15	34.93	7.67		
87	87	691	3/28/2015	34.36918	-93.43809	40	99	0	37	0	190.98	6005.44	108.44	6.54	56.15	34.93	7.67		
87	87	692	3/28/2015	34.36918	-93.43809	32	95	0	19	0	189.33	6006.66	98.91	6.93	56.15	34.93	7.67		
87	87	693	3/28/2015	34.36918	-93.43809	33	85	1	17	0	189.43	6007.89	89.37	6.64	56.15	34.93	7.67		
87	87	694	3/28/2015	34.36918	-93.43809	28	85	0	7	0	189.39	6009.61	79.84	7.00	56.15	34.93	7.67		
87	87	695	3/28/2015	34.36918	-93.43809	18	75	0	47	0	188.26	6013.01	70.30	6.74	56.15	34.93	7.67		
88	88	696	3/24/2015	34.45258	-92.81526	0	75	0	27	0	184.63	5996.38	138.02	6.69	17.94	66.90	15.16		
88	88	697	3/24/2015	34.45258	-92.81526	0	95	0	110	0	184.09	5985.61	146.54	8.25	17.94	66.90	15.16		
88	88	698	3/24/2015	34.45258	-92.81526	0	100	0	27	0	183.34	5989.06	155.24	8.42	17.94	66.90	15.16		
88	88	699	3/24/2015	34.45258	-92.81526	6	100	1	19	0	182.80	5991.87	164.10	8.93	17.94	66.90	15.16		
88	88	700	3/24/2015	34.45258	-92.81526	24	95	1	32	0	182.01	5997.63	173.00	7.88	17.94	66.90	15.16		

Table C.1 cont'd

Site	Transect	Quadrat	Date	Latitude	Longitude	Canopy	Herb	Sedge	Stem	Burrow	Elevation	Solar	Waterdist	CTI	Sand	Silt	Clay	Burrow ownership	Water_trns
88	88	701	3/24/2015	34.45258	-92.81526	40	80	1	47	0	181.81	6005.45	182.09	8.84	17.94	66.90	15.16		
90	90	702	3/31/2015	34.25821	-93.80802	0	90	0	22	1	214.38	6022.78	409.74	8.52	44.30	45.60	10.10		
90	90	703	3/31/2015	34.25821	-93.80802	0	85	1	12	0	214.40	6024.58	405.60	8.74	44.30	45.60	10.10		
90	90	704	3/31/2015	34.25821	-93.80802	0	78	1	9	0	214.36	6025.05	401.65	10.77	44.30	45.60	10.10		
90	90	705	3/31/2015	34.25821	-93.80802	0	89	1	24	0	214.36	6024.83	398.02	8.35	44.30	45.60	10.10		
90	90	706	3/31/2015	34.25821	-93.80802	0	80	1	20	0	214.31	6026.11	394.51	8.68	44.30	45.60	10.10		
90	90	707	3/31/2015	34.25821	-93.80802	0	75	1	7	0	214.21	6029.53	391.25	7.96	44.30	45.60	10.10		
92	92	708	3/26/2015	34.30846	-93.06246	50	80	1	79	3	153.03	5989.16	69.29	8.45	37.96	54.17	7.87		
92	92	709	3/26/2015	34.30846	-93.06246	99	65	0	37	1	152.90	5987.67	65.34	8.06	37.96	54.17	7.87		
92	92	710	3/26/2015	34.30846	-93.06246	0	15	0	7	0	153.04	5987.61	62.74	8.05	37.96	54.17	7.87		
92	92	711	3/26/2015	34.30846	-93.06246	0	100	0	52	0	152.97	5988.56	61.67	7.73	37.96	54.17	7.87		
92	92	712	3/26/2015	34.30846	-93.06246	0	100	0	37	0	152.80	5988.50	62.21	7.63	37.96	54.17	7.87		
92	92	713	3/26/2015	34.30846	-93.06246	32	100	0	37	0	152.75	5986.78	64.31	8.35	37.96	54.17	7.87		
93	93	714	4/16/2015	34.58444	-93.97827	0	90	0	18	0	261.47	6049.36	89.06	12.14	22.14	65.75	12.11		
93	93	715	4/16/2015	34.58444	-93.97827	0	95	0	17	0	261.49	6048.92	99.04	10.83	22.14	65.75	12.11		
93	93	716	4/16/2015	34.58444	-93.97827	0	97	0	18	0	261.67	6048.49	109.01	10.02	22.14	65.75	12.11		
93	93	717	4/16/2015	34.58444	-93.97827	0	96	0	20	0	261.73	6047.83	118.99	9.87	22.14	65.75	12.11		
93	93	718	4/16/2015	34.58444	-93.97827	0	85	1	21	0	261.93	6047.60	128.98	7.90	22.14	65.75	12.11		
93	93	719	4/16/2015	34.58444	-93.97827	0	78	1	19	0	261.95	6046.36	138.96	8.13	22.14	65.75	12.11		
94	94	720	3/26/2015	34.31986	-93.16096	24	70	0	3	2	167.44	5971.32	106.56	12.11	23.63	62.74	13.63		
94	94	721	3/26/2015	34.31986	-93.16096	8	80	0	28	0	167.86	5933.07	116.47	7.45	23.63	62.74	13.63		
94	94	722	3/26/2015	34.31986	-93.16096	42	80	0	12	0	169.23	5904.58	115.50	6.89	23.63	62.74	13.63		
94	94	723	3/26/2015	34.31986	-93.16096	11	70	0	36	0	170.43	5940.31	109.66	6.96	23.63	62.74	13.63		
94	94	724	3/26/2015	34.31986	-93.16096	22	75	0	46	0	170.89	5967.89	104.61	7.44	23.63	62.74	13.63		
94	94	725	3/26/2015	34.31986	-93.16096	24	100	0	36	0	171.15	5969.93	100.30	7.06	23.63	62.74	13.63		
95	95	726	3/31/2015	34.6651	-93.61877	44	65	0	22	0	213.94	6015.32	145.61	9.79	30.46	57.84	11.71		
95	95	727	3/31/2015	34.6651	-93.61877	42	55	0	12	0	214.07	6015.99	147.06	8.45	30.46	57.84	11.71		

Table C.1 cont'd

Site	Transect	Quadrat	Date	Latitude	Longitude	Canopy	Herb	Sedge	Stem	Burrow	Elevation	Solar	Waterdist	CTI	Sand	Silt	Clay	Burrow ownership	Water_trns
95	95	728	3/31/2015	34.6651	-93.61877	22	30	0	10	0	214.13	6018.34	148.77	8.16	30.46	57.84	11.71		
95	95	729	3/31/2015	34.6651	-93.61877	16	35	0	7	0	214.12	6019.26	149.88	9.09	30.46	57.84	11.71		
95	95	730	3/31/2015	34.6651	-93.61877	25	40	0	9	0	214.12	6020.42	150.90	8.48	30.46	57.84	11.71		
95	95	731	3/31/2015	34.6651	-93.61877	44	35	0	34	0	214.05	6020.80	151.84	11.60	30.46	57.84	11.71		
97	97	732	3/27/2015	34.15429	-93.58212	24	55	0	1	1	159.06	5986.72	106.13	8.17	36.12	56.42	7.47		
97	97	733	3/27/2015	34.15429	-93.58212	28	65	0	40	1	159.26	5988.10	106.21	8.43	36.12	56.42	7.47		
97	97	734	3/27/2015	34.15429	-93.58212	52	65	0	37	1	159.22	5987.61	107.22	7.60	36.12	56.42	7.47		
97	97	735	3/27/2015	34.15429	-93.58212	27	75	0	2	0	159.16	5987.12	108.53	8.58	36.12	56.42	7.47		
97	97	736	3/27/2015	34.15429	-93.58212	76	30	0	0	0	159.09	5986.79	104.76	8.81	36.12	56.42	7.47		
97	97	737	3/27/2015	34.15429	-93.58212	60	30	0	0	0	159.04	5986.36	101.37	7.74	36.12	56.42	7.47		
101	101	738	3/30/2015	34.86133	-93.3456	32	15	0	3	0	194.78	6004.73	141.58	8.72	30.33	59.99	9.68		
101	101	739	3/30/2015	34.86133	-93.3456	28	17	1	7	0	194.78	6004.73	134.41	8.72	30.33	59.99	9.68		
101	101	740	3/30/2015	34.86133	-93.3456	94	20	1	35	0	194.76	6007.06	127.48	9.96	30.33	59.99	9.68		
101	101	741	3/30/2015	34.86133	-93.3456	36	70	1	32	0	194.76	6007.06	121.06	9.96	30.33	59.99	9.68		
101	101	742	3/30/2015	34.86133	-93.3456	49	18	1	2	0	194.73	6007.82	114.63	9.52	30.33	59.99	9.68		
101	101	743	3/30/2015	34.86133	-93.3456	64	8	0	0	0	194.75	6001.92	108.26	9.02	30.33	59.99	9.68		
102	102	744	3/23/2015	34.65306	-92.50546	79	3	1	2	0	156.80	5991.64	135.22	7.96	19.44	67.75	12.80		
102	102	745	3/23/2015	34.65306	-92.50546	15	15	1	0	0	157.30	5993.49	125.33	6.67	19.44	67.75	12.80		
102	102	746	3/23/2015	34.65306	-92.50546	13	18	1	2	0	157.40	5987.79	115.46	7.51	19.44	67.75	12.80		
102	102	747	3/23/2015	34.65306	-92.50546	22	17	1	9	0	157.48	5986.97	105.63	8.16	19.44	67.75	12.80		
102	102	748	3/23/2015	34.65306	-92.50546	24	90	1	34	0	157.48	5986.62	95.70	9.33	19.44	67.75	12.80		
102	102	749	3/23/2015	34.65306	-92.50546	37	95	1	33	0	157.48	5984.93	85.94	7.00	19.44	67.75	12.80		
104	104	750	3/25/2015	34.64087	-92.93977	0	90	1	22	0	175.24	6002.95	74.09	6.87	29.68	59.40	10.92		
104	104	751	3/25/2015	34.64087	-92.93977	4	75	1	19	0	176.32	6007.61	84.05	6.75	29.68	59.40	10.92		
104	104	752	3/25/2015	34.64087	-92.93977	72	90	1	16	0	176.44	5999.41	94.06	7.16	29.68	59.40	10.92		
104	104	753	3/25/2015	34.64087	-92.93977	40	90	0	17	0	176.64	5996.12	104.03	7.88	29.68	59.40	10.92		
104	104	754	3/25/2015	34.64087	-92.93977	8	65	0	26	0	175.95	5989.17	113.96	7.02	29.68	59.40	10.92		

Table C.1 cont'd

Site	Transect	Quadrat	Date	Latitude	Longitude	Canopy	Herb	Sedge	Stem	Burrow	Elevation	Solar	Water_dist	CTI	Sand	Silt	Clay	Burrow ownership	Water_trns
104	104	755	3/25/2015	34.64087	-92.93977	0	100	0	27	0	176.03	5989.03	124.03	7.76	29.68	59.40	10.92		
109	109	756	4/1/2015	34.15819	-93.89613	25	18	0	0	1	206.80	6012.68	401.59	6.73	69.01	27.73	3.26		
109	109	757	4/1/2015	34.15819	-93.89613	6	45	1	0	0	207.08	6009.64	393.12	6.10	69.01	27.73	3.26		
109	109	758	4/1/2015	34.15819	-93.89613	68	70	1	0	0	207.14	6004.54	384.77	7.58	69.01	27.73	3.26		
109	109	759	4/1/2015	34.15819	-93.89613	45	30	0	0	0	207.08	6001.57	376.45	7.87	69.01	27.73	3.26		
109	109	760	4/1/2015	34.15819	-93.89613	12	30	1	3	0	207.03	6009.04	368.27	9.77	69.01	27.73	3.26		
109	109	761	4/1/2015	34.15819	-93.89613	91	25	1	7	0	207.22	6019.48	360.21	9.10	69.01	27.73	3.26		
111	111	762	3/26/2015	34.42896	-93.28929	0	30	0	0	0	186.05	5993.48	230.75	7.13	39.91	49.27	10.83		
111	111	763	3/26/2015	34.42896	-93.28929	0	75	1	5	0	186.55	5998.84	222.67	7.23	39.91	49.27	10.83		
111	111	764	3/26/2015	34.42896	-93.28929	0	90	1	62	0	187.12	6003.36	214.79	5.51	39.91	49.27	10.83		
111	111	765	3/26/2015	34.42896	-93.28929	0	90	1	0	0	186.05	5999.42	207.06	6.51	39.91	49.27	10.83		
111	111	766	3/26/2015	34.42896	-93.28929	0	80	0	52	0	186.26	6002.58	199.52	7.11	39.91	49.27	10.83		
111	111	767	3/26/2015	34.42896	-93.28929	0	100	1	54	0	186.66	6003.74	192.26	7.93	39.91	49.27	10.83		
112	112	768	4/3/2015	34.19992	-94.26167	0	65	1	32	0	160.58	5980.97	63.86	8.43	32.42	55.98	11.60		
112	112	769	4/3/2015	34.19992	-94.26167	0	90	0	29	0	160.96	5979.16	63.08	8.07	32.42	55.98	11.60		
112	112	770	4/3/2015	34.19992	-94.26167	0	85	0	39	0	161.38	5977.52	63.75	8.89	32.42	55.98	11.60		
112	112	771	4/3/2015	34.19992	-94.26167	0	95	0	32	0	161.87	5961.86	61.49	8.31	32.42	55.98	11.60		
112	112	772	4/3/2015	34.19992	-94.26167	0	79	0	22	0	162.80	5956.22	60.02	8.15	32.42	55.98	11.60		
112	112	773	4/3/2015	34.19992	-94.26167	0	80	1	30	0	163.70	5972.21	58.61	6.46	32.42	55.98	11.60		
117	117	774	4/3/2015	34.09761	-94.46558	52	100	0	32	0	159.36	5978.63	689.92	8.33	35.22	53.75	11.03		
117	117	775	4/3/2015	34.09761	-94.46558	32	90	0	29	0	158.97	5972.28	683.58	8.51	35.22	53.75	11.03		
117	117	776	4/3/2015	34.09761	-94.46558	32	95	0	42	0	158.90	5972.29	677.26	6.74	35.22	53.75	11.03		
117	117	777	4/3/2015	34.09761	-94.46558	5	98	1	70	0	158.62	5976.08	671.03	7.76	35.22	53.75	11.03		
117	117	778	4/3/2015	34.09761	-94.46558	3	95	1	42	0	158.52	5979.40	664.72	9.49	35.22	53.75	11.03		
117	117	779	4/3/2015	34.09761	-94.46558	20	88	1	20	0	158.52	5979.43	658.40	7.17	35.22	53.75	11.03		
118	118	780	3/31/2015	34.646	-93.71104	66	15	0	10	0	221.08	6026.54	46.50	7.31	21.64	63.39	14.97		
118	118	781	3/31/2015	34.646	-93.71104	99	5	0	3	0	221.25	6025.70	49.70	7.48	21.64	63.39	14.97		

Table C.1 cont'd

Site	Transect	Quadrat	Date	Latitude	Longitude	Canopy	Herb	Sedge	Stem	Burrow	Elevation	Solar	Water dist	CTI	Sand	Silt	Clay	Burrow ownership	Water trns
118	118	782	3/31/2015	34.646	-93.71104	98	3	0	0	0	221.34	6026.21	54.48	7.66	21.64	63.39	14.97		
118	118	783	3/31/2015	34.646	-93.71104	67	10	0	0	0	221.42	6026.88	60.62	7.32	21.64	63.39	14.97		
118	118	784	3/31/2015	34.646	-93.71104	91	15	0	6	0	221.63	6027.60	67.61	6.86	21.64	63.39	14.97		
118	118	785	3/31/2015	34.646	-93.71104	98	3	0	3	0	222.01	6028.77	75.35	6.65	21.64	63.39	14.97		
120	120	786	3/23/2015	34.74667	-92.85654	28	45	1	7	1	177.04	6003.57	67.64	8.11	18.28	61.07	20.65		
120	120	787	3/23/2015	34.74667	-92.85654	36	35	1	32	1	177.74	6004.33	71.67	7.97	18.28	61.07	20.65		
120	120	788	3/23/2015	34.74667	-92.85654	32	90	0	37	0	178.51	6003.93	76.43	7.74	18.28	61.07	20.65		
120	120	789	3/23/2015	34.74667	-92.85654	52	95	1	48	0	179.07	6004.52	79.82	7.64	18.28	61.07	20.65		
120	120	790	3/23/2015	34.74667	-92.85654	20	70	1	28	0	179.81	6002.95	82.73	7.24	18.28	61.07	20.65		
120	120	791	3/23/2015	34.74667	-92.85654	16	90	1	16	0	180.68	6003.31	85.53	6.91	18.28	61.07	20.65		
122	122	792	3/23/2015	34.87427	-92.63171	0	100	0	32	0	101.15	5945.89	131.53	6.48	6.48	83.64	9.89		
122	122	793	3/23/2015	34.87427	-92.63171	0	100	0	35	0	101.83	5943.45	141.48	6.39	6.48	83.64	9.89		
122	122	794	3/23/2015	34.87427	-92.63171	0	100	1	22	0	102.25	5944.48	151.43	6.54	6.48	83.64	9.89		
122	122	795	3/23/2015	34.87427	-92.63171	0	100	0	60	0	103.28	5946.37	161.46	6.34	6.48	83.64	9.89		
122	122	796	3/23/2015	34.87427	-92.63171	0	100	0	39	0	103.74	5941.17	171.41	6.31	6.48	83.64	9.89		
122	122	797	3/23/2015	34.87427	-92.63171	76	100	0	66	0	104.18	5909.96	181.36	9.88	6.48	83.64	9.89		
125	125	798	3/29/2015	34.31173	-93.67787	24	0	0	0	0	262.93	6059.46	150.24	6.93	35.57	51.99	12.44		
125	125	799	3/29/2015	34.31173	-93.67787	26	35	0	5	0	262.62	6060.30	148.22	6.78	35.57	51.99	12.44		
125	125	800	3/29/2015	34.31173	-93.67787	48	3	0	0	0	262.30	6062.24	146.93	6.26	35.57	51.99	12.44		
125	125	801	3/29/2015	34.31173	-93.67787	10	80	0	11	0	261.56	6060.60	146.19	7.95	35.57	51.99	12.44		
125	125	802	3/29/2015	34.31173	-93.67787	41	20	0	3	0	261.56	6060.60	146.26	7.95	35.57	51.99	12.44		
125	125	803	3/29/2015	34.31173	-93.67787	30	15	0	2	0	260.60	6053.77	146.92	7.80	35.57	51.99	12.44		
128	128	804	4/2/2015	34.26218	-93.95017	0	45	0	42	0	248.84	5973.12	42.88	5.59	37.65	53.37	8.98		
128	128	805	4/2/2015	34.26218	-93.95017	19	80	1	13	0	248.24	5985.35	46.66	8.07	37.65	53.37	8.98		
128	128	806	4/2/2015	34.26218	-93.95017	0	85	1	33	0	248.61	5994.62	51.93	5.89	37.65	53.37	8.98		
128	128	807	4/2/2015	34.26218	-93.95017	0	70	1	25	0	248.90	6000.21	57.61	8.27	37.65	53.37	8.98		
128	128	808	4/2/2015	34.26218	-93.95017	0	85	1	23	0	248.71	6009.19	64.24	6.15	37.65	53.37	8.98		

Table C.1 cont'd

Site	Transect	Quadrat	Date	Latitude	Longitude	Canopy	Herb	Sedge	Stem	Burrow	Elevation	Solar	Water dist	CTI	Sand	Silt	Clay	Burrow ownership	Water trns
128	128	809	4/2/2015	34.26218	-93.95017	24	65	1	12	0	248.87	6013.06	71.54	8.25	37.65	53.37	8.98		
132	132	810	3/29/2015	34.26529	-93.47874	13	80	1	68	0	142.72	5978.30	138.73	9.93	30.56	58.63	10.81		
132	132	811	3/29/2015	34.26529	-93.47874	25	99	0	32	0	142.89	5976.37	148.62	10.36	30.56	58.63	10.81		
132	132	812	3/29/2015	34.26529	-93.47874	26	85	0	22	0	143.08	5969.68	158.42	9.08	30.56	58.63	10.81		
132	132	813	3/29/2015	34.26529	-93.47874	58	99	0	22	0	143.90	5971.36	168.34	8.83	30.56	58.63	10.81		
132	132	814	3/29/2015	34.26529	-93.47874	75	90	1	47	0	144.28	5974.32	176.86	8.61	30.56	58.63	10.81		
132	132	815	3/29/2015	34.26529	-93.47874	50	99	1	48	0	145.09	5980.38	185.26	6.87	30.56	58.63	10.81		
133	133	816	4/2/2015	34.31225	-94.13503	23	2	0	0	0	275.09	6010.90	336.35	7.27	36.92	49.99	13.08		
133	133	817	4/2/2015	34.31225	-94.13503	0	0	0	0	0	275.92	6041.97	339.25	6.50	36.92	49.99	13.08		
133	133	818	4/2/2015	34.31225	-94.13503	2	5	0	0	0	275.83	6061.12	342.36	7.92	36.92	49.99	13.08		
133	133	819	4/2/2015	34.31225	-94.13503	43	15	0	3	0	275.89	6066.14	345.78	7.94	36.92	49.99	13.08		
133	133	820	4/2/2015	34.31225	-94.13503	0	88	0	21	0	275.61	6070.73	349.50	7.26	36.92	49.99	13.08		
133	133	821	4/2/2015	34.31225	-94.13503	0	95	0	37	0	275.36	6071.35	353.42	7.00	36.92	49.99	13.08		
134	134	822	4/3/2015	34.29995	-94.14393	16	20	1	9	1	222.23	5955.91	352.45	7.91	36.92	49.99	13.08		
134	134	823	4/3/2015	34.29995	-94.14393	32	60	1	13	1	221.90	5925.54	361.75	5.51	36.92	49.99	13.08		
134	134	824	4/3/2015	34.29995	-94.14393	2	65	0	29	0	221.02	5883.19	371.18	6.36	36.92	49.99	13.08		
134	134	825	4/3/2015	34.29995	-94.14393	48	35	0	7	0	219.62	5964.49	380.55	6.02	36.92	49.99	13.08		
134	134	826	4/3/2015	34.29995	-94.14393	25	78	1	7	0	219.02	5960.58	386.23	9.27	36.92	49.99	13.08		
134	134	827	4/3/2015	34.29995	-94.14393	0	20	0	0	0	218.68	5988.61	378.76	10.28	36.92	49.99	13.08		
136	136	828	3/30/2015	34.85619	-93.82548	16	30	0	2	0	203.44	5992.63	156.44	5.46	14.22	72.57	13.21		
136	136	829	3/30/2015	34.85619	-93.82548	22	70	0	4	0	202.30	5986.73	160.08	8.56	14.22	72.57	13.21		
136	136	830	3/30/2015	34.85619	-93.82548	8	25	0	12	0	200.77	5988.71	162.86	8.69	14.22	72.57	13.21		
136	136	831	3/30/2015	34.85619	-93.82548	14	20	0	0	0	201.07	5971.50	164.17	8.55	14.22	72.57	13.21		
136	136	832	3/30/2015	34.85619	-93.82548	32	70	1	20	0	199.91	5991.65	165.47	10.98	14.22	72.57	13.21		
136	136	833	3/30/2015	34.85619	-93.82548	8	65	1	27	0	200.86	5984.05	166.78	5.55	14.22	72.57	13.21		
137	137	834	4/3/2015	34.23858	-94.08827	52	88	0	8	0	226.23	6023.98	278.28	6.07	20.41	64.93	14.66		
137	137	835	4/3/2015	34.23858	-94.08827	62	85	0	32	0	227.76	6036.55	274.63	5.73	20.41	64.93	14.66		

Table C.1 cont'd

Site	Transect	Quadrat	Date	Latitude	Longitude	Canopy	Herb	Sedge	Stem	Burrow	Elevation	Solar	Water dist	CTI	Sand	Silt	Clay	Burrow ownership	Water trns
137	137	836	4/3/2015	34.23858	-94.08827	2	90	0	42	0	227.57	6035.04	271.41	5.65	20.41	64.93	14.66		
137	137	837	4/3/2015	34.23858	-94.08827	0	95	0	27	0	227.57	6037.57	268.41	9.72	20.41	64.93	14.66		
137	137	838	4/3/2015	34.23858	-94.08827	76	65	0	47	0	227.41	6037.59	265.87	9.83	20.41	64.93	14.66		
137	137	839	4/3/2015	34.23858	-94.08827	38	55	0	9	0	228.75	6030.41	263.20	7.24	20.41	64.93	14.66		
138	138	840	4/3/2015	34.07553	-94.46408	40	55	0	12	0	130.71	5962.98	199.68	8.35	35.22	53.75	11.03		
138	138	841	4/3/2015	34.07553	-94.46408	36	85	0	12	0	130.89	5961.46	199.20	8.49	35.22	53.75	11.03		
138	138	842	4/3/2015	34.07553	-94.46408	68	20	0	7	0	130.81	5958.42	198.71	6.86	35.22	53.75	11.03		
138	138	843	4/3/2015	34.07553	-94.46408	28	85	1	28	0	131.09	5959.74	198.23	8.35	35.22	53.75	11.03		
138	138	844	4/3/2015	34.07553	-94.46408	48	90	1	25	0	131.31	5958.40	197.67	8.48	35.22	53.75	11.03		
138	138	845	4/3/2015	34.07553	-94.46408	82	90	1	36	0	131.20	5952.30	197.19	6.46	35.22	53.75	11.03		
139	139	846	4/2/2015	34.31769	-94.05876	0	20	0	0	0	279.26	6055.71	567.72	9.39	30.55	59.51	9.94		
139	139	847	4/2/2015	34.31769	-94.05876	2	25	0	11	0	279.32	6055.30	572.91	14.10	30.55	59.51	9.94		
139	139	848	4/2/2015	34.31769	-94.05876	0	0	0	0	0	279.64	6046.50	578.12	6.82	30.55	59.51	9.94		
139	139	849	4/2/2015	34.31769	-94.05876	12	5	0	11	0	279.64	6046.50	583.56	6.82	30.55	59.51	9.94		
139	139	850	4/2/2015	34.31769	-94.05876	0	8	1	12	0	280.24	6045.67	589.13	7.43	30.55	59.51	9.94		
139	139	851	4/2/2015	34.31769	-94.05876	0	7	0	3	0	280.39	6043.50	594.69	6.68	30.55	59.51	9.94		
140	140	852	4/1/2015	34.1141	-93.83714	0	98	1	42	0	147.22	5988.63	227.48	6.94	20.46	71.47	8.07		
140	140	853	4/1/2015	34.1141	-93.83714	0	80	0	27	0	147.01	5988.84	237.37	8.51	20.46	71.47	8.07		
140	140	854	4/1/2015	34.1141	-93.83714	0	85	0	14	0	146.64	5984.48	247.37	6.90	20.46	71.47	8.07		
140	140	855	4/1/2015	34.1141	-93.83714	28	65	1	16	0	146.12	5978.75	257.27	6.70	20.46	71.47	8.07		
140	140	856	4/1/2015	34.1141	-93.83714	66	57	0	4	0	145.37	5971.65	267.27	6.56	20.46	71.47	8.07		
140	140	857	4/1/2015	34.1141	-93.83714	18	87	1	35	0	144.56	5964.76	277.20	7.15	20.46	71.47	8.07		
142	142	858	4/12/2015	34.48577	-93.8707	80	35	1	2	0	330.55	6082.79	158.68	6.90	14.56	70.84	14.61		
142	142	859	4/12/2015	34.48577	-93.8707	65	15	1	0	0	330.32	6084.97	153.24	7.21	14.56	70.84	14.61		
142	142	860	4/12/2015	34.48577	-93.8707	50	65	1	6	0	330.19	6086.60	145.23	7.48	14.56	70.84	14.61		
142	142	861	4/12/2015	34.48577	-93.8707	40	85	0	27	0	330.09	6086.05	137.39	7.51	14.56	70.84	14.61		
142	142	862	4/12/2015	34.48577	-93.8707	30	95	0	30	0	329.82	6082.85	129.85	7.85	14.56	70.84	14.61		

Table C.1 cont'd

Site	Transect	Quadrat	Date	Latitude	Longitude	Canopy	Herb	Sedge	Stem	Burrow	Elevation	Solar	Water_dist	CTI	Sand	Silt	Clay	Burrow ownership	Watertrns
142	142	863	4/12/2015	34.48577	-93.8707	47	65	0	16	0	329.57	6082.22	122.74	7.08	14.56	70.84	14.61		
143	143	864	3/29/2015	34.30464	-93.39529	44	78	0	20	0	157.03	5992.37	141.38	6.83	41.52	49.43	9.05		
143	143	865	3/29/2015	34.30464	-93.39529	78	55	0	14	0	157.03	5992.37	144.72	6.83	41.52	49.43	9.05		
143	143	866	3/29/2015	34.30464	-93.39529	75	56	0	28	0	156.68	5992.18	148.24	6.53	41.52	49.43	9.05		
143	143	867	3/29/2015	34.30464	-93.39529	55	20	0	7	0	156.86	5991.67	151.89	7.75	41.52	49.43	9.05		
143	143	868	3/29/2015	34.30464	-93.39529	57	15	0	8	0	157.11	5992.14	155.91	6.50	41.52	49.43	9.05		
143	143	869	3/29/2015	34.30464	-93.39529	84	30	0	12	0	156.10	5990.17	159.98	7.89	41.52	49.43	9.05		
146	146	870	4/9/2015	34.59105	-94.36073	0	80	1	28	0	321.98	6096.20	345.48	7.13	40.99	47.79	11.21		
146	146	871	4/9/2015	34.59105	-94.36073	0	95	1	17	0	322.05	6096.51	342.46	8.66	40.99	47.79	11.21		
146	146	872	4/9/2015	34.59105	-94.36073	0	90	0	33	0	322.05	6096.33	339.58	8.91	40.99	47.79	11.21		
146	146	873	4/9/2015	34.59105	-94.36073	0	70	0	22	0	322.12	6096.17	336.64	9.56	40.99	47.79	11.21		
146	146	874	4/9/2015	34.59105	-94.36073	0	85	0	19	0	322.20	6096.02	334.08	9.19	40.99	47.79	11.21		
146	146	875	4/9/2015	34.59105	-94.36073	0	65	0	17	0	322.29	6095.21	331.69	9.59	40.99	47.79	11.21		
148	148	876	3/27/2015	34.15646	-93.27261	0	35	0	4	0	140.15	5973.27	336.14	8.72	42.49	50.46	7.05		
148	148	877	3/27/2015	34.15646	-93.27261	32	45	0	27	0	139.79	5972.44	331.73	6.46	42.49	50.46	7.05		
148	148	878	3/27/2015	34.15646	-93.27261	60	15	0	0	0	139.23	5965.52	327.57	7.57	42.49	50.46	7.05		
148	148	879	3/27/2015	34.15646	-93.27261	40	30	0	2	0	138.50	5967.37	323.67	7.82	42.49	50.46	7.05		
148	148	880	3/27/2015	34.15646	-93.27261	68	5	0	0	0	138.03	5972.65	319.98	8.20	42.49	50.46	7.05		
148	148	881	3/27/2015	34.15646	-93.27261	80	5	0	0	0	137.42	5977.41	316.61	13.41	42.49	50.46	7.05		
149	149	882	3/24/2015	34.53686	-92.68876	0	90	0	24	0	139.90	5966.85	180.83	6.07	29.23	61.50	9.26		
149	149	883	3/24/2015	34.53686	-92.68876	0	100	0	54	0	139.28	5958.41	184.28	5.95	29.23	61.50	9.26		
149	149	884	3/24/2015	34.53686	-92.68876	0	100	0	46	0	139.28	5958.41	188.12	5.95	29.23	61.50	9.26		
149	149	885	3/24/2015	34.53686	-92.68876	0	100	0	38	0	138.64	5942.94	192.42	6.64	29.23	61.50	9.26		
149	149	886	3/24/2015	34.53686	-92.68876	0	100	0	21	0	137.71	5913.38	197.11	6.46	29.23	61.50	9.26		
149	149	887	3/24/2015	34.53686	-92.68876	0	100	0	61	0	135.72	5898.00	202.22	8.89	29.23	61.50	9.26		
150	150	888	3/25/2015	34.69566	-92.97722	84	15	1	0	0	195.90	5954.82	117.59	6.72	28.35	52.90	18.75		

Table C.1 cont'd

Site	Transect	Quadrat	Date	Latitude	Longitude	Canopy	Herb	Sedge	Stem	Burrow	Elevation	Solar	Water dist	CTI	Sand	Silt	Clay	Burrow ownership	Watertrns
150	150	889	3/25/2015	34.69566	-92.97722	55	8	0	0	0	194.81	5840.70	119.83	6.73	28.35	52.90	18.75		
150	150	890	3/25/2015	34.69566	-92.97722	88	15	0	2	0	194.87	5814.70	122.90	5.80	28.35	52.90	18.75		
150	150	891	3/25/2015	34.69566	-92.97722	76	5	0	0	0	194.58	5830.99	126.55	4.72	28.35	52.90	18.75		
150	150	892	3/25/2015	34.69566	-92.97722	82	5	0	15	0	190.01	5818.43	131.01	5.84	28.35	52.90	18.75		
150	150	893	3/25/2015	34.69566	-92.97722	68	3	1	0	0	188.94	5822.54	135.97	8.06	28.35	52.90	18.75		
154	154	894	3/25/2015	34.64.829	-92.95192	76	58	0	98	0	226.60	5978.55	438.85	7.59	29.68	59.40	10.92		
154	154	895	3/25/2015	34.64.829	-92.95192	62	90	1	66	0	227.31	5980.47	432.29	7.17	29.68	59.40	10.92		
154	154	896	3/25/2015	34.64.829	-92.95192	56	85	1	47	0	227.90	5972.09	425.73	7.31	29.68	59.40	10.92		
154	154	897	3/25/2015	34.64.829	-92.95192	68	55	1	17	0	229.41	5964.91	419.44	6.98	29.68	59.40	10.92		
154	154	898	3/25/2015	34.64.829	-92.95192	68	15	0	7	0	230.03	5946.68	413.16	6.12	29.68	59.40	10.92		
154	154	899	3/25/2015	34.64.829	-92.95192	72	15	1	6	0	232.06	5969.51	407.16	5.61	29.68	59.40	10.92		
156	156	900	3/27/2015	34.12791	-93.32538	20	65	0	9	0	104.60	5956.59	85.90	8.44	46.96	45.42	7.62		
156	156	901	3/27/2015	34.12791	-93.32538	12	35	0	3	0	104.59	5955.92	75.88	6.75	46.96	45.42	7.62		
156	156	902	3/27/2015	34.12791	-93.32538	20	65	0	23	0	104.43	5952.14	65.95	8.36	46.96	45.42	7.62		
156	156	903	3/27/2015	34.12791	-93.32538	80	5	0	3	0	103.79	5944.02	56.03	8.64	46.96	45.42	7.62		
156	156	904	3/27/2015	34.12791	-93.32538	68	3	0	1	0	103.22	5926.52	46.01	6.29	46.96	45.42	7.62		
156	156	905	3/27/2015	34.12791	-93.32538	100	2	0	0	0	101.89	5873.00	36.08	5.82	46.96	45.42	7.62		
159	159	906	3/24/2015	34.57591	-92.98907	96	5	0	0	0	206.90	5845.52	313.55	5.74	10.29	74.76	14.95		
159	159	907	3/24/2015	34.57591	-92.98907	62	2	0	0	0	206.24	5814.19	306.15	5.63	10.29	74.76	14.95		
159	159	908	3/24/2015	34.57591	-92.98907	60	3	0	0	0	205.85	5776.71	298.91	5.53	10.29	74.76	14.95		
159	159	909	3/24/2015	34.57591	-92.98907	76	2	0	0	0	205.82	5761.93	291.83	5.49	10.29	74.76	14.95		
159	159	910	3/24/2015	34.57591	-92.98907	92	0	0	0	0	206.08	5757.94	284.93	5.48	10.29	74.76	14.95		
159	159	911	3/24/2015	34.57591	-92.98907	48	4	0	2	0	206.63	5744.00	278.21	6.16	10.29	74.76	14.95		

APPENDIX D: *PROCAMBARUS REIMERI* SAMPLING SUPPLEMENTARY MATERIAL

Table D.1: Habitat covariates and counts measured at each quadrat for *Procambarus reimeri* in western Arkansas in the spring of 2014 and 2015. See Table 2.1 in main body of thesis for a description of each variable. The water_trns corresponds to the water_trans covariate in the main body, this variable was not measured in 2015. The burrow ownership column depicts the assumed ownership of each burrow counted in the corresponding quadrat.

Site	Transect	Quadrat	Date	Latitude	Longitude	Canopy	Herb	Sedge	Stem	Burrow	Elevation	Solar	Water_dist	CTI	Sand	Silt	Clay	Burrow ownership	Water_trns
1	36	210	4/21/2014	34.58384	-94.14815	56	82	0	26	0	289.98	6071.78	62.75	10.83	23.360	60.210	16.430		Y
1	36	211	4/21/2014	34.58385	-94.14825	2	76	1	36	0	289.68	6072.71	70.37	8.97	23.360	60.210	16.430		Y
1	36	212	4/21/2014	34.58385	-94.14836	2	48	1	18	0	289.53	6072.47	78.44	9.40	13.380	67.570	19.050		Y
1	36	213	4/21/2014	34.58385	-94.14847	1	68	1	29	0	289.39	6072.79	86.95	9.64	13.380	67.570	19.050		Y
1	36	214	4/21/2014	34.58386	-94.14858	0	25	1	46	0	289.24	6073.48	95.64	8.61	11.060	66.200	22.740		Y
1	36	215	4/21/2014	34.58386	-94.14869	0	65	1	36	1	289.03	6073.88	104.45	9.24	11.060	66.200	22.740	*P. reimeri*	Y
1	37	216	4/21/2014	34.58377	-94.14818	100	3	1	0	0	289.72	6070.08	59.81	8.12	27.270	57.390	14.510		N
1	37	217	4/21/2014	34.58377	-94.14829	95	10	1	5	0	289.57	6070.61	68.18	10.55	27.270	57.390	14.510		N
1	37	218	4/21/2014	34.58378	-94.14840	60	60	0	15	0	289.43	6070.98	76.85	9.64	37.370	47.920	14.710		N
1	37	219	4/21/2014	34.58378	-94.14850	96	3	1	0	0	289.24	6071.04	85.63	10.26	37.370	47.920	14.710		N
1	37	220	4/21/2014	34.58379	-94.14861	100	54	1	6	0	288.82	6072.07	94.58	8.81	33.190	54.500	12.310		N
1	37	221	4/21/2014	34.58379	-94.14872	0	100	1	28	0	288.70	6073.28	103.76	9.31	33.190	54.500	12.310		N
1	38	222	4/21/2014	34.58403	-94.14822	0	80	1	48	0	290.12	6074.66	82.89	9.33	37.070	48.260	14.670		N
1	38	223	4/21/2014	34.58403	-94.14834	0	85	1	40	2	289.90	6075.23	90.29	7.99	37.070	48.260	14.670	*P. reimeri*	N
1	38	224	4/21/2014	34.58404	-94.14846	0	85	1	4	1	289.74	6075.35	98.26	9.02	35.460	49.660	14.880	*P. reimeri*	N
1	38	225	4/21/2014	34.58404	-94.14857	0	90	1	47	0	289.62	6075.40	106.79	9.60	35.460	49.660	14.880		N
1	38	226	4/21/2014	34.58405	-94.14869	0	100	0	27	1	289.50	6075.64	115.63	13.85	31.860	52.520	15.420	*P. reimeri*	N
1	38	227	4/21/2014	34.58405	-94.14881	0	100	0	48	0	289.32	6076.25	124.85	10.48	31.860	52.520	15.420		N
2	39	228	4/21/2014	34.60709	-94.16787	0	95	0	46	0	293.31	6073.12	75.45	11.32	16.540	44.370	39.090		Y
2	39	229	4/21/2014	34.60715	-94.16779	1	100	0	14	0	293.42	6074.25	84.59	9.35	16.540	44.370	39.090		Y
2	39	230	4/21/2014	34.60720	-94.16770	26	95	0	37	0	293.42	6074.50	93.74	10.37	43.760	38.000	18.250		Y
2	39	231	4/21/2014	34.60726	-94.16762	8	95	0	14	0	293.52	6074.46	102.85	9.72	43.760	38.000	18.250		Y
2	39	232	4/21/2014	34.60732	-94.16753	50	100	0	36	1	293.63	6074.14	112.10	9.03	29.640	43.410	26.950	*P. reimeri*	Y
2	39	233	4/21/2014	34.60737	-94.16745	0	100	0	27	0	293.61	6073.53	121.35	7.93	29.640	43.410	26.950		Y

Table D.1 cont'd

Site	Transect	Quadrat	Date	Latitude	Longitude	Canopy	Herb	Sedge	Stem	Burrow	Elevation	Solar	Water_dist	CTI	Sand	Silt	Clay	Burrow ownership	Water_trns
2	40	234	4/21/2014	34.60757	-94.16715	0	100	0	37	0	293.51	6073.14	134.72	9.33	31.880	50.250	17.870		Y
2	40	235	4/21/2014	34.60763	-94.16707	0	100	1	38	0	293.50	6072.22	124.80	8.05	31.880	50.250	17.870		Y
2	40	236	4/21/2014	34.60768	-94.16698	32	100	1	19	0	293.41	6072.10	114.89	12.73	32.590	46.350	21.060		Y
2	40	237	4/21/2014	34.60774	-94.16690	0	100	0	34	0	293.34	6072.01	105.03	8.22	32.590	46.350	21.060		Y
2	40	238	4/21/2014	34.60780	-94.16681	0	90	1	38	0	293.32	6071.59	95.04	8.54	30.470	51.870	17.660		Y
2	40	239	4/21/2014	34.60785	-94.16673	0	80	0	28	0	293.26	6072.10	85.12	9.22	30.470	51.870	17.660		Y
2	41	240	4/21/2014	34.60764	-94.16742	0	100	0	22	0	293.77	6073.43	134.28	8.59	26.830	53.480	19.690		Y
2	41	241	4/21/2014	34.60758	-94.16750	0	100	0	40	0	293.75	6073.99	124.82	8.51	26.830	53.480	19.690		Y
2	41	242	4/21/2014	34.60753	-94.16759	0	100	0	48	0	293.75	6073.99	115.37	8.93	25.490	51.550	22.960		Y
2	41	243	4/21/2014	34.60747	-94.16767	0	100	0	50	0	293.73	6074.64	105.87	9.26	25.490	51.550	22.960		Y
2	41	244	4/21/2014	34.60741	-94.16776	0	100	1	47	0	293.77	6075.34	96.41	9.86	29.880	49.570	20.550		Y
2	41	245	4/21/2014	34.60736	-94.16784	0	97	1	47	1	293.66	6075.79	86.96	8.24	29.880	49.570	20.550	*P. reimeri*	Y
3	42	246	4/22/2014	34.65613	-94.10335	9	100	0	14	0	336.38	6093.12	4.68	10.58	41.960	37.590	20.450		Y
3	42	247	4/22/2014	34.65613	-94.10324	0	100	1	27	0	336.81	6094.18	14.41	7.55	41.960	37.590	20.450		Y
3	42	248	4/22/2014	34.65613	-94.10313	0	75	1	47	0	336.97	6094.48	24.01	9.05	42.230	37.290	20.480		Y
3	42	249	4/22/2014	34.65613	-94.10301	1	100	1	26	0	337.15	6094.47	33.80	9.13	42.230	37.290	20.480		Y
3	42	250	4/22/2014	34.65613	-94.10290	0	100	0	36	0	337.32	6094.35	43.76	9.15	29.740	50.410	19.850		Y
3	42	251	4/22/2014	34.65613	-94.10279	0	95	0	56	0	337.47	6094.52	53.84	6.94	29.740	50.410	19.850		Y
3	43	252	4/22/2014	34.65633	-94.10260	100	1	0	0	0	338.48	6096.04	76.64	8.25	34.910	45.970	19.120		Y
3	43	253	4/22/2014	34.65633	-94.10249	87	3	1	0	0	338.94	6096.24	86.33	8.76	34.910	45.970	19.120		Y
3	43	254	4/22/2014	34.65633	-94.10238	100	3	0	0	0	339.25	6096.65	96.06	8.19	36.600	46.430	16.970		Y
3	43	255	4/22/2014	34.65633	-94.10226	100	15	0	4	0	339.50	6097.07	105.82	8.28	36.600	46.430	16.970		Y
3	43	256	4/22/2014	34.65633	-94.10215	76	3	1	0	0	339.77	6096.89	115.70	7.43	40.460	43.610	15.930		Y
3	43	257	4/22/2014	34.65633	-94.10204	96	15	0	0	0	340.10	6097.78	125.62	8.36	40.460	43.610	15.930		Y
3	44	258	4/22/2014	34.65633	-94.10268	27	100	1	22	0	338.48	6096.04	69.71	8.25	30.070	54.680	15.250		N
3	44	259	4/22/2014	34.65642	-94.10267	36	95	0	25	0	338.72	6095.63	74.21	9.11	30.070	54.680	15.250		N
3	44	260	4/22/2014	34.65651	-94.10265	32	35	1	12	0	338.92	6096.64	79.33	8.38	43.570	35.540	20.890		N

Table D.1 cont'd

Site	Transect	Quadrat	Date	Latitude	Longitude	Canopy	Herb	Sedge	Stem	Burrow	Elevation	Solar	Water_dist	CTI	Sand	Silt	Clay	Burrow ownership	Water_trns
3	44	261	4/22/2014	34.65660	-94.10264	68	43	1	0	0	339.31	6097.35	84.45	8.03	43.570	35.540	20.890		N
3	44	262	4/22/2014	34.65670	-94.10263	40	95	1	13	0	339.62	6096.41	89.57	7.83	46.010	38.830	15.050		N
3	44	263	4/22/2014	34.65679	-94.10261	60	100	1	17	0	339.94	6095.55	94.48	7.63	46.010	38.830	15.050		N
4	45	264	4/22/2014	34.65406	-94.11623	0	90	1	36	1	340.93	6097.71	148.57	8.13	43.810	34.560	21.530	*P. reimeri*	Y
4	45	265	4/22/2014	34.65396	-94.11623	0	100	1	47	0	340.78	6095.67	149.13	10.89	43.810	34.560	21.530		Y
4	45	266	4/22/2014	34.65387	-94.11623	0	98	1	52	2	340.72	6092.59	150.10	7.32	25.890	51.290	22.820	*P. reimeri*	Y
4	45	267	4/22/2014	34.65378	-94.11623	0	85	1	38	1	340.76	6087.56	151.22	9.03	25.890	51.290	22.820	*P. reimeri*	Y
4	45	268	4/22/2014	34.65369	-94.11623	0	97	1	62	1	340.95	6081.14	153.07	8.59	50.620	32.430	16.950	*P. reimeri*	Y
4	45	269	4/22/2014	34.65360	-94.11624	72	90	1	69	1	341.30	6078.54	155.52	7.52	50.620	32.430	16.950	*P. reimeri*	Y
4	46	270	4/22/2014	34.65407	-94.11607	0	100	1	37	0	341.40	6096.86	134.18	7.41	32.840	41.970	25.190		Y
4	46	271	4/22/2014	34.65398	-94.11607	0	100	1	63	0	341.31	6094.63	134.66	10.28	32.840	41.970	25.190		Y
4	46	272	4/22/2014	34.65389	-94.11607	0	100	0	81	0	341.27	6091.33	135.54	9.47	38.530	43.190	18.280		Y
4	46	273	4/22/2014	34.65379	-94.11608	0	100	0	52	0	341.36	6087.79	136.74	8.94	38.530	43.190	18.280		Y
4	46	274	4/22/2014	34.65370	-94.11608	0	100	0	46	2	341.52	6086.21	138.66	8.10	30.240	47.400	22.360	*P. reimeri*	Y
4	46	275	4/22/2014	34.65361	-94.11608	0	99	1	32	1	341.70	6085.94	141.05	7.72	30.240	47.400	22.360	*P. reimeri*	Y
4	47	276	4/22/2014	34.65407	-94.11564	100	1	0	0	0	342.28	6097.74	94.78	9.47	30.240	47.400	22.360		N
4	47	277	4/22/2014	34.65398	-94.11564	92	0	0	0	0	342.21	6095.20	95.37	8.07	30.440	59.680	9.880		N
4	47	278	4/22/2014	34.65389	-94.11564	92	1	1	0	0	342.28	6094.26	96.25	8.94	30.440	59.680	9.880		N
4	47	279	4/22/2014	34.65379	-94.11565	96	3	0	0	0	342.29	6096.11	97.84	9.14	30.440	59.680	9.880		N
4	47	280	4/22/2014	34.65370	-94.11565	92	3	1	6	0	342.23	6096.54	100.26	8.90	32.510	53.950	13.540		N
4	47	281	4/22/2014	34.65361	-94.11565	100	2	1	0	0	342.21	6095.66	103.04	10.55	32.510	53.950	13.540		N
4	48	282	4/22/2014	34.65445	-94.11616	0	100	0	39	0	342.56	6102.63	147.33	8.67	25.880	50.260	23.860		Y
4	48	283	4/22/2014	34.65445	-94.11627	0	70	1	29	0	342.33	6102.67	157.09	9.50	25.880	50.260	23.860		Y
4	48	284	4/22/2014	34.65445	-94.11638	0	80	1	21	0	342.14	6102.39	166.86	9.23	35.070	47.370	17.370		Y
4	48	285	4/22/2014	34.65445	-94.11649	0	70	1	28	1	341.89	6102.36	176.67	6.66	35.070	47.370	17.370	*P. reimeri*	Y
4	48	286	4/22/2014	34.65445	-94.11660	0	65	0	17	0	341.78	6102.63	186.39	9.33	35.440	43.560	21.000		Y
4	48	287	4/22/2014	34.65445	-94.11670	0	95	1	22	1	341.62	6101.88	196.24	6.94	35.440	43.560	21.000	*P. reimeri*	Y

Table D.1 cont'd

Site	Transect	Quadrat	Date	Latitude	Longitude	Canopy	Herb	Sedge	Stem	Burrow	Elevation	Solar	Water_dist	CTI	Sand	Silt	Clay	Burrow ownership	Water_trns
5	49	288	4/22/2014	34.65771	-94.15163	0	100	0	29	0	338.28	6103.39	55.14	6.63	21.780	60.450	17.770		N
5	49	289	4/22/2014	34.65763	-94.15168	0	75	0	25	0	338.13	6103.77	52.21	6.42	21.780	60.450	17.770		N
5	49	290	4/22/2014	34.65755	-94.15172	0	100	0	44	0	337.60	6103.11	49.69	6.83	19.290	57.040	23.670		N
5	49	291	4/22/2014	34.65747	-94.15177	48	100	1	58	0	337.48	6102.39	49.07	7.39	19.290	57.040	23.670		N
5	49	292	4/22/2014	34.65739	-94.15182	0	100	0	39	0	337.18	6100.67	49.83	7.86	29.950	43.900	26.060		N
5	49	293	4/22/2014	34.65730	-94.15187	0	100	0	55	0	337.12	6102.34	52.14	7.86	29.950	43.900	26.060		N
5	50	294	4/22/2014	34.65777	-94.15186	0	70	1	52	1	339.35	6105.56	75.21	7.23	24.640	55.140	20.230	*P. reimeri*	Y
5	50	295	4/22/2014	34.65785	-94.15181	0	90	1	30	0	339.50	6104.29	78.15	7.54	24.640	55.140	20.230		Y
5	50	296	4/22/2014	34.65793	-94.15177	0	85	1	46	1	339.94	6104.49	81.75	8.22	23.290	51.220	25.490	*P. reimeri*	Y
5	50	297	4/22/2014	34.65801	-94.15172	0	12	1	0	2	340.10	6105.66	86.12	7.29	23.290	51.220	25.490	*P. reimeri*	Y
5	50	298	4/22/2014	34.65810	-94.15167	0	15	1	26	1	340.55	6104.40	90.74	8.71	39.180	40.680	20.140	*P. reimeri*	Y
5	50	299	4/22/2014	34.65818	-94.15162	0	100	1	76	0	340.58	6103.52	95.95	7.51	39.180	40.680	20.140		Y
5	51	300	4/22/2014	34.65734	-94.15216	1	70	0	37	0	338.11	6105.17	79.38	6.40	36.710	49.040	14.250		Y
5	51	301	4/22/2014	34.65726	-94.15221	38	90	0	44	0	337.72	6105.57	82.85	6.41	36.710	49.040	14.250		Y
5	51	302	4/22/2014	34.65718	-94.15225	52	75	1	37	0	337.38	6104.59	87.33	12.47	20.090	48.300	31.610		Y
5	51	303	4/22/2014	34.65710	-94.15230	32	70	1	3	0	336.85	6102.62	92.20	8.18	20.090	48.300	31.610		Y
5	51	304	4/22/2014	34.65701	-94.15235	3	100	1	55	0	336.87	6099.00	97.72	9.76	29.630	51.970	18.400		Y
5	51	305	4/22/2014	34.65693	-94.15240	8	45	1	0	0	336.62	6097.52	103.83	6.27	29.630	51.970	18.400		Y
5	52	306	4/22/2014	34.65712	-94.15261	100	0	0	0	0	338.65	6093.40	120.28	8.44	43.000	44.710	12.100		Y
5	52	307	4/22/2014	34.65720	-94.15256	92	3	1	0	0	338.37	6097.75	115.49	9.76	43.000	44.710	12.100		Y
5	52	308	4/22/2014	34.65728	-94.15252	84	10	1	0	0	338.58	6100.26	111.26	8.09	20.450	56.420	23.130		Y
5	52	309	4/22/2014	34.65736	-94.15247	80	20	0	2	0	338.69	6104.68	107.79	7.53	20.450	56.420	23.130		Y
5	52	310	4/22/2014	34.65745	-94.15242	100	30	1	11	0	339.08	6105.17	105.06	12.77	15.690	54.640	29.570		Y
5	52	311	4/22/2014	34.65753	-94.15237	72	100	1	22	0	339.30	6106.47	103.15	7.09	15.690	54.640	29.570		Y
6	53	312	4/22/2014	34.65057	-94.16443	0	100	0	27	0	333.64	6101.48	82.42	7.68	28.160	50.520	21.320		Y
6	53	313	4/22/2014	34.65055	-94.16454	0	100	1	48	0	333.79	6096.39	92.49	7.91	28.160	50.520	21.320		Y
6	53	314	4/22/2014	34.65053	-94.16465	8	24	1	32	0	334.00	6091.73	102.59	9.49	17.250	46.870	35.760		Y

Table D.1 cont'd

Site	Transect	Quadrat	Date	Latitude	Longitude	Canopy	Herb	Sedge	Stem	Burrow	Elevation	Solar	Water_dist	CTI	Sand	Silt	Clay	Burrow ownership	Water_trns
6	53	315	4/22/2014	34.65050	-94.16475	0	54	1	42	0	334.28	6085.21	112.71	7.69	17.250	46.870	35.760		Y
6	53	316	4/22/2014	34.65048	-94.16486	0	38	1	47	1	334.55	6076.03	122.83	6.98	26.820	54.330	18.850	*P. reimeri*	Y
6	53	317	4/22/2014	34.65046	-94.16497	0	100	1	17	0	335.44	6078.82	132.97	6.35	26.820	54.330	18.850		Y
6	54	318	4/22/2014	34.65041	-94.16422	0	80	1	33	1	332.43	6099.03	65.97	7.97	25.430	52.440	22.130	*P. reimeri*	Y
6	54	319	4/22/2014	34.65039	-94.16433	0	76	1	17	1	332.76	6098.63	76.16	8.12	25.430	52.440	22.130	*P. reimeri*	Y
6	54	320	4/22/2014	34.65037	-94.16444	0	100	1	64	0	332.82	6095.64	86.35	13.01	32.510	50.530	16.960		Y
6	54	321	4/22/2014	34.65034	-94.16455	0	95	1	26	1	333.60	6090.38	96.54	9.01	32.510	50.530	16.960	*P. reimeri*	Y
6	54	322	4/22/2014	34.65032	-94.16465	0	95	1	32	2	334.11	6084.98	106.73	7.55	30.510	55.880	13.610	*P. reimeri*	Y
6	54	323	4/22/2014	34.65030	-94.16476	0	70	1	42	1	334.68	6082.76	116.92	6.63	30.510	55.880	13.610	*P. reimeri*	Y
6	55	324	4/22/2014	34.65014	-94.16463	100	3	0	0	0	333.98	6091.83	110.85	7.44	29.580	54.460	15.960		N
6	55	325	4/22/2014	34.65016	-94.16452	80	3	0	0	0	333.01	6091.11	100.80	7.84	29.580	54.460	15.960		N
6	55	326	4/22/2014	34.65018	-94.16441	100	10	1	18	0	332.60	6092.50	90.79	6.52	31.710	53.850	14.440		N
6	55	327	4/22/2014	34.65021	-94.16431	100	3	0	0	0	332.32	6093.50	80.82	9.95	31.710	53.850	14.440		N
6	55	328	4/22/2014	34.65023	-94.16420	100	3	0	1	0	331.92	6093.30	70.87	12.88	30.740	52.610	16.660		N
6	55	329	4/22/2014	34.65025	-94.16409	100	3	1	1	0	331.48	6093.46	60.93	12.87	30.740	52.610	16.660		N
7	56	330	4/23/2014	34.63678	-94.20505	0	100	1	46	0	329.33	6087.85	21.38	11.56	29.360	53.140	17.500		Y
7	56	331	4/23/2014	34.63677	-94.20494	0	70	1	38	0	329.31	6086.41	18.80	9.19	29.360	53.140	17.500		Y
7	56	332	4/23/2014	34.63676	-94.20483	0	70	1	48	0	329.32	6085.65	18.87	7.61	29.190	52.660	18.070		Y
7	56	333	4/23/2014	34.63675	-94.20472	0	75	1	33	0	329.50	6085.06	21.98	7.64	29.190	52.660	18.070		Y
7	56	334	4/23/2014	34.63674	-94.20462	0	45	1	31	2	329.42	6085.45	27.88	10.37	29.940	52.400	17.660	*P. reimeri*	Y
7	56	335	4/23/2014	34.63673	-94.20451	0	100	1	54	1	329.43	6084.77	35.14	8.37	29.940	52.400	17.660	*P. reimeri*	Y
7	57	336	4/23/2014	34.63665	-94.20506	0	100	0	66	0	329.51	6087.07	34.81	9.48	29.980	55.560	14.460		Y
7	57	337	4/23/2014	34.63664	-94.20495	0	100	1	48	0	329.57	6087.42	33.09	9.25	29.980	55.560	14.460		Y
7	57	338	4/23/2014	34.63663	-94.20484	0	100	1	36	0	329.61	6088.30	33.13	8.58	27.440	49.400	23.160		Y
7	57	339	4/23/2014	34.63662	-94.20473	0	100	1	84	0	329.59	6087.91	35.65	8.25	27.440	49.400	23.160		Y
7	57	340	4/23/2014	34.63661	-94.20462	0	100	1	76	4	329.53	6086.30	39.82	10.55	33.460	48.700	17.840	*P. reimeri*	Y
7	57	341	4/23/2014	34.63660	-94.20452	0	100	1	88	0	329.61	6086.68	45.77	8.03	33.460	48.700	17.840		Y

Table D.1 cont'd

Site	Transect	Quadrat	Date	Latitude	Longitude	Canopy	Herb	Sedge	Stem	Burrow	Elevation	Solar	Water_dist	CTI	Sand	Silt	Clay	Burrow ownership	Water_trns
7	58	342	4/23/2014	34.63655	-94.20507	0	100	0	100	1	329.59	6087.16	45.70	8.77	18.600	46.330	35.060	*P. reimeri*	Y
7	58	343	4/23/2014	34.63654	-94.20496	0	99	0	66	0	329.63	6087.65	44.20	8.28	18.600	46.330	35.060		Y
7	58	344	4/23/2014	34.63653	-94.20485	0	99	1	74	0	329.62	6088.35	44.33	9.39	22.830	54.120	23.050		Y
7	58	345	4/23/2014	34.63652	-94.20474	0	100	1	48	1	329.62	6088.60	46.30	10.98	22.830	54.120	23.050	*P. reimeri*	Y
7	58	346	4/23/2014	34.63651	-94.20464	0	100	1	44	1	329.62	6088.13	49.69	9.45	39.500	44.100	16.290	*P. reimeri*	Y
7	58	347	4/23/2014	34.63650	-94.20453	0	100	1	53	1	329.61	6089.15	54.73	8.86	39.500	44.100	16.290	*P. reimeri*	Y
7	59	348	4/23/2014	34.63643	-94.20512	0	100	0	57	0	329.63	6085.98	59.77	8.97	33.410	50.130	16.460		Y
7	59	349	4/23/2014	34.63642	-94.20501	0	100	1	74	0	329.69	6086.94	58.10	8.73	33.410	50.130	16.460		Y
7	59	350	4/23/2014	34.63641	-94.20490	0	95	1	44	0	329.71	6088.60	57.86	11.48	23.540	59.580	16.880		Y
7	59	351	4/23/2014	34.63640	-94.20479	0	100	1	40	3	329.68	6089.11	58.84	8.84	23.540	59.580	16.880	*P. reimeri*	Y
7	59	352	4/23/2014	34.63639	-94.20469	0	100	1	47	0	329.62	6089.48	61.40	11.59	39.290	43.600	17.110		Y
7	59	353	4/23/2014	34.63638	-94.20458	0	100	0	38	0	329.70	6089.90	65.08	13.22	39.290	43.600	17.110		Y
7	60	354	4/23/2014	34.63633	-94.20512	0	100	0	54	0	329.81	6087.58	70.35	9.30	34.360	50.370	15.270		Y
7	60	355	4/23/2014	34.63632	-94.20501	0	100	1	56	0	329.76	6087.88	69.02	8.81	34.360	50.370	15.270		Y
7	60	356	4/23/2014	34.63631	-94.20490	0	100	1	51	1	329.73	6088.44	68.91	10.08	24.780	51.240	23.980	*P. reimeri*	Y
7	60	357	4/23/2014	34.63630	-94.20479	0	100	0	44	0	329.70	6088.37	69.89	8.45	24.780	51.240	23.980		Y
7	60	358	4/23/2014	34.63629	-94.20469	0	100	1	28	1	329.74	6088.56	72.27	10.13	43.890	42.900	13.220	*P. reimeri*	Y
7	60	359	4/23/2014	34.63628	-94.20458	0	100	0	58	0	329.71	6089.74	75.64	9.49	43.890	42.900	13.220		Y
7	61	360	4/23/2014	34.63607	-94.20445	0	35	1	28	0	329.63	6090.51	101.66	13.12	40.690	40.770	18.530		N
7	61	361	4/23/2014	34.63598	-94.20445	0	15	1	26	0	329.65	6089.67	111.47	9.32	40.690	40.770	18.530		N
7	61	362	4/23/2014	34.63588	-94.20445	0	80	1	29	0	329.70	6088.70	121.33	8.51	38.250	48.790	12.960		N
7	61	363	4/23/2014	34.63579	-94.20445	0	85	1	68	0	329.74	6089.26	131.29	8.90	38.250	48.790	12.960		N
7	61	364	4/23/2014	34.63570	-94.20445	0	35	1	34	0	329.77	6089.65	141.29	11.11	39.400	46.090	14.510		N
7	61	365	4/23/2014	34.63561	-94.20445	0	100	1	88	0	329.81	6089.56	151.34	8.85	39.400	46.090	14.510		N
8	62	366	4/23/2014	34.55729	-94.20766	0	95	1	36	3	315.93	6083.94	127.84	12.24	20.710	58.550	20.740	*P. reimeri*	N
8	62	367	4/23/2014	34.55736	-94.20773	0	90	1	26	1	315.85	6084.73	137.91	10.56	20.710	58.550	20.740	*P. reimeri*	N
8	62	368	4/23/2014	34.55743	-94.20780	0	99	1	36	0	315.83	6084.69	148.05	8.39	31.090	54.750	14.130		N

Table D.1 cont'd

Site	Transect	Quadrat	Date	Latitude	Longitude	Canopy	Herb	Sedge	Stem	Burrow	Elevation	Solar	Water_dist	CTI	Sand	Silt	Clay	Burrow ownership	Water_trns
8	62	369	4/23/2014	34.55751	-94.20786	0	99	0	47	0	315.80	6085.73	158.13	12.84	31.090	54.750	14.130		N
8	62	370	4/23/2014	34.55758	-94.20793	0	100	0	34	0	315.82	6086.18	168.35	8.44	18.400	61.510	20.090		N
8	62	371	4/23/2014	34.55765	-94.20800	0	100	0	51	0	315.77	6083.60	178.42	8.28	18.400	61.510	20.090		N
8	63	372	4/23/2014	34.55721	-94.20782	0	95	1	46	0	315.88	6082.88	130.84	8.87	12.600	66.800	20.600		N
8	63	373	4/23/2014	34.55728	-94.20789	0	85	1	32	0	315.78	6082.64	140.93	9.19	12.600	66.800	20.600		N
8	63	374	4/23/2014	34.55736	-94.20796	0	80	1	34	0	315.75	6083.58	151.04	14.29	12.750	65.050	22.200		N
8	63	375	4/23/2014	34.55743	-94.20802	0	100	1	47	1	315.71	6085.78	161.08	14.29	12.750	65.050	22.200	*P. reimeri*	N
8	63	376	4/23/2014	34.55750	-94.20809	0	65	1	29	1	315.70	6084.86	171.17	8.64	12.330	63.640	24.030	*P. reimeri*	N
8	63	377	4/23/2014	34.55757	-94.20816	0	90	1	39	1	315.60	6083.29	181.21	8.09	12.330	63.640	24.030	*P. reimeri*	N
8	64	378	4/23/2014	34.55741	-94.20753	52	100	1	38	0	316.01	6084.26	132.20	8.98	41.980	44.780	12.730		N
8	64	379	4/23/2014	34.55748	-94.20760	74	100	1	52	0	315.92	6084.39	142.25	8.84	41.980	44.780	12.730		N
8	64	380	4/23/2014	34.55755	-94.20767	100	65	1	28	0	315.96	6085.68	152.26	12.20	19.840	65.160	15.000		N
8	64	381	4/23/2014	34.55763	-94.20773	56	100	1	41	0	315.95	6086.03	162.42	7.98	19.840	65.160	15.000		N
8	64	382	4/23/2014	34.55770	-94.20780	100	95	1	35	0	315.89	6085.73	172.44	10.39	38.170	52.950	8.880		N
8	64	383	4/23/2014	34.55777	-94.20787	100	80	1	52	0	315.89	6085.73	182.48	10.59	38.170	52.950	8.880		N
8	65	384	4/23/2014	34.55681	-94.20713	0	85	1	12	3	316.49	6078.72	56.16	7.69	18.460	60.980	20.560	*P. reimeri*	Y
8	65	385	4/23/2014	34.55675	-94.20705	0	45	1	0	1	316.65	6078.77	46.31	7.61	18.460	60.980	20.560	*P. reimeri*	Y
8	65	386	4/23/2014	34.55668	-94.20697	0	80	1	16	2	316.67	6077.63	36.60	8.24	26.720	52.760	20.520	*P. reimeri*	Y
8	65	387	4/23/2014	34.55662	-94.20689	0	80	1	5	0	316.89	6076.70	27.30	7.41	26.720	52.760	20.520		Y
8	65	388	4/23/2014	34.55655	-94.20681	0	95	1	27	0	317.02	6076.96	18.89	7.74	21.180	47.990	30.830		Y
8	65	389	4/23/2014	34.55649	-94.20674	0	95	1	29	1	317.01	6075.42	11.81	7.64	21.180	47.990	30.830	*P. reimeri*	Y
8	66	390	4/23/2014	34.55671	-94.20714	0	85	1	47	1	316.55	6079.65	47.68	9.04	19.860	63.060	17.080	*P. reimeri*	Y
8	66	391	4/23/2014	34.55665	-94.20706	0	100	1	63	0	316.74	6079.83	37.58	8.75	19.860	63.060	17.080		Y
8	66	392	4/23/2014	34.55658	-94.20698	0	100	0	29	0	316.84	6078.35	27.47	8.40	14.430	57.760	27.800		Y
8	66	393	4/23/2014	34.55652	-94.20690	0	100	0	29	0	317.00	6078.82	17.53	7.74	14.430	57.760	27.800		Y
8	66	394	4/23/2014	34.55645	-94.20682	0	100	0	44	0	317.20	6078.91	8.13	7.83	21.450	47.890	30.660		Y
8	66	395	4/23/2014	34.55639	-94.20674	0	100	0	48	0	317.34	6077.19	1.31	7.39	21.450	47.890	30.660		Y

Table D.1 cont'd

Site	Transect	Quadrat	Date	Latitude	Longitude	Canopy	Herb	Sedge	Stem	Burrow	Elevation	Solar	Water_dist	CTI	Sand	Silt	Clay	Burrow ownership	Water_trns
8	67	396	4/23/2014	34.55673	-94.20679	0	45	1	18	0	316.68	6079.42	38.79	8.10	18.980	52.060	28.960		Y
8	67	397	4/23/2014	34.55667	-94.20670	0	30	1	0	0	316.61	6079.52	32.74	8.76	18.980	52.060	28.960		Y
8	67	398	4/23/2014	34.55661	-94.20662	0	40	1	30	0	316.67	6074.62	28.96	8.76	20.420	51.990	27.580		Y
8	67	399	4/23/2014	34.55656	-94.20653	0	99	0	46	0	316.81	6074.01	26.25	7.93	20.420	51.990	27.580		Y
8	67	400	4/23/2014	34.55650	-94.20645	0	90	1	37	0	316.69	6076.65	25.51	7.88	27.910	52.700	19.390		Y
8	67	401	4/23/2014	34.55644	-94.20636	0	80	1	48	2	316.70	6075.76	24.89	9.28	27.910	52.700	19.390	*P. reimeri*	Y
4	68	402	4/23/2014	34.65411	-94.11646	0	100	1	34	2	340.53	6099.74	170.11	7.89	18.860	55.870	25.270	*P. reimeri*	Y
4	68	403	4/23/2014	34.65411	-94.11657	0	80	0	23	0	340.24	6100.02	180.00	7.57	18.860	55.870	25.270		Y
4	68	404	4/23/2014	34.65411	-94.11668	0	100	1	27	1	339.66	6099.93	189.98	7.75	17.010	52.980	30.010	*P. reimeri*	Y
4	68	405	4/23/2014	34.65411	-94.11679	0	100	1	36	1	339.32	6099.67	199.97	7.33	17.010	52.980	30.010	*P. reimeri*	Y
4	68	406	4/23/2014	34.65411	-94.11690	22	3	0	7	0	338.98	6099.25	209.95	7.08	40.420	37.260	22.320		Y
4	68	407	4/23/2014	34.65411	-94.11701	0	100	1	47	2	338.63	6098.68	219.93	6.98	40.420	37.260	22.320	*P. reimeri*	Y
4	69	408	4/23/2014	34.65365	-94.11678	0	100	0	48	0	338.63	6069.79	189.36	8.65	30.580	45.250	24.170		Y
4	69	409	4/23/2014	34.65365	-94.11689	0	80	1	38	0	337.88	6067.71	193.60	8.63	30.580	45.250	24.170		Y
4	69	410	4/23/2014	34.65365	-94.11700	0	90	1	19	0	337.06	6068.06	197.76	8.34	36.980	41.570	21.450		Y
4	69	411	4/23/2014	34.65365	-94.11711	0	85	1	22	1	336.20	6074.97	202.35	8.58	36.980	41.570	21.450	*P. reimeri*	Y
4	69	412	4/23/2014	34.65365	-94.11722	92	15	0	7	0	335.57	6084.38	207.02	11.80	45.750	41.720	12.530		Y
4	69	413	4/23/2014	34.65365	-94.11733	96	3	0	0	0	334.92	6092.19	211.79	12.63	45.750	41.720	12.530		Y
9	70	414	4/23/2014	34.65422	-94.15506	0	80	1	24	0	336.27	6092.25	29.68	10.52	24.220	62.050	13.730		Y
9	70	415	4/23/2014	34.65428	-94.15498	0	100	1	28	0	336.61	6097.11	36.57	9.74	24.220	62.050	13.730		Y
9	70	416	4/23/2014	34.65435	-94.15490	0	95	1	34	0	336.76	6099.29	43.55	9.53	24.880	60.440	14.680		Y
9	70	417	4/23/2014	34.65441	-94.15482	0	95	1	27	0	336.95	6097.45	50.56	9.47	24.880	60.440	14.680		Y
9	70	418	4/23/2014	34.65447	-94.15474	0	30	1	7	1	337.23	6098.50	58.37	9.04	35.400	42.620	21.980	*P. reimeri*	Y
9	70	419	4/23/2014	34.65453	-94.15466	0	30	1	6	0	337.39	6100.11	66.46	9.30	35.400	42.620	21.980		Y
9	71	420	4/23/2014	34.65416	-94.15478	36	3	0	7	0	336.48	6102.91	55.71	11.65	37.930	40.190	21.890		N
9	71	421	4/23/2014	34.65423	-94.15470	36	35	0	5	0	336.79	6102.51	62.68	9.25	37.930	40.190	21.890		N
9	71	422	4/23/2014	34.65429	-94.15462	56	20	0	4	0	337.14	6101.90	69.56	6.84	29.240	56.090	14.670		N

Table D.1 cont'd

Site	Transect	Quadrat	Date	Latitude	Longitude	Canopy	Herb	Sedge	Stem	Burrow	Elevation	Solar	Water_dist	CTI	Sand	Silt	Clay	Burrow ownership	Water_trns
9	71	423	4/23/2014	34.65435	-94.15454	80	10	0	0	0	337.18	6102.68	76.45	7.07	29.240	56.090	14.670		N
9	71	424	4/23/2014	34.65441	-94.15446	72	35	0	5	0	337.46	6102.54	83.56	8.05	21.100	55.270	23.630		N
9	71	425	4/23/2014	34.65447	-94.15438	88	3	0	0	0	337.81	6102.74	91.21	7.52	21.100	55.270	23.630		N
9	72	426	4/23/2014	34.65387	-94.15496	96	3	0	0	0	335.56	6104.52	44.74	7.80	25.650	51.520	22.840		N
9	72	427	4/23/2014	34.65393	-94.15488	100	3	0	0	0	335.76	6104.56	50.70	8.23	25.650	51.520	22.840		N
9	72	428	4/23/2014	34.65400	-94.15480	66	10	0	3	0	336.18	6104.92	56.65	7.31	34.740	44.130	21.130		N
9	72	429	4/23/2014	34.65406	-94.15472	100	10	0	2	0	336.10	6105.60	62.59	7.47	34.740	44.130	21.130		N
9	72	430	4/23/2014	34.65412	-94.15464	84	3	0	0	0	336.52	6104.41	68.62	7.25	31.490	44.830	23.690		N
9	72	431	4/23/2014	34.65418	-94.15456	94	3	0	1	0	336.89	6103.58	75.32	10.91	31.490	44.830	23.690		N
160	160	912	4/17/2015	34.41528	-94.35553	10	98	0	42	0	335.83	6101.24	128.79	8.89	42.17	49.05	8.78		
160	160	913	4/17/2015	34.41528	-94.35553	4	95	0	38	0	335.93	6101.94	131.34	8.52	42.17	49.05	8.78		
160	160	914	4/17/2015	34.41528	-94.35553	10	97	0	25	0	335.96	6101.54	134.20	8.68	42.17	49.05	8.78		
160	160	915	4/17/2015	34.41528	-94.35553	6	95	0	32	0	336.15	6101.35	137.13	8.08	42.17	49.05	8.78		
160	160	916	4/17/2015	34.41528	-94.35553	5	93	0	23	0	336.10	6101.62	140.07	10.40	42.17	49.05	8.78		
160	160	917	4/17/2015	34.41528	-94.35553	10	97	0	26	0	336.25	6102.43	142.94	8.46	42.17	49.05	8.78		
161	161	918	4/21/2015	34.35899	-94.41644	21	80	1	25	0	335.80	6093.39	251.31	8.35	28.46	55.12	16.42		
161	161	919	4/21/2015	34.35899	-94.41644	27	95	1	16	0	335.60	6090.48	244.19	8.53	28.46	55.12	16.42		
161	161	920	4/21/2015	34.35899	-94.41644	27	90	1	32	0	335.33	6092.45	237.41	8.89	28.46	55.12	16.42		
161	161	921	4/21/2015	34.35899	-94.41644	25	85	1	30	0	335.14	6091.08	230.73	7.15	28.46	55.12	16.42		
161	161	922	4/21/2015	34.35899	-94.41644	32	80	1	7	0	334.90	6089.15	224.43	9.19	28.46	55.12	16.42		
161	161	923	4/21/2015	34.35899	-94.41644	20	85	0	9	0	334.67	6088.68	218.27	7.02	28.46	55.12	16.42		
163	163	924	4/19/2015	34.37500	-94.34000	8	90	0	13	0	350.27	6109.74	96.26	8.25	45.70	47.08	7.23		
163	163	925	4/19/2015	34.37500	-94.34000	20	85	0	12	0	350.27	6109.74	105.46	8.25	45.70	47.08	7.23		
163	163	926	4/19/2015	34.37500	-94.34000	24	95	0	12	0	349.97	6110.47	114.75	6.98	45.70	47.08	7.23		
163	163	927	4/19/2015	34.37500	-94.34000	30	85	0	16	0	349.64	6104.44	124.20	6.68	45.70	47.08	7.23		
163	163	928	4/19/2015	34.37500	-94.34000	7	95	0	19	0	349.04	6100.00	133.67	7.21	45.70	47.08	7.23		
163	163	929	4/19/2015	34.37500	-94.34000	42	90	0	11	0	349.03	6102.05	143.19	7.28	45.70	47.08	7.23		

Table D.1 cont'd

Site	Transect	Quadrat	Date	Latitude	Longitude	Canopy	Herb	Sedge	Stem	Burrow	Elevation	Solar	Water_dist	CTI	Sand	Silt	Clay	Burrow ownership	Water_trns
164	164	930	4/18/2015	34.39311	-94.33522	1	86	1	18	0	344.91	6105.27	137.81	8.38	45.70	47.08	7.23		
164	164	931	4/18/2015	34.39311	-94.33522	40	80	1	7	0	345.23	6103.47	139.03	9.73	45.70	47.08	7.23		
164	164	932	4/18/2015	34.39311	-94.33522	37	90	1	11	0	345.52	6100.98	140.86	8.83	45.70	47.08	7.23		
164	164	933	4/18/2015	34.39311	-94.33522	24	95	1	10	0	345.96	6084.59	143.26	6.37	45.70	47.08	7.23		
164	164	934	4/18/2015	34.39311	-94.33522	8	95	1	14	0	346.23	6077.47	145.98	7.65	45.70	47.08	7.23		
164	164	935	4/18/2015	34.39311	-94.33522	0	97	1	7	0	346.59	6076.64	149.33	6.26	45.70	47.08	7.23		
166	166	936	4/14/2015	34.65028	-94.16457	12	80	1	27	3	333.51	6087.30	108.98	7.48	36.14	52.07	11.79	*P. reimeri*	
166	166	937	4/14/2015	34.65028	-94.16457	1	88	1	47	2	333.03	6090.12	99.04	7.94	36.14	52.07	11.79	*P. reimeri*	
166	166	938	4/14/2015	34.65028	-94.16457	0	90	1	17	1	332.66	6092.11	89.20	9.50	36.14	52.07	11.79	*P. reimeri*	
166	166	939	4/14/2015	34.65028	-94.16457	0	75	1	46	1	332.32	6092.59	79.31	10.01	36.14	52.07	11.79	*P. reimeri*	
166	166	940	4/14/2015	34.65028	-94.16457	0	90	1	32	1	332.10	6094.76	69.49	10.05	36.14	52.07	11.79	*P. reimeri*	
166	166	941	4/14/2015	34.65028	-94.16457	0	70	1	42	0	331.73	6095.21	59.78	8.20	36.14	52.07	11.79		
167	167	942	4/11/2015	34.53557	-94.22560	0	35	1	20	1	344.11	6096.20	105.09	11.33	12.52	70.16	17.31	*P. reimeri*	
167	167	943	4/11/2015	34.53557	-94.22560	0	45	1	14	1	344.04	6096.26	107.12	7.83	12.52	70.16	17.31	*P. reimeri*	
167	167	944	4/11/2015	34.53557	-94.22560	0	65	1	7	1	343.92	6095.70	109.82	9.15	12.52	70.16	17.31	*P. reimeri*	
167	167	945	4/11/2015	34.53557	-94.22560	0	65	1	18	0	343.77	6095.52	113.30	9.47	12.52	70.16	17.31		
167	167	946	4/11/2015	34.53557	-94.22560	0	55	1	11	0	343.68	6096.08	117.12	8.19	12.52	70.16	17.31		
167	167	947	4/11/2015	34.53557	-94.22560	0	45	1	27	0	343.52	6095.09	121.51	7.25	12.52	70.16	17.31		
170	170	948	4/15/2015	34.60364	-94.20482	0	45	1	19	4	336.64	6103.95	139.87	7.23	33.99	54.64	11.36	*P. reimeri*	
170	170	949	4/15/2015	34.60364	-94.20482	0	65	1	22	2	336.32	6105.98	143.95	7.49	33.99	54.64	11.36	*P. reimeri*	
170	170	950	4/15/2015	34.60364	-94.20482	0	70	1	27	2	335.92	6106.37	148.48	7.73	33.99	54.64	11.36	*P. reimeri*	
170	170	951	4/15/2015	34.60364	-94.20482	1	65	1	16	1	335.49	6104.36	153.18	8.92	33.99	54.64	11.36	*P. reimeri*	
170	170	952	4/15/2015	34.60364	-94.20482	2	80	1	19	1	335.33	6100.18	157.87	11.33	33.99	54.64	11.36	*P. reimeri*	
170	170	953	4/15/2015	34.60364	-94.20482	3	45	1	13	0	335.44	6099.15	162.59	7.57	33.99	54.64	11.36		
172	172	954	4/17/2015	34.59108	-94.36240	1	95	0	16	0	319.80	6095.80	403.50	7.73	40.99	47.79	11.21		
172	172	955	4/17/2015	34.59108	-94.36240	4	87	1	9	0	319.71	6094.38	408.57	7.06	40.99	47.79	11.21		
172	172	956	4/17/2015	34.59108	-94.36240	20	90	0	13	0	319.55	6091.48	413.68	7.21	40.99	47.79	11.21		

Table D.1 cont'd

Site	Transect	Quadrat	Date	Latitude	Longitude	Canopy	Herb	Sedge	Stem	Burrow	Elevation	Solar	Water_dist	CTI	Sand	Silt	Clay	Burrow ownership	Water_trns
172	172	957	4/17/2015	34.59108	-94.36240	20	90	1	64	0	319.37	6091.11	418.90	7.60	40.99	47.79	11.21		
172	172	958	4/17/2015	34.59108	-94.36240	25	90	1	9	0	319.28	6091.67	424.02	7.73	40.99	47.79	11.21		
172	172	959	4/17/2015	34.59108	-94.36240	41	85	1	16	0	319.19	6091.65	429.34	7.75	40.99	47.79	11.21		
174	174	960	4/10/2015	34.59349	-94.32080	0	85	0	27	0	335.91	6097.06	102.73	8.80	37.67	51.73	10.60		
174	174	961	4/10/2015	34.59349	-94.32080	0	90	0	10	0	335.74	6094.72	92.77	8.67	37.67	51.73	10.60		
174	174	962	4/10/2015	34.59349	-94.32080	0	85	0	37	0	335.31	6091.80	82.83	8.51	37.67	51.73	10.60		
174	174	963	4/10/2015	34.59349	-94.32080	0	90	0	25	0	334.88	6095.24	72.91	8.84	37.67	51.73	10.60		
174	174	964	4/10/2015	34.59349	-94.32080	0	80	0	21	0	334.66	6098.22	63.01	12.44	37.67	51.73	10.60		
174	174	965	4/10/2015	34.59349	-94.32080	0	90	0	7	0	334.42	6097.53	53.13	8.01	37.67	51.73	10.60		
175	175	966	4/18/2015	34.43276	-94.46193	13	78	0	24	2	322.95	6092.07	492.64	8.95	13.11	73.70	13.19	*P. reimeri*	
175	175	967	4/18/2015	34.43276	-94.46193	4	85	0	19	0	322.78	6090.96	483.71	9.14	13.11	73.70	13.19		
175	175	968	4/18/2015	34.43276	-94.46193	2	80	0	23	0	322.61	6089.95	474.78	10.12	13.11	73.70	13.19		
175	175	969	4/18/2015	34.43276	-94.46193	8	90	0	22	0	322.46	6089.94	466.02	9.66	13.11	73.70	13.19		
175	175	970	4/18/2015	34.43276	-94.46193	12	83	0	13	0	322.00	6090.88	457.18	10.15	13.11	73.70	13.19		
175	175	971	4/18/2015	34.43276	-94.46193	9	90	0	6	0	321.96	6094.79	448.44	11.46	13.11	73.70	13.19		
178	178	972	4/18/2015	34.35961	-94.37667	0	98	0	32	0	360.66	6113.61	263.95	9.07	28.46	55.12	16.42		
178	178	973	4/18/2015	34.35961	-94.37667	0	97	0	17	0	360.65	6113.04	263.91	8.80	28.46	55.12	16.42		
178	178	974	4/18/2015	34.35961	-94.37667	0	99	0	21	0	360.64	6113.16	263.88	8.86	28.46	55.12	16.42		
178	178	975	4/18/2015	34.35961	-94.37667	0	100	0	17	0	360.65	6113.28	263.84	8.80	28.46	55.12	16.42		
178	178	976	4/18/2015	34.35961	-94.37667	0	97	0	17	0	360.58	6112.64	263.92	8.18	28.46	55.12	16.42		
178	178	977	4/18/2015	34.35961	-94.37667	0	98	0	12	0	360.50	6112.08	263.89	9.66	28.46	55.12	16.42		
180	180	978	4/14/2015	34.72329	-94.23804	70	45	0	7	0	341.56	6088.55	67.76	11.24	43.01	49.47	7.52		
180	180	979	4/14/2015	34.72329	-94.23804	56	35	1	12	0	341.64	6091.15	74.53	11.66	43.01	49.47	7.52		
180	180	980	4/14/2015	34.72329	-94.23804	1	65	0	6	0	341.86	6091.34	81.50	7.94	43.01	49.47	7.52		
180	180	981	4/14/2015	34.72329	-94.23804	5	80	1	14	0	341.92	6092.15	88.71	8.14	43.01	49.47	7.52		
180	180	982	4/14/2015	34.72329	-94.23804	12	88	0	16	0	341.97	6092.13	96.09	11.77	43.01	49.47	7.52		
180	180	983	4/14/2015	34.72329	-94.23804	9	90	1	5	0	342.07	6091.99	103.41	11.70	43.01	49.47	7.52		

Table D.1 cont'd

Site	Transect	Quadrat	Date	Latitude	Longitude	Canopy	Herb	Sedge	Stem	Burrow	Elevation	Solar	Water_dist	CTI	Sand	Silt	Clay	Burrow ownership	Water_trns
182	182	984	4/10/2015	34.59201	-94.28992	0	45	1	57	2	334.40	6094.97	230.52	9.80	37.67	51.73	10.60		
182	182	985	4/10/2015	34.59201	-94.28992	4	75	1	37	0	334.56	6092.85	235.50	9.38	37.67	51.73	10.60		
182	182	986	4/10/2015	34.59201	-94.28992	9	30	1	16	0	334.82	6089.92	229.68	8.52	37.67	51.73	10.60		
182	182	987	4/10/2015	34.59201	-94.28992	12	78	1	24	0	335.08	6075.46	223.97	7.13	37.67	51.73	10.60		
182	182	988	4/10/2015	34.59201	-94.28992	21	90	0	18	0	336.27	6083.15	218.50	5.85	37.67	51.73	10.60		
182	182	989	4/10/2015	34.59201	-94.28992	12	85	0	46	0	336.90	6099.95	213.36	8.08	37.67	51.73	10.60		
186	186	990	4/2/2015	34.33675	-93.98843	4	90	0	37	0	323.47	6090.94	90.14	7.76	30.88	55.90	13.22		
186	186	991	4/2/2015	34.33675	-93.98843	4	95	0	27	0	323.65	6092.44	93.93	7.96	30.88	55.90	13.22		
186	186	992	4/2/2015	34.33675	-93.98843	0	85	0	31	0	323.79	6089.52	98.13	7.70	30.88	55.90	13.22		
186	186	993	4/2/2015	34.33675	-93.98843	9	100	0	32	0	323.83	6089.71	103.04	8.45	30.88	55.90	13.22		
186	186	994	4/2/2015	34.33675	-93.98843	100	15	0	0	0	323.86	6088.00	106.32	9.28	30.88	55.90	13.22		
186	186	995	4/2/2015	34.33675	-93.98843	40	35	0	16	0	323.87	6087.17	96.27	9.58	30.88	55.90	13.22		
187	187	996	4/21/2015	34.16070	-94.41533	89	5	0	2	0	384.51	6129.44	142.54	9.33	34.00	54.29	11.71		
187	187	997	4/21/2015	34.16070	-94.41533	100	5	0	0	0	384.58	6130.74	138.68	8.70	34.00	54.29	11.71		
187	187	998	4/21/2015	34.16070	-94.41533	53	15	0	8	0	384.82	6131.70	135.46	8.95	34.00	54.29	11.71		
187	187	999	4/21/2015	34.16070	-94.41533	43	65	0	13	0	384.89	6130.13	132.90	8.38	34.00	54.29	11.71		
187	187	1000	4/21/2015	34.16070	-94.41533	90	20	0	0	0	384.88	6129.56	130.99	9.47	34.00	54.29	11.71		
187	187	1001	4/21/2015	34.16070	-94.41533	25	55	0	9	0	384.95	6128.73	129.96	12.76	34.00	54.29	11.71		
188	188	1002	4/16/2015	34.46184	-93.90311	46	85	0	12	0	353.10	6093.93	38.95	7.75	14.56	70.84	14.61		
188	188	1003	4/16/2015	34.46184	-93.90311	40	88	0	19	0	352.78	6094.68	40.83	7.75	14.56	70.84	14.61		
188	188	1004	4/16/2015	34.46184	-93.90311	20	90	0	25	0	352.22	6095.19	42.98	8.24	14.56	70.84	14.61		
188	188	1005	4/16/2015	34.46184	-93.90311	3	97	1	22	0	352.06	6095.44	46.00	8.36	14.56	70.84	14.61		
188	188	1006	4/16/2015	34.46184	-93.90311	31	87	0	20	0	351.85	6095.10	49.04	8.36	14.56	70.84	14.61		
188	188	1007	4/16/2015	34.46184	-93.90311	41	83	0	21	0	351.46	6092.62	53.08	9.23	14.56	70.84	14.61		
190	190	1008	4/14/2015	34.65643	-94.13260	0	90	0	27	0	337.77	6094.20	78.65	8.18	36.14	52.07	11.79		
190	190	1009	4/14/2015	34.65643	-94.13260	0	95	0	28	0	338.10	6093.85	88.64	6.79	36.14	52.07	11.79		
190	190	1010	4/14/2015	34.65643	-94.13260	0	95	0	23	0	338.36	6094.56	98.62	8.95	36.14	52.07	11.79		

Table D.1 cont'd

Site	Transect	Quadrat	Date	Latitude	Longitude	Canopy	Herb	Sedge	Stem	Burrow	Elevation	Solar	Water_dist	CTI	Sand	Silt	Clay	Burrow ownership	Water_trns
190	190	1011	4/14/2015	34.65643	-94.13260	25	85	0	27	0	338.69	6094.03	89.50	8.55	36.14	52.07	11.79		
190	190	1012	4/14/2015	34.65643	-94.13260	98	65	0	32	0	339.17	6085.93	79.70	6.83	36.14	52.07	11.79		
190	190	1013	4/14/2015	34.65643	-94.13260	32	35	0	18	0	339.99	6084.82	69.93	6.08	36.14	52.07	11.79		
195	195	1014	4/9/2015	34.55227	-94.28708	0	88	0	58	0	335.57	6095.05	600.17	7.80	24.99	63.65	11.35		
195	195	1015	4/9/2015	34.55227	-94.28708	0	85	0	78	0	335.43	6094.60	601.73	9.03	24.99	63.65	11.35		
195	195	1016	4/9/2015	34.55227	-94.28708	0	95	0	32	0	335.39	6094.35	603.35	13.29	24.99	63.65	11.35		
195	195	1017	4/9/2015	34.55227	-94.28708	0	95	0	52	0	335.39	6094.45	605.13	10.29	24.99	63.65	11.35		
195	195	1018	4/9/2015	34.55227	-94.28708	0	99	0	56	0	335.39	6095.34	607.00	8.73	24.99	63.65	11.35		
195	195	1019	4/9/2015	34.55227	-94.28708	4	98	0	49	0	335.47	6096.80	608.87	7.96	24.99	63.65	11.35		
197	197	1020	4/18/2015	34.37649	-94.38387	0	87	0	4	2	330.07	6050.43	630.76	7.77	28.46	55.12	16.42	*P. reimeri*	
197	197	1021	4/18/2015	34.37649	-94.38387	0	85	0	11	0	329.42	6064.75	624.01	8.26	28.46	55.12	16.42		
197	197	1022	4/18/2015	34.37649	-94.38387	0	78	0	8	0	328.96	6072.55	617.48	9.46	28.46	55.12	16.42		
197	197	1023	4/18/2015	34.37649	-94.38387	0	65	0	3	0	328.72	6077.65	610.91	9.78	28.46	55.12	16.42		
197	197	1024	4/18/2015	34.37649	-94.38387	0	85	1	7	0	328.46	6082.26	604.52	7.27	28.46	55.12	16.42		
197	197	1025	4/18/2015	34.37649	-94.38387	0	98	0	16	0	328.36	6083.27	598.19	7.31	28.46	55.12	16.42		
198	198	1026	4/15/2015	34.61970	-94.10235	0	35	0	3	0	332.86	6093.08	90.91	9.26	31.30	52.55	16.15		
198	198	1027	4/15/2015	34.61970	-94.10235	0	95	1	17	0	332.86	6091.96	88.45	6.32	31.30	52.55	16.15		
198	198	1028	4/15/2015	34.61970	-94.10235	0	98	0	11	0	332.60	6090.78	87.18	7.24	31.30	52.55	16.15		
198	198	1029	4/15/2015	34.61970	-94.10235	1	97	1	32	0	332.37	6082.16	86.62	9.28	31.30	52.55	16.15		
198	198	1030	4/15/2015	34.61970	-94.10235	6	98	1	22	0	332.31	6073.00	87.19	6.95	31.30	52.55	16.15		
198	198	1031	4/15/2015	34.61970	-94.10235	43	97	0	27	0	332.28	6066.16	88.15	7.75	31.30	52.55	16.15		
200	200	1032	4/9/2015	34.56834	-94.25409	0	70	0	18	0	347.16	6104.49	281.88	8.77	33.99	54.64	11.36		
200	200	1033	4/9/2015	34.56834	-94.25409	0	95	0	32	0	347.08	6104.98	287.83	7.84	33.99	54.64	11.36		
200	200	1034	4/9/2015	34.56834	-94.25409	0	95	0	42	0	347.03	6105.17	293.94	8.48	33.99	54.64	11.36		
200	200	1035	4/9/2015	34.56834	-94.25409	0	98	0	38	0	347.00	6104.60	300.32	10.93	33.99	54.64	11.36		
200	200	1036	4/9/2015	34.56834	-94.25409	0	99	0	47	0	347.02	6100.45	306.90	11.48	33.99	54.64	11.36		
200	200	1037	4/9/2015	34.56834	-94.25409	0	95	0	28	0	347.24	6096.77	313.66	8.84	33.99	54.64	11.36		

Table D.1 cont'd

Site	Transect	Quadrat	Date	Latitude	Longitude	Canopy	Herb	Sedge	Stem	Burrow	Elevation	Solar	Water_dist	CTI	Sand	Silt	Clay	Burrow ownership	Water_trns
201	201	1038	4/11/2015	34.50230	-94.02217	0	90	1	13	0	325.26	6062.69	128.03	8.62	23.47	64.09	12.44		
201	201	1039	4/11/2015	34.50230	-94.02217	0	55	0	11	0	324.58	6041.73	134.27	10.03	23.47	64.09	12.44		
201	201	1040	4/11/2015	34.50230	-94.02217	0	90	0	24	0	324.58	6041.73	140.93	10.03	23.47	64.09	12.44		
201	201	1041	4/11/2015	34.50230	-94.02217	22	85	1	16	0	324.67	6025.75	147.92	6.78	23.47	64.09	12.44		
201	201	1042	4/11/2015	34.50230	-94.02217	32	95	0	24	0	325.68	6008.29	155.29	6.59	23.47	64.09	12.44		
201	201	1043	4/11/2015	34.50230	-94.02217	40	90	1	18	0	326.81	6002.90	162.83	6.12	23.47	64.09	12.44		
204	204	1044	4/21/2015	34.32547	-94.38884	0	90	1	28	0	330.76	6084.87	89.82	10.82	34.00	54.29	11.71		
204	204	1045	4/21/2015	34.32547	-94.38884	0	95	0	22	0	330.80	6093.35	85.62	9.06	34.00	54.29	11.71		
204	204	1046	4/21/2015	34.32547	-94.38884	4	97	1	32	0	331.17	6092.14	82.41	6.15	34.00	54.29	11.71		
204	204	1047	4/21/2015	34.32547	-94.38884	3	95	1	27	0	331.52	6094.89	78.89	6.45	34.00	54.29	11.71		
204	204	1048	4/21/2015	34.32547	-94.38884	8	90	1	26	0	330.88	6096.14	73.55	6.28	34.00	54.29	11.71		
204	204	1049	4/21/2015	34.32547	-94.38884	4	98	0	28	0	330.85	6092.27	68.11	6.71	34.00	54.29	11.71		
205	205	1050	4/11/2015	34.58804	-94.26631	15	60	1	34	4	348.45	6105.15	180.40	10.28	37.67	51.73	10.60	*P. reimeri*	
205	205	1051	4/11/2015	34.58804	-94.26631	11	45	1	41	4	348.49	6105.18	183.18	8.14	37.67	51.73	10.60	*P. reimeri*	
205	205	1052	4/11/2015	34.58804	-94.26631	16	65	1	30	3	348.49	6105.30	186.33	10.08	37.67	51.73	10.60	*P. reimeri*	
205	205	1053	4/11/2015	34.58804	-94.26631	9	55	1	7	2	348.58	6106.05	187.88	7.77	37.67	51.73	10.60	*P. reimeri*	
205	205	1054	4/11/2015	34.58804	-94.26631	10	50	1	41	2	348.61	6106.77	188.34	7.64	37.67	51.73	10.60	*P. reimeri*	
205	205	1055	4/11/2015	34.58804	-94.26631	0	65	1	21	1	348.54	6107.48	189.21	8.05	37.67	51.73	10.60	*P. reimeri*	
206	206	1056	4/11/2015	34.58151	-94.22252	0	99	0	118	0	329.98	6093.57	171.49	8.14	33.99	54.64	11.36		
206	206	1057	4/11/2015	34.58151	-94.22252	0	100	0	114	0	329.98	6093.80	164.22	8.50	33.99	54.64	11.36		
206	206	1058	4/11/2015	34.58151	-94.22252	0	100	1	96	0	329.97	6094.73	157.32	10.12	33.99	54.64	11.36		
206	206	1059	4/11/2015	34.58151	-94.22252	0	100	0	84	0	329.90	6094.95	150.61	11.04	33.99	54.64	11.36		
206	206	1060	4/11/2015	34.58151	-94.22252	0	87	1	88	0	329.90	6094.04	143.87	10.77	33.99	54.64	11.36		
206	206	1061	4/11/2015	34.58151	-94.22252	0	100	0	110	0	329.93	6093.38	137.57	8.24	33.99	54.64	11.36		
207	207	1062	4/14/2015	34.65010	-94.16549	12	65	1	21	0	336.45	6090.78	186.58	6.53	36.14	52.07	11.79		
207	207	1063	4/14/2015	34.65010	-94.16549	17	75	1	24	0	336.18	6091.54	176.60	6.96	36.14	52.07	11.79		
207	207	1064	4/14/2015	34.65010	-94.16549	15	55	1	21	0	335.76	6095.30	166.64	6.47	36.14	52.07	11.79		

Table D.1 cont'd

Site	Transect	Quadrat	Date	Latitude	Longitude	Canopy	Herb	Sedge	Stem	Burrow	Elevation	Solar	Water_dist	CTI	Sand	Silt	Clay	Burrow ownership	Water_trns
207	207	1065	4/14/2015	34.65010	-94.16549	22	45	1	10	0	335.19	6099.94	156.57	7.60	36.14	52.07	11.79		
207	207	1066	4/14/2015	34.65010	-94.16549	7	95	1	14	0	335.08	6102.84	146.59	7.31	36.14	52.07	11.79		
207	207	1067	4/14/2015	34.65010	-94.16549	6	95	1	37	0	335.01	6103.02	136.60	7.29	36.14	52.07	11.79		
211	211	1068	4/12/2015	34.49381	-93.90891	100	10	0	0	0	306.86	6077.92	47.25	8.95	14.56	70.84	14.61		
211	211	1069	4/12/2015	34.49381	-93.90891	66	15	0	0	0	307.04	6076.74	54.48	7.75	14.56	70.84	14.61		
211	211	1070	4/12/2015	34.49381	-93.90891	88	35	1	0	0	307.10	6074.59	62.15	8.06	14.56	70.84	14.61		
211	211	1071	4/12/2015	34.49381	-93.90891	47	70	1	23	0	307.33	6073.61	70.34	7.53	14.56	70.84	14.61		
211	211	1072	4/12/2015	34.49381	-93.90891	73	60	0	7	0	307.26	6072.11	78.91	9.42	14.56	70.84	14.61		
211	211	1073	4/12/2015	34.49381	-93.90891	51	100	1	19	0	307.23	6071.94	87.74	10.10	14.56	70.84	14.61		
213	213	1074	4/14/2015	34.64357	-94.19765	59	88	1	22	0	327.27	6089.95	56.88	7.77	36.14	52.07	11.79		
213	213	1075	4/14/2015	34.64357	-94.19765	98	95	1	36	0	327.13	6088.71	60.28	7.28	36.14	52.07	11.79		
213	213	1076	4/14/2015	34.64357	-94.19765	35	85	0	27	0	326.74	6093.04	55.91	7.45	36.14	52.07	11.79		
213	213	1077	4/14/2015	34.64357	-94.19765	27	90	1	17	0	326.58	6094.14	48.50	7.26	36.14	52.07	11.79		
213	213	1078	4/14/2015	34.64357	-94.19765	89	85	0	21	0	326.35	6094.77	39.32	7.57	36.14	52.07	11.79		
213	213	1079	4/14/2015	34.64357	-94.19765	53	95	0	24	0	326.15	6094.93	30.55	7.16	36.14	52.07	11.79		
214	214	1080	4/17/2015	34.43245	-94.34029	31	70	0	18	0	337.10	6080.77	162.08	8.59	42.17	49.05	8.78		
214	214	1081	4/17/2015	34.43245	-94.34029	30	80	0	6	0	337.10	6081.92	153.27	7.15	42.17	49.05	8.78		
214	214	1082	4/17/2015	34.43245	-94.34029	23	35	0	3	0	337.08	6087.24	144.61	6.97	42.17	49.05	8.78		
214	214	1083	4/17/2015	34.43245	-94.34029	42	45	0	11	0	336.98	6095.90	136.09	6.69	42.17	49.05	8.78		
214	214	1084	4/17/2015	34.43245	-94.34029	31	70	0	0	0	336.78	6100.21	127.83	8.49	42.17	49.05	8.78		
214	214	1085	4/17/2015	34.43245	-94.34029	24	20	3	0	0	336.62	6100.98	119.84	8.94	42.17	49.05	8.78		
216	216	1086	4/10/2015	34.63903	-94.41092	0	67	1	47	3	303.15	6083.77	258.03	8.13	23.42	62.71	13.87	*P. reimeri*	
216	216	1087	4/10/2015	34.63903	-94.41092	4	60	1	10	3	303.15	6083.70	261.41	8.12	23.42	62.71	13.87	*P. reimeri*	
216	216	1088	4/10/2015	34.63903	-94.41092	1	98	1	42	1	302.32	6082.44	264.60	8.22	23.42	62.71	13.87	*P. reimeri*	
216	216	1089	4/10/2015	34.63903	-94.41092	24	80	1	28	1	301.81	6081.68	267.94	8.61	23.42	62.71	13.87	*P. reimeri*	
216	216	1090	4/10/2015	34.63903	-94.41092	45	80	1	36	1	301.88	6081.46	271.53	8.75	23.42	62.71	13.87	*P. reimeri*	
216	216	1091	4/10/2015	34.63903	-94.41092	60	45	1	52	1	301.45	6078.69	275.56	10.53	23.42	62.71	13.87	*P. reimeri*	

Table D.1 cont'd

Site	Transect	Quadrat	Date	Latitude	Longitude	Canopy	Herb	Sedge	Stem	Burrow	Elevation	Solar	Water_dist	CTI	Sand	Silt	Clay	Burrow ownership	Water_trns
221	221	1092	4/19/2015	34.35120	-94.35957	17	25	1	4	2	359.12	6103.89	126.75	7.80	45.70	47.08	7.23		
221	221	1093	4/19/2015	34.35120	-94.35957	23	65	1	11	1	359.52	6095.68	123.29	7.19	45.70	47.08	7.23		
221	221	1094	4/19/2015	34.35120	-94.35957	12	77	1	21	0	360.44	6101.30	120.58	6.80	45.70	47.08	7.23		
221	221	1095	4/19/2015	34.35120	-94.35957	15	87	1	19	0	360.72	6111.65	118.63	6.97	45.70	47.08	7.23		
221	221	1096	4/19/2015	34.35120	-94.35957	13	80	1	10	0	360.57	6106.22	117.13	6.98	45.70	47.08	7.23		
221	221	1097	4/19/2015	34.35120	-94.35957	15	85	1	8	0	360.27	6100.05	115.66	7.52	45.70	47.08	7.23		
222	222	1098	4/10/2015	34.56499	-94.40951	0	55	0	22	0	323.46	6097.77	379.35	7.74	25.36	64.04	10.60		
222	222	1099	4/10/2015	34.56499	-94.40951	0	70	0	16	0	323.79	6097.17	370.92	7.15	25.36	64.04	10.60		
222	222	1100	4/10/2015	34.56499	-94.40951	0	65	0	13	0	323.88	6094.42	362.57	8.14	25.36	64.04	10.60		
222	222	1101	4/10/2015	34.56499	-94.40951	0	70	0	39	0	324.04	6092.73	354.14	7.58	25.36	64.04	10.60		
222	222	1102	4/10/2015	34.56499	-94.40951	0	90	0	32	0	323.94	6092.21	345.79	9.42	25.36	64.04	10.60		
222	222	1103	4/10/2015	34.56499	-94.40951	0	85	0	32	0	324.06	6094.30	337.40	8.40	25.36	64.04	10.60		
223	223	1104	4/16/2015	34.59415	-93.94822	2	15	0	14	0	317.98	6090.08	35.02	6.63	22.14	65.75	12.11		
223	223	1105	4/16/2015	34.59415	-93.94822	12	35	0	27	0	318.15	6087.11	36.12	6.79	22.14	65.75	12.11		
223	223	1106	4/16/2015	34.59415	-93.94822	13	85	0	24	0	318.33	6088.51	37.32	6.94	22.14	65.75	12.11		
223	223	1107	4/16/2015	34.59415	-93.94822	22	90	0	18	0	318.42	6087.00	38.43	7.17	22.14	65.75	12.11		
223	223	1108	4/16/2015	34.59415	-93.94822	100	5	0	0	0	318.42	6085.32	39.83	7.38	22.14	65.75	12.11		
223	223	1109	4/16/2015	34.59415	-93.94822	81	75	0	11	0	318.42	6086.75	41.64	7.41	22.14	65.75	12.11		
227	227	1110	4/10/2015	34.64431	-94.45112	0	70	1	37	0	304.94	6065.24	13.50	8.43	23.42	62.71	13.87		
227	227	1111	4/10/2015	34.64431	-94.45112	0	80	0	29	0	305.21	6069.18	23.48	8.85	23.42	62.71	13.87		
227	227	1112	4/10/2015	34.64431	-94.45112	0	90	0	76	0	305.46	6069.38	33.45	8.70	23.42	62.71	13.87		
227	227	1113	4/10/2015	34.64431	-94.45112	0	85	1	31	0	305.70	6067.55	43.42	7.81	23.42	62.71	13.87		
227	227	1114	4/10/2015	34.64431	-94.45112	0	95	1	54	0	306.20	6069.57	53.30	7.41	23.42	62.71	13.87		
227	227	1115	4/10/2015	34.64431	-94.45112	0	65	1	38	0	306.46	6072.64	63.28	7.10	23.42	62.71	13.87		
229	229	1116	4/17/2015	34.48272	-94.39660	2	80	0	21	0	301.56	6083.85	281.25	8.27	15.85	64.74	19.40		
229	229	1117	4/17/2015	34.48272	-94.39660	1	70	0	17	0	301.54	6083.97	280.19	8.26	15.85	64.74	19.40		
229	229	1118	4/17/2015	34.48272	-94.39660	10	85	0	31	0	301.54	6084.56	279.19	9.78	15.85	64.74	19.40		

Table D.1 cont'd

Site	Transect	Quadrat	Date	Latitude	Longitude	Canopy	Herb	Sedge	Stem	Burrow	Elevation	Solar	Water_dist	CTI	Sand	Silt	Clay	Burrow ownership	Water_trns
229	229	1119	4/17/2015	34.48272	-94.39660	41	80	0	12	0	301.53	6085.16	278.51	8.27	15.85	64.74	19.40		
229	229	1120	4/17/2015	34.48272	-94.39660	36	87	0	11	0	301.53	6085.26	278.07	6.59	15.85	64.74	19.40		
229	229	1121	4/17/2015	34.48272	-94.39660	5	90	0	21	0	301.31	6084.34	277.73	11.32	15.85	64.74	19.40		
234	234	1122	4/10/2015	34.59190	-94.29022	0	75	1	32	1	333.54	6102.55	172.79	8.32	37.67	51.73	10.60		
234	234	1123	4/10/2015	34.59190	-94.29022	20	70	1	27	0	333.90	6104.13	164.80	7.83	37.67	51.73	10.60		
234	234	1124	4/10/2015	34.59190	-94.29022	20	95	1	37	0	333.83	6103.68	156.87	6.73	37.67	51.73	10.60		
234	234	1125	4/10/2015	34.59190	-94.29022	4	80	1	25	0	333.69	6103.19	148.80	8.39	37.67	51.73	10.60		
234	234	1126	4/10/2015	34.59190	-94.29022	0	99	0	42	0	333.60	6102.79	140.81	6.84	37.67	51.73	10.60		
234	234	1127	4/10/2015	34.59190	-94.29022	0	95	0	46	0	333.45	6102.28	132.81	8.25	37.67	51.73	10.60		
236	236	1128	4/18/2015	34.39311	-94.33360	0	96	1	16	2	344.95	6111.40	168.01	8.26	45.70	47.08	7.23	*P. reimeri*	
236	236	1129	4/18/2015	34.39311	-94.33360	0	97	1	23	0	345.09	6111.67	166.14	9.11	45.70	47.08	7.23		
236	236	1130	4/18/2015	34.39311	-94.33360	0	90	1	23	0	344.99	6111.92	164.98	8.75	45.70	47.08	7.23		
236	236	1131	4/18/2015	34.39311	-94.33360	17	95	1	22	0	344.88	6111.94	164.30	8.86	45.70	47.08	7.23		
236	236	1132	4/18/2015	34.39311	-94.33360	38	90	1	27	0	344.91	6111.88	164.23	8.86	45.70	47.08	7.23		
236	236	1133	4/18/2015	34.39311	-94.33360	10	97	1	14	0	345.06	6111.30	164.88	6.73	45.70	47.08	7.23		
237	237	1134	4/17/2015	34.59109	-94.36215	0	87	0	24	0	320.40	6092.66	387.88	7.36	40.99	47.79	11.21		
237	237	1135	4/17/2015	34.59109	-94.36215	0	95	0	29	0	320.53	6093.45	383.92	7.27	40.99	47.79	11.21		
237	237	1136	4/17/2015	34.59109	-94.36215	0	90	0	11	0	320.63	6092.07	379.85	7.51	40.99	47.79	11.21		
237	237	1137	4/17/2015	34.59109	-94.36215	6	65	0	7	0	320.82	6094.51	375.88	6.67	40.99	47.79	11.21		
237	237	1138	4/17/2015	34.59109	-94.36215	0	86	0	16	0	320.93	6096.54	371.82	8.06	40.99	47.79	11.21		
237	237	1139	4/17/2015	34.59109	-94.36215	0	90	1	11	0	321.00	6096.52	367.85	8.40	40.99	47.79	11.21		
238	238	1140	4/12/2015	34.51524	-94.03127	0	100	0	17	0	318.26	6067.73	80.81	8.42	23.47	64.09	12.44		
238	238	1141	4/12/2015	34.51524	-94.03127	0	90	1	21	0	318.26	6067.73	74.33	8.42	23.47	64.09	12.44		
238	238	1142	4/12/2015	34.51524	-94.03127	1	65	1	32	0	318.79	6065.63	68.58	7.35	23.47	64.09	12.44		
238	238	1143	4/12/2015	34.51524	-94.03127	4	13	0	7	0	319.37	6064.75	62.97	6.68	23.47	64.09	12.44		
238	238	1144	4/12/2015	34.51524	-94.03127	0	90	1	33	0	319.46	6066.28	58.07	8.53	23.47	64.09	12.44		
238	238	1145	4/12/2015	34.51524	-94.03127	0	95	0	22	0	320.03	6068.23	54.12	6.69	23.47	64.09	12.44		

Table D.1 cont'd

Site	Transect	Quadrat	Date	Latitude	Longitude	Canopy	Herb	Sedge	Stem	Burrow	Elevation	Solar	Water_dist	CTI	Sand	Silt	Clay	Burrow ownership	Water_trns
239	239	1146	4/2/2015	34.36409	-94.10996	6	20	0	4	0	333.08	6104.82	392.31	6.53	22.59	65.96	11.45		
239	239	1147	4/2/2015	34.36409	-94.10996	44	95	1	32	0	332.69	6102.92	396.72	7.21	22.59	65.96	11.45		
239	239	1148	4/2/2015	34.36409	-94.10996	8	85	0	35	0	332.86	6099.65	401.27	6.84	22.59	65.96	11.45		
239	239	1149	4/2/2015	34.36409	-94.10996	98	78	0	8	0	332.86	6099.65	406.07	6.84	22.59	65.96	11.45		
239	239	1150	4/2/2015	34.36409	-94.10996	100	45	1	2	0	332.57	6097.50	411.00	9.90	22.59	65.96	11.45		
239	239	1151	4/2/2015	34.36409	-94.10996	80	65	1	28	0	332.24	6095.86	416.16	10.18	22.59	65.96	11.45		
241	241	1152	4/15/2015	34.60992	-94.11887	45	75	0	12	0	303.83	6087.03	43.85	8.31	31.30	52.55	16.15		
241	241	1153	4/15/2015	34.60992	-94.11887	43	90	0	19	0	303.75	6087.22	52.17	6.58	31.30	52.55	16.15		
241	241	1154	4/15/2015	34.60992	-94.11887	50	90	1	4	0	303.57	6087.31	60.94	8.17	31.30	52.55	16.15		
241	241	1155	4/15/2015	34.60992	-94.11887	2	95	0	29	0	303.36	6086.77	69.89	7.93	31.30	52.55	16.15		
241	241	1156	4/15/2015	34.60992	-94.11887	89	90	0	32	0	303.09	6086.10	78.85	8.49	31.30	52.55	16.15		
241	241	1157	4/15/2015	34.60992	-94.11887	86	98	1	25	0	302.99	6086.35	87.99	7.56	31.30	52.55	16.15		
243	243	1158	4/12/2015	34.48455	-93.96854	40	100	1	32	0	312.43	6055.25	121.95	7.53	34.00	52.96	13.05		
243	243	1159	4/12/2015	34.48455	-93.96854	85	90	0	26	0	312.84	6054.75	129.03	6.84	34.00	52.96	13.05		
243	243	1160	4/12/2015	34.48455	-93.96854	22	95	1	37	0	313.17	6052.17	136.18	11.39	34.00	52.96	13.05		
243	243	1161	4/12/2015	34.48455	-93.96854	70	85	1	7	0	313.46	6051.17	143.34	7.32	34.00	52.96	13.05		
243	243	1162	4/12/2015	34.48455	-93.96854	60	65	0	7	0	313.84	6047.42	150.43	7.17	34.00	52.96	13.05		
243	243	1163	4/12/2015	34.48455	-93.96854	80	90	1	26	0	314.30	6047.21	157.51	7.14	34.00	52.96	13.05		
245	245	1164	4/9/2015	34.55144	-94.34115	0	0	0	0	0	281.51	6063.31	19.50	7.60	40.99	47.79	11.21		
245	245	1165	4/9/2015	34.55144	-94.34115	0	30	0	16	0	281.50	6062.41	28.11	8.61	40.99	47.79	11.21		
245	245	1166	4/9/2015	34.55144	-94.34115	0	25	1	17	0	281.68	6062.63	37.33	8.34	40.99	47.79	11.21		
245	245	1167	4/9/2015	34.55144	-94.34115	16	20	0	13	0	281.75	6062.12	46.91	13.59	40.99	47.79	11.21		
245	245	1168	4/9/2015	34.55144	-94.34115	52	15	0	4	0	281.75	6062.12	56.46	13.59	40.99	47.79	11.21		
245	245	1169	4/9/2015	34.55144	-94.34115	17	45	0	11	0	281.91	6062.67	65.94	10.50	40.99	47.79	11.21		
247	247	1170	4/9/2015	34.55140	-94.30366	0	85	1	92	4	305.95	6056.27	134.58	7.38	24.99	63.65	11.35	*P. reimeri*	
247	247	1171	4/9/2015	34.55140	-94.30366	0	77	1	27	2	306.10	6052.73	141.86	7.55	24.99	63.65	11.35	*P. reimeri*	
247	247	1172	4/9/2015	34.55140	-94.30366	0	85	1	27	2	306.79	6060.97	149.43	6.61	24.99	63.65	11.35	*P. reimeri*	

Table D.1 cont'd

Site	Transect	Quadrat	Date	Latitude	Longitude	Canopy	Herb	Sedge	Stem	Burrow	Elevation	Solar	Water_dist	CTI	Sand	Silt	Clay	Burrow ownership	Water_trns
247	247	1173	4/9/2015	34.55140	-94.30366	0	86	1	48	1	307.05	6051.87	157.13	6.14	24.99	63.65	11.35	*P. reimeri*	
247	247	1174	4/9/2015	34.55140	-94.30366	8	90	0	64	0	308.10	6060.34	164.84	6.23	24.99	63.65	11.35		
247	247	1175	4/9/2015	34.55140	-94.30366	0	88	1	47	0	308.49	6065.66	172.76	6.59	24.99	63.65	11.35		
249	249	1176	4/14/2015	34.69905	-94.38186	1	78	1	27	0	448.56	6105.86	68.99	6.17	46.46	46.22	7.32		
249	249	1177	4/14/2015	34.69905	-94.38186	0	70	1	24	0	448.32	6105.41	70.92	8.55	46.46	46.22	7.32		
249	249	1178	4/14/2015	34.69905	-94.38186	7	65	1	23	0	448.90	6108.31	72.74	8.17	46.46	46.22	7.32		
249	249	1179	4/14/2015	34.69905	-94.38186	6	85	1	19	0	448.69	6105.18	74.15	6.11	46.46	46.22	7.32		
249	249	1180	4/14/2015	34.69905	-94.38186	7	90	1	14	0	448.80	6104.16	75.60	9.09	46.46	46.22	7.32		
249	249	1181	4/14/2015	34.69905	-94.38186	1	65	1	12	0	448.58	6105.34	75.94	8.36	46.46	46.22	7.32		
251	251	1182	4/15/2015	34.66298	-94.26706	30	90	1	22	0	408.48	6117.65	61.98	12.02	39.35	48.97	11.68		
251	251	1183	4/15/2015	34.66298	-94.26706	35	95	1	35	0	408.56	6111.54	59.02	7.68	39.35	48.97	11.68		
251	251	1184	4/15/2015	34.66298	-94.26706	37	95	1	13	0	408.52	6111.05	56.17	10.15	39.35	48.97	11.68		
251	251	1185	4/15/2015	34.66298	-94.26706	31	98	0	42	0	408.52	6114.16	53.40	10.79	39.35	48.97	11.68		
251	251	1186	4/15/2015	34.66298	-94.26706	42	45	0	9	0	408.58	6113.18	51.24	7.78	39.35	48.97	11.68		
251	251	1187	4/15/2015	34.66298	-94.26706	39	97	0	36	0	408.58	6112.26	49.12	7.77	39.35	48.97	11.68		
253	253	1188	4/11/2015	34.52831	-94.03785	9	65	0	14	0	289.89	6032.45	63.44	11.11	23.47	64.09	12.44		
253	253	1189	4/11/2015	34.52831	-94.03785	7	90	0	37	0	289.68	6038.71	57.87	9.39	23.47	64.09	12.44		
253	253	1190	4/11/2015	34.52831	-94.03785	2	95	0	27	0	289.53	6039.14	52.67	8.94	23.47	64.09	12.44		
253	253	1191	4/11/2015	34.52831	-94.03785	1	88	1	17	0	289.37	6038.74	48.44	8.98	23.47	64.09	12.44		
253	253	1192	4/11/2015	34.52831	-94.03785	2	80	1	39	0	289.25	6040.18	44.99	7.91	23.47	64.09	12.44		
253	253	1193	4/11/2015	34.52831	-94.03785	4	95	0	27	0	289.23	6041.45	42.86	8.89	23.47	64.09	12.44		
255	255	1194	4/15/2015	34.58909	-94.05024	0	95	1	17	0	286.04	6074.95	78.02	7.52	29.06	57.83	13.11		
255	255	1195	4/15/2015	34.58909	-94.05024	0	96	0	14	0	286.32	6072.18	78.37	7.43	29.06	57.83	13.11		
255	255	1196	4/15/2015	34.58909	-94.05024	0	98	0	19	0	286.32	6070.31	79.36	10.94	29.06	57.83	13.11		
255	255	1197	4/15/2015	34.58909	-94.05024	16	100	0	21	0	286.52	6072.22	81.25	9.09	29.06	57.83	13.11		
255	255	1198	4/15/2015	34.58909	-94.05024	43	80	0	19	0	286.71	6072.10	84.33	7.56	29.06	57.83	13.11		
255	255	1199	4/15/2015	34.58909	-94.05024	48	96	0	18	0	286.82	6071.17	88.26	8.56	29.06	57.83	13.11		

Table D.1 cont'd

Site	Transect	Quadrat	Date	Latitude	Longitude	Canopy	Herb	Sedge	Stem	Burrow	Elevation	Solar	Water_dist	CTI	Sand	Silt	Clay	Burrow ownership	Water_trns
257	257	1200	4/18/2015	34.42169	-94.45216	1	65	1	7	0	286.45	6068.99	78.80	7.81	13.11	73.70	13.19		
257	257	1201	4/18/2015	34.42169	-94.45216	0	95	0	22	0	286.45	6069.26	79.47	7.94	13.11	73.70	13.19		
257	257	1202	4/18/2015	34.42169	-94.45216	31	65	0	19	0	286.60	6072.89	75.62	8.29	13.11	73.70	13.19		
257	257	1203	4/18/2015	34.42169	-94.45216	80	20	0	8	0	286.87	6074.51	71.79	7.62	13.11	73.70	13.19		
257	257	1204	4/18/2015	34.42169	-94.45216	62	35	0	6	0	287.24	6074.72	68.01	6.97	13.11	73.70	13.19		
257	257	1205	4/18/2015	34.42169	-94.45216	40	80	0	13	0	287.47	6073.40	65.36	7.15	13.11	73.70	13.19		
259	259	1206	4/15/2015	34.58384	-94.14469	0	65	1	7	0	292.28	6075.70	67.54	7.48	31.67	56.99	11.34		
259	259	1207	4/15/2015	34.58384	-94.14469	0	70	1	6	0	292.19	6075.28	64.47	8.57	31.67	56.99	11.34		
259	259	1208	4/15/2015	34.58384	-94.14469	0	80	1	6	0	292.12	6075.08	62.87	8.25	31.67	56.99	11.34		
259	259	1209	4/15/2015	34.58384	-94.14469	0	78	1	13	0	292.10	6074.37	62.31	7.71	31.67	56.99	11.34		
259	259	1210	4/15/2015	34.58384	-94.14469	0	95	1	11	0	292.13	6072.18	62.25	7.99	31.67	56.99	11.34		
259	259	1211	4/15/2015	34.58384	-94.14469	0	90	1	8	0	292.01	6071.88	61.69	14.66	31.67	56.99	11.34		
261	261	1212	4/2/2015	34.31176	-93.96832	0	30	0	0	0	298.77	6071.29	159.46	8.33	30.88	55.90	13.22		
261	261	1213	4/2/2015	34.31176	-93.96832	0	15	0	3	0	298.77	6074.04	158.23	8.26	30.88	55.90	13.22		
261	261	1214	4/2/2015	34.31176	-93.96832	0	3	0	5	0	298.73	6073.17	157.14	9.58	30.88	55.90	13.22		
261	261	1215	4/2/2015	34.31176	-93.96832	0	15	0	0	0	298.75	6069.67	156.76	7.52	30.88	55.90	13.22		
261	261	1216	4/2/2015	34.31176	-93.96832	0	35	0	27	0	298.38	6056.87	156.94	6.67	30.88	55.90	13.22		
261	261	1217	4/2/2015	34.31176	-93.96832	2	78	0	22	0	297.76	6058.71	157.89	7.34	30.88	55.90	13.22		
263	263	1218	3/23/2015	34.89122	-92.92284	100	0	0	0	0	300.80	6077.18	103.88	8.32	23.21	65.81	10.98		
263	263	1219	3/23/2015	34.89122	-92.92284	100	0	0	0	0	300.78	6077.08	104.92	8.28	23.21	65.81	10.98		
263	263	1220	3/23/2015	34.89122	-92.92284	100	2	0	0	0	300.71	6077.36	106.90	8.21	23.21	65.81	10.98		
263	263	1221	3/23/2015	34.89122	-92.92284	100	2	0	0	0	300.74	6077.49	109.16	8.12	23.21	65.81	10.98		
263	263	1222	3/23/2015	34.89122	-92.92284	100	1	0	0	0	300.80	6077.35	111.47	8.17	23.21	65.81	10.98		
263	263	1223	3/23/2015	34.89122	-92.92284	100	5	1	0	0	300.69	6077.06	114.35	8.04	23.21	65.81	10.98		
265	265	1224	4/15/2015	34.56628	-94.10775	16	80	0	21	0	292.74	6065.88	81.11	8.30	29.06	57.83	13.11		
265	265	1225	4/15/2015	34.56628	-94.10775	32	98	0	20	0	292.74	6065.88	91.00	8.30	29.06	57.83	13.11		
265	265	1226	4/15/2015	34.56628	-94.10775	5	80	0	27	0	292.90	6067.05	100.84	7.73	29.06	57.83	13.11		

Table D.1 cont'd

Site	Transect	Quadrat	Date	Latitude	Longitude	Canopy	Herb	Sedge	Stem	Burrow	Elevation	Solar	Water_dist	CTI	Sand	Silt	Clay	Burrow ownership	Water_trns
265	265	1227	4/15/2015	34.56628	-94.10775	8	97	1	27	0	292.95	6068.87	110.73	9.96	29.06	57.83	13.11		
265	265	1228	4/15/2015	34.56628	-94.10775	11	83	0	42	0	292.95	6069.13	120.68	7.98	29.06	57.83	13.11		
265	265	1229	4/15/2015	34.56628	-94.10775	4	90	1	27	0	292.81	6070.10	130.58	9.12	29.06	57.83	13.11		
267	267	1230	4/19/2015	34.32731	-94.33955	0	98	0	61	0	357.64	6117.54	171.94	6.68	37.95	50.40	11.64		
267	267	1231	4/19/2015	34.32731	-94.33955	0	97	0	72	0	357.64	6117.54	181.89	6.68	37.95	50.40	11.64		
267	267	1232	4/19/2015	34.32731	-94.33955	0	96	0	28	0	357.70	6117.56	191.90	6.59	37.95	50.40	11.64		
267	267	1233	4/19/2015	34.32731	-94.33955	0	96	0	98	0	358.46	6122.41	201.86	8.19	37.95	50.40	11.64		
267	267	1234	4/19/2015	34.32731	-94.33955	0	96	0	86	0	359.07	6122.75	211.87	5.92	37.95	50.40	11.64		
267	267	1235	4/19/2015	34.32731	-94.33955	0	98	0	18	0	359.83	6125.75	221.83	6.15	37.95	50.40	11.64		
269	269	1236	4/15/2015	34.57042	-94.17729	0	85	1	4	1	303.60	6070.99	171.45	8.13	31.67	56.99	11.34	*P. reimeri*	
269	269	1237	4/15/2015	34.57042	-94.17729	1	45	1	6	1	303.65	6071.80	170.67	9.77	31.67	56.99	11.34	*P. reimeri*	
269	269	1238	4/15/2015	34.57042	-94.17729	0	45	1	11	0	303.85	6073.96	170.48	9.98	31.67	56.99	11.34		
269	269	1239	4/15/2015	34.57042	-94.17729	0	80	1	14	0	304.02	6076.33	170.87	9.88	31.67	56.99	11.34		
269	269	1240	4/15/2015	34.57042	-94.17729	0	65	1	15	0	304.26	6077.18	171.84	9.56	31.67	56.99	11.34		
269	269	1241	4/15/2015	34.57042	-94.17729	0	95	0	16	0	304.53	6078.43	173.38	9.66	31.67	56.99	11.34		
271	271	1242	4/14/2015	34.60773	-94.16734	0	90	1	36	2	293.79	6072.51	138.35	8.36	31.67	56.99	11.34	*P. reimeri*	
271	271	1243	4/14/2015	34.60773	-94.16734	0	80	1	23	1	293.61	6072.41	128.40	8.78	31.67	56.99	11.34	*P. reimeri*	
271	271	1244	4/14/2015	34.60773	-94.16734	0	100	1	42	0	293.67	6071.72	118.38	8.45	31.67	56.99	11.34		
271	271	1245	4/14/2015	34.60773	-94.16734	0	95	1	46	0	293.59	6071.93	108.35	8.11	31.67	56.99	11.34		
271	271	1246	4/14/2015	34.60773	-94.16734	0	95	1	33	0	293.50	6071.72	98.38	11.01	31.67	56.99	11.34		
271	271	1247	4/14/2015	34.60773	-94.16734	0	85	1	26	0	293.47	6073.03	88.41	11.64	31.67	56.99	11.34		
273	273	1248	4/17/2015	34.47988	-94.43716	21	75	0	3	0	314.30	6094.29	271.10	6.25	15.85	64.74	19.40		
273	273	1249	4/17/2015	34.47988	-94.43716	31	55	0	0	0	314.50	6094.38	264.66	7.99	15.85	64.74	19.40		
273	273	1250	4/17/2015	34.47988	-94.43716	4	60	0	6	0	314.73	6093.56	258.07	6.58	15.85	64.74	19.40		
273	273	1251	4/17/2015	34.47988	-94.43716	21	70	0	3	0	314.97	6093.75	251.63	6.93	15.85	64.74	19.40		
273	273	1252	4/17/2015	34.47988	-94.43716	30	65	0	4	0	315.07	6093.46	245.20	7.21	15.85	64.74	19.40		
273	273	1253	4/17/2015	34.47988	-94.43716	24	80	0	19	0	315.24	6092.84	238.90	7.19	15.85	64.74	19.40		

Table D.1 cont'd

Site	Transect	Quadrat	Date	Latitude	Longitude	Canopy	Herb	Sedge	Stem	Burrow	Elevation	Solar	Water_dist	CTI	Sand	Silt	Clay	Burrow ownership	Water_trns
275	275	1254	4/11/2015	34.50260	-94.02230	0	45	1	41	0	326.17	6077.60	117.14	6.99	23.47	64.09	12.44		
275	275	1255	4/11/2015	34.50260	-94.02230	0	80	1	6	0	326.17	6083.74	116.69	6.43	23.47	64.09	12.44		
275	275	1256	4/11/2015	34.50260	-94.02230	0	85	1	22	0	325.83	6083.36	117.17	8.67	23.47	64.09	12.44		
275	275	1257	4/11/2015	34.50260	-94.02230	0	80	1	21	0	325.73	6080.80	118.51	7.68	23.47	64.09	12.44		
275	275	1258	4/11/2015	34.50260	-94.02230	1	90	0	24	0	325.27	6077.45	120.67	7.69	23.47	64.09	12.44		
275	275	1259	4/11/2015	34.50260	-94.02230	6	90	1	17	0	325.24	6073.52	123.60	7.60	23.47	64.09	12.44		
277	277	1260	4/21/2015	34.31052	-94.41735	9	65	0	7	0	372.14	6119.89	289.44	8.05	34.00	54.29	11.71		
277	277	1261	4/21/2015	34.31052	-94.41735	5	70	0	5	0	372.14	6118.25	282.79	6.98	34.00	54.29	11.71		
277	277	1262	4/21/2015	34.31052	-94.41735	10	80	0	6	0	372.06	6116.08	276.34	7.61	34.00	54.29	11.71		
277	277	1263	4/21/2015	34.31052	-94.41735	2	65	0	4	0	371.99	6104.17	270.19	7.26	34.00	54.29	11.71		
277	277	1264	4/21/2015	34.31052	-94.41735	11	55	0	7	0	371.35	6089.87	264.20	7.35	34.00	54.29	11.71		
277	277	1265	4/21/2015	34.31052	-94.41735	18	55	0	10	0	370.80	6094.48	258.45	8.87	34.00	54.29	11.71		
279	279	1266	4/14/2015	34.64384	-94.20309	0	85	1	46	2	330.69	6086.65	260.74	9.52	39.35	48.97	11.68	*P. reimeri*	
279	279	1267	4/14/2015	34.64384	-94.20309	0	90	1	49	1	330.74	6085.83	263.74	8.13	39.35	48.97	11.68	*P. reimeri*	
279	279	1268	4/14/2015	34.64384	-94.20309	0	80	0	13	0	330.73	6086.18	263.12	8.95	39.35	48.97	11.68		
279	279	1269	4/14/2015	34.64384	-94.20309	0	75	1	37	0	330.74	6086.96	261.43	9.77	39.35	48.97	11.68		
279	279	1270	4/14/2015	34.64384	-94.20309	0	85	1	36	0	330.74	6086.96	259.97	9.77	39.35	48.97	11.68		
279	279	1271	4/14/2015	34.64384	-94.20309	0	90	1	46	0	330.73	6089.20	258.02	13.05	39.35	48.97	11.68		
282	282	1272	3/24/2015	34.50892	-93.16727	56	95	0	52	0	142.35	5977.61	99.54	7.04	18.31	65.67	16.02		
282	282	1273	3/24/2015	34.50892	-93.16727	60	100	0	48	0	142.10	5982.97	89.57	7.31	18.31	65.67	16.02		
282	282	1274	3/24/2015	34.50892	-93.16727	99	100	0	20	0	141.67	5984.37	79.61	7.28	18.31	65.67	16.02		
282	282	1275	3/24/2015	34.50892	-93.16727	80	100	0	47	0	140.90	5982.19	69.53	7.41	18.31	65.67	16.02		
282	282	1276	3/24/2015	34.50892	-93.16727	60	100	0	25	0	140.16	5981.47	59.56	7.86	18.31	65.67	16.02		
282	282	1277	3/24/2015	34.50892	-93.16727	52	100	0	34	0	139.74	5977.39	49.59	9.37	18.31	65.67	16.02		
283	283	1278	3/23/2015	34.93295	-92.78596	0	56	0	15	0	238.73	6020.07	165.61	6.65	38.71	52.62	8.68		
283	283	1279	3/23/2015	34.93295	-92.78596	0	55	0	33	0	238.99	6036.46	157.85	8.25	38.71	52.62	8.68		
283	283	1280	3/23/2015	34.93295	-92.78596	0	75	0	29	0	238.95	6032.58	150.45	7.49	38.71	52.62	8.68		

Table D.1 cont'd

Site	Transect	Quadrat	Date	Latitude	Longitude	Canopy	Herb	Sedge	Stem	Burrow	Elevation	Solar	Water_dist	CTI	Sand	Silt	Clay	Burrow ownership	Water_trns
283	283	1281	3/23/2015	34.93295	-92.78596	0	25	0	25	0	238.93	6037.53	143.25	8.54	38.71	52.62	8.68		
283	283	1282	3/23/2015	34.93295	-92.78596	0	30	0	5	0	238.88	6032.18	136.42	7.35	38.71	52.62	8.68		
283	283	1283	3/23/2015	34.93295	-92.78596	0	20	0	5	0	238.83	6034.55	130.11	7.73	38.71	52.62	8.68		
287	287	1284	4/2/2015	34.25264	-93.96960	92	5	0	2	0	258.79	6045.18	522.98	7.69	37.65	53.37	8.98		
287	287	1285	4/2/2015	34.25264	-93.96960	96	5	0	2	0	258.38	6040.14	523.92	7.07	37.65	53.37	8.98		
287	287	1286	4/2/2015	34.25264	-93.96960	43	5	0	2	0	259.35	6035.40	525.05	5.28	37.65	53.37	8.98		
287	287	1287	4/2/2015	34.25264	-93.96960	78	48	0	23	0	259.81	6034.74	526.37	5.92	37.65	53.37	8.98		
287	287	1288	4/2/2015	34.25264	-93.96960	76	10	0	0	0	258.54	6030.43	527.87	6.55	37.65	53.37	8.98		
287	287	1289	4/2/2015	34.25264	-93.96960	100	5	0	0	0	258.54	6030.43	529.56	6.55	37.65	53.37	8.98		
288	288	1290	4/3/2015	34.10164	-94.32694	0	80	1	33	0	179.89	5992.97	477.59	7.60	26.65	62.37	10.98		
288	288	1291	4/3/2015	34.10164	-94.32694	0	80	0	57	0	179.09	5989.43	467.64	7.07	26.65	62.37	10.98		
288	288	1292	4/3/2015	34.10164	-94.32694	0	90	1	33	0	178.76	5993.02	457.69	6.82	26.65	62.37	10.98		
288	288	1293	4/3/2015	34.10164	-94.32694	0	80	1	28	0	178.49	5992.70	447.74	6.94	26.65	62.37	10.98		
288	288	1294	4/3/2015	34.10164	-94.32694	0	95	0	54	0	178.25	5985.05	437.72	7.30	26.65	62.37	10.98		
288	288	1295	4/3/2015	34.10164	-94.32694	0	85	0	22	0	177.96	5981.67	427.72	7.26	26.65	62.37	10.98		
290	290	1296	3/24/2015	34.54783	-93.01835	52	30	1	0	0	206.76	5995.25	57.21	6.98	10.29	74.76	14.95		
290	290	1297	3/24/2015	34.54783	-93.01835	28	100	1	36	0	206.73	5992.44	58.76	7.97	10.29	74.76	14.95		
290	290	1298	3/24/2015	34.54783	-93.01835	20	80	1	6	0	206.73	5992.44	60.33	7.97	10.29	74.76	14.95		
290	290	1299	3/24/2015	34.54783	-93.01835	8	100	1	26	0	206.86	5988.72	62.06	7.91	10.29	74.76	14.95		
290	290	1300	3/24/2015	34.54783	-93.01835	16	80	1	7	0	207.49	5986.00	64.57	7.80	10.29	74.76	14.95		
290	290	1301	3/24/2015	34.54783	-93.01835	4	75	1	14	0	207.32	5986.14	67.12	6.28	10.29	74.76	14.95		
292	292	1302	3/25/2015	34.52044	-93.26630	44	100	0	39	0	162.85	5966.66	43.36	7.18	29.70	56.02	14.28		
292	292	1303	3/25/2015	34.52044	-93.26630	52	80	0	42	0	163.53	5970.01	49.59	6.57	29.70	56.02	14.28		
292	292	1304	3/25/2015	34.52044	-93.26630	52	60	0	25	0	163.65	5983.75	55.74	7.07	29.70	56.02	14.28		
292	292	1305	3/25/2015	34.52044	-93.26630	16	100	0	37	0	163.42	5979.59	61.55	8.67	29.70	56.02	14.28		
292	292	1306	3/25/2015	34.52044	-93.26630	12	90	0	12	0	163.77	5982.10	66.88	10.60	29.70	56.02	14.28		
292	292	1307	3/25/2015	34.52044	-93.26630	44	100	0	7	0	164.00	5981.97	71.04	6.49	29.70	56.02	14.28		

Table D.1 cont'd

Site	Transect	Quadrat	Date	Latitude	Longitude	Canopy	Herb	Sedge	Stem	Burrow	Elevation	Solar	Water_dist	CTI	Sand	Silt	Clay	Burrow ownership	Water_trns
294	294	1308	4/3/2015	34.18113	-94.38779	0	65	0	0	0	204.64	5936.70	149.36	7.04	21.14	67.09	11.78		
294	294	1309	4/3/2015	34.18113	-94.38779	0	55	0	6	0	203.84	5939.49	154.88	7.76	21.14	67.09	11.78		
294	294	1310	4/3/2015	34.18113	-94.38779	0	20	0	32	0	203.00	5948.07	160.84	7.38	21.14	67.09	11.78		
294	294	1311	4/3/2015	34.18113	-94.38779	0	65	0	13	0	203.43	5951.59	167.19	6.51	21.14	67.09	11.78		
294	294	1312	4/3/2015	34.18113	-94.38779	0	70	0	12	0	203.02	5953.24	173.90	6.55	21.14	67.09	11.78		
294	294	1313	4/3/2015	34.18113	-94.38779	0	65	0	22	0	203.36	5954.67	180.80	5.87	21.14	67.09	11.78		
296	296	1314	3/28/2015	34.17333	-93.65472	0	85	1	37	1	192.44	5988.88	119.62	7.29	46.90	47.11	5.99		
296	296	1315	3/28/2015	34.17333	-93.65472	0	45	1	12	0	193.23	5960.09	120.40	6.20	46.90	47.11	5.99		
296	296	1316	3/28/2015	34.17333	-93.65472	0	70	0	7	0	194.99	5972.72	121.39	5.58	46.90	47.11	5.99		
296	296	1317	3/28/2015	34.17333	-93.65472	1	25	1	5	0	195.49	6009.04	122.40	6.44	46.90	47.11	5.99		
296	296	1318	3/28/2015	34.17333	-93.65472	1	55	1	27	0	195.76	6014.07	124.00	6.47	46.90	47.11	5.99		
296	296	1319	3/28/2015	34.17333	-93.65472	2	80	1	3	0	195.86	6014.86	126.23	6.40	46.90	47.11	5.99		
297	297	1320	3/25/2015	34.70231	-93.22823	80	35	0	3	0	234.77	5871.49	76.48	6.68	9.07	73.41	17.52		
297	297	1321	3/25/2015	34.70231	-93.22823	100	15	0	1	0	235.74	5961.32	76.45	7.94	9.07	73.41	17.52		
297	297	1322	3/25/2015	34.70231	-93.22823	96	45	0	16	0	234.81	5957.27	76.45	6.63	9.07	73.41	17.52		
297	297	1323	3/25/2015	34.70231	-93.22823	94	15	0	0	0	234.80	5970.32	76.42	6.77	9.07	73.41	17.52		
297	297	1324	3/25/2015	34.70231	-93.22823	80	5	0	0	0	234.76	5972.85	76.38	6.71	9.07	73.41	17.52		
297	297	1325	3/25/2015	34.70231	-93.22823	76	8	0	12	0	233.65	5981.14	76.44	5.77	9.07	73.41	17.52		
300	300	1326	3/30/2015	34.70973	-93.46104	0	70	0	7	0	217.91	6020.78	88.13	7.11	29.07	59.14	11.79		
300	300	1327	3/30/2015	34.70973	-93.46104	1	45	1	62	0	217.78	6023.96	87.35	7.83	29.07	59.14	11.79		
300	300	1328	3/30/2015	34.70973	-93.46104	0	30	1	42	0	217.62	6023.66	87.62	10.38	29.07	59.14	11.79		
300	300	1329	3/30/2015	34.70973	-93.46104	9	70	1	56	0	217.62	6023.66	89.11	10.38	29.07	59.14	11.79		
300	300	1330	3/30/2015	34.70973	-93.46104	25	80	1	27	0	217.49	6021.72	91.31	7.14	29.07	59.14	11.79		
300	300	1331	3/30/2015	34.70973	-93.46104	21	68	1	7	0	217.04	6020.64	94.28	7.20	29.07	59.14	11.79		
301	301	1332	3/26/2015	34.40034	-93.08140	0	100	1	36	0	138.34	5968.42	73.05	9.89	17.79	64.94	17.27		
301	301	1333	3/26/2015	34.40034	-93.08140	0	100	1	52	0	138.34	5968.42	68.32	9.89	17.79	64.94	17.27		
301	301	1334	3/26/2015	34.40034	-93.08140	24	85	1	55	0	138.68	5969.53	64.65	6.69	17.79	64.94	17.27		

Table D.1 cont'd

Site	Transect	Quadrat	Date	Latitude	Longitude	Canopy	Herb	Sedge	Stem	Burrow	Elevation	Solar	Water_dist	CTI	Sand	Silt	Clay	Burrow ownership	Water_trns
301	301	1335	3/26/2015	34.40034	-93.08140	100	100	1	42	0	138.39	5970.81	61.37	7.20	17.79	64.94	17.27		
301	301	1336	3/26/2015	34.40034	-93.08140	75	100	1	38	0	138.54	5969.52	58.13	7.94	17.79	64.94	17.27		
301	301	1337	3/26/2015	34.40034	-93.08140	76	100	0	47	0	138.35	5968.43	55.92	9.81	17.79	64.94	17.27		
302	302	1338	3/30/2015	34.80457	-93.46675	85	5	1	0	0	287.10	6064.50	523.81	8.07	18.77	71.83	9.40		
302	302	1339	3/30/2015	34.80457	-93.46675	100	3	1	0	0	286.96	6063.33	532.24	7.50	18.77	71.83	9.40		
302	302	1340	3/30/2015	34.80457	-93.46675	86	3	0	0	0	286.83	6055.17	540.73	6.88	18.77	71.83	9.40		
302	302	1341	3/30/2015	34.80457	-93.46675	55	15	1	12	0	286.28	6035.59	549.29	6.18	18.77	71.83	9.40		
302	302	1342	3/30/2015	34.80457	-93.46675	30	5	0	0	0	285.99	6037.02	557.97	6.09	18.77	71.83	9.40		
302	302	1343	3/30/2015	34.80457	-93.46675	0	65	1	11	0	284.62	6031.33	566.60	6.72	18.77	71.83	9.40		
307	307	1344	3/27/2015	34.14621	-93.59637	12	65	1	9	3	189.57	6011.37	809.12	9.00	36.12	56.42	7.47		
307	307	1345	3/27/2015	34.14621	-93.59637	8	60	1	7	2	189.34	6011.48	810.98	9.11	36.12	56.42	7.47		
307	307	1346	3/27/2015	34.14621	-93.59637	32	85	1	5	2	189.10	6011.47	812.88	9.13	36.12	56.42	7.47		
307	307	1347	3/27/2015	34.14621	-93.59637	50	75	1	4	0	188.97	6011.77	814.92	9.21	36.12	56.42	7.47		
307	307	1348	3/27/2015	34.14621	-93.59637	24	90	1	17	0	188.53	6009.38	817.05	8.70	36.12	56.42	7.47		
307	307	1349	3/27/2015	34.14621	-93.59637	52	95	1	27	0	188.53	6009.38	819.39	8.70	36.12	56.42	7.47		
308	308	1350	3/29/2015	34.21942	-93.50683	50	70	1	0	3	123.97	5965.63	78.59	8.86	34.51	58.90	6.59		
308	308	1351	3/29/2015	34.21942	-93.50683	21	35	1	0	3	124.18	5965.79	87.96	7.86	34.51	58.90	6.59		
308	308	1352	3/29/2015	34.21942	-93.50683	9	35	1	2	2	124.66	5965.62	97.25	7.42	34.51	58.90	6.59		
308	308	1353	3/29/2015	34.21942	-93.50683	32	15	1	3	2	124.89	5960.19	106.62	8.80	34.51	58.90	6.59		
308	308	1354	3/29/2015	34.21942	-93.50683	68	5	0	4	0	125.87	5962.59	115.94	7.93	34.51	58.90	6.59		
308	308	1355	3/29/2015	34.21942	-93.50683	2	60	1	3	0	126.10	5967.80	125.31	8.35	34.51	58.90	6.59		
309	309	1356	3/24/2015	34.49177	-92.97778	0	100	0	34	0	115.58	5941.04	64.30	8.05	16.45	63.52	20.03		
309	309	1357	3/24/2015	34.49177	-92.97778	0	100	0	37	0	115.34	5943.56	66.81	6.00	16.45	63.52	20.03		
309	309	1358	3/24/2015	34.49177	-92.97778	0	100	0	48	0	114.90	5944.79	69.93	7.69	16.45	63.52	20.03		
309	309	1359	3/24/2015	34.49177	-92.97778	0	100	0	42	0	115.10	5948.00	73.14	5.71	16.45	63.52	20.03		
309	309	1360	3/24/2015	34.49177	-92.97778	0	100	0	35	0	115.10	5948.00	76.35	5.71	16.45	63.52	20.03		
309	309	1361	3/24/2015	34.49177	-92.97778	0	100	0	72	0	114.24	5952.60	78.56	7.15	16.45	63.52	20.03		

Table D.1 cont'd

Site	Transect	Quadrat	Date	Latitude	Longitude	Canopy	Herb	Sedge	Stem	Burrow	Elevation	Solar	Water_dist	CTI	Sand	Silt	Clay	Burrow ownership	Water_trns
310	310	1362	3/23/2015	34.57099	-92.49532	97	8	1	0	0	109.38	5957.51	445.62	7.74	53.07	40.18	6.75		
310	310	1363	3/23/2015	34.57099	-92.49532	98	7	0	0	0	109.40	5957.06	435.81	8.85	53.07	40.18	6.75		
310	310	1364	3/23/2015	34.57099	-92.49532	100	5	0	1	0	109.47	5957.55	425.98	7.56	53.07	40.18	6.75		
310	310	1365	3/23/2015	34.57099	-92.49532	99	12	0	1	0	109.44	5955.82	416.17	7.52	53.07	40.18	6.75		
310	310	1366	3/23/2015	34.57099	-92.49532	96	5	1	2	0	109.36	5953.11	406.34	7.95	53.07	40.18	6.75		
310	310	1367	3/23/2015	34.57099	-92.49532	78	7	0	0	0	109.18	5947.27	396.53	6.92	53.07	40.18	6.75		
311	311	1368	3/25/2015	34.70194	-93.24039	24	95	1	23	0	223.25	6018.72	259.11	8.44	9.07	73.41	17.52		
311	311	1369	3/25/2015	34.70194	-93.24039	40	90	1	16	0	222.71	6019.38	265.97	8.61	9.07	73.41	17.52		
311	311	1370	3/25/2015	34.70194	-93.24039	40	100	1	47	0	222.90	6016.94	261.23	8.85	9.07	73.41	17.52		
311	311	1371	3/25/2015	34.70194	-93.24039	28	95	0	37	0	222.61	6011.93	256.75	7.61	9.07	73.41	17.52		
311	311	1372	3/25/2015	34.70194	-93.24039	25	100	0	25	0	222.11	5972.26	252.63	6.79	9.07	73.41	17.52		
311	311	1373	3/25/2015	34.70194	-93.24039	30	100	0	54	0	222.11	5972.26	248.85	6.79	9.07	73.41	17.52		
313	313	1374	3/25/2015	34.65171	-92.92240	52	90	0	15	0	188.16	5994.13	259.63	6.12	29.68	59.40	10.92		
313	313	1375	3/25/2015	34.65171	-92.92240	20	85	1	27	0	185.55	5985.64	251.30	6.95	29.68	59.40	10.92		
313	313	1376	3/25/2015	34.65171	-92.92240	24	70	0	12	0	185.08	5995.74	242.95	6.20	29.68	59.40	10.92		
313	313	1377	3/25/2015	34.65171	-92.92240	0	70	0	56	0	184.95	6000.90	234.87	5.63	29.68	59.40	10.92		
313	313	1378	3/25/2015	34.65171	-92.92240	0	80	0	48	0	183.84	6000.90	226.86	6.41	29.68	59.40	10.92		
313	313	1379	3/25/2015	34.65171	-92.92240	1	90	0	64	0	183.85	6000.90	219.05	6.91	29.68	59.40	10.92		
314	314	1380	3/26/2015	34.44160	-93.20860	24	10	0	4	0	170.71	5990.99	257.48	7.55	37.98	48.68	13.35		
314	314	1381	3/26/2015	34.44160	-93.20860	48	98	1	169	0	170.72	5983.36	256.28	6.73	37.98	48.68	13.35		
314	314	1382	3/26/2015	34.44160	-93.20860	24	90	1	47	0	170.21	5968.15	255.36	7.25	37.98	48.68	13.35		
314	314	1383	3/26/2015	34.44160	-93.20860	98	1	0	0	0	169.58	5964.83	254.93	6.72	37.98	48.68	13.35		
314	314	1384	3/26/2015	34.44160	-93.20860	100	1	0	0	0	168.94	5973.70	254.78	7.24	37.98	48.68	13.35		
314	314	1385	3/26/2015	34.44160	-93.20860	100	1	0	0	0	168.40	5983.38	255.14	7.74	37.98	48.68	13.35		
318	318	1386	3/30/2015	34.82550	-93.42265	48	20	0	0	1	241.92	6007.05	88.01	7.95	16.24	72.24	11.52		
318	318	1387	3/30/2015	34.82550	-93.42265	60	70	1	17	0	242.01	6005.04	86.39	7.86	16.24	72.24	11.52		
318	318	1388	3/30/2015	34.82550	-93.42265	93	65	0	14	0	242.11	6003.11	85.39	7.80	16.24	72.24	11.52		

Table D.1 cont'd

Site	Transect	Quadrat	Date	Latitude	Longitude	Canopy	Herb	Sedge	Stem	Burrow	Elevation	Solar	Water_dist	CTI	Sand	Silt	Clay	Burrow ownership	Water_trns
318	318	1389	3/30/2015	34.82550	-93.42265	90	80	1	29	0	242.13	6002.03	84.93	7.77	16.24	72.24	11.52		
318	318	1390	3/30/2015	34.82550	-93.42265	96	55	1	2	0	242.16	6001.64	84.36	7.76	16.24	72.24	11.52		
318	318	1391	3/30/2015	34.82550	-93.42265	88	55	1	0	0	241.73	5998.10	84.06	7.91	16.24	72.24	11.52		
